# Supplementary material for: In situ copper photocatalysts triggering halide atom transfer of unactivated alkyl halides for general C(sp3)-N couplings
Source: Nat Commun. 2024 Jul 5;15:5647. doi: 10.1038/s41467-024-50082-w (PMC11226431; doi:10.1038/s41467-024-50082-w)
Supplement: Supplementary file 1 — Supplementary Information [file 41467_2024_50082_MOESM1_ESM.pdf]

Supplementary information

In situ copper photocatalysts triggering halide atom transfer of unactivated alkyl halides for general C(sp3)-N Couplings

Hang Luo[1], Yupeng Yang[2], Yukang Fu[3], Fangnian Yu[1], Lei Gao[1], Yunpeng Ma[1], Yang Li[3]*, Kaifeng Wu[2]*, Luqing Lin[1]*

[1]School of Chemistry, Dalian University of Technology, Dalian, Liaoning 116024, China.

[2]State Key Laboratory of Molecular Reaction Dynamics and Collaborative Innovation Center of Chemistry for Energy Materials (iChEM), Dalian Institute of Chemical Physics, Chinese Academy of Sciences Dalian, Liaoning 116023, China.

[3]State Key Laboratory of Fine Chemicals, School of Chemical Engineering, Dalian University of Technology, Liaoning 116024, China.

These authors contributed equally: Hang Luo, Yupeng Yang, Yukang Fu.

Email: chyangli@dlut.edu.cn, kwu@dicp.ac.cn, linluqing@dlut.edu.cn.

Table of Contents

Supplementary Methods **S4**

1. General Information **S4**

1.1 Materials and methods S4

2. Starting Material Synthesis **S5**

1.1 General procedure of the synthesis of alkyl iodides S5

1.2 General procedure of the synthesis of alkyl bromides S10

1.3 General procedure of the synthesis of Pyridyl-NHC ligands S11

3. Experimental details of C(sp3)-N couplings **S13**

3.1 Reaction optimization of C(sp3)-N couplings S13

3.2 General procedure for C(sp3)-N couplings S16

3.3 Scale up reaction S17

3.4 Characterization data of amination products S18

4. Mechanistic Studies **S42**

A. Investigating into the active Cu(I) species during photocatalytic process **S42**

4.1 UV-vis absorption of in-situ generated copper complex S42

4.2 Synthesis of pyridyl-carbene ligated copper(I) complex ex situ S43

4.3 Electronchemical of $Cu(Py\text{-}NHC)_2PF_6$ S45

4.4 HRMS analysis of $Cu(Py\text{-}NHC)_2PF_6$ and in situ generated copper complex S45

4.5 ^1H NMR analysis of in-situ generated copper complex S47

4.6 The reactivity of $Cu(Py\text{-}NHC)_2PF_6$ for C-N coupling S48

B. Alkyl radical species trapping experiments **S49**

4.7 TEMPO trapping experiments S49

4.8 Radical clock experiments: involvement of alkyl radicals S49

C. Investigation of Cu(II) intermediate for C-N coupling **S51**

4.9 Exploration of copper(II) as the one of possible intermediates S51

4.10 EPR data collection and analysis S51

D. Inner-Sphere SET Process Investigation **S53**

4.11 Transient absorption experiments S53

4.12 Lifetime quenching experiments S55

4.13 Quenching analysis S56

4.14 Estimated excited-state oxidation potential of in situ generated Cu-1 complex S58

4.15 The exploration of interactions between in-situ generated Cu-1 complex with alkyl iodides on ground state with ^{1}H NMR and UV-vis spectra S59

E. DFT calculation S60

4.16 Computational details S60

F Other attempted couplings S61

5. Copies of NMR Spectra S63

Supplementary References S149

6. Reference S149

Supplementary Methods

1. General Information

1.1 Materials and methods

Unless otherwise noted, all the materials were commercially available and used without further purification. All solvents were dried before use according to the standard methods. All reactions were performed in an N_2-filled glovebox using standard Schlenk techniques unless otherwise noted. All reactions were monitored by thin-layer chromatography (TLC), visualized by UV, Ninhydrin and $KMnO_4$ staining. Chromatographic purification of products was accomplished by silica gel chromatography. ^{1}H NMR, ^{13}C NMR and ^{19}F NMR, ^{31}P NMR spectra were recorded on a Bruker Avance II 400. NMR data is reported relative to internal $CHCl_3$ (^{1}H, δ = 7.26), $CDCl_3$ (^{13}C, δ = 77.0), CH_2Cl_2 (^{1}H, δ = 5.30), CD_2Cl_2 (^{13}C, δ = 53.0), DMSO (^{1}H, δ = 2.50), DMSO-*d6* (^{13}C, δ = 39.5), CH_3CN (^{1}H, δ = 1.94), CH_3CN-*d3* (^{13}C, δ = 1.3, 118.3). Data for ^{1}H NMR spectra are reported as follows: chemical shift (δ) in ppm; multiplicities are indicated s (singlet), brs (broad singlet), d (doublet), t (triplet), m (multiplet); coupling constants (J) are in Hertz (Hz) ^{13}C NMR spectra were reported as chemical shifts in ppm. The HRMS were obtained by using a Q Exactive high resolution liquid chromatography mass spectrometer (Q Exactive Plus) in ESI^+ mode or ESI^- mode. UV/Vis spectra were recorded using Shanghai Yoke T-UV756 instrument for samples in solution. The eight-position parallel light reaction system (RLH-18) and the large volume light reaction system (RLH-054) with 410 nm LEDs were purchased from Beijing Roger Technologies. The fluorescence emission spectra were collected on an Edinburgh Analytical Instruments FLS 1000. EPR data were collect on Bruke ELEXSYS-II E 500 CW-EPR.

2. Starting Material Synthesis

1.1 General procedure of the synthesis of alkyl iodides

General procedure A (GPA)

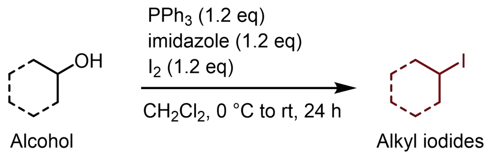

To a solution of the PPh_3 (1.2 equiv), Imidazole (1.2 equiv) and I_2 (1.2 equiv) in dry CH_2Cl_2 (0.3 M) was added alcohol (1.0 equiv) portionwise at 0 °C. The reaction was stirred for additional 12 hours at room temperature. The progress of the reactions was monitored by TLC. After the reaction was completed, the mixture was washed with a solution of $Na_2S_2O_3$, H_2O, brine, dried over Na_2SO_4, filtered and concentrated under reduced pressure. The resulting alkyl iodide was purified by flash chromatography.

Iodocycloheptane (S1)

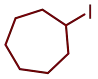

Prepared by **GPA**. All other spectroscopic analyses were in agreement with the literature[1].

^{1}H NMR (400 MHz, $CDCl_3$): δ 4.48 (tt, J = 8.8, 4.4 Hz, 1H), 2.33 – 2.23 (m, 2H), 2.21 – 2.10 (m, 2H), 1.58 (d, J = 7.2 Hz, 6H), 1.49 – 1.39 (m, 2H).

8-iodo-1,4-dioxaspiro[4.5]decane (S2)

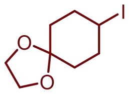

Prepared by **GPA**. All other spectroscopic analyses were in agreement with the literature[1].

^{1}H NMR (400 MHz, $CDCl_3$): δ 4.42 (brs, 1H), 3.99 – 3.89 (m, 4H), 2.21 – 2.03 (m, 4H), 1.84 – 1.76 (m, 2H), 1.67 – 1.56 (m, 2H).
All other spectroscopic analyses were in agreement with the literature[1].

1,1-difluoro-4-iodocyclohexane (S3)

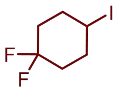

Prepared by **GPA**. All other spectroscopic analyses were in agreement with the literature[2].

^{1}H NMR (400 MHz, CDCl$_3$): δ 4.47 (brs, 1H), 2.20 – 2.09 (m, 4H), 2.09 – 2.00 (m, 2H), 1.97 – 1.86 (m, 2H).

3-iodo-1-tosylpyrrolidine (S4)

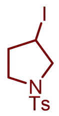

Prepared by **GPA**. All other spectroscopic analyses were in agreement with the literature[3].

^{1}H NMR (400 MHz, CDCl$_3$): δ 7.74 (d, *J* = 8.0 Hz, 2H), 7.34 (d, *J* = 8.0 Hz, 2H), 4.22 – 4.12 (m, 1H), 3.90 (dd, *J* = 11.7, 5.8 Hz, 1H), 3.56 (dd, *J* = 11.7, 4.8 Hz, 1H), 3.46 – 3.41 (m, 2H), 2.44 (s, 3H), 2.31 – 2.21 (m, 1H), 2.17 – 2.08 (m, 1H).

4-Iodotetrahydro-2H-thiopyran (S5)

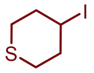

Prepared by **GPA**. All other spectroscopic analyses were in agreement with the literature[2].

^{1}H NMR (400 MHz, CDCl$_3$): δ 4.54 – 4.42 (m, 1H), 2.85 – 2.73 (m, 2H), 2.61 – 2.52 (m, 2H), 2.36 – 2.26 (m, 4H).

***tert*-Butyl 6-iodo-2-azaspiro[3.3]heptane-2-carboxylate (S6)**

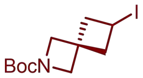

Prepared by **GPA**. All other spectroscopic analyses were in agreement with the literature[2].

^{1}H NMR (400 MHz, CDCl$_3$): δ 4.29 (p, *J* = 7.8 Hz, 1H), 3.93 (d, *J* = 11.8 Hz, 4H), 2.95 – 2.87 (m, 2H), 2.75 – 2.66 (m, 2H), 1.42 (s, 9H).

***tert*-Butyl 2-Iodo-7-azaspiro[3.5]nonane-7-carboxylate (S7)**

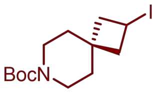

Prepared by **GPA**. All other spectroscopic analyses were in agreement with the literature[2].

^{1}H NMR (400 MHz, CDCl$_3$): δ 4.49 (p, *J* = 8.3 Hz, 1H), 3.33 – 3.23 (m, 4H), 2.69 – 2.61 (m, 2H), 2.45 – 2.37 (m, 2H), 1.68 – 1.63 (m, 2H), 1.57 – 1.53 (m, 2H), 1.43 (s, 9H).

Methyl 3-Iodocyclobutane-1-carboxylate (S8)

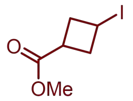

Prepared by **GPA**, dr = 2:1. All other spectroscopic analyses were in agreement with the literature[2].

^{1}H NMR (400 MHz, CDCl$_3$): δ 4.64 (tt, *J* = 7.2 Hz, 1H), 3.68 (s, 3H), 3.44 – 3.33 (m, 1H), 2.99 – 2.84 (m, 4H).

3-iodoheptane (S9)

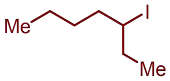

Prepared by **GPA**.

^{1}H NMR (400 MHz, CDCl$_3$): δ 4.09 (tt, *J* = 8.7, 4.7 Hz, 1H), 1.94 – 1.63 (m, 4H), 1.54 – 1.23 (m, 4H), 1.02 (t, *J* = 7.2 Hz, 3H), 0.91 (t, *J* = 7.0 Hz, 3H).
^{13}C NMR (101 MHz, CDCl$_3$): δ 42.6, 40.0, 33.7, 31.7, 21.9, 14.1, 13.9.
HRMS (ESI): Calcd for $C_7H_{16}I$ $[M+H]^+$:227.0291, found 227.0285

(2-iodopropoxy)benzene (S10)

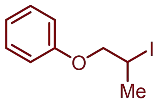

Prepared by **GPA**. All other spectroscopic analyses were in agreement with the literature[4].

^{1}H NMR (400 MHz, CDCl$_3$): δ 7.35 – 7.27 (m, 2H), 6.99 (t, *J* = 7.4 Hz, 1H), 6.92 (d, *J* = 8.0 Hz, 2H), 4.42 – 4.32 (m, 1H), 4.26 (dd, *J* = 10.0, 5.6 Hz, 1H), 4.05 (dd, *J* = 9.9, 7.9 Hz, 1H), 2.01 (d, *J* = 6.8 Hz, 3H).

4-(1-iodoethyl)tetrahydro-2H-pyran (S11)

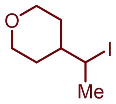

Prepared by **GPA**.

^{1}H NMR (400 MHz, CDCl$_3$): δ 4.19 – 4.08 (m, 1H), 4.02 – 3.93 (m, 2H), 3.44 – 3.33 (m, 2H), 1.91 (d, *J* = 7.0 Hz, 3H), 1.78 – 1.66 (m, 2H), 1.47 – 1.36 (m, 3H).
^{13}C NMR (101 MHz, CDCl$_3$): δ 67.8, 67.4, 44.2, 37.2, 32.3, 31.5, 25.4.
HRMS (ESI): Calcd for $C_7H_{14}IO$ $[M+H]^+$:241.0084, found 241.0077

6-iodohexahydro-2H-3,5-methanocyclopenta[b]furan-2-one (S12)

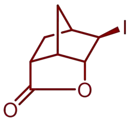

Prepared by reported literature[2]. All other spectroscopic analyses were in agreement with the literature[2].

^{1}H NMR (400 MHz, CDCl$_3$): δ 5.14 (d, J = 5.1 Hz, 1H), 3.92 – 3.87 (m, 1H), 3.20 (t, J = 4.7 Hz, 1H), 2.73 (d, J = 3.9 Hz, 1H), 2.58 (dd, J = 11.3, 4.4 Hz, 1H), 2.39 (dd, J = 11.6, 2.1 Hz, 1H), 2.08 (ddd, J = 13.6, 11.2, 4.0 Hz, 1H), 1.92 – 1.80 (m, 2H).

***tert*-Butyl 3-exo-Iodo-8-azabicyclo[3.2.1]octane-8-carboxylate (S13)**

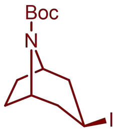

Prepared by **GPA**. All other spectroscopic analyses were in agreement with the literature[2].

^{1}H NMR (400 MHz, CDCl$_3$): δ 4.52 (tt, J = 11.9, 5.7 Hz, 1H), 4.13 – 3.94 (m, 2H), 2.43 – 2.24 (m, 2H), 2.22 – 2.16 (m, 2H), 1.94 – 1.89 (m, 2H), 1.68 – 1.61 (m, 2H), 1.48 (s, 9H).

(1S,2S,4R)-2-iodo-1-isopropyl-4-methylcyclohexane (S14)

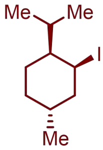

Prepared by **GPA**. All other spectroscopic analyses were in agreement with the literature[5].

^{1}H NMR (400 MHz, CDCl$_3$): δ 4.77 (s, 1H), 2.25 – 2.18 (m, 1H), 2.02 – 1.91 (m, 1H), 1.81 – 1.70 (m, 2H), 1.41 – 1.20 (m, 4H), 0.98 – 0.85 (m, 9H).

1-fluoro-2-iodocyclohexane (S15)

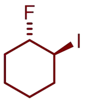

Prepared by reported literature[2]. All other spectroscopic analyses were in agreement with the literature[2].

^{1}H NMR (400 MHz, CDCl$_3$): δ 4.66 – 4.45 (m, 1H), 4.16 – 4.06 (m, 1H), 2.45 – 2.33 (m, 1H), 2.27 – 2.15 (m, 1H), 2.00 – 1.89 (m, 1H), 1.87 – 1.79 (m, 1H), 1.60 – 1.53 (m, 1H), 1.41 – 1.19 (m, 3H).

1-ethoxy-2-iodocyclohexane (S16)

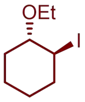

Prepared by reported literature[2]. All other spectroscopic analyses were in agreement with the literature[2].

^{1}H NMR (400 MHz, CDCl$_3$): δ 4.09 – 4.01 (m, 1H), 3.70 – 3.49 (m, 2H), 3.35 – 3.27 (m, 1H), 2.46 – 2.38 (m, 1H), 2.17 – 2.10 (m, 1H), 2.03 – 1.91 (m, 1H), 1.85 – 1.77 (m, 1H), 1.56 – 1.50 (m, 1H), 1.37 – 1.27 (m, 3H), 1.23 (t, J = 7.0 Hz, 3H).

(3aR,5S,5aR,8aS,8bR)-5-(iodomethyl)-2,2,7,7-tetramethyltetrahydro-5H-bis([1,3]dioxolo)[4,5-b:4',5'-d]pyran (S19)

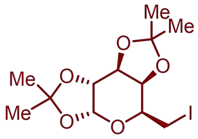

Prepared by **GPA**. All other spectroscopic analyses were in agreement with the literature[6].

^{1}H NMR (400 MHz, CDCl$_3$): δ 5.54 (d, J = 5.0 Hz, 1H), 4.61 (d, J = 7.8 Hz, 1H), 4.40 (d, J = 7.8 Hz, 1H), 4.31 – 4.27 (m, 1H), 3.94 (t, J = 7.0 Hz, 1H), 3.35 – 3.27 (m, 1H), 3.24 – 3.16 (m, 1H), 1.54 (s, 3H), 1.44 (s, 3H), 1.35 (s, 3H), 1.33 (s, 3H).

1-((3R,8S,9S,10R,13S,14S,17S)-3-iodo-10,13-dimethyl-2,3,4,7,8,9,10,11,12,13,14,15,16,17-tetradecahydro-1H-cyclopenta[a]phenanthren-17-yl)ethan-1-one (S20)

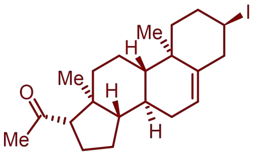

Prepared by **GPA**. All other spectroscopic analyses were in agreement with the literature[7].

^{1}H NMR (400 MHz, CDCl$_3$): δ 5.35 – 5.31 (m, 1H), 4.09 – 3.97 (m, 1H), 2.98 – 2.87 (m, 1H), 2.73 – 2.64 (m, 1H), 2.56 – 2.48 (m, 1H), 2.29 – 2.15 (m, 3H), 2.12 (s, 3H), 2.02 – 1.94 (m, 2H), 1.79 – 1.63 (m, 3H), 1.55 – 1.40 (m, 5H), 1.28 – 1.11 (m, 3H), 1.04 (s, 3H), 0.99 – 0.94 (m, 1H), 0.62 (s, 3H).

***N*-allyl-N-(2-iodoethyl)-4-methylbenzenesulfonamide (S21)**

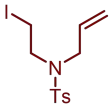

Prepared by **GPA**. All other spectroscopic analyses were in agreement with the literature[8].

^{1}H NMR (400 MHz, CDCl$_3$): δ 7.70 (d, J = 8.3 Hz, 2H), 7.32 (d, J = 8.0 Hz, 2H), 5.74 – 5.62 (m, 1H), 5.22 – 5.16 (m, 2H), 3.79 (d, J = 6.5 Hz, 2H), 3.45 – 3.39 (m, 2H), 3.26 – 3.21 (m, 2H), 2.44 (s, 3H).

(iodomethyl)cyclopropane (S22)

Prepared by **GPA**. All other spectroscopic analyses were in agreement with the literature[9].

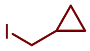

^{1}H NMR (400 MHz, $CDCl_3$): δ 3.13 (d, *J* = 7.7 Hz, 2H), 1.37 – 1.23 (m, 1H), 0.91 – 0.78 (m, 2H), 0.35 – 0.28 (m, 2H).

1.2 General procedure of the synthesis of alkyl bromides

General procedure B (GPB)

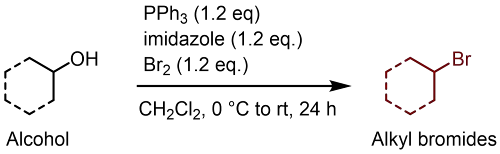

Br_2 (1.2 equiv) was added to a suspension of PPh_3 (1.2 equiv)in dry CH_2Cl_2 (0.3 M) at 0 °C. The mixture was stirred for 10 min and a white solid will precipitate from mixture. And then imidazole (1.2 equiv) was added to the mixture, the solution become clean again. After that alcohol (1.0 equiv) was added portionwise at 0 °C. The reaction was stirred for additional 12 hours at room temperature. The progress of the reactions was monitored by TLC. After the reaction was completed, the mixture was washed with a solution of $Na_2S_2O_3$, H_2O, brine, dried over Na_2SO_4, filtered and concentrated under reduced pressure. The resulting alkyl bromides was purified by flash chromatography.

Bromocycloheptane (S17)

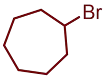

Prepared by **GPB,** Spectral data match the reported literature values[10].

^{1}H NMR (400 MHz, $CDCl_3$): δ 4.33 (tt, *J* = 8.7, 4.3 Hz, 1H), 2.32 – 2.22 (m, 2H), 2.10 – 2.00 (m, 2H), 1.78 – 1.49 (m, 8H).

(2-bromopropoxy)benzene (S18)

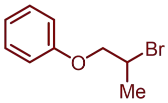

Prepared by **GPB**.

^{1}H NMR (400 MHz, $CDCl_3$): δ 7.37 – 7.27 (m, 2H), 7.04 – 6.97 (m, 1H), 6.93 (d, *J* = 7.8 Hz, 2H), 4.41 – 4.29 (m, 1H), 4.24 (dd, *J* = 9.9, 5.6 Hz, 1H), 4.07 (dd, *J* = 9.9, 7.2 Hz, 1H), 1.82 (d, *J* = 6.7 Hz, 3H).
^{13}C NMR (101 MHz, $CDCl_3$): δ 158.1, 129.5, 121.3, 114.7, 73.0, 45.3, 22.7.

HRMS (ESI): Calcd for $C_9H_{12}BrO$ $[M+H]^+$:215.0066, found 215.0070

1.3 General procedure of the synthesis of Pyridyl-NHC ligands

General Procedure C (GPC)

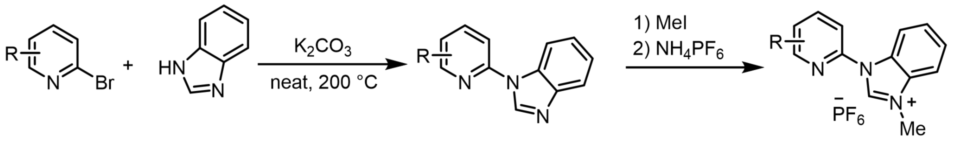

Step 1: substituted 2-bromopyridine (1.0 equiv), benzimidazole (2.2 equiv) and K_2CO_3 (2.0 equiv) were stirred in a seal tube at 200°C for 24 h. After cooling to room temperature, quenched with H_2O and extracted with ethyl acetate. The combined organic layers were dried over anhydrous Na_2SO_4, concentrated in vacuo, then purified by silica gel column chromatography to afford the product.
Step 2: substituted benzimidazole and MeI (10 M) were stirred in a seal tube at 100°C for 2-12 h. After cooling to room temperature resulting yellow solid was washed with ethyl acetate for several times and then dissolved in deionized water, followed by ion exchange with saturated aqueous NH_4PF_6 to afford **L1, L2, L3** as white solid.

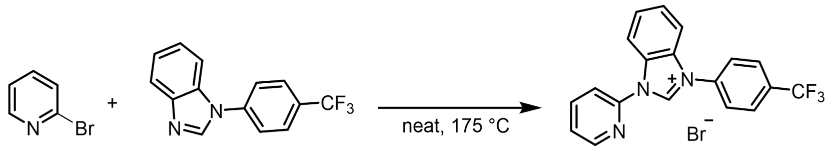

L4

Experiments procedure: 2-bromopyridine (1.0 equiv), benzimidazole (1.5 equiv) were stirred in a seal tube at 200 °C for 3 days. After cooling to room temperature, the resulting mixture was washed with Et_2O for several times until no starting materials were detectable by TLC, then recrystallized from Et_2O/DCM to afford **L4** as brown solid.

3-methyl-1-(pyridin-2-yl)-1H-benzo[d]imidazol-3-ium hexafluorophosphate (L1)

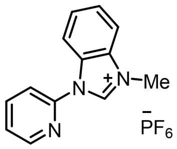

Prepared by **GPC**. All other spectroscopic analyses were in agreement with the literature[11].

^{1}H NMR (400 MHz, DMSO-*d*6): δ 10.47 (s, 1H), 8.79 (d, *J* = 4.2 Hz, 1H), 8.51 – 8.45 (m, 1H), 8.29 (t, *J* = 7.3 Hz, 1H), 8.15 (dd, *J* = 5.7, 3.1 Hz, 1H), 8.03 (d, *J* = 8.1 Hz, 1H), 7.84 – 7.69 (m, 3H), 4.20 (s, 3H).
^{13}C NMR (101 MHz, DMSO-*d*6): δ 149.5, 147.4, 142.8, 140.6, 132.3, 129.4, 127.7, 127.1, 125.1, 116.8, 115.8, 114.0, 33.7.

^{19}**F NMR (377 MHz, DMSO-*d*$_6$):** δ -70.1 (d, *J* = 711.5 Hz).
^{31}**P NMR (162 MHz, DMSO-*d*$_6$):** δ -144.2 (hept, *J* = 712.8 Hz)
HRMS (ESI): Calcd for $C_{13}H_{12}N_3$ $[M-PF_6]^+$:210.1026, found 210.1020

3-methyl-1-(4-methylpyridin-2-yl)-1H-benzo[d]imidazol-3-ium hexafluorophosphate (L2)

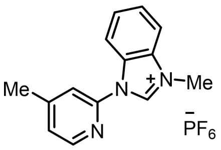

Prepared by **GPC**.

^{1}**H NMR (400 MHz, DMSO-*d*$_6$):** δ 10.44 (s, 1H), 8.63 (d, *J* = 4.7 Hz, 1H), 8.48 (d, *J* = 7.2 Hz, 1H), 8.13 (d, *J* = 6.9 Hz, 1H), 7.89 (s, 1H), 7.81 – 7.76 (m, 2H), 7.55 (d, *J* = 4.3 Hz, 1H), 4.19 (s, 3H), 2.54 (s, 3H).
^{13}**C NMR (101 MHz, DMSO-*d*$_6$):** δ 152.0, 149.1, 147.4, 142.7, 132.3, 129.3, 127.7, 127.1, 125.8, 117.1, 115.8, 113.9, 33.7, 20.8.
^{19}**F NMR (377 MHz, DMSO-*d*$_6$):** δ -70.1 (d, *J* = 711.5 Hz).
^{31}**P NMR (162 MHz, DMSO-*d*$_6$):** δ -144.2 (hept, *J* = 712.8 Hz)
HRMS (ESI): Calcd for $C_{14}H_{14}N_3$ $[M-PF_6]^+$:224.1182, found 224.1176

3-methyl-1-(4-(trifluoromethyl)pyridin-2-yl)-1H-benzo[d]imidazol-3-ium hexafluorophosphate (L3)

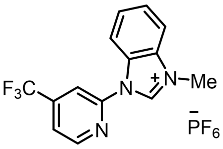

Prepared by **GPC**.

^{1}**H NMR (400 MHz, DMSO-*d*$_6$):** δ 10.65 (s, 1H), 9.07 (s, 1H), 8.57 (s, 1H), 8.47 (s, 1H), 8.15 (d, *J* = 16.4 Hz, 2H), 7.82 (s, 2H), 4.22 (s, 3H).
^{13}**C NMR (101 MHz, DMSO-*d*$_6$):** δ 151.2, 148.7, 143.5, 140.0 (q, *J* = 33.5 Hz), 132.2, 129.3, 127.9, 127.3, 122.3 (q, *J* = 274.7 Hz), 120.4 (q, *J* = 274.7 Hz), 116.2, 113.1 (q, *J* = 3.7 Hz), 114.0, 33.9.
^{19}**F NMR (377 MHz, DMSO-*d*$_6$)** δ -63.3, -70.2 (d, *J* = 711.0 Hz).
^{31}**P NMR (162 MHz, DMSO-*d*$_6$):** δ -144.2 (hept, *J* = 712.8 Hz)
HRMS (ESI): Calcd for $C_{14}H_{11}F_3N_3$ $[M-PF_6]^+$:278.0900, found 278.0893

1-(pyridin-2-yl)-3-(4-(trifluoromethyl)phenyl)-1H-benzo[d]imidazol-3-ium bromide (L4)

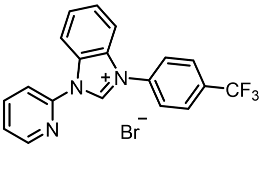

Prepared by experiments procedure.

^{1}H NMR (400 MHz, DMSO-*d*$_6$): δ 11.03 (s, 1H), 8.85 (d, *J* = 4.0 Hz, 1H), 8.64 (d, *J* = 8.1 Hz, 1H), 8.35 (td, *J* = 8.0, 1.6 Hz, 1H), 8.28 – 8.21 (m, 5H), 8.00 (d, *J* = 8.2 Hz, 1H), 7.90 – 7.76 (m, 3H).
^{13}C NMR (101 MHz, DMSO-*d*$_6$): δ 149.5, 147.4, 143.1, 140.7, 139.7, 136.3, 131.5, 130.9 (q, *J* = 32.2 Hz), 129.7, 128.1 (q, *J* = 6.1 Hz), 127.6 (q, *J* = 3.7 Hz), 126.9, 125.5, 123.7 (q, *J* = 272.5 Hz), 117.6, 116.6, 113.8.
^{19}F NMR (377 MHz, DMSO-*d*$_6$): δ -61.2.
HRMS (ESI): Calcd for $C_{19}H_{13}F_3N_3$ $[M-Br]^+$:340.1056, found 340.1048

3-methyl-1-(quinolin-8-yl)-1H-benzo[d]imidazol-3-ium hexafluorophosphate (L5)

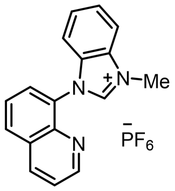

Prepared by reported procedure[12]. All other spectroscopic analyses were in agreement with the literature[12].

^{1}H NMR (400 MHz, DMSO-*d*$_6$): δ 10.21 (s, 1H), 8.94 (d, *J* = 3.2 Hz, 1H), 8.69 (d, *J* = 8.1 Hz, 1H), 8.42 (d, *J* = 8.2 Hz, 1H), 8.30 (d, *J* = 7.3 Hz, 1H), 8.19 (d, *J* = 8.3 Hz, 1H), 7.97 (t, *J* = 7.8 Hz, 1H), 7.79 (t, *J* = 7.1 Hz, 2H), 7.66 (t, *J* = 7.8 Hz, 1H), 7.54 (d, *J* = 8.3 Hz, 1H), 4.28 (s, 3H).
^{13}C NMR (101 MHz, DMSO-*d*$_6$): δ 152.1, 144.4, 141.7, 137.1, 132.4, 131.5, 131.5, 129.5, 129.0, 128.3, 127.3, 126.8, 126.7, 123.2, 113.9, 113.9, 33.7.
^{19}F NMR (377 MHz, DMSO-*d*$_6$) δ -70.1 (d, *J* = 711.6 Hz).
^{31}P NMR (162 MHz, DMSO-*d*$_6$): δ -143.1 (hept, *J* = 712.8 Hz)
HRMS (ESI): Calcd for $C_{17}H_{14}N_3$ $[M-PF_6]^+$:260.1182, found 260.1178

3. Experimental details of C(sp3)-N couplings

3.1 Reaction optimization of C(sp3)-N couplings

Procedure for optimization : To an oven-dried 10 mL reaction vial were added Cu salt , ligand, and 1 mL solvent in a nitrogen-filled glove box. The resulting mixture was stirred for 5 min, followed by adding base, *p*-toluidine and *tert*-butyl 4-iodopiperidine-1-carboxylate in sequence, and sealed with a screwed cap. The sealed vial was placed on a photo-reactor under irradiation of 6W LEDs. The mixture was stirred at 25 °C for 15 h, quenched with H_2O, and extracted with ethyl acetate. The combined organic layers were dried over anhydrous Na_2SO_4, concentrated in vacuo. The crude product was analyzed by ^{1}H NMR with 1,3,5-Trimethoxybenzene as the internal standard.

Supplementary Table 1. Base effect

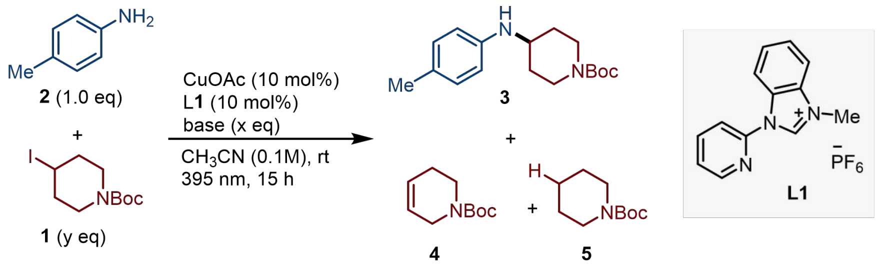

entry	base	additive	x	y	**3**(%)	**4**(%)	**5**(%)
1	LiO*t*Bu	-	2.5	1.5	21	16	28
2	LiO*t*Bu	-	2.5	2.0	25	21	42
3	LiO*t*Bu	-	1.8	1.5	23	10	29
4	LiO*t*Bu	H_2O (10 eq)	1.8	1.5	0	<1	0
5	LiOH•H_2O	-	1.8	1.5	0	0	9
6	TMG	-	1.8	1.5	0	23	21
7	BTMG	-	1.8	1.5	73	16	-
8	DBU	-	1.8	1.5	0	-	-

Supplementary Table 2. Light and base effect

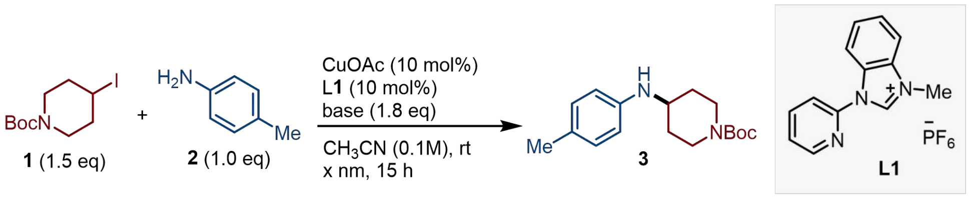

entry	base	x (nm)	**3**(%)
1	BTMG	395	73
2	BTMG	410	89
3	BTMG	430	34
4	BTMG	450	25
5	MTBD	410	95

Supplementary Table 3. Light and catalyst loading effect

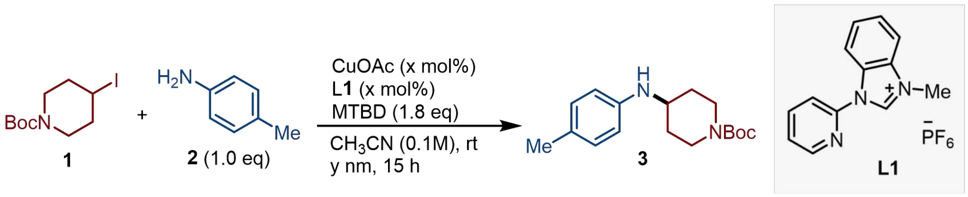

entry	**1** (eq)	x	y	**3**(%)
1	1.5	10	410	95
2	1.5	5	410	94
3	1.5	5	395	92
4	1.5	5	450	45
5	1.2	10	410	92

Supplementary Table 4. Other effect and control experiments

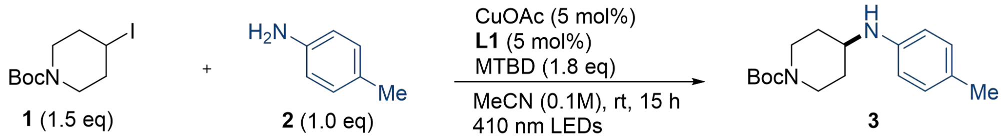

Entry	Variants from standard conditions	Yield (%)[b]
1	None	94(93)[c]
2	**L2** instead of **L1**	94
3	**L3** instead of **L1**	75
4	**L4** instead of **L1**	91
5	**L5** instead of **L1**	trace
6	*t*BuOLi instead of MTBD	47
7	BTMG instead of MTBD	30
8	DBU instead of MTBD	N.D.
9	DIPEA instead of MTBD	N.D.
10	DMF instead of CH_3CN	70
11	THF instead of CH_3CN	80
12	MeOH instead of CH_3CN	N.D.
13	CuI instead of CuOAc	93
14	CuBr instead of CuOAc	93
15	CuCl instead of CuOAc	30
16	$Cu(CH_3CN)_4PF_6$ instead of CuOAc	20
17	Without L1	N.D.
18	In dark	N.D
19	Without MTBD or CuOAc	N.D
20	(N,N)-Cu instead of L1 and CuOAc	N.D.

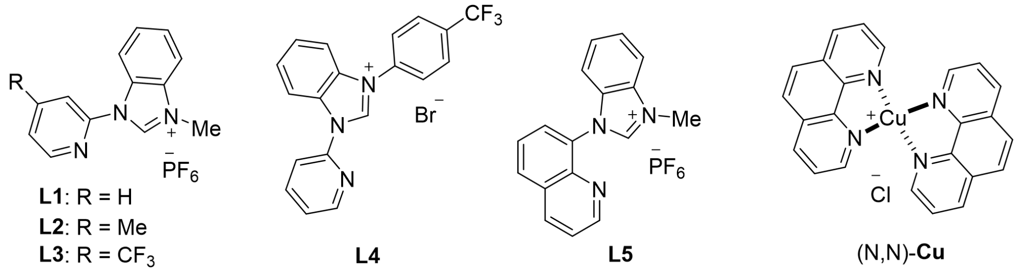

3.2 General procedure for C(sp3)-N couplings

General procedure D (GPD)

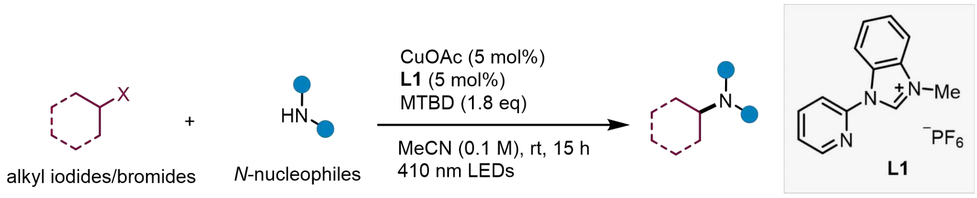

To an oven-dried 10 mL reaction vial were added CuOAc (0.6 mg, 0.005 mmol, 5 mol%), L1 (1.8 mg, 0.005 mmol, 5 mol%), and 1 mL MeCN in a nitrogen-filled glove box. The resulting mixture was stirred for 5 min, followed by adding 7-Methyl-1,5,7-triazabicyclo[4.4.0]dec-5-ene (MTBD) (26 µL, 0.18 mmol, 1.8 equiv), *N*-nucleophiles (0.1 mmol, 1.0 equiv) and alkyl iodides/bromides (0.15 mmol, 1.5 equiv) in sequence, and sealed with a screwed cap. The sealed vial was placed on a photo-reactor under irradiation of LEDs (410 nm, 6 W). The mixture was stirred at 20 ~ 25 °C for 15 h, quenched with H_2O, and extracted with ethyl acetate. The combined organic layers were dried over anhydrous Na_2SO_4, concentrated in vacuo. The crude product was purified by silica gel column chromatography to afford the coupling product.

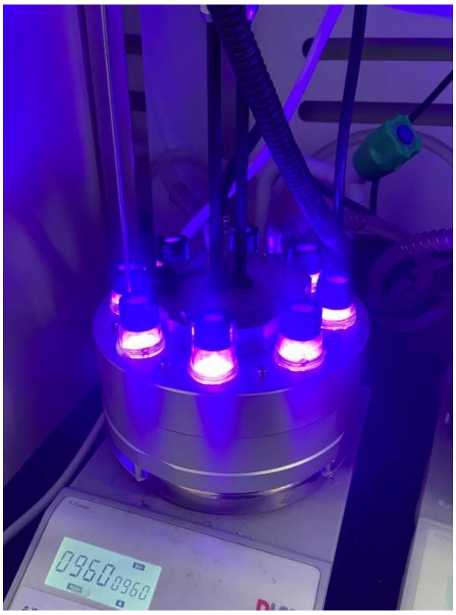

Supplementary Figure 1. The eight-position parallel light reaction system (RLH-18)

3.3 Scale up reaction

General procedure for scale up reaction

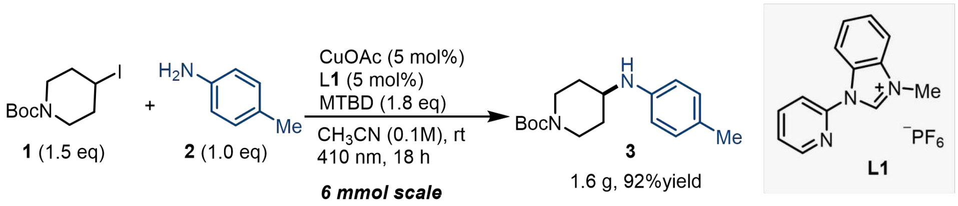

6 mmol scale for synthesis of 3:

To an oven-dried 250 mL round-bottomed flask were added CuOAc (36.6 mg, 0.30 mmol, 5 mol%), **L1** (106.6 mg, 0.30 mmol, 5 mol%), and 60 mL MeCN in a nitrogen-filled glove box. The resulting mixture was stirred for 10 min, followed by adding 7-Methyl-1,5,7-triazabicyclo[4.4.0]dec-5-ene (MTBD) (1.55 mL, 10.8 mmol, 1.8 equiv), *p*-toluidine (642.9 mg, 6.0 mmol, 1.0 equiv) and *tert*-butyl 4-iodopiperidine-1-carboxylate (2.80 g, 9.0 mmol, 1.5 equiv) in sequence, and sealed with a screwed cap. The sealed flask was placed on a large volume photo-reactor under irradiation of LEDs (410 nm, 50 W × 2). The mixture was stirred at 25 °C for 18 h, quenched with H_2O, and extracted with ethyl acetate. The combined organic layers were dried over anhydrous Na_2SO_4, concentrated in vacuo. The crude product was purified by silica gel column chromatography to afford the coupling product **3** (1.60 g, 92% yield).

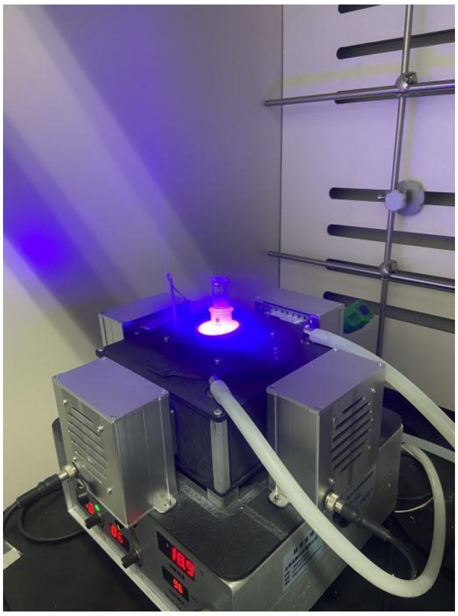

Supplementary Figure 2. The large volume light reaction system (RLH-054)

3.4 Characterization data of amination products

***tert*-butyl 4-(*p*-tolylamino)piperidine-1-carboxylate (3)**

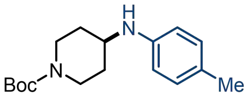

Prepared according to the **GPD** from *p*-toluidine (10.7 mg, 0.1 mmol) and *tert*-butyl 4-iodopiperidine-1-carboxylate (46.7 mg, 0.15 mmol), The crude residue was purified by column chromatography to yield **3** (27.2 mg, 93% yield) as a white solid.

^{1}H NMR (400 MHz, CDCl$_3$): δ 6.98 (d, *J* = 8.3 Hz, 2H), 6.53 (d, *J* = 8.4 Hz, 2H), 4.04 (brs, 2H), 3.44 – 3.34 (m, 1H), 2.91 (t, *J* = 12.7 Hz, 2H), 2.23 (s, 3H), 2.03 (d, *J* = 14.5 Hz, 2H), 1.46 (s, 9H), 1.36 – 1.28 (m, 2H).
^{13}C NMR (101 MHz, CDCl$_3$): δ 154.8, 144.4, 129.8, 126.8, 113.6, 79.5, 50.4, 42.7, 32.4, 28.4, 20.3.
HRMS (ESI): Calcd for $C_{17}H_{26}N_2O_2^+$ [M+H]$^+$:291.2067, found 291.2060

***tert*-butyl 4-((3,5-bis(trifluoromethyl)phenyl)amino)piperidine-1-carboxylate (4)**

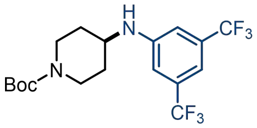

Prepared according to the **GPD** from 3,5-bis(trifluoromethyl)aniline (22.9 mg, 0.1 mmol) and *tert*-butyl 4-iodopiperidine-1-carboxylate (46.7 mg, 0.15 mmol), The crude residue was purified by column chromatography to yield **4** (38.8 mg, 94% yield) as a white solid.

^{1}H NMR (400 MHz, CDCl$_3$): δ 7.13 (s, 1H), 6.91 (s, 2H), 4.14 – 3.99 (m, 3H), 3.53 – 3.43 (m, 1H), 2.96 (t, *J* = 12.5 Hz, 2H), 2.05 – 1.99 (m, 2H), 1.47 (s, 9H), 1.42 – 1.32 (m, 2H).
^{13}C NMR (101 MHz, CDCl$_3$): δ 154.7, 147.4, 132.5 (q, *J* = 32.7 Hz), 123.1 (q, *J* = 273.7), 112.0 (q, *J* = 10.1 Hz), 110.1 (q, *J* = 10.1 Hz), 79.9, 49.9, 42.6, 31.9, 28.4.
^{19}F NMR (377 MHz, CDCl$_3$): δ -63.2.
HRMS (ESI): Calcd for $C_{18}H_{23}F_6N_2O_2^+$ [M+H]$^+$:413.1658, found 413.1646

***tert*-butyl 4-((2-bromo-3-methylphenyl)amino)piperidine-1-carboxylate (5)**

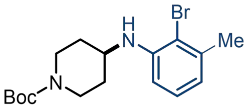

Prepared according to the **GPD** from 2-bromo-3-methylaniline (18.6 mg, 0.1 mmol) and *tert*-butyl 4-iodopiperidine-1-carboxylate (46.7 mg, 0.15 mmol), The crude residue was purified by column chromatography to yield **5** (29.2 mg, 79% yield) as a white solid.

^{1}H NMR (400 MHz, CDCl$_3$): δ 7.06 (dd, *J* = 7.8 Hz, 1H), 6.59 (d, *J* = 7.4 Hz, 1H), 6.51 (d, *J* = 8.1 Hz, 1H), 4.36 (d, *J* = 6.5 Hz, 1H), 4.04 – 3.96 (m, 2H), 3.53 – 3.41 (m, 1H), 2.99 (t, *J* = 11.5 Hz, 2H), 2.36 (s, 3H), 2.03 (d, *J* = 12.4 Hz, 2H), 1.47 (s, 9H), 1.44 – 1.38 (m, 2H).
^{13}C NMR (101 MHz, CDCl$_3$): δ 154.7, 143.7, 138.7, 127.5, 119.0, 112.7, 109.2, 79.6, 50.0, 42.6, 32.0, 28.4, 23.9.
HRMS (ESI): Calcd for $C_{17}H_{26}BrN_2O_2^+$ [M+H]$^+$:369.1172, found 369.1166

***tert*-butyl 4-((4-fluoro-2-methoxyphenyl)amino)piperidine-1-carboxylate (6)**

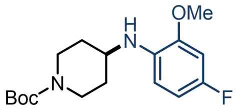

Prepared according to the **GPD** from 4-fluoro-2-methoxyaniline (14.1 mg, 0.1 mmol) and *tert*-butyl 4-iodopiperidine-1-carboxylate (46.7 mg, 0.15 mmol), The crude residue was purified by column chromatography to yield **6** (32.1 mg, 99% yield) as a white solid.

^{1}H NMR (400 MHz, CDCl$_3$): δ 6.60 – 6.47 (m, 3H), 4.10 – 3.96 (m, 2H), 3.81 (s, 3H), 3.41 – 3.32 (m, 1H), 2.92 (t, *J* = 11.7 Hz, 2H), 2.02 (d, *J* = 12.7 Hz, 2H), 1.46 (s, 9H), 1.41 – 1.29 (m, 2H).
^{13}C NMR (101 MHz, CDCl$_3$):: δ 155.3 (d, *J* = 234.7 Hz), 154.8, 147.5 (d, *J* = 9.4 Hz), 132.8 (d, *J* = 2.4 Hz), 110.3 (d, *J* = 8.9 Hz), 106.1 (d, *J* = 21.7 Hz), 98.7 (d, *J* = 27.4 Hz), 79.5, 55.6, 50.3, 42.5, 32.2, 28.4.
^{19}F NMR (377 MHz, CDCl$_3$): δ -126.1.
HRMS (ESI): Calcd for $C_{17}H_{26}FN_2O_3^+$ [M+H]$^+$:325.1922, found 325.1916

***tert*-butyl 4-((2,6-dimethylphenyl)amino)piperidine-1-carboxylate (7)**

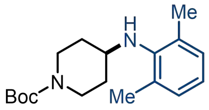

Prepared according to the **GPD** from 2,6-dimethylaniline (12.1 mg, 0.1 mmol) and *tert*-butyl 4-iodopiperidine-1-carboxylate (46.7 mg, 0.15 mmol), The crude residue was purified by column chromatography to yield **7** (15.5 mg, 51% yield) as a colorless oil.

^{1}H NMR (400 MHz, CDCl$_3$): δ 6.99 (d, *J* = 7.4 Hz, 2H), 6.81 (t, *J* = 7.5 Hz, 1H), 4.17 – 3.97 (m, 2H), 3.11 – 3.01 (m, 1H), 2.70 (t, *J* = 12.0 Hz, 2H), 2.26 (s, 6H), 1.90 (d, *J* = 12.7 Hz, 2H), 1.46 (s, 9H), 1.36 – 1.20 (m, 2H).
^{13}C NMR (101 MHz, CDCl$_3$): δ 154.7, 144.3, 129.3, 128.8, 121.7, 79.5, 54.7, 43.1, 33.8, 28.4, 18.9.
HRMS (ESI): Calcd for $C_{18}H_{29}N_2O_2^+$ [M+H]$^+$:305.2224, found 305.2218

***tert*-butyl 4-((3-cyanophenyl)amino)piperidine-1-carboxylate (8)**

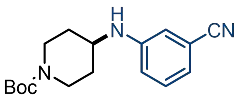

Prepared according to the **GPD** from 3-aminobenzonitrile (11.8 mg, 0.1 mmol) and *tert*-butyl 4-iodopiperidine-1-carboxylate (46.7 mg, 0.15 mmol), The crude residue was purified by column chromatography to yield **8** (19.6 mg, 65% yield) as a white solid.

^{1}H NMR (400 MHz, CDCl$_3$): δ 7.21 (t, *J* = 7.9 Hz, 1H), 6.94 (d, *J* = 7.5 Hz, 1H), 6.81 – 6.74 (m, 2H), 4.16 – 3.98 (m, 2H), 3.85 – 3.75 (m, 1H), 3.46 – 3.35 (m, 1H), 2.93 (t, *J* = 12.5 Hz, 2H), 2.02 (d, *J* = 12.5 Hz, 2H), 1.47 (s, 9H), 1.38 – 1.31 (m, 2H).
^{13}C NMR (101 MHz, CDCl$_3$): δ 154.7, 147.0, 130.0, 120.8, 119.4, 117.6, 115.2, 113.1, 79.8, 49.9, 42.5, 32.0, 28.4.
HRMS (ESI): Calcd for $C_{17}H_{24}N_3O_2^+$ $[M+H]^+$:302.1863, found 302.1858

***tert*-butyl 4-(pyridin-3-ylamino)piperidine-1-carboxylate (9)**

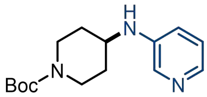

Prepared according to the **GPD** from pyridin-3-amine (9.4 mg, 0.1 mmol) and *tert*-butyl 4-iodopiperidine-1-carboxylate (46.7 mg, 0.15 mmol), The crude residue was purified by column chromatography to yield **9** (17.2 mg, 62% yield) as a white solid.

^{1}H NMR (400 MHz, CDCl$_3$): δ 8.04 – 7.92 (m, 2H), 7.07 (dd, *J* = 8.3, 4.6 Hz, 1H), 6.89 – 6.84 (m, 1H), 4.11 – 3.99 (m, 2H), 3.61 (d, *J* = 7.0 Hz, 1H), 3.48 – 3.36 (m, 1H), 2.92 (t, *J* = 12.1 Hz, 2H), 2.03 (d, *J* = 14.6 Hz, 2H), 1.46 (s, 9H), 1.40 – 1.28 (m, 2H).
^{13}C NMR (101 MHz, CDCl$_3$): δ 154.7, 142.8, 138.8, 136.3, 123.8, 119.1, 79.7, 49.9, 42.6, 32.1, 28.4.
HRMS (ESI): Calcd for $C_{15}H_{24}N_3O_2^+$ $[M+H]^+$:278.1863, found 278.1858

***tert*-butyl 4-(pyridin-2-ylamino)piperidine-1-carboxylate (10)**

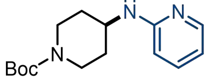

Prepared according to the **GPD** from pyridin-2-amine (9.4 mg, 0.1 mmol) and *tert*-butyl 4-iodopiperidine-1-carboxylate (46.7 mg, 0.15 mmol), The crude residue was purified by column chromatography to yield **10** (27.2 mg, 98% yield) as a white solid. All other spectroscopic analyses were in agreement with the literature[2].

^{1}H NMR (400 MHz, CDCl$_3$): δ 8.05 (d, *J* = 4.3 Hz, 1H), 7.41 – 7.34 (m, 1H), 6.58 – 6.52 (m, 1H), 6.35 (d, *J* = 8.4 Hz, 1H), 4.39 (d, *J* = 8.0 Hz, 1H), 4.11 – 3.95 (m, 2H),

3.85 – 3.71 (m, 1H), 2.94 (t, J = 12.5 Hz, 2H), 2.05 – 2.00 (m, 2H), 1.45 (s, 9H), 1.40 – 1.29 (m, 2H).
^{13}C NMR (101 MHz, $CDCl_3$): δ 157.6, 154.7, 148.1, 137.4, 112.8, 107.4, 79.5, 48.3, 42.5, 32.3, 28.4.
HRMS (ESI): Calcd for $C_{15}H_{24}N_3O_2^+$ [M+H]$^+$:278.1863, found 278.1858

***tert*-butyl 4-(pyrimidin-2-ylamino)piperidine-1-carboxylate (11)**

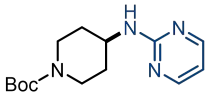

Prepared according to the **GPD** from pyrimidin-2-amine (9.5 mg, 0.1 mmol) and *tert*-butyl 4-iodopiperidine-1-carboxylate (46.7 mg, 0.15 mmol), The crude residue was purified by column chromatography to yield **11** (27.3 mg, 98% yield) as a white solid. All other spectroscopic analyses were in agreement with the literature[2].

^{1}H NMR (400 MHz, $CDCl_3$): δ 8.25 (d, J = 4.8 Hz, 2H), 6.52 (t, J = 4.8 Hz, 1H), 5.22 (d, J = 7.4 Hz, 1H), 4.16 – 3.84 (m, 3H), 2.93 (t, J = 11.8 Hz, 2H), 2.11 – 1.98 (m, 2H), 1.45 (s, 9H), 1.41 – 1.33 (m, 2H).
^{13}C NMR (101 MHz, $CDCl_3$): δ 161.6, 158.0, 154.7, 110.7, 79.6, 48.1, 42.6, 32.1, 28.4.
HRMS (ESI): Calcd for $C_{14}H_{23}N_4O_2^+$ [M+H]$^+$:279.1816, found 279.1817

***tert*-butyl 4-((6-chloropyridin-3-yl)amino)piperidine-1-carboxylate (12)**

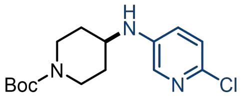

Prepared according to the **GPD** from 6-chloropyridin-3-amine (12.9 mg, 0.1 mmol) and *tert*-butyl 4-iodopiperidine-1-carboxylate (46.7 mg, 0.15 mmol), The crude residue was purified by column chromatography to yield **12** (14.9 mg, 48% yield) as a white solid.

^{1}H NMR (400 MHz, $CDCl_3$): δ 7.75 (d, J = 3.1 Hz, 1H), 7.08 (d, J = 8.6 Hz, 1H), 6.86 (dd, J = 8.6, 3.1 Hz, 1H), 4.12 – 3.99 (m, 2H), 3.62 (d, J = 7.8 Hz, 1H), 3.45 – 3.31 (m, 1H), 2.91 (t, J = 12.6 Hz, 2H), 2.09 – 1.98 (m, 2H), 1.46 (s, 9H), 1.38 – 1.29 (m, 2H).
^{13}C NMR (101 MHz, $CDCl_3$): δ 154.7, 141.9, 139.0, 134.9, 124.1, 122.5, 79.8, 50.1, 42.4, 32.0, 28.4.
HRMS (ESI): Calcd for $C_{15}H_{23}ClN_3O_2^+$ [M+H]$^+$:312.1473, found 312.1465

***tert*-butyl 4-((5-fluoropyridin-2-yl)amino)piperidine-1-carboxylate (13)**

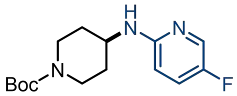

Prepared according to the **GPD** from 5-fluoropyridin-2-amine (11.2 mg, 0.1 mmol) and *tert*-butyl 4-iodopiperidine-1-carboxylate (46.7 mg, 0.15 mmol), The crude residue was purified by column chromatography to yield **13** (28.9 mg, 98% yield) as a white solid.

^{1}H NMR (400 MHz, CDCl$_3$): δ 8.22 – 7.89 (m, 1H), 6.35 – 6.24 (m, 1H), 6.08 – 6.00 (m, 1H), 4.63 (d, *J* = 7.6 Hz, 1H), 4.09 – 3.96 (m, 2H), 3.78 – 3.65 (m, 1H), 3.00 – 2.86 (m, 2H), 2.05 – 1.92 (m, 2H), 1.45 (s, 9H), 1.40 – 1.29 (m, 2H).
^{13}C NMR (101 MHz, CDCl$_3$):: δ 170.0 (d, *J* = 257.1 Hz), 159.9 (d, *J* = 10.5 Hz), 154.7, 150.5 (d, *J* = 9.4 Hz), 101.7 (d, *J* = 18.7 Hz), 93.3 (d, *J* = 20.6 Hz), 79.6, 48.6, 42.5, 32.1, 28.4.
^{19}F NMR (377 MHz, CDCl$_3$): δ -103.4.
HRMS (ESI): Calcd for $C_{15}H_{23}FN_3O_2^+$ [M+H]$^+$:296.1769, found 296.1763

***tert-butyl* 4-(pyrazin-2-ylamino)piperidine-1-carboxylate (14)**

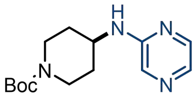

Prepared according to the **GPD** from pyrazin-2-amine (9.5 mg, 0.1 mmol) and *tert*-butyl 4-iodopiperidine-1-carboxylate (46.7 mg, 0.15 mmol), The crude residue was purified by column chromatography to yield **14** (18.1 mg, 65% yield) as a white solid. All other spectroscopic analyses were in agreement with the literature[2].

^{1}H NMR (400 MHz, CDCl$_3$): δ 7.96 (s, 1H), 7.86 (s, 1H), 7.78 (d, *J* = 2.3 Hz, 1H), 4.54 – 4.48 (m, 1H), 4.15 – 4.00 (m, 2H), 3.96 – 3.83 (m, 1H), 2.93 (t, *J* = 11.9 Hz, 2H), 2.04 (d, *J* = 12.1 Hz, 2H), 1.46 (s, 9H), 1.42 – 1.31 (m, 2H).
^{13}C NMR (101 MHz, CDCl$_3$): δ 154.7, 153.7, 141.9, 132.8, 132.6, 79.7, 48.0, 42.4, 32.1, 28.4.
HRMS (ESI): Calcd for $C_{14}H_{23}N_4O_2^+$ [M+H]$^+$:279.1816, found 279.1811

***tert*-butyl 4-(pyrrolo[1,2-b][1,2,4]triazin-2-ylamino)piperidine-1-carboxylate (15)**

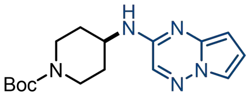

Prepared according to the **GPD** from pyrrolo[1,2-b][1,2,4]triazin-2-amine (13.4 mg, 0.1 mmol) and *tert*-butyl 4-iodopiperidine-1-carboxylate (46.7 mg, 0.15 mmol), The crude residue was purified by column chromatography to yield **15** (30.1 mg, 95% yield) as a colorless oil. All other spectroscopic analyses were in agreement with the literature[2].

^{1}H NMR (400 MHz, CDCl$_3$): δ 7.91 (s, 1H), 7.55 – 7.47 (m, 1H), 6.64 – 6.53 (m, 2H), 5.40 (d, *J* = 7.9 Hz, 1H), 4.47 – 4.28 (m, 1H), 4.20 – 4.06 (m, 2H), 3.07 – 2.85 (m, 2H), 2.09 (d, *J* = 12.1 Hz, 2H), 1.50 – 1.43 (m, 11H).

^{13}C NMR (101 MHz, CDCl$_3$): δ 154.7, 153.4, 147.4, 118.9, 114.6, 110.4, 99.0, 79.8, 47.6, 42.6, 32.1, 28.4.
HRMS (ESI): Calcd for $C_{16}H_{24}N_5O_2^+$ [M+H]$^+$:318.1925, found 318.1918
All other spectroscopic analyses were in agreement with the literature[2].

***tert*-butyl 4-(3-chloro-1H-indazol-1-yl)piperidine-1-carboxylate (16)**

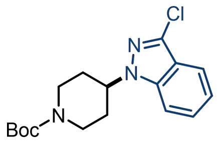

Prepared according to the **GPD** from 3-chloro-1H-indazole (15.3 mg, 0.1 mmol) and *tert*-butyl 4-iodopiperidine-1-carboxylate (46.7 mg, 0.15 mmol), using LiO*t*Bu (14.4 mg, 0.18 mmol) as base. The crude residue was purified by column chromatography to yield **16** (20.8 mg, 62% yield) as a colorless oil. All other spectroscopic analyses were in agreement with the literature[2].

^{1}H NMR (400 MHz, CDCl$_3$): δ 7.68 (d, J = 8.2 Hz, 1H), 7.45 – 7.41 (m, 2H), 7.23 – 7.18 (m, 1H), 4.58 – 4.45 (m, 1H), 4.31 (brs, 2H), 2.93 (brs, 2H), 2.30 – 2.15 (m, 2H), 1.99 (d, J = 11.9 Hz, 2H), 1.48 (s, 9H).
^{13}C NMR (101 MHz, CDCl$_3$): δ 154.5, 140.0, 132.8, 127.3, 121.3, 121.1, 120.0, 109.1, 79.8, 56.5, 42.7, 31.4, 28.4.
HRMS (ESI): Calcd for $C_{17}H_{23}ClN_3O_2^+$ [M+H]$^+$:336.1473, found 336.1469

***tert*-butyl 4-(1H-pyrrol-1-yl)piperidine-1-carboxylate (17)**

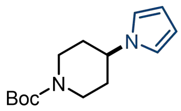

Prepared according to the **GPD** from 1H-pyrrole (6.7 mg, 0.1 mmol) and *tert*-butyl 4-iodopiperidine-1-carboxylate (46.7 mg, 0.15 mmol), The crude residue was purified by column chromatography to yield **17** (18.0 mg, 72% yield) as a colorless oil. All other spectroscopic analyses were in agreement with the literature[2].

^{1}H NMR (400 MHz, CDCl$_3$): δ 6.74 – 6.71 (m, 2H), 6.17 – 6.15 (m, 2H), 4.34 – 4.20 (m, 2H), 4.05 – 3.89 (m, 1H), 2.83 (t, J = 12.6 Hz, 2H), 2.05 (d, J = 12.7 Hz, 2H), 1.91 – 1.74 (m, 2H), 1.48 (s, 9H).
^{13}C NMR (101 MHz, CDCl$_3$): δ 154.6, 118.4, 107.9, 79.9, 56.8, 43.0, 33.5, 28.4.
HRMS (ESI): Calcd for $C_{14}H_{23}N_2O_2^+$ [M+H]$^+$:251.1754, found 251.1749

***tert*-butyl 4-(1H-indol-1-yl)piperidine-1-carboxylate (18)**

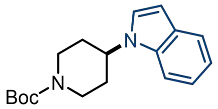

Prepared according to the **GPD** from 1H-indole (11.7 mg, 0.1 mmol) and *tert*-butyl 4-iodopiperidine-1-carboxylate (46.7 mg, 0.15 mmol), The crude residue was purified

by column chromatography to yield **18** (12.0 mg, 40% yield) as a white solid. All other spectroscopic analyses were in agreement with the literature[2].

^{1}H NMR (400 MHz, CDCl$_3$): δ 7.65 (d, *J* = 7.8 Hz, 1H), 7.38 (d, *J* = 8.1 Hz, 1H), 7.24 – 7.20 (m, 1H), 7.19 (d, *J* = 3.3 Hz, 1H), 7.12 (t, *J* = 7.8 Hz, 1H), 6.54 (d, *J* = 3.1 Hz, 1H), 4.43 – 4.25 (m, 3H), 2.91 (t, *J* = 13.8 Hz, 2H), 2.09 (d, *J* = 12.6 Hz, 2H), 1.98 – 1.85 (m, 2H), 1.51 (s, 9H).
^{13}C NMR (101 MHz, CDCl$_3$): δ 154.7, 135.4, 128.5, 123.8, 121.4, 121.1, 119.5, 109.1, 101.7, 79.9, 53.4, 43.5, 32.3, 28.4.
HRMS (ESI): Calcd for $C_{18}H_{25}N_2O_2^+$ [M+H]$^+$:301.1911, found 301.1904

***tert*-butyl 4-(9H-carbazol-9-yl)piperidine-1-carboxylate (19)**

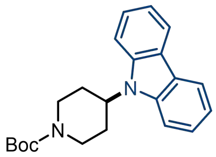

Prepared according to the **GPD** from 9H-carbazole (16.7 mg, 0.1 mmol) and *tert*-butyl 4-iodopiperidine-1-carboxylate (46.7 mg, 0.15 mmol), The crude residue was purified by column chromatography to yield **19** (28.0 mg, 80% yield) as a white solid. All other spectroscopic analyses were in agreement with the literature[2].

^{1}H NMR (400 MHz, CDCl$_3$): δ 8.16 (d, *J* = 7.7 Hz, 2H), 7.56 (d, *J* = 8.3 Hz, 2H), 7.49 (t, *J* = 7.2 Hz, 2H), 7.32 – 7.24 (m, 2H), 4.75 – 4.62 (m, 1H), 4.45 (brs, 2H), 3.00 (t, *J* = 11.6 Hz, 2H), 2.73 – 2.56 (m, 2H), 1.96 (d, *J* = 12.5 Hz, 2H), 1.59 (s, 9H).
^{13}C NMR (101 MHz, CDCl$_3$): δ 154.8, 139.4, 125.5, 123.4, 120.4, 118.9, 109.9, 80.0, 53.5, 43.9, 29.6, 28.4.
HRMS (ESI): Calcd for $C_{22}H_{27}N_2O_2^+$ [M+H]$^+$:351.2067, found 351.2061

***tert*-butyl 4-(2,2,2-trifluoroacetamido)piperidine-1-carboxylate (20)**

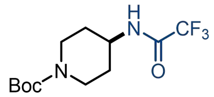

Prepared according to the **GPD** from 2,2,2-trifluoroacetamide (11.3 mg, 0.1 mmol) and *tert*-butyl 4-iodopiperidine-1-carboxylate (46.7 mg, 0.15 mmol), using LiO*t*Bu (14.4 mg, 0.18 mmol) as base. The crude residue was purified by column chromatography to yield **20** (22.8 mg, 77% yield) as a white solid.

^{1}H NMR (400 MHz, CDCl$_3$): δ 6.41 (d, *J* = 6.7 Hz, 1H), 4.18 – 3.88 (m, 3H), 2.94 – 2.77 (m, 2H), 1.95 (d, *J* = 12.0 Hz, 2H), 1.45 (s, 9H), 1.43 – 1.35 (m, 2H).
^{13}C NMR (101 MHz, CDCl$_3$):: δ 156.5 (q, *J* = 37.3 Hz), 154.5, 117.1 (q, *J* = 288.9 Hz), 80.0, 47.5, 42.1, 31.3, 28.3.
HRMS (ESI): Calcd for $C_{12}H_{19}F_3N_2O_3Na^+$ [M+Na]$^+$:319.1240, found 319.1234

***tert*-butyl 4-(4-methylbenzamido)piperidine-1-carboxylate (21)**

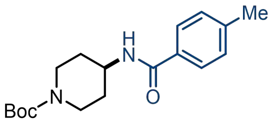

Prepared according to the **GPD** from 4-methylbenzamide (13.5 mg, 0.1 mmol) and *tert*-butyl 4-iodopiperidine-1-carboxylate (46.7 mg, 0.15 mmol), The crude residue was purified by column chromatography to yield **21** (29.6 mg, 93% yield) as a white solid.

^{1}H NMR (400 MHz, CDCl$_3$): δ 7.64 (d, *J* = 8.2 Hz, 2H), 7.21 (d, *J* = 8.1 Hz, 2H), 6.10 (d, *J* = 8.0 Hz, 1H), 4.10 (dt, *J* = 15.2, 4.8 Hz, 3H), 2.88 (t, *J* = 12.7 Hz, 2H), 2.38 (s, 3H), 2.10 – 1.95 (m, 2H), 1.50 – 1.33 (m, 11H).
^{13}C NMR (101 MHz, CDCl$_3$): δ 166.8, 154.7, 141.9, 131.6, 129.2, 126.8, 79.6, 47.1, 42.8, 32.1, 28.4, 21.4.
HRMS (ESI): Calcd for $C_{18}H_{27}N_2O_3^+$ [M+H]$^+$:319.2016, found 319.2012

***tert*-butyl 4-(2-oxopyrrolidin-1-yl)piperidine-1-carboxylate (22)**

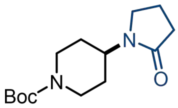

Prepared according to the **GPD** from pyrrolidin-2-one (8.5 mg, 0.1 mmol) and *tert*-butyl 4-iodopiperidine-1-carboxylate (46.7 mg, 0.15 mmol), The crude residue was purified by column chromatography to yield **22** (22.8 mg, 85% yield) as a white solid.

^{1}H NMR (400 MHz, CDCl$_3$): δ 4.28 – 3.99 (m, 3H), 3.31 (t, *J* = 7.0 Hz, 2H), 2.86 – 2.68 (m, 2H), 2.38 (t, *J* = 8.1 Hz, 2H), 2.07 – 1.93 (m, 2H), 1.84 (brs, 1H), 1.67 – 1.59 (m, 1H), 1.58 – 1.50 (m, 2H), 1.44 (s, 9H).
^{13}C NMR (101 MHz, CDCl$_3$): δ 174.5, 154.6, 79.7, 48.8, 43.0, 42.8, 31.4, 29.1, 28.4, 18.1.
HRMS (ESI): Calcd for $C_{14}H_{25}N_2O_3^+$ [M+H]$^+$:269.1860, found 269.1853

***tert*-butyl 4-(3-phenylureido)piperidine-1-carboxylate (23)**

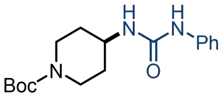

Prepared according to the **GPD** from 1-phenylurea (13.6 mg, 0.1 mmol) and *tert*-butyl 4-iodopiperidine-1-carboxylate (46.7 mg, 0.15 mmol), The crude residue was purified by column chromatography to yield **23** (28.1 mg, 88% yield) as a white solid.

^{1}H NMR (400 MHz, CDCl$_3$): δ 7.52 (brs, 1H), 7.30 – 7.19 (m, 4H), 7.02 – 6.95 (m, 1H), 5.51 (d, *J* = 7.8 Hz, 1H), 3.98 – 3.70 (m, 3H), 2.78 (t, *J* = 11.9 Hz, 2H), 1.84 (d, *J* = 10.7 Hz, 2H), 1.44 (s, 9H), 1.21 – 1.08 (m, 2H).
^{13}C NMR (101 MHz, CDCl$_3$): δ 155.4, 154.8, 139.0, 129.0, 123.0, 119.8, 79.9, 46.8, 42.6, 32.4, 28.4.

HRMS (ESI): Calcd for $C_{17}H_{26}N_3O_3^+$ $[M+H]^+$:320.1969, found 320.1978

***tert*-butyl 4-(phenethylamino)piperidine-1-carboxylate (24)**

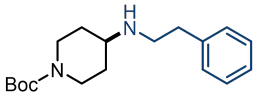

Prepared according to the **GPD** from 2-phenylethan-1-amine (12.1 mg, 0.1 mmol) and *tert*-butyl 4-iodopiperidine-1-carboxylate (46.7 mg, 0.15 mmol), The crude residue was purified by column chromatography to yield **24** (28.3 mg, 93% yield) as a colorless oil. All other spectroscopic analyses were in agreement with the literature[13].

^{1}H NMR (400 MHz, CDCl$_3$): δ 7.32 – 7.27 (m, 2H), 7.23 – 7.17 (m, 3H), 4.12 – 3.91(m, 2H), 2.90 (t, J = 7.0 Hz, 2H), 2.83 – 2.70 (m, 4H), 2.65 – 2.55 (m, 2H), 1.80 (d, J = 12.9 Hz, 2H), 1.43 (s, 9H), 1.28 – 1.19 (m, 2H).
^{13}C NMR (101 MHz, CDCl$_3$): δ 154.8, 139.8, 128.6, 128.5, 126.2, 79.4, 54.9, 47.9, 42.5, 36.4, 32.4, 28.4.
HRMS (ESI): Calcd for $C_{18}H_{29}N_2O_2^+$ $[M+H]^+$:305.2224, found 305.2217

***tert*-butyl 4-((thiophen-2-ylmethyl)amino)piperidine-1-carboxylate (25)**

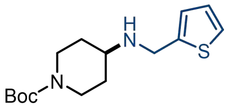

Prepared according to the **GPD** from thiophen-2-ylmethanamine (11.3 mg, 0.1 mmol) and *tert*-butyl 4-iodopiperidine-1-carboxylate (46.7 mg, 0.15 mmol), The crude residue was purified by column chromatography to yield **25** (21.6 mg, 73% yield) as a colorless oil.

^{1}H NMR (400 MHz, CDCl$_3$): δ 7.20 (d, J = 5.0 Hz, 1H), 6.96 – 6.93 (m, 1H), 6.92 – 6.90 (m, 1H), 4.02 (brs, 4H), 2.79 (t, J = 11.7 Hz, 3H), 2.74 – 2.62 (m, 2H), 1.85 (d, J = 12.0 Hz, 2H), 1.45 (s, 9H), 1.34 – 1.21 (m, 2H).
^{13}C NMR (101 MHz, CDCl$_3$): δ 154.8, 144.3, 126.6, 124.7, 124.3, 79.4, 53.7, 45.2, 42.4, 32.3, 28.4.
HRMS (ESI): Calcd for $C_{15}H_{25}N_2O_2S^+$ $[M+H]^+$:297.1631, found 269.1624

***tert*-butyl 4-((3-hydroxyphenyl)amino)piperidine-1-carboxylate (26)**

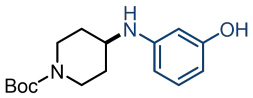

Prepared according to the **GPD** from 3-aminophenol (10.9 mg, 0.1 mmol) and *tert*-butyl 4-iodopiperidine-1-carboxylate (46.7 mg, 0.15 mmol), using MTBD (40.2 µL, 0.28 mmol). The crude residue was purified by column chromatography to yield **26** (26.3 mg, 90% yield) as a white solid.

^{1}H NMR (400 MHz, DMSO-*d*$_6$): δ 8.89 (s, 1H), 6.81 (t, *J* = 7.9 Hz, 1H), 6.05 – 5.91 (m, 3H), 5.33 (d, *J* = 7.8 Hz, 1H), 3.86 (d, *J* = 12.2 Hz, 2H), 3.32 – 3.25 (m, 1H), 2.95 – 2.81 (m, 2H), 1.84 (d, *J* = 10.8 Hz, 2H), 1.39 (s, 9H), 1.26 – 1.14 (m, 2H). **^{13}C NMR (101 MHz, DMSO-*d*$_6$):** δ 158.3, 154.0, 149.0, 129.6, 104.0, 103.2, 99.4, 78.6, 48.7, 42.3, 31.7, 28.1.
HRMS (ESI): Calcd for $C_{16}H_{24}N_2O_3^+$ [M+H]$^+$:293.1860, found 293.1852

***tert*-butyl 4-((2-(hydroxymethyl)phenyl)amino)piperidine-1-carboxylate (27)**

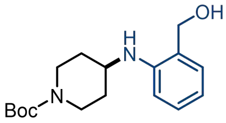

Prepared according to the **GPD** from (2-aminophenyl)methanol (12.3 mg, 0.1 mmol) and *tert*-butyl 4-iodopiperidine-1-carboxylate (46.7 mg, 0.15 mmol), The crude residue was purified by column chromatography to yield **27** (16.8 mg, 55% yield) as a colorless oil.

^{1}H NMR (400 MHz, CDCl$_3$): δ 7.24 – 7.16 (m, 1H), 7.07 – 7.04 (m, 1H), 6.71 – 6.62 (m, 2H), 4.64 (s, 2H), 4.02 – 3.94 (m, 2H), 3.57 – 3.45 (m, 1H), 3.00 (t, *J* = 12.3 Hz, 2H), 2.03 (d, *J* = 10.5 Hz, 2H), 1.50 – 1.38 (m, 11H).
^{13}C NMR (101 MHz, CDCl$_3$): δ 154.8, 146.3, 129.6, 129.4, 124.4, 116.4, 111.1, 79.6, 64.9, 49.3, 42.6, 32.1, 28.4.
HRMS (ESI): Calcd for $C_{17}H_{27}N_2O_3^+$ [M+H]$^+$:307.2016, found 307.2008

***tert*-butyl 4-((2-(2-hydroxyethyl)phenyl)amino)piperidine-1-carboxylate (28)**

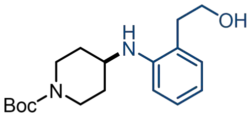

Prepared according to the **GPD** from 2-(2-aminophenyl)ethan-1-ol (13.7 mg, 0.1 mmol) and *tert*-butyl 4-iodopiperidine-1-carboxylate (46.7 mg, 0.15 mmol), The crude residue was purified by column chromatography to yield **28** (17.3 mg, 54% yield) as a colorless oil.

^{1}H NMR (400 MHz, CDCl$_3$): δ 7.13 (t, *J* = 7.7 Hz, 1H), 7.05 (d, *J* = 7.3 Hz, 1H), 6.74 – 6.64 (m, 2H), 4.06 – 3.95 (m, 2H), 3.88 (t, *J* = 6.1 Hz, 2H), 3.52 – 3.39 (m, 1H), 2.98 (t, *J* = 12.0 Hz, 2H), 2.76 (t, *J* = 6.1 Hz, 2H), 2.03 (d, *J* = 12.4 Hz, 2H), 1.47 (s, 9H), 1.44 – 1.32 (m, 2H).
^{13}C NMR (101 MHz, CDCl$_3$): δ 154.8, 145.3, 130.7, 127.8, 124.2, 117.5, 111.6, 79.6, 63.2, 49.8, 42.5, 34.8, 32.3, 28.4.
HRMS (ESI): Calcd for $C_{18}H_{29}N_2O_3^+$ [M+H]$^+$:321.2173, found 321.2165

***tert*-butyl 4-(3-(hydroxymethyl)benzamido)piperidine-1-carboxylate (29)**

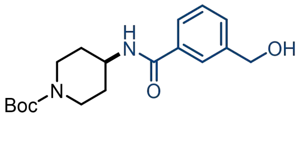

Prepared according to the **GPD** from 3-(hydroxymethyl)benzamide (15.1 mg, 0.1 mmol) and *tert*-butyl 4-iodopiperidine-1-carboxylate (46.7 mg, 0.15 mmol), The crude residue was purified by column chromatography to yield **29** (25.7 mg, 77% yield) as a white solid.

^{1}H NMR (400 MHz, CDCl$_3$): δ 7.72 (s, 1H), 7.64 (d, *J* = 7.6 Hz, 1H), 7.45 (d, *J* = 7.5 Hz, 1H), 7.40 – 7.34 (m, 1H), 6.34 (brs, 1H), 4.69 (s, 2H), 4.13 – 4.00 (m, 3H), 2.93 – 2.76 (m, 2H), 2.03 – 1.87 (m, 3H), 1.44 (s, 9H), 1.40 – 1.29 (m, 2H).
^{13}C NMR (101 MHz, CDCl$_3$): δ 167.0, 154.8, 141.6, 134.7, 129.9, 128.7, 126.0, 125.2, 79.8, 64.5, 47.2, 42.4, 32.0, 28.4.
HRMS (ESI): Calcd for $C_{18}H_{27}N_2O_4^+$ [M+H]$^+$:335.1965, found 335.1965

***tert*-butyl 4-((4-(hydroxymethyl)phenyl)amino)piperidine-1-carboxylate (30)**

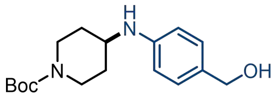

Prepared according to the **GPD** from (4-aminophenyl)methanol (12.3 mg, 0.1 mmol) and *tert*-butyl 4-iodopiperidine-1-carboxylate (46.7 mg, 0.15 mmol), The crude residue was purified by column chromatography to yield **30** (19.6 mg, 64% yield) as a white solid.

^{1}H NMR (400 MHz, CDCl$_3$): δ 7.18 (d, *J* = 8.1 Hz, 2H), 6.59 (d, *J* = 8.2 Hz, 2H), 4.55 (s, 2H), 4.04 (d, *J* = 10.0 Hz, 2H), 3.47 – 3.38 (m, 1H), 2.92 (t, *J* = 12.4 Hz, 2H), 2.03 (d, *J* = 10.8 Hz, 2H), 1.46 (s, 9H), 1.38 – 1.29 (m, 2H).
^{13}C NMR (101 MHz, CDCl$_3$): δ 154.8, 146.5, 129.9, 129.0, 113.3, 79.7, 65.4, 50.2, 42.7, 32.4, 28.5.
HRMS (ESI): Calcd for Calcd for $C_{17}H_{27}N_2O_3^+$ [M+H]$^+$:307.2016, found 307.2012

***tert*-butyl 4-((4-(2-hydroxyethyl)phenyl)amino)piperidine-1-carboxylate (31)**

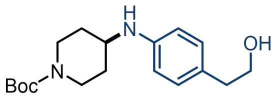

Prepared according to the **GPD** from 2-(4-aminophenyl)ethan-1-ol (13.7 mg, 0.1 mmol) and *tert*-butyl 4-iodopiperidine-1-carboxylate (46.7 mg, 0.15 mmol), The crude residue was purified by column chromatography to yield **30** (26.6 mg, 83% yield) as a white solid.

^{1}H NMR (400 MHz, CDCl$_3$): δ 7.03 (d, *J* = 8.1 Hz, 2H), 6.56 (d, *J* = 8.1 Hz, 2H), 4.07 – 3.97 (m, 2H), 3.79 (t, *J* = 6.5 Hz, 2H), 3.46 – 3.35 (m, 1H), 2.92 (t, *J* = 12.5 Hz, 2H), 2.75 (t, *J* = 6.5 Hz, 2H), 2.02 (d, *J* = 14.9 Hz, 2H), 1.46 (s, 9H), 1.36 – 1.29 (m, 2H).
^{13}C NMR (101 MHz, CDCl$_3$): δ 154.8, 145.4, 129.9, 127.1, 113.6, 79.6, 63.9, 50.3, 42.6, 38.2, 32.4, 28.4.
HRMS (ESI): Calcd for $C_{18}H_{29}N_2O_3^+$ [M+H]$^+$:321.2173, found 321.2168

***tert*-butyl 4-((5-(hydroxymethyl)pyridin-2-yl)amino)piperidine-1-carboxylate (32)**

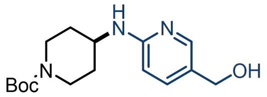

Prepared according to the **GPD** from (6-aminopyridin-3-yl)methanol (12.4 mg, 0.1 mmol) and *tert*-butyl 4-iodopiperidine-1-carboxylate (46.7 mg, 0.15 mmol), The crude residue was purified by column chromatography to yield **32** (23.0 mg, 75% yield) as a white solid.

^{1}H NMR (400 MHz, CDCl$_3$): δ 7.96 (d, *J* = 5.3 Hz, 1H), 6.50 (d, *J* = 5.2 Hz, 1H), 6.40 (s, 1H), 4.59 (s, 2H), 4.46 (d, *J* = 8.0 Hz, 1H), 3.99 (d, *J* = 13.2 Hz, 2H), 3.79 – 3.68 (m, 1H), 3.03 – 2.84 (m, 2H), 2.06 – 1.94 (m, 2H), 1.45 (s, 9H), 1.37 – 1.26 (m, 2H).
^{13}C NMR (101 MHz, CDCl$_3$): δ 158.0, 154.8, 151.8, 147.9, 110.7, 104.2, 79.7, 63.6, 48.3, 42.6, 32.3, 28.4.
HRMS (ESI): Calcd for $C_{16}H_{26}N_3O_3^+$ $[M+H]^+$:308.1969, found 308.1962

***tert*-butyl 4-(4-hydroxy-2-oxopyrrolidin-1-yl)piperidine-1-carboxylate (33)**

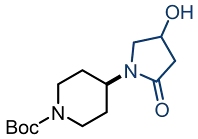

Prepared according to the **GPD** from 4-hydroxypyrrolidin-2-one (10.1 mg, 0.1 mmol) and *tert*-butyl 4-iodopiperidine-1-carboxylate (46.7 mg, 0.15 mmol), The crude residue was purified by column chromatography to yield **33** (21.0 mg, 74% yield) as a colorless oil.

^{1}H NMR (400 MHz, CDCl$_3$): δ 4.48 (s, 1H), 4.26 – 4.04 (m, 3H), 3.58 – 3.48 (m, 1H), 3.24 (d, *J* = 10.5 Hz, 1H), 2.81 – 2.60 (m, 3H), 2.40 – 2.34 (m, 1H), 1.71 – 1.49 (m, 4H), 1.43 (s, 9H), 1.28 – 1.16 (m, 1H).
^{13}C NMR (101 MHz, CDCl$_3$): δ 172.5, 154.6, 79.8, 64.3, 52.0, 48.6, 42.7, 41.5, 29.0, 28.3.
HRMS (ESI): Calcd for $C_{14}H_{25}N_2O_4^+$ $[M+H]^+$:285.1809, found 285.1801

2-(4-(cyclohexylamino)phenyl)ethan-1-ol (34)

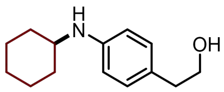

Prepared according to the **GPD** from 2-(4-aminophenyl)ethan-1-ol (13.7 mg, 0.1 mmol) and iodocyclohexane (31.5 mg, 0.15 mmol), The crude residue was purified by column chromatography to yield **34** (11.6 mg, 53% yield) as a colorless oil.

^{1}H NMR (400 MHz, CDCl$_3$): δ 7.01 (d, *J* = 8.4 Hz, 2H), 6.55 (d, *J* = 8.4 Hz, 2H), 4.72 (s, 1H), 3.79 (t, *J* = 6.5 Hz, 2H), 3.30 – 3.16 (m, 1H), 2.74 (t, *J* = 6.5 Hz, 2H), 2.07 – 2.00 (m, 2H), 1.81 – 1.71 (m, 2H), 1.70 – 1.59 (m, 1H), 1.40 – 1.31 (m, 2H), 1.27 – 1.20 (m, 1H), 1.19 – 1.08 (m, 2H).
^{13}C NMR (101 MHz, CDCl$_3$): δ 146.1, 129.8, 126.3, 113.4, 63.9, 51.9, 38.2, 33.5, 25.9, 25.0.
HRMS (ESI): Calcd for $C_{14}H_{22}NO^+$ [M+H]$^+$:220.1696, found 220.1691

2-(4-(cycloheptylamino)phenyl)ethan-1-ol (35)

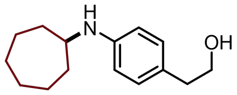

Prepared according to the **GPD** from 2-(4-aminophenyl)ethan-1-ol (13.7 mg, 0.1 mmol) and iodocycloheptane (33.6 mg, 0.15 mmol), The crude residue was purified by column chromatography to yield **35** (20.3 mg, 87% yield) as a white solid.

^{1}H NMR (400 MHz, CDCl$_3$): δ 7.02 (d, *J* = 8.2 Hz, 2H), 6.51 (d, *J* = 8.4 Hz, 2H), 3.78 (t, *J* = 6.5 Hz, 2H), 3.47 – 3.38 (m, 1H), 2.74 (t, *J* = 6.5 Hz, 2H), 2.03 – 1.96 (m, 2H), 1.72 – 1.60 (m, 4H), 1.57 – 1.40 (m, 6H).
^{13}C NMR (101 MHz, CDCl$_3$): δ 146.0, 129.8, 126.2, 113.5, 63.9, 53.8, 38.2, 34.8, 28.3, 24.4.
HRMS (ESI): Calcd for $C_{15}H_{24}NO^+$ [M+H]$^+$:234.1842, found 234.1847

2-(4-((1,4-dioxaspiro[4.5]decan-8-yl)amino)phenyl)ethan-1-ol (36)

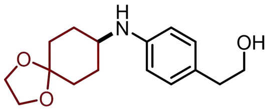

Prepared according to the **GPD** from 2-(4-aminophenyl)ethan-1-ol (13.7 mg, 0.1 mmol) and 8-iodo-1,4-dioxaspiro[4.5]decane (40.2 mg, 0.15 mmol), The crude residue was purified by column chromatography to yield **36** (20.5 mg, 74% yield) as a white solid.

^{1}H NMR (400 MHz, CDCl$_3$): δ 7.02 (d, *J* = 8.4 Hz, 2H), 6.56 (d, *J* = 8.4 Hz, 2H), 3.95 (s, 4H), 3.79 (t, *J* = 6.5 Hz, 2H), 3.39 – 3.29 (m, 1H), 2.75 (t, *J* = 6.5 Hz, 2H), 2.10 – 2.00 (m, 2H), 1.84 – 1.74 (m6, 2H), 1.70 – 1.61 (m, 2H), 1.56 – 1.48 (m, 2H).
^{13}C NMR (101 MHz, CDCl$_3$): δ 145.9, 129.9, 126.6, 113.5, 108.3, 64.4, 64.3, 63.9, 50.4, 38.2, 33.0, 30.0.
HRMS (ESI): Calcd for $C_{16}H_{24}NO_3^+$ [M+H]$^+$:278.1751, found 278.1745

***N*-(4,4-difluorocyclohexyl)-4-methylaniline (37)**

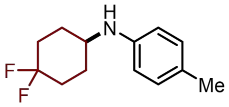

Prepared according to the **GPD** from *p*-toluidine (10.7 mg, 0.1 mmol) and 1,1-difluoro-4-iodocyclohexane (36.9 mg, 0.15 mmol), The crude residue was purified by column chromatography to yield **37** (20.0 mg, 89% yield) as a white solid.

^{1}H NMR (400 MHz, CDCl$_3$): δ 7.00 (d, *J* = 8.0 Hz, 2H), 6.54 (d, *J* = 8.4 Hz, 2H), 3.46 – 3.27 (m, 2H), 2.24 (s, 3H), 2.18 – 2.05 (m, 4H), 1.97 – 1.78 (m, 2H), 1.62 – 1.47 (m, 2H).
^{13}C NMR (101 MHz, CDCl$_3$): δ 144.5, 129.9, 127.0, 122.9 (t, *J* = 242.4 Hz), 113.6, 49.9, 32.0 (t, *J* = 24.7 Hz), 28.8 (d, *J* = 8.0 Hz), 20.3. **^{19}F NMR (377 MHz, CDCl$_3$):** δ -95.5 (d, *J* = 236.6 Hz), -99.9 (d, *J* = 236.2 Hz).
HRMS (ESI): Calcd for $C_{13}H_{18}F_2N^+$ $[M+H]^+$:226.1402, found 226.1396

***N*-(*p*-tolyl)-1-tosylpyrrolidin-3-amine (38)**

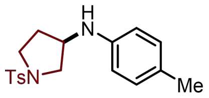

Prepared according to the **GPD** from *p*-toluidine (10.7 mg, 0.1 mmol) and 3-iodo-1-tosylpyrrolidine (52.7 mg, 0.15 mmol), The crude residue was purified by column chromatography to yield **38** (13.9 mg, 42% yield) as a white solid.

^{1}H NMR (400 MHz, CDCl$_3$): δ 7.71 (d, *J* = 8.2 Hz, 2H), 7.32 (d, *J* = 8.0 Hz, 2H), 6.96 (d, *J* = 8.1 Hz, 2H), 6.34 (d, *J* = 8.3 Hz, 2H), 3.95 – 3.89 (m, 1H), 3.53 – 3.47 (m, 1H), 3.40 – 3.24 (m, 3H), 3.21 – 3.14 (m, 1H), 2.45 (s, 3H), 2.23 (s, 3H), 2.16 – 2.04 (m, 1H), 1.88 – 1.75 (m, 1H).
^{13}C NMR (101 MHz, CDCl$_3$): δ 143.8, 143.6, 133.1, 129.7, 129.7, 127.6, 127.3, 113.4, 53.8, 52.8, 46.1, 31.5, 21.5, 20.3.
HRMS (ESI): Calcd for $C_{18}H_{23}N_2O_2S^+$ $[M+H]^+$:331.1475, found 331.1469

2-(4-(oxetan-3-ylamino)phenyl)ethan-1-ol (39)

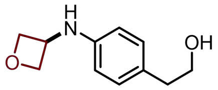

Prepared according to the **GPD** from 2-(4-aminophenyl)ethan-1-ol (13.7 mg, 0.1 mmol) and 3-iodooxetane (27.6 mg, 0.15 mmol), The crude residue was purified by column chromatography to yield **39** (11.8 mg, 61% yield) as a white solid.

^{1}H NMR (400 MHz, CDCl$_3$): δ 7.05 (d, *J* = 8.2 Hz, 2H), 6.47 (d, *J* = 8.3 Hz, 2H), 4.99 (t, *J* = 6.6 Hz, 2H), 4.61 (brs, 1H), 4.51 (t, *J* = 6.1 Hz, 2H), 4.08 (brs, 1H), 3.80 (t, *J* = 6.5 Hz, 2H), 2.76 (t, *J* = 6.5 Hz, 2H).
^{13}C NMR (101 MHz, CDCl$_3$): δ 144.7, 130.0, 128.3, 113.4, 79.2, 63.8, 48.7, 38.2.
HRMS (ESI): Calcd for $C_{11}H_{16}NO_2^+$ $[M+H]^+$:194.1176, found 194.1172

***N*-(*p*-tolyl)tetrahydrofuran-3-amine (40)**

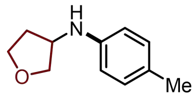

Prepared according to the **GPD** from *p*-toluidine (10.7 mg, 0.1 mmol) and 3-iodooxetane (29.7 mg, 0.15 mmol), The crude residue was purified by column chromatography to yield **40** (12.6 mg, 71% yield) as a white solid.

^{1}H NMR (400 MHz, CDCl$_3$): δ 7.00 (d, *J* = 8.2 Hz, 2H), 6.53 (d, *J* = 8.4 Hz, 2H), 4.11 – 4.04 (m, 1H), 4.00 – 3.91 (m, 2H), 3.87 – 3.80 (m, 1H), 3.72 – 3.64 (m, 2H), 2.32 – 2.18 (m, 4H), 1.92 – 1.80 (m, 1H).
^{13}C NMR (101 MHz, CDCl$_3$): δ 144.8, 129.8, 127.1, 113.6, 73.8, 67.1, 54.1, 33.2, 20.3.
HRMS (ESI): Calcd for $C_{11}H_{16}NO^+$ $[M+H]^+$:178.1226, found 178.1223

2-(4-((tetrahydro-2H-pyran-4-yl)amino)phenyl)ethan-1-ol (41)

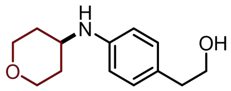

Prepared according to the **GPD** from 2-(4-aminophenyl)ethan-1-ol (13.7 mg, 0.1 mmol) and 4-iodotetrahydro-2H-pyran (31.8 mg, 0.15 mmol), The crude residue was purified by column chromatography to yield **41** (16.6 mg, 75% yield) as a white solid.

^{1}H NMR (400 MHz, CDCl$_3$): δ 7.03 (d, *J* = 8.4 Hz, 2H), 6.58 (d, *J* = 8.4 Hz, 2H), 4.06 – 3.95 (m, 2H), 3.79 (t, *J* = 6.5 Hz, 2H), 3.56 – 3.39 (m, 3H), 2.75 (t, *J* = 6.6 Hz, 2H), 2.03 (d, *J* = 13.5 Hz, 2H), 1.54 – 1.37 (m, 2H).
^{13}C NMR (101 MHz, CDCl$_3$): δ 145.3, 129.9, 127.1, 113.7, 66.8, 63.9, 49.2, 38.2, 33.6.
HRMS (ESI): Calcd for $C_{13}H_{20}NO_2^+$ $[M+H]^+$:222.1489, found 222.1484

2-(4-((tetrahydro-2H-thiopyran-4-yl)amino)phenyl)ethan-1-ol (42)

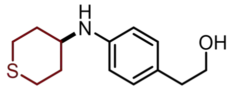

Prepared according to the **GPD** from 2-(4-aminophenyl)ethan-1-ol (13.7 mg, 0.1 mmol) and 4-iodotetrahydro-2H-thiopyran (34.2 mg, 0.15 mmol), The crude residue was purified by column chromatography to yield **42** (13.3 mg, 56% yield) as a white solid.
^{1}H NMR (400 MHz, CDCl$_3$): δ 7.02 (d, *J* = 8.2 Hz, 2H), 6.54 (d, *J* = 8.3 Hz, 2H), 3.79 (t, *J* = 6.5 Hz, 2H), 3.47 (brs, 1H), 3.31 – 3.20 (m, 1H), 2.83 – 2.67 (m, 6H), 2.39 – 2.29 (m, 2H), 1.64 – 1.50 (m, 2H).
^{13}C NMR (101 MHz, CDCl$_3$): δ 145.2, 129.9, 127.0, 113.6, 63.9, 51.2, 38.2, 34.5, 27.8.
HRMS (ESI): Calcd for $C_{13}H_{20}NOS^+$ $[M+H]^+$:238.1260, found 238.1255

***tert*-butyl**
6-((4-(2-hydroxyethyl)phenyl)amino)-2-azaspiro[3.3]heptane-2-carboxylate (43)

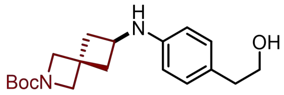

Prepared according to the **GPD** from 2-(4-aminophenyl)ethan-1-ol (13.7 mg, 0.1 mmol) and *tert*-butyl 6-iodo-2-azaspiro[3.3]heptane-2-carboxylate (48.5 mg, 0.15 mmol), The crude residue was purified by column chromatography to yield **43** (16.6 mg, 50% yield) as a white solid.

^{1}H NMR (400 MHz, CDCl$_3$): δ 7.03 (d, *J* = 8.1 Hz, 2H), 6.48 (d, *J* = 8.1 Hz, 2H), 3.98 (s, 2H), 3.87 (s, 2H), 3.80 – 3.75 (m, 3H), 2.75 (t, *J* = 6.6 Hz, 2H), 2.70 – 2.62 (m, 2H), 2.03 – 1.96 (m, 2H), 1.74 – 1.58 (m, 2H), 1.43 (s, 9H).
^{13}C NMR (101 MHz, CDCl$_3$): δ 156.2, 145.5, 129.9, 127.4, 113.3, 79.4, 63.9, 61.3, 60.5, 43.9, 41.8, 38.2, 32.1, 28.4.
HRMS (ESI): Calcd for $C_{19}H_{29}N_2O_3^+$ $[M+H]^+$:333.2173, found 333.2163

***tert*-butyl 2-(p-tolylamino)-7-azaspiro[3.5]nonane-7-carboxylate (44)**

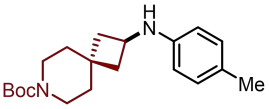

Prepared according to the **GPD** from *p*-toluidine (10.7 mg, 0.1 mmol) and *tert*-butyl 2-iodo-7-azaspiro[3.5]nonane-7-carboxylate (52.7 mg, 0.15 mmol), The crude residue was purified by column chromatography to yield **44** (20.5 mg, 62% yield) as a white solid.

^{1}H NMR (400 MHz, CDCl$_3$): δ 6.98 (d, *J* = 8.0 Hz, 2H), 6.46 (d, *J* = 8.2 Hz, 2H), 3.94 – 3.84 (m, 1H), 3.64 (brs, 1H), 3.42 – 3.35 (m, 2H), 3.34 – 3.25 (m, 3H), 2.41 – 2.32 (m, 2H), 2.23 (s, 3H), δ 1.64 – 1.58 (m, 3H), 1.55 – 1.50 (m, 3H), 1.45 (s, 9H).
^{13}C NMR (101 MHz, CDCl$_3$): δ 144.9, 129.7, 126.8, 113.2, 79.3, 43.9, 40.7, 40.5, 36.6, 32.5, 28.4, 20.4.
HRMS (ESI): Calcd for $C_{20}H_{31}N_2O_2^+$ $[M+H]^+$:331.2380, found 331.2372

methyl 3-(p-tolylamino)cyclobutane-1-carboxylate (45)

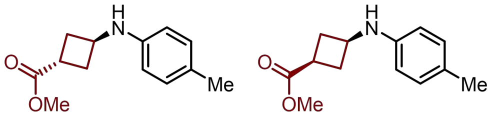

Prepared according to the **GPD** from *p*-toluidine (10.7 mg, 0.1 mmol) and methyl 3-iodocyclobutane-1-carboxylate (36.0 mg, 0.15 mmol), The crude residue was purified by column chromatography to yield **45** (18.4 mg, 84% yield) as an unseparable mixture of diastereomers. dr = 1.8:1

^{1}H NMR (400 MHz, CDCl$_3$, diastereomers): δ 6.99 (d, *J* = 8.0 Hz, 2H), 6.52 – 6.46 (m, 2H), 4.21 – 4.07 (m, 0.68H), 3.96 – 3.83 (m, 0.43H), 3.73 (s, 1.87H), 3.69 (s,

1.09H), 3.21 – 3.09 (m, 0.77H), 2.94 – 2.82 (m, 0.60H), 2.77 – 2.66 (m, 2.15H), 2.24 (s, 3.07H), 2.19 – 2.05 (m, 2.14H).
^{13}C NMR (101 MHz, $CDCl_3$, diastereomers): δ 176.3, 175.3, 144.5, 144.3, 129.8, 127.1, 113.3, 113.3, 51.9, 51.8, 47.0, 45.2, 34.7, 33.5, 33.0, 31.4, 20.3.
HRMS (ESI): Calcd for $C_{13}H_{18}NO_2^+$ $[M+H]^+$:220.1332, found 220.1327

***N*-(heptan-3-yl)-4-methylaniline (46)**

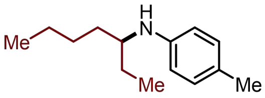

Prepared according to the **GPD** from *p*-toluidine (10.7 mg, 0.1 mmol) and 3-iodoheptane (33.9 mg, 0.15 mmol), The crude residue was purified by column chromatography to yield **46** (15.4 mg, 75% yield) as a colorless oil. All other spectroscopic analyses were in agreement with the literature[14].

^{1}H NMR (400 MHz, $CDCl_3$): δ 6.97 (d, J = 8.1 Hz, 2H), 6.50 (d, J = 8.4 Hz, 2H), 3.33 – 3.21 (m, 2H), 2.23 (s, 3H), 1.64 – 1.54 (m, 2H), 1.51 – 1.43 (m, 2H), 1.38 – 1.29 (m, 4H), 0.95 – 0.88 (m, 6H).
^{13}C NMR (101 MHz, $CDCl_3$): δ 146.0, 129.7, 125.6, 113.1, 54.3, 34.1, 28.2, 27.2, 22.8, 20.3, 14.1, 10.0.
HRMS (ESI): Calcd for $C_{14}H_{24}N^+$ $[M+H]^+$:206.1903, found 206.1898

2-(4-((1-phenoxypropan-2-yl)amino)phenyl)ethan-1-ol (47)

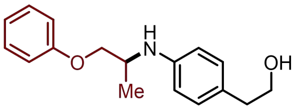

Prepared according to the **GPD** from 2-(4-aminophenyl)ethan-1-ol (13.7 mg, 0.1 mmol) and (2-iodopropoxy)benzene (39.3 mg, 0.15 mmol), The crude residue was purified by column chromatography to yield **47** (17.1 mg, 63% yield) as a colorless oil.

^{1}H NMR (400 MHz, $CDCl_3$): δ 7.31 – 7.25 (m, 2H), 7.04 (d, J = 8.2 Hz, 2H), 6.98 – 6.93 (m, 1H), 6.91 (d, J = 8.0 Hz, 2H), 6.62 (d, J = 8.2 Hz, 2H), 4.04 – 4.00 (m, 1H), 3.95 – 3.84 (m, 2H), 3.80 (t, J = 6.5 Hz, 2H), 2.76 (t, J = 6.5 Hz, 2H), 1.36 (d, J = 6.2 Hz, 3H).
^{13}C NMR (101 MHz, $CDCl_3$): δ 158.8, 145.7, 129.9, 129.5, 127.1, 120.9, 114.6, 113.7, 70.8, 63.9, 48.3, 38.2, 18.1.
HRMS (ESI): Calcd for $C_{17}H_{22}NO_2^+$ $[M+H]^+$:272.1645, found 272.1638

2-(4-((1-(tetrahydro-2H-pyran-4-yl)ethyl)amino)phenyl)ethan-1-ol (48)

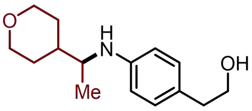

Prepared according to the **GPD** from 2-(4-aminophenyl)ethan-1-ol (13.7 mg, 0.1 mmol) and (4-(1-iodoethyl)tetrahydro-2H-pyran (36.0 mg, 0.15 mmol), The crude residue was purified by column chromatography to yield **48** (13.2 mg, 53% yield) as a colorless oil.

^{1}H NMR (400 MHz, CDCl$_3$): δ 7.02 (d, *J* = 8.2 Hz, 2H), 6.53 (d, *J* = 8.4 Hz, 2H), 4.81 – 4.66 (m, 1H), 4.01 (dd, *J* = 11.3, 3.9 Hz, 2H), 3.79 (t, *J* = 6.5 Hz, 2H), 3.44 – 3.24 (m, 3H), 2.75 (t, *J* = 6.5 Hz, 2H), 1.78 – 1.71 (m, 1H), 1.69 – 1.59 (m, 2H), 1.51 – 1.39 (m, 2H), 1.14 (d, *J* = 6.4 Hz, 3H).
^{13}C NMR (101 MHz, CDCl$_3$): δ 146.3, 129.9, 126.4, 113.3, 68.2, 68.0, 63.9, 52.8, 40.6, 38.2, 29.8, 28.7, 17.5.
HRMS (ESI): Calcd for $C_{15}H_{24}NO_2^+$ $[M+H]^+$:250.1802, found 250.1795

6-(*p*-tolylamino)hexahydro-2H-3,5-methanocyclopenta[b]furan-2-one (49)

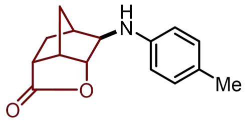

Prepared according to the **GPD** from *p*-toluidine (10.7 mg, 0.1 mmol) and 6-iodohexahydro-2H-3,5-methanocyclopenta[b]furan-2-one (39.6 mg, 0.15 mmol), The crude residue was purified by column chromatography to yield **49** (16.1 mg, 66% yield) as a white solid.

^{1}H NMR (400 MHz, CDCl$_3$): δ 7.02 (d, *J* = 8.1 Hz, 2H), 6.55 (d, *J* = 8.4 Hz, 2H), 4.44 (d, *J* = 5.1 Hz, 1H), 3.45 (brs, 1H), 3.26 (s, 1H), 3.20 (t, *J* = 4.4 Hz, 1H), 2.61 (dd, *J* = 11.2, 4.5 Hz, 1H), 2.44 (s, 1H), 2.25 (s, 3H), 2.15 – 2.05 (m, 2H), 1.84 (d, *J* = 13.4 Hz, 1H), 1.67 (d, *J* = 10.6 Hz, 1H).
^{13}C NMR (101 MHz, CDCl$_3$): δ 180.3, 143.3, 130.0, 127.6, 113.3, 84.7, 62.3, 45.6, 42.3, 38.6, 35.0, 33.6, 20.3.
HRMS (ESI): Calcd for $C_{15}H_{18}NO_2^+$ $[M+H]^+$:244.1332, found 244.1326

***tert*-butyl 3-(*p*-tolylamino)-8-azabicyclo[3.2.1]octane-8-carboxylate (50)**

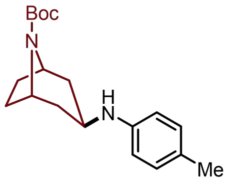

Prepared according to the **GPD** from *p*-toluidine (10.7 mg, 0.1 mmol) and *tert*-butyl 3-exo-iodo-8-azabicyclo[3.2.1]octane-8-carboxylate (50.6 mg, 0.15 mmol), The crude residue was purified by column chromatography to yield **50** (21.2 mg, 67% yield) as a white solid.

^{1}H NMR (400 MHz, CDCl$_3$): δ 6.97 (d, *J* = 8.1 Hz, 2H), 6.53 (d, *J* = 8.2 Hz, 2H), 4.26 (d, *J* = 26.1 Hz, 2H), 3.83 – 3.71 (m, 1H), 3.16 (brs, 1H), 2.23 (s, 3H), 2.09 – 1.98 (m, 4H), 1.74 (q, *J* = 6.7 Hz, 2H), 1.49 – 1.42 (m, 11H).

^{13}C NMR (101 MHz, CDCl$_3$): δ 153.3, 144.7, 129.8, 126.8, 113.6, 79.3, 53.5, 52.8, 45.1, 38.9, 38.3, 28.5, 28.4, 20.3.
HRMS (ESI): Calcd for $C_{19}H_{29}N_2O_2^+$ $[M+H]^+$:317.2224, found 317.2215

***N*-((1R,2S,5R)-2-isopropyl-5-methylcyclohexyl)-4-methylaniline (51)**

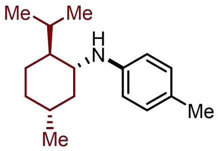

Prepared according to the **GPD** from *p*-toluidine (10.7 mg, 0.1 mmol) and (1S,4R)-2-iodo-1-isopropyl-4-methylcyclohexane (39.9 mg, 0.15 mmol), The crude residue was purified by column chromatography to yield **51** (12.3 mg, 50% yield) as a colorless oil.

^{1}H NMR (400 MHz, CDCl$_3$): δ 6.96 (d, J = 8.1 Hz, 2H), 6.50 (d, J = 8.1 Hz, 2H), 4.73 (s, 1H), 3.17 – 3.06 (m, 1H), 2.25 – 2.12 (m, 5H), 1.77 – 1.68 (m, 2H), 1.48 – 1.38 (m, 1H), 1.16 – 1.06 (m, 2H), 0.94 – 0.85 (m, 8H), 0.76 (d, J = 6.9 Hz, 3H).
^{13}C NMR (101 MHz, CDCl$_3$): δ 145.8, 129.8, 125.6, 113.0, 53.8, 48.8, 42.9, 34.9, 32.0, 26.3, 24.2, 22.2, 21.2, 20.3, 16.2.
HRMS (ESI): Calcd for $C_{17}H_{28}N^+$ $[M+H]^+$:246.2216, found 246.2209

***N*-(2-fluorocyclohexyl)-4-methylaniline (52)**

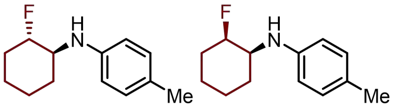

Prepared according to the **GPD** from *p*-toluidine (10.7 mg, 0.1 mmol) and 1-fluoro-2-iodocyclohexane (34.2 mg, 0.15 mmol), The crude residue was purified by column chromatography to yield **52** (12.3 mg, 50% yield) as an inseparable mixture of.diastereomers. dr = 4.1:1

^{1}H NMR (400 MHz, CDCl$_3$, diastereomers): δ 6.99 (d, J = 8.2 Hz, 2H), 6.61 (d, J = 8.2 Hz, 1.60H), 6.55 (d, J = 8.2 Hz, 0.37H), 4.47 – 4.26 (m, 0.97H), 3.63 (brs, 0.85H), 3.44 – 3.23 (m, 1.10H), 2.29 – 2.05 (m, 5.42H), 1.84 – 1.76 (m, 1.19H), 1.69 – 1.16 (m, 7.49H).
^{13}C NMR (101 MHz, CDCl$_3$, diastereomers): δ 145.2, 144.4, 129.8, 129.7, 127.0, 126.9, 114.0, 113.9, 95.5, 93.7, 91.3, 89.6, 56.8, 56.6, 54.3, 54.1, 30.9, 30.8, 30.8, 30.8, 30.5, 30.3, 27.2, 27.1, 24.4, 23.5, 23.2, 23.1, 20.3, 19.6.
^{19}F NMR (377 MHz, CDCl$_3$, diastereomers): δ -169.2, -178.2.
HRMS (ESI): Calcd for $C_{13}H_{19}FN^+$ $[M+H]^+$:208.1496, found 208.1490

***trans*-O-(2-ethoxycyclohexyl)-4-methylaniline (53)**

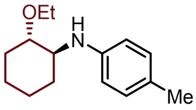

Prepared according to the **GPD** from *p*-toluidine (10.7 mg, 0.1 mmol) and (1S,2S)-1-ethoxy-2-iodocyclohexane (38.1 mg, 0.15 mmol), The crude residue was purified by column chromatography to yield **53** (10.5 mg, 45% yield) as a mixture of.diastereomers. dr = 4.5:1

^{1}H NMR (400 MHz, $CDCl_3$, major diastereomer): δ 6.97 (d, *J* = 8.2 Hz, 2H), 6.62 (d, *J* = 8.3 Hz, 2H), 3.80 (brs, 1H), 3.69 – 3.58 (m, 1H), 3.52 – 3.42 (m, 1H), 3.25 – 3.11 (m, 2H), 2.23 (s, 3H), 2.22 – 2.17 (m, 1H), 2.08 – 2.00 (m, 1H), 1.76 (dd, *J* = 10.2, 3.5 Hz, 1H), 1.64 (dd, *J* = 10.9, 5.3 Hz, 1H), 1.42 – 1.25 (m, 4H), 1.16 (t, *J* = 7.0 Hz, 3H).
^{13}C NMR (101 MHz, $CDCl_3$, major diastereomer): δ 145.9, 129.5, 126.7, 114.3, 80.8, 63.9, 57.2, 31.4, 30.0, 23.9, 23.8, 20.4, 15.7.
HRMS (ESI): Calcd for $C_{15}H_{24}NO^+$ $[M+H]^+$:234.1852, found 234.1847

***tert*-butyl 4-((2,6-dimethoxyphenyl)amino)piperidine-1-carboxylate (54)**

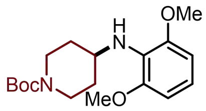

Prepared according to the **GPD** from 2,6-dimethoxyaniline (15.3 mg, 0.1 mmol) and *tert*-butyl 4-bromopiperidine-1-carboxylate (39.6 mg, 0.15 mmol), The crude residue was purified by column chromatography to yield **54** (30.3 mg, 90% yield) as a white solid.

^{1}H NMR (400 MHz, $CDCl_3$): δ 6.83 (t, *J* = 8.3 Hz, 1H), 6.53 (d, *J* = 8.3 Hz, 2H), 3.98 (brs, 2H), 3.82 (s, 6H), 3.64 – 3.52 (m, 1H), 2.87 – 2.77 (m, 2H), 1.86 (d, *J* = 10.3 Hz, 2H), 1.45 (s, 9H), 1.32 – 1.18 (m, 2H).
^{13}C NMR (101 MHz, $CDCl_3$): δ 154.8, 151.4, 125.2, 120.5, 104.4, 79.3, 55.8, 52.2, 42.8, 33.1, 28.4.
HRMS (ESI): Calcd for $C_{18}H_{29}N_2O_4^+$ $[M+H]^+$:337.2122, found 337.2114

3-(4-(cyclopropylamino)phenyl)ethan-1-ol (55)

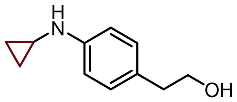

Prepared according to the **GPD** from 2-(4-aminophenyl)ethan-1-ol (13.7 mg, 0.1 mmol) and bromocyclopropane (18.1 mg, 0.15 mmol), The crude residue was purified by column chromatography to yield **55** (7.1 mg, 40% yield) as a white solid.

^{1}H NMR (400 MHz, CD_2Cl_2): δ 7.01 (d, *J* = 8.3 Hz, 2H), 6.71 (d, *J* = 8.4 Hz, 2H), 4.16 (brs, 1H), 3.72 (t, *J* = 6.6 Hz, 2H), 2.70 (t, *J* = 6.6 Hz, 2H), 2.38 (tt, *J* = 6.8, 3.6 Hz, 1H), 0.69 (td, *J* = 6.6, 4.6 Hz, 2H), 0.47 – 0.43 (m, 2H).
^{13}C NMR (101 MHz, CD_2Cl_2): δ 147.0, 129.1, 127.0, 112.7, 63.4, 37.9, 24.8, 6.7.
HRMS (ESI): Calcd for $C_{11}H_{16}NO^+$ $[M+H]^+$:178.1226, found 178.1223

N-cyclopropyl-4-(trifluoromethyl)aniline (56)

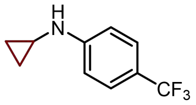

Prepared according to the **GPD** from 4-(trifluoromethyl)aniline (16.1 mg, 0.1 mmol) and bromocyclopropane (18.1 mg, 0.15 mmol), The crude residue was purified by column chromatography to yield **56** (10.1 mg, 50% yield) as a white solid. All other spectroscopic analyses were in agreement with the literature[15].

^{1}H NMR (400 MHz, CD_2Cl_2): δ 7.39 (d, *J* = 8.5 Hz, 2H), 6.80 (d, *J* = 8.5 Hz, 2H), 4.43 (brs, 1H), 2.44 (tt, *J* = 6.7, 3.6 Hz, 1H), 0.76 (td, *J* = 6.6, 4.8 Hz, 2H), 0.53 – 0.47 (m, 2H).
^{13}C NMR (101 MHz, CD_2Cl_2): δ 151.5, 126.3 (q, *J* = 3.8 Hz), 125.3 (q, *J* = 270.1 Hz), 118.7 (q, *J* = 33.1 Hz), 112.4, 24.9, 7.3.
^{19}F NMR (377 MHz, CD_2Cl_2): δ -61.2.
HRMS (ESI): Calcd for $C_{10}H_{11}F_3N^+$ $[M+H]^+$:202.0838, found 202.0830

7-ethyl-N-(oxetan-3-yl)pyridin-2-amine (57)

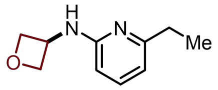

Prepared according to the **GPD** from 6-ethylpyridin-2-amine (12.2 mg, 0.1 mmol) and 3-bromooxetane (20.5 mg, 0.15 mmol), The crude residue was purified by column chromatography to yield **57** (13.4 mg, 75% yield) as a colorless oil.

^{1}H NMR (400 MHz, $CDCl_3$): δ 7.35 (t, *J* = 7.8 Hz, 1H), 6.50 (d, *J* = 7.3 Hz, 1H), 6.11 (d, *J* = 8.2 Hz, 1H), 5.02 – 4.81 (m, 4H), 4.55 (t, *J* = 5.8 Hz, 2H), 2.62 (q, *J* = 7.6 Hz, 2H), 1.23 (t, *J* = 7.6 Hz, 3H).
^{13}C NMR (101 MHz, $CDCl_3$): δ 162.5, 156.7, 138.0, 111.9, 103.7, 79.3, 47.1, 31.1, 13.6.
HRMS (ESI): Calcd for $C_{10}H_{15}N_2O^+$ $[M+H]^+$:179.1179, found 179.1175

N-(4-fluorophenyl)cycloheptanamine (58)

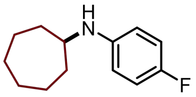

Prepared according to the **GPD** from 4-fluoroaniline (11.1 mg, 0.1 mmol) and bromocycloheptane (26.6 mg, 0.15 mmol), The crude residue was purified by column chromatography to yield **58** (9.5 mg, 46% yield) as a colorless oil.

^{1}H NMR (400 MHz, $CDCl_3$): δ 6.91 – 6.82 (m, 2H), 6.51 – 6.43 (m, 2H), 3.50 – 3.32 (m, 2H), 2.05 – 1.93 (m, 2H), 1.73 – 1.60 (m, 4H), 1.54 – 1.39 (m, 6H).
^{13}C NMR (101 MHz, $CDCl_3$): δ 155.6 (d, *J* = 234.6 Hz), 143.5, 115.6 (d, *J* = 22.4 Hz), 114.2 (d, *J* = 7.2 Hz), 54.5, 34.7, 28.3, 24.4.

^{19}F NMR (377 MHz, CDCl$_3$): δ -128.6.
HRMS (ESI): Calcd for $C_{13}H_{19}FN^+$ [M+H]$^+$:208.1496, found 208.1494

***N*-cyclohexyl-2-methoxy-6-methylaniline (59)**

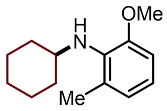

Prepared according to the **GPD** from 2-methoxy-6-methylaniline (13.7 mg, 0.1 mmol) and bromocyclohexane (24.5 mg, 0.15 mmol), The crude residue was purified by column chromatography to yield **59** (10.1 mg, 46% yield) as a colorless oil.

^{1}H NMR (400 MHz, CDCl$_3$): δ 6.83 – 6.77 (m, 1H), 6.75 – 6.67 (m, 2H), 3.81 (s, 3H), 3.55 (brs, 1H), 3.14 – 2.98 (m, 1H), 2.27 (s, 3H), 1.90 (d, *J* = 12.2 Hz, 2H), 1.78 – 1.68 (m, 2H), 1.60 (d, *J* = 10.1 Hz, 1H), δ 1.31 – 1.18 (m, 3H), 1.19 – 1.04 (m, 2H).
^{13}C NMR (101 MHz, CDCl$_3$): δ 143.8, 129.9, 127.9, 113.3, 79.3, 48.9, 20.4.
HRMS (ESI): Calcd for $C_{14}H_{22}NO^+$ [M+H]$^+$:220.1696, found 220.1690

2-methoxy-N-(1-phenoxypropan-2-yl)aniline (60)

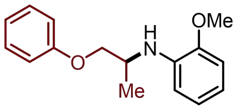

Prepared according to the **GPD** from 2-methoxyaniline (12.3 mg, 0.1 mmol) and (2-bromopropoxy)benzene (32.3 mg, 0.15 mmol), The crude residue was purified by column chromatography to yield **60** (19.0 mg, 74% yield) as a colorless oil.

^{1}H NMR (400 MHz, CDCl$_3$): δ 7.31 – 7.26 (m, 2H), 6.99 – 6.85 (m, 4H), 6.79 (d, *J* = 7.6 Hz, 1H), 6.72 – 6.65 (m, 2H), 4.39 (brs, 1H), 4.09 (d, *J* = 4.9 Hz, 1H), 3.90 (dd, *J* = 13.0, 6.3 Hz, 2H), 3.85 (s, 3H), 1.41 (d, *J* = 5.8 Hz, 3H).
^{13}C NMR (101 MHz, CDCl$_3$): δ 158.9, 146.9, 136.9, 129.4, 121.2, 120.8, 116.6, 116.2, 114.6, 110.2, 109.7, 70.9, 55.4, 47.7, 18.3.
HRMS (ESI): Calcd for $C_{16}H_{20}NO_2^+$ [M+H]$^+$:258.1489, found 258.1483

***tert*-butyl 4-((((2S,4aR,10aR)-7-isopropyl-2,4a-dimethyl-1,2,3,4,4a,9,10,10a-octahydrophenanthren-2-yl)methyl)amino)piperidine-1-carboxylate (61)**

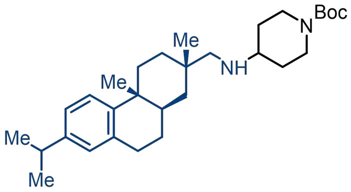

Prepared according to the **GPD** from Leelamine (28.5 mg, 0.1 mmol) and *tert*-butyl 4-iodopiperidine-1-carboxylate (46.7 mg, 0.15 mmol), The crude residue was purified by column chromatography to yield **61** (30.0 mg, 64% yield) as a colorless oil.

^{1}H NMR (400 MHz, CDCl$_3$): δ 7.18 (d, *J* = 8.2 Hz, 1H), 6.99 (d, *J* = 8.1 Hz, 1H), 6.89 (s, 1H), 3.95 (brs, 2H), 2.94 – 2.77 (m, 5H), 2.62 – 2.45 (m, 2H), 2.26 (d, *J* = 12.0 Hz, 2H), 1.85 – 1.70 (m, 6H), 1.64 – 1.60 (m, 1H), 1.45 (s, 9H), 1.44 – 1.34 (m, 5H), 1.25 – 1.21 (m, 9H), 0.90 (s, 3H).
^{13}C NMR (101 MHz, CDCl$_3$): δ 154.8, 147.6, 145.4, 134.8, 126.8, 124.3, 123.8, 79.2, 58.2, 55.3, 44.9, 42.6, 38.4, 37.4, 36.8, 36.0, 33.4, 32.6, 30.3, 28.4, 25.4, 23.9, 19.4, 18.8, 18.7.
HRMS (ESI): Calcd for $C_{30}H_{49}N_2O_2^+$ $[M+H]^+$:469.3789, found 469.3779

tert-butyl 4-((1-isobutyl-1H-imidazo[4,5-c]quinolin-4-yl)amino)piperidine-1-carboxylate (62)

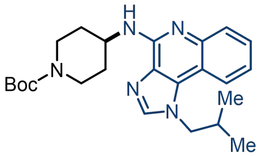

Prepared according to the **GPD** from Imiquimod (24.0 mg, 0.1 mmol) and *tert*-butyl 4-iodopiperidine-1-carboxylate (46.7 mg, 0.15 mmol), using DMF/MeCN= 9/1 as solvent. The crude residue was purified by column chromatography to yield **62** (34.3 mg, 81% yield) as a colorless oil.

^{1}H NMR (400 MHz, CDCl$_3$): δ 7.85 (d, *J* = 8.4 Hz, 2H), 7.70 (s, 1H), 7.49 (dd, 1H), 7.31 – 7.24 (m, 1H), 5.65 (d, *J* = 7.6 Hz, 1H), 4.53 – 4.40 (m, 1H), 4.24 (d, *J* = 7.4 Hz, 1H), 4.10 (brs, 1H), 3.02 (t, *J* = 10.5 Hz, 2H), 2.33 (dt, *J* = 13.6, 6.5 Hz, 1H), 2.19 (d, *J* = 9.9 Hz, 2H), 1.56 – 1.50 (m, 2H), 1.47 (s, 9H), 1.00 (d, *J* = 6.6 Hz, 6H).
^{13}C NMR (101 MHz, CDCl$_3$): δ 154.9, 149.9, 145.4, 141.8, 131.9, 128.7, 127.6, 127.2, 121.7, 119.8, 115.1, 79.4, 55.0, 47.1, 42.9, 32.4, 28.7, 28.4, 19.7.
HRMS (ESI): Calcd for $C_{24}H_{34}N_5O_2^+$ $[M+H]^+$:424.2707, found 424.2697

tert-butyl 4-(5-((3,5-dimethylphenoxy)methyl)-2-oxooxazolidin-3-yl)piperidine-1-carboxylate (63)

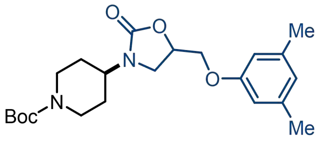

Prepared according to the **GPD** from Metaxalone (22.1 mg, 0.1 mmol) and *tert*-butyl 4-iodopiperidine-1-carboxylate (46.7 mg, 0.15 mmol), The crude residue was purified by column chromatography to yield **63** (34.4 mg, 85% yield) as a colorless oil.

^{1}H NMR (400 MHz, CDCl$_3$): δ 6.63 (s, 1H), 6.50 (s, 2H), 4.84 – 4.76 (m, 1H), 4.27 – 4.16 (m, 2H), 4.11 – 4.01 (m, 2H), 3.94 – 3.82 (m, 1H), 3.63 (t, *J* = 8.7 Hz, 1H), 3.48 (dd, *J* = 8.6, 5.6 Hz, 1H), 2.84 – 2.70 (m, 2H), 2.27 (s, 6H), 1.77 (d, *J* = 9.7 Hz, 2H), 1.62 – 1.52 (m, 2H), 1.45 (s, 9H).
^{13}C NMR (101 MHz, CDCl$_3$): δ 158.1, 156.8, 154.5, 139.4, 123.3, 112.2, 79.8, 71.0, 67.9, 50.9, 42.7, 29.2, 29.0, 28.3, 21.3.

HRMS (ESI): Calcd for $C_{22}H_{33}N_2O_5^+$ [M+H]$^+$:405.2384, found 405.2375

4-methyl-N-(((3aR,5R,5aS,8aS,8bR)-2,2,7,7-tetramethyltetrahydro-5H-bis([1,3]dioxolo)[4,5-b:4',5'-d]pyran-5-yl)methyl)aniline (64)

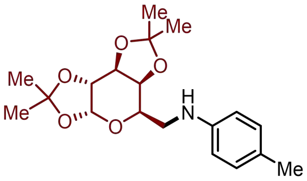

Prepared according to the **GPD** from *p*-toluidine (10.7 mg, 0.1 mmol) (22.1 mg, 0.1 mmol) and 5-(iodomethyl)-2,2,7,7-tetramethyltetrahydro-5H-bis([1,3]dioxolo)[4,5-b:4',5'-d]pyran (46.7 mg, 0.15 mmol), The crude residue was purified by column chromatography to yield **64** (28.3 mg, 81% yield) as a colorless oil.

^{1}H NMR (400 MHz, CDCl$_3$): δ 6.97 (d, J = 8.0 Hz, 2H), 6.59 (d, J = 8.2 Hz, 2H), 5.55 (d, J = 5.0 Hz, 1H), 4.61 (dd, J = 7.9, 2.4 Hz, 1H), 4.32 (dd, J = 5.1, 2.4 Hz, 1H), 4.24 (dd, J = 7.9, 1.9 Hz, 1H), 4.04 – 3.98 (m, 1H), 3.86 (brs, 1H), 3.43 – 3.23 (m, 2H), 2.22 (s, 3H), 1.47 (s, 3H), 1.38 (s, 3H), 1.36 (s, 3H), 1.31 (s, 3H).
^{13}C NMR (101 MHz, CDCl$_3$): δ 145.8, 129.7, 126.9, 113.6, 109.4, 108.7, 96.4, 71.8, 70.8, 70.6, 65.7, 44.5, 26.0, 25.8, 25.0, 24.4, 20.3.
HRMS (ESI): Calcd for $C_{19}H_{28}NO_5^+$ [M+H]$^+$:350.1962, found 350.1953

1-(8,10,13-trimethyl-3-(p-tolylamino)-2,3,4,7,8,9,10,11,12,13,14,15,16,17-tetradecahydro-1H-cyclopenta[a]phenanthren-17-yl)ethan-1-one (65)

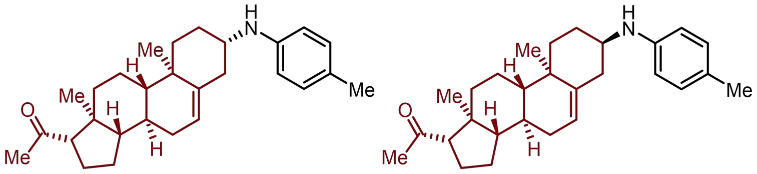

Prepared according to the **GPD** from *p*-toluidine (10.7 mg, 0.1 mmol) (22.1 mg, 0.1 mmol) and 1-((8S,9S,10R,13S,17R)-3-iodo-8,10,13-trimethyl-2,3,4,7,8,9,10,11,12,13,14,15,16,17-tetradecahydro-1H-cyclopenta[a]phenanthren-17-yl)ethan-1-one (64.0 mg, 0.15 mmol), using $PhCF_3$/MeCN= 9/1 as solvent. The crude residue was purified by column chromatography to yield **65** (34.1 mg, 84% yield) as an inseparable mixture of diastereomers. dr = 4.4:1.

^{1}H NMR (400 MHz, CDCl$_3$, diastereomers): δ 6.97 (d, J = 8.1 Hz, 2H), 6.52 (d, J = 8.2 Hz, 2H), 5.39 – 5.35 (m, 1H), 3.23 – 3.11 (m, 0.90H), 2.73 – 2.42 (m, 2.04H), 2.25 – 2.17 (m, 4.01H), 2.15 – 2.11 (m, 3.20H), 2.09 – 1.96 (m, 4.15H), 1.94 – 1.87 (m, 1.17H), 1.76 – 1.60 (m,4.32H), 1.58 – 1.17 (m, 10.11H), 1.07 – 1.00 (m, 4.04H), 0.70 – 0.61 (m, 3.01H).
^{13}C NMR (101 MHz, CDCl$_3$, diastereomers): δ 209.6, 145.0, 144.8, 141.4, 139.2, 129.8, 129.8, 126.4, 126.0, 123.0, 121.0, 113.7, 113.5, 63.7, 57.0, 53.7, 50.4, 50.2,

48.4, 44.0, 40.1, 38.9, 38.9, 38.3, 37.7, 37.3, 37.0, 33.8, 31.9, 31.9, 31.8, 31.6, 29.7, 24.6, 24.5, 22.9, 21.1, 20.8, 20.4, 19.5, 19.0, 13.3, 13.2.

HRMS (ESI): Calcd for $C_{28}H_{40}NO^+$ $[M+H]^+$:406.3104, found 406.3095

4. Mechanistic Studies

A. Investigating into the active Cu(I) species during photocatalytic process

4.1 UV-vis absorption of in-situ generated copper complex

The UV-vis spectra of single component (1.25×10^{-4} M) was measured directly in MeCN. A mixture of components (the amount of component is equal to each other) was stirred for 30 min before measurement in 10 mm pathlength quartz cuvette.

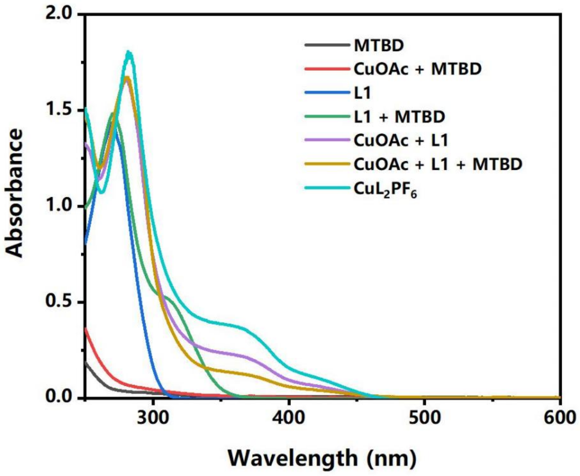

Supplementary Figure 3. UV-vis absorption spectra of reaction complexes in MeCN

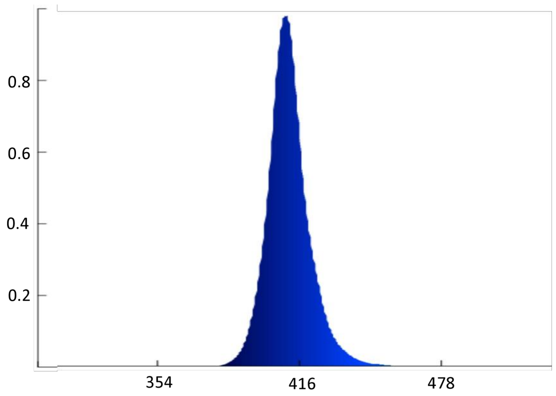

Supplementary Figure 4. Emission spectra of 410 nm LEDs

4.2 Synthesis of pyridyl-carbene ligated copper(I) complex ex situ

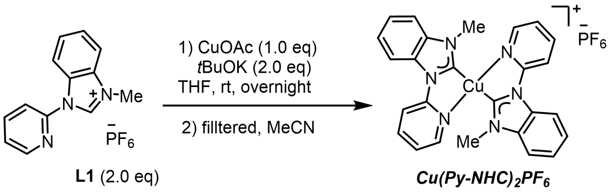

Experimental Procedure: To an oven-dried 5 mL reaction vial were added L1 (213 mg, 0.6 mmol, 2.0 equiv), and *t*BuOK (67.3mg, 0.6 mmol, 2.0 equiv), 2 mL THF in a nitrogen-filled glove box. The resulting mixture was stirred for 5 min, followed by adding CuOAc (36.6 mg, 0.3 mmol, 1.0 equiv) and sealed with a screwed cap stirred for overnight. The resulting orange solid was filtered and washed with Et_2O, then redissolved in anhydrous MeCN and the solution was filtered. Finally, removed solvent in vacuo and yellow solid (130 mg, 72% yield) was obtained.

Cu(Py-NHC)$_2$PF$_6$: ^{1}H NMR (400 MHz, CD_3CN): δ 8.35 – 7.94 (m, 8H), 7.68 – 7.61 (m, 2H), 7.54 – 7.47 (m, 4H), 7.41 – 7.30 (m, 2H), 3.93 (s, 6H).
^{13}C NMR (101 MHz, CD_3CN): δ 194.6, 152.2, 149.7, 141.2, 137.3, 132.3, 125.5, 125.4, 123.9, 115.5, 113.8, 113.2, 36.6.
^{19}F NMR (377 MHz, CD_3CN) δ -72.9 (d, J = 706.8 Hz).
^{31}P NMR (162 MHz, CD_3CN): δ-144.6 (hept, 696.6 Hz)

Supplementary Table 5. Diffraction data of Cu(Py-NHC)$_2$PF$_6$ (**CCDC 2224303**)

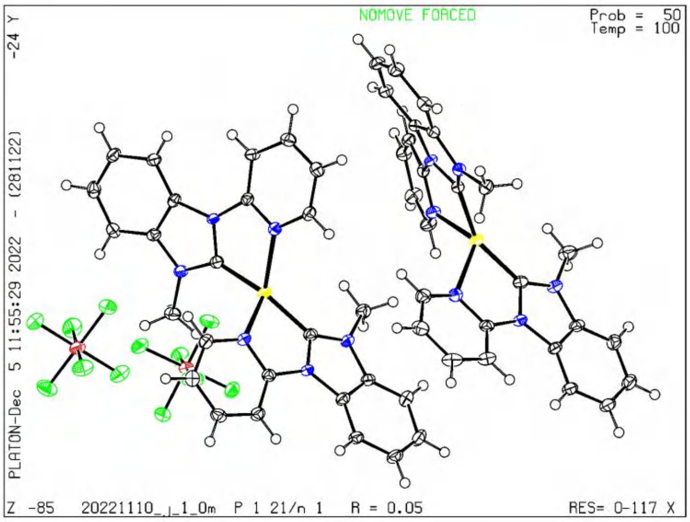

Identification code	Cu (Py-NHC)$_2$PF$_6$ complex
	**Crystal data**
Chemical formula	$C_{26}H_{22}CuN_6 \cdot F_6P$
Mr	627.00

Crystal system, space group	Monoclinic, $P2_1/n$
Temperature (K)	100
a, b, c (Å)	17.5287 (8), 13.4713 (6), 23.4942 (9)
β (°)	110.637 (2)
V (Å^3)	5191.8 (4)
Z	8
Radiation type	Mo $K\alpha$
μ (mm^{-1})	0.98
Crystal size (mm)	0.05 × 0.05 × 0.05
**Data collection**	
Diffractometer	Bruker *APEX*-II CCD
Absorption correction	Multi-scan
	SADABS2016/2 (Bruker,2016/2) was used for absorption correction. wR2(int) was 0.1558 before and 0.0792 after correction. The Ratio of minimum to maximum transmission is 0.8559. The λ/2 correction factor is Not present.
T_{min}, T_{max}	0.638, 0.746
No. of measured, independent and observed [$I > 2\sigma(I)$] reflections	54293, 14592, 9587
R_{int}	0.085
$(\sin \theta/\lambda)_{max}$ (Å^{-1})	0.696
**Refinement**	
$R[F^2 > 2\sigma(F^2)]$, $wR(F^2)$, S	0.046, 0.131, 1.06
No. of reflections	14592
No. of parameters	725
H-atom treatment	H-atom parameters constrained
$\Delta\rho_{max}$, $\Delta\rho_{min}$ (e Å^{-3})	0.63, −0.67

Discussion: To figure out the active Cu complex, we tried to synthesize

pyridyl-carbene ligated **Cu-1** complex ex-situ. In the presence of *t*BuOK, a stable Cu(Py-NHC)$_2$PF$_6$ was obtained, which was confirmed by NMR, HRMS and X-ray spectra.

4.3 Electronchemical of Cu(Py-NHC)$_2$PF$_6$

Cyclic voltammograms were recorded using a CH Instruments 656E potentiostat, a glassy carbon working electrode, a Ag/AgCl reference electrode, and a Pt counter electrode. The voltammograms were recorded at room temperature under nitrogen atmosphere in 0.1 M Bu_4NPF_6 in MeCN containing samples (1 mM). Scan rate = 0.1 V/s, All potentials are reported in V vs SCE. The $E_{1/2}$ of the Fc/Fc$^+$ redox couple used as a standard is 0.42 V vs SCE in MeCN.

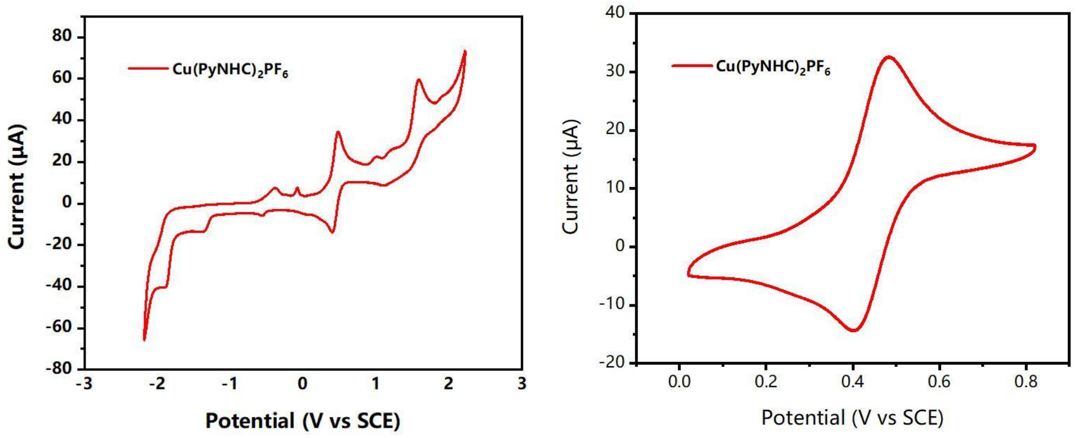

Supplementary Figure 5. Cyclic voltammogram of Cu(Py-NHC)$_2$PF$_6$

4.4 HRMS analysis of Cu(Py-NHC)$_2$PF$_6$ and in situ generated copper complex

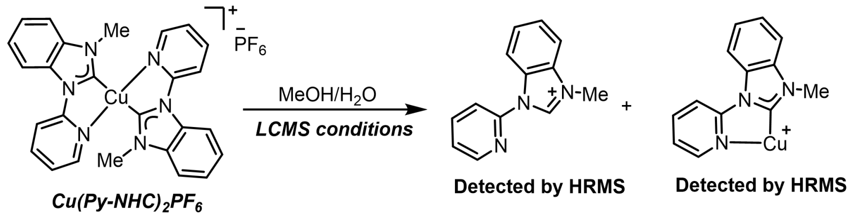

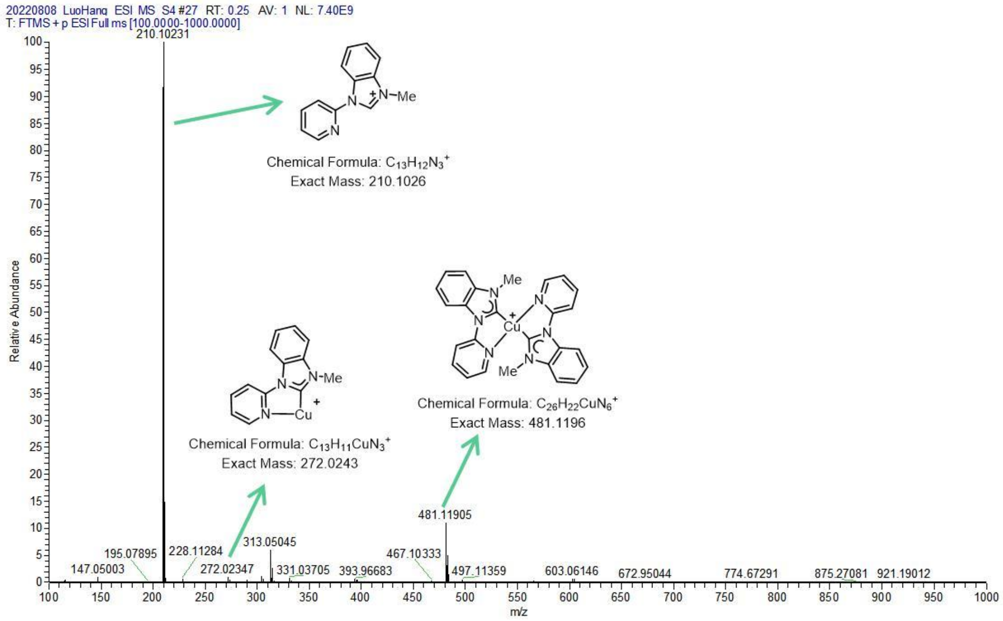

Supplementary Figure 6. HRMS analysis of Cu(Py-NHC)$_2$PF$_6$ in presence of MeOH

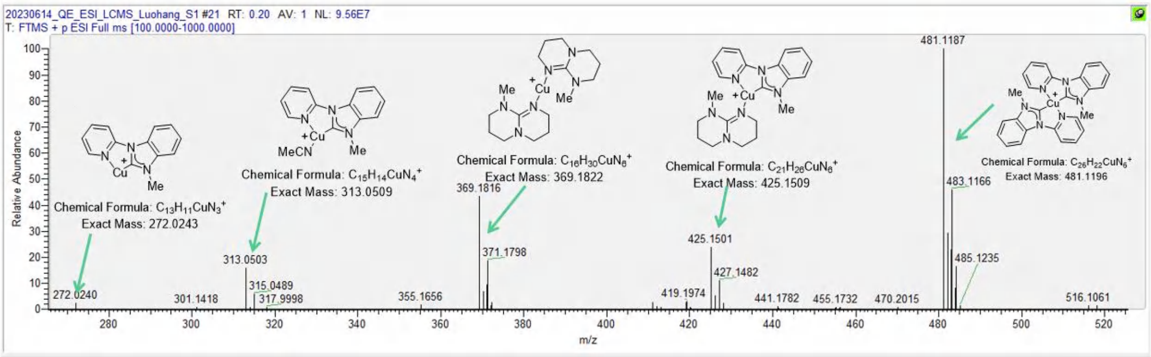

Supplementary Figure 7. HRMS analysis of in-situ generated copper complex in MeCN

Discussion: Cu(Py-NHC)$_2$PF$_6$ complex showed the ability to dissociate one ligand, forming a mono-Py-NHC-ligated Cu(I) complex (Cu-1) when dissolved in solution.

4.5 ^{1}H NMR analysis of in-situ generated copper complex

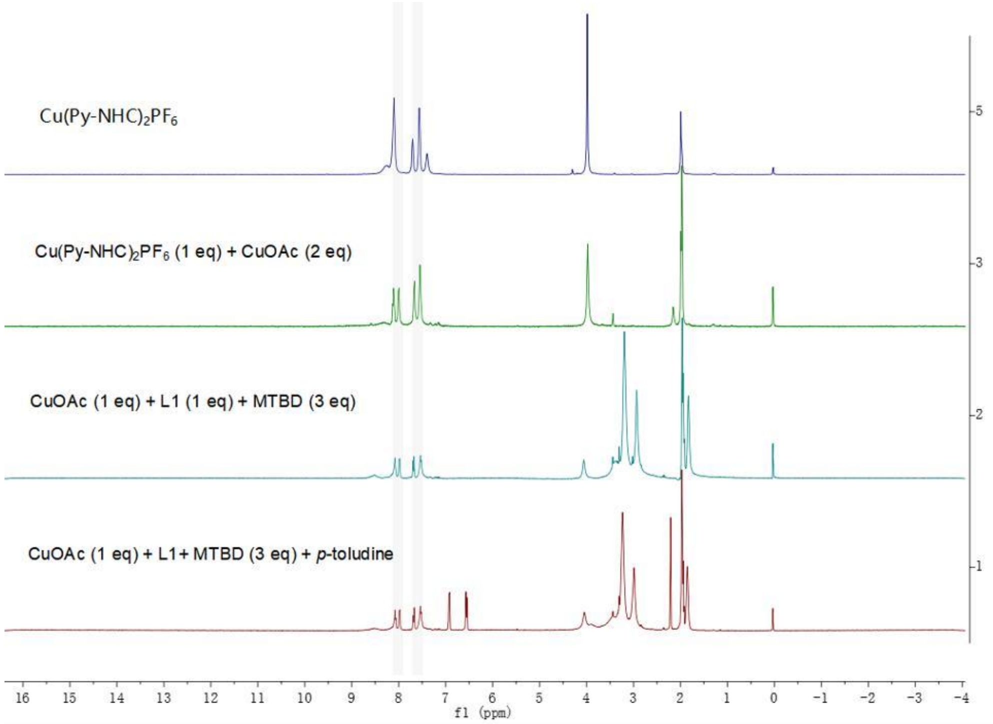

Supplementary Figure 8. ^{1}H NMR analysis of in-situ generated Cu(I) complex in different conditions in Acetonitrile-*d3*

Discussion: The results indicated that mono-PyNHC ligated **Cu-1** complex can be in situ generated by mix of CuOAc and PyNHC ligand and/orMTBD, and Cu(Py-NHCs)$_2$PF$_6$ can be easily converted into mono-Py-NHC ligated **Cu-1** complex in the presence of the excess copper acetate, and in Acetonitrile-*d3*

4.6 The reactivity of $Cu(Py\text{-}NHC)_2PF_6$ for C-N coupling

Supplementary Table 6. Investigation of the actual Cu(I) species

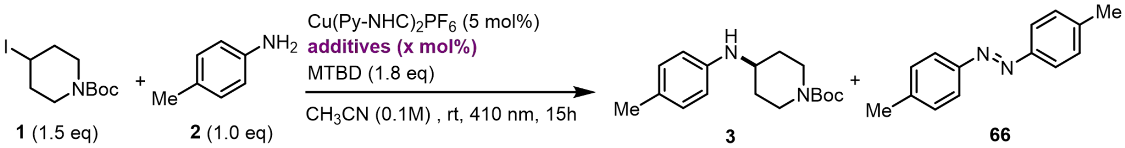

entry	Additives	**3** (%)	**66** (%)
1	none	15	N.D.
2	CuOAc (20 mol%)	94	N.D.
3	LiOAc or NaOAc (20 mol%)	15	N.D.
4	$Zn(OAc)_2$ (20 mol%)	87	N.D.
5	CuI (20 mol%)	32	33

Experimental Procedure: To an oven-dried 10 mL reaction vial were added $Cu(Py\text{-}NHc)_2PF_6$ (3.1 mg, 0.005 mmol, 5 mol%), additives (0.02 mmol, 20 mol%), and 1 mL MeCN in a nitrogen-filled glove box. The resulting mixture was stirred for 5 min, followed by adding 7-Methyl-1,5,7-triazabicyclo[4.4.0]dec-5-ene (MTBD) (26 μL, 0.18 mmol, 1.8 equiv), *p*-toluidine (10.7 mg 0.1 mmol, 1.0 equiv), *tert*-butyl 4-iodopiperidine-1-carboxylate (46.7 mg, 0.15 mmol) in sequence, and sealed with a screwed cap. The sealed vial was placed on a photo-reactor under irradiation of LEDs (410 nm, 6 W). The mixture was stirred at 25°C for 15 h, quenched with H_2O, and extracted with ethyl acetate. The combined organic layers were dried over anhydrous Na_2SO_4, concentrated in vacuo. The crude product was analyzed by ^1H NMR with 1,3,5-Trimethoxybenzene as an internal standard.

Discussion: The reactivity of $Cu(Py\text{-}NHC)_2PF_6$ is lower than the mono-pyridyl-carbene ligated **Cu-1** complex in situ generated for couplings. The reactivity was enhanced by adding CuOAc, $Zn(OAc)_2$ implying that the $Cu(Py\text{-}NHC)_2PF_6$ might decompose to form mono-pyridyl-carbene **Cu-1** complex. The HRMS result also revealed that the [Cu^I(Py-NHC)] complex can be detected with releasing the pyridyl-carbene ligand in the presence of methanol.

B. Alkyl radical species trapping experiments

4.7 TEMPO trapping experiments

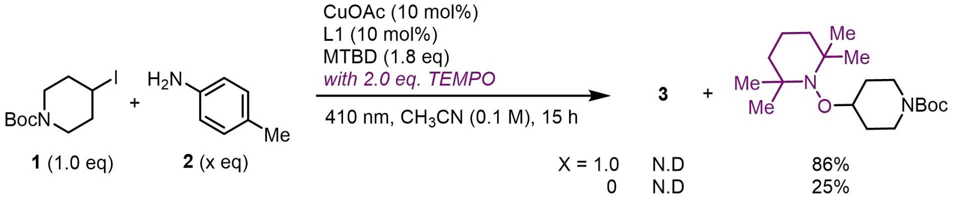

Experimental Procedure: To an oven-dried 10 mL reaction vial were added CuOAc (1.2 mg, 0.01 mmol, 10 mol%), L1 (3.55 mg, 0.01 mmol, 10 mol%), and 1 mL MeCN in a nitrogen-filled glove box. The resulting mixture was stirred for 5 min, followed by adding 7-Methyl-1,5,7-triazabicyclo[4.4.0]dec-5-ene (MTBD) (26 µL, 0.18 mmol, 1.8 equiv), with/without *p*-toluidine (10.7 mg 0.1 mmol, 1.0 equiv), *tert*-butyl 4-iodopiperidine-1-carboxylate (31.1 mg, 0.1 mmol), TEMPO (31.3 mg, 0.2 mmol, 2 equiv) in sequence, and sealed with a screwed cap. The sealed vial was placed on a photo-reactor under irradiation of LEDs (410 nm, 6 W). The mixture was stirred at 25 °C for 15 h, quenched with H_2O, and extracted with ethyl acetate. The combined organic layers were dried over anhydrous Na_2SO_4, concentrated in vacuo. The crude product was analyzed by ^{1}H NMR with 1,3,5-Trimethoxybenzene as an internal standard.

4.8 Radical clock experiments: involvement of alkyl radicals

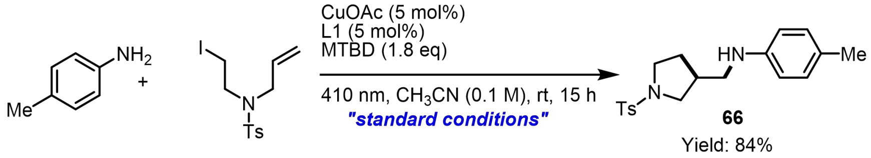

Experimental Procedure: To an oven-dried 10 mL reaction vial were added CuOAc (0.6 mg, 0.005 mmol, 5 mol%), L1 (1.8 mg, 0.005 mmol, 5 mol%), and 1 mL MeCN in a nitrogen-filled glove box. The resulting mixture was stirred for 5 min, followed by adding 7-Methyl-1,5,7-triazabicyclo[4.4.0]dec-5-ene (MTBD) (26 µL, 0.18 mmol, 1.8 equiv), *p*-toluidine (10.7 mg 0.1 mmol, 1.0 equiv), *N*-allyl-*N*-(2-iodoethyl)-4-methylbenzenesulfonamide (54.8 mg, 0.15 mmol, 1.5 equiv), in sequence, and sealed with a screwed cap. The sealed vial was placed on a photo-reactor under irradiation of LEDs (410 nm, 6 W). The mixture was stirred at 25 °C for 15 h, quenched with H_2O, and extracted with ethyl acetate. The crude product was purified by silica gel column chromatography to afford the product (28.9 mg, 84% yield).

4-methyl-*N*-((1-tosylpyrrolidin-3-yl)methyl)aniline (66)

^{1}H NMR (400 MHz, $CDCl_3$): δ 7.72 (d, J = 8.0 Hz, 2H), 7.31 (d, J = 7.9 Hz, 2H), 6.97 (d, J = 8.1 Hz, 2H), 6.44 (d, J = 8.1 Hz, 2H), 3.49 (brs, 1H), 3.41 – 3.32 (m, 2H), 3.26 – 3.17 (m, 1H), 3.09 – 3.03 (m, 1H), 2.97 – 2.84 (m, 1H), 2.43 (s, 3H), 2.40 – 2.33 (m, 1H), 2.23 (s, 3H), 2.03 – 1.91 (m, 1H), 1.66 – 1.53 (m, 1H).

^{13}C NMR (101 MHz, CDCl$_3$): δ 145.4, 143.5, 133.3, 129.7, 129.6, 127.5, 126.8, 112.8, 51.4, 47.2, 46.8, 38.2, 29.0, 21.5, 20.3.

HRMS (ESI): Calcd for $C_{19}H_{25}N_2O_2S^+$ $[M+H]^+$:345.1631, found 345.1626

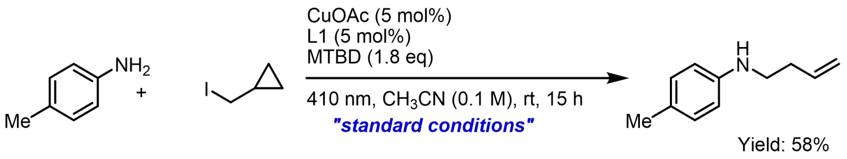

Experimental Procedure: To an oven-dried 10 mL reaction vial were added CuOAc (0.6 mg, 0.005 mmol, 5 mol%), L1 (1.8 mg, 0.005 mmol, 5 mol%), and 1 mL MeCN in a nitrogen-filled glove box. The resulting mixture was stirred for 5 min, followed by adding 7-Methyl-1,5,7-triazabicyclo[4.4.0]dec-5-ene (MTBD) (26 µL, 0.18 mmol, 1.8 equiv), *p*-toluidine (10.7 mg 0.1 mmol, 1.0 equiv), (iodomethyl)cyclopropane (27.3 mg, 0.15 mmol) in sequence, and sealed with a screwed cap. The sealed vial was placed on a photo-reactor under irradiation of LEDs (410 nm, 6 W). The mixture was stirred at 25 °C for 15 h, quenched with H_2O, and extracted with ethyl acetate. The combined organic layers were dried over anhydrous Na_2SO_4, concentrated in vacuo. The crude product was purified by silica gel column chromatography to afford the product (9.4 mg, 58% yield).

***N*-(but-3-en-1-yl)-4-methylaniline**

^{1}H NMR (400 MHz, CDCl$_3$): δ 6.99 (d, J = 8.0 Hz, 2H), 6.55 (d, J = 8.4 Hz, 2H), 5.88 – 5.75 (m, 1H), 5.18 – 5.07 (m, 2H), 3.55 (brs, 1H), 3.17 (t, J = 6.7 Hz, 2H), 2.38 (q, J = 6.7 Hz, 1H), 2.24 (s, 3H).

All other spectroscopic analyses were in agreement with the literature[16].

C. Investigation of Cu(II) intermediate for C-N coupling

4.9 Exploration of copper(II) as the one of possible intermediates

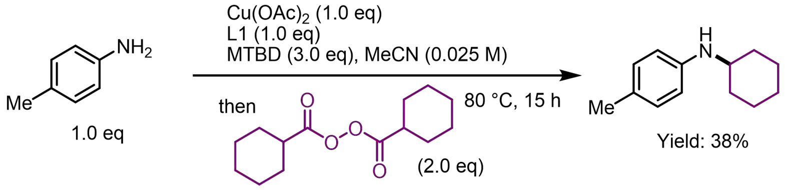

Experimental Procedure: To an oven-dried 10 mL reaction vial were added CuOAc (12.3 mg, 0.1 mmol, 1.0 equiv), L1 (42.6 mg, 0.1 mmol, 1.0 equiv), and 4 mL MeCN in a nitrogen-filled glove box. The resulting mixture was stirred for 5 min, followed by adding 7-Methyl-1,5,7-triazabicyclo[4.4.0]dec-5-ene (MTBD) (43 μL, 0.3 mmol, 3.0 equiv), *p*-toluidine (10.7 mg 0.1 mmol, 1.0 equiv), cyclohexanecarboxylic peroxyanhydride (50.9 mg, 0.2 mmol, 2.0 equiv) in sequence, and sealed with a screwed cap. The sealed vial was stirred at 80 °C for 15 h, then quenched with H_2O, and extracted with ethyl acetate. The combined organic layers were dried over anhydrous Na_2SO_4, concentrated in vacuo. The crude product was analyzed by ^1H NMR with 1,3,5-Trimethoxybenzene as an internal standard.

4.10 EPR data collection and analysis

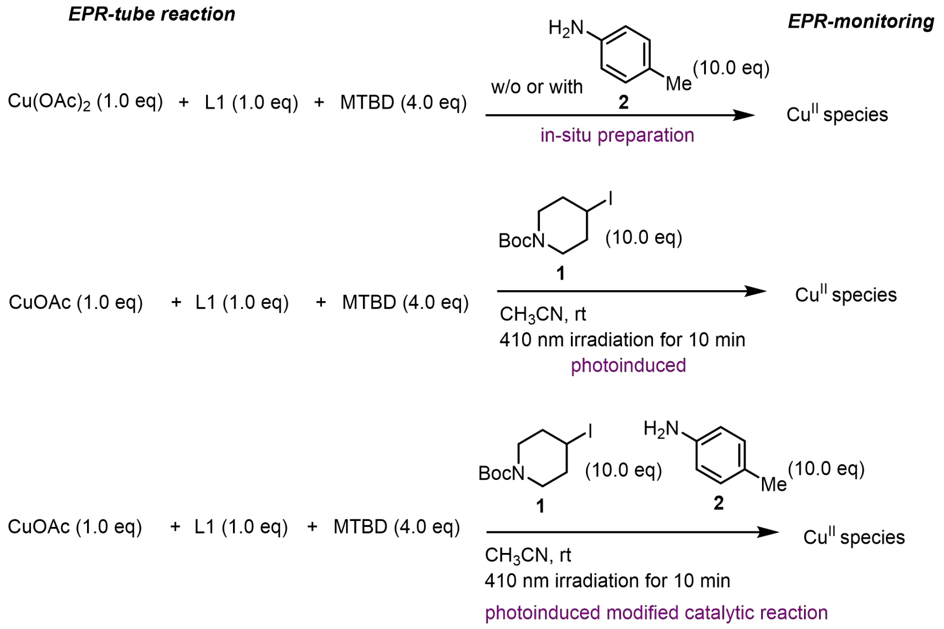

Experimental detail: To an oven-dried 10 mL reaction vial were added Cu salt (,0.1 mmol, 1.0 equiv), L1 (42.6 mg, 0.1 mmol, 1.0 equiv), 7-Methyl-1,5,7-triazabicyclo[4.4.0]dec-5-ene (MTBD) 58 μL, 0.4 mmol, 4.0 equiv), *p*-toluidine (107 mg 0.1 mmol, 10.0 equiv), R-I (4-iodopiperidine-1-carboxylate) (311 mg, 1.0 mmol, 10.0 equiv) and 3 mL MeCN in a nitrogen-filled glove box. After stirred for 5 min, the resulting homogenous solution was transferred to EPR tubes (100 μL per tube). The tubes were sealed with a rubber stopper and removed from the

glovebox. The sealed tube was placed on a photo-reactor under irradiation of LEDs (410 nm, 50 W) for 10 minutes and then immediately frozen at 100 K. The samples were analyzed by X-band spectroscopy.

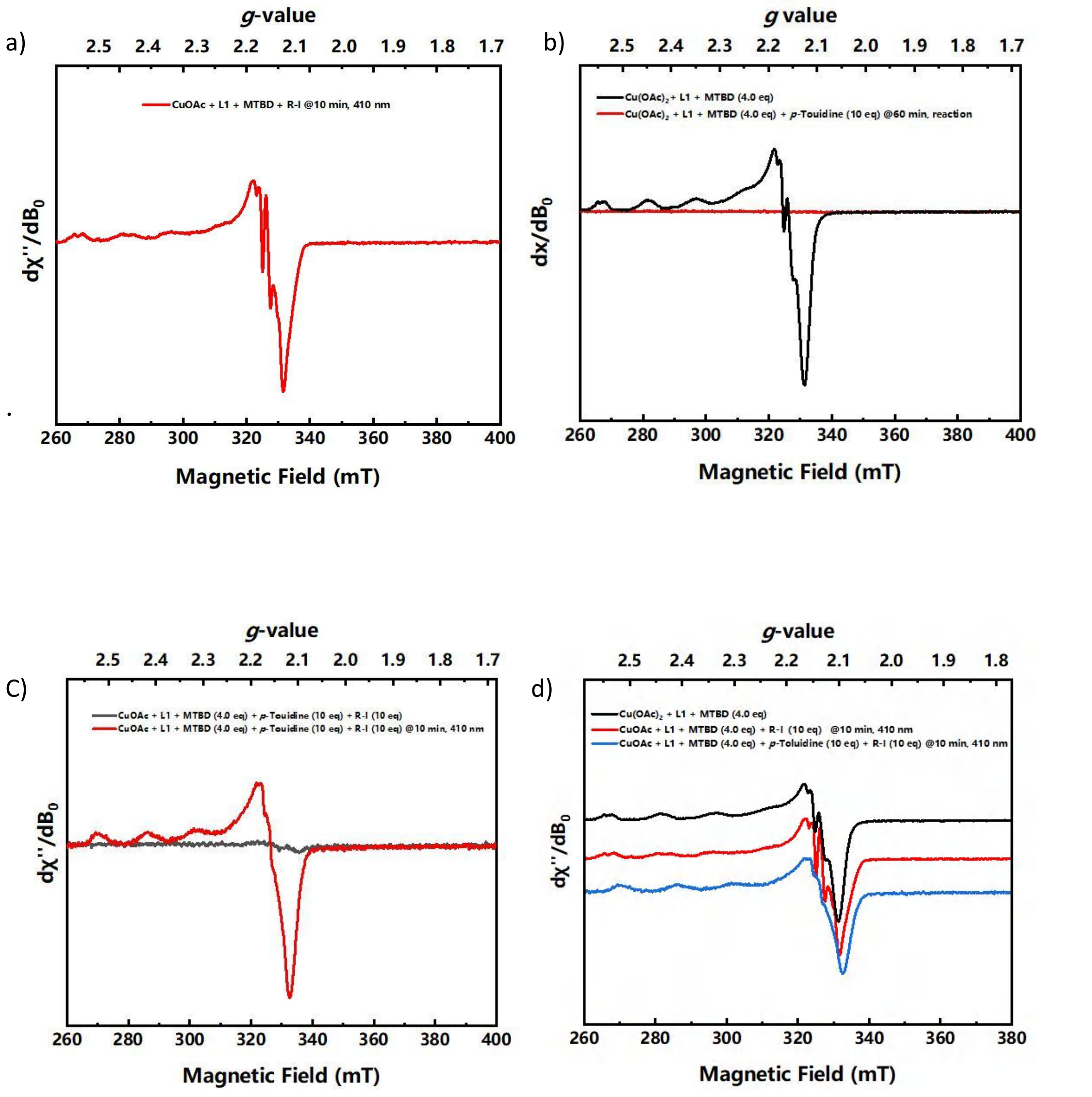

Supplementary Figure 9. X-band EPR spectra of reaction complex generated in tube from a) CuOAc, L1, MTBD, R-I (4-iodopiperidine-1-carboxylate) after 10 min of 410 nm irradiation (red trace). b) $Cu(OAc)_2$, L1, MTBD (black trace) and with *p*-toluidine after 60 min of 410 nm irradiation (red trace). c) modified catalytic condition: CuOAc, L1, MTBD, *p*-toluidine, R-I (black trace) and after 10 min of 410 nm irradiation (red trace). d) $Cu(OAc)_2$, L1, MTBD (black trace), modified catalytic condition without *p*-toluidine after 10 min of light irradiation (red trace), modified catalytic condition after 10 min of light irradiation (blue trace). Acquisition parameters : MW frequency = 9.404939 GHz; temperature = 100 K; MW power = 0.7962 mW; modulation amplitude = 0.1 mT; conversion time = 18.75 ms.

D. Inner-Sphere SET Process Investigation

4.11 Transient absorption experiments

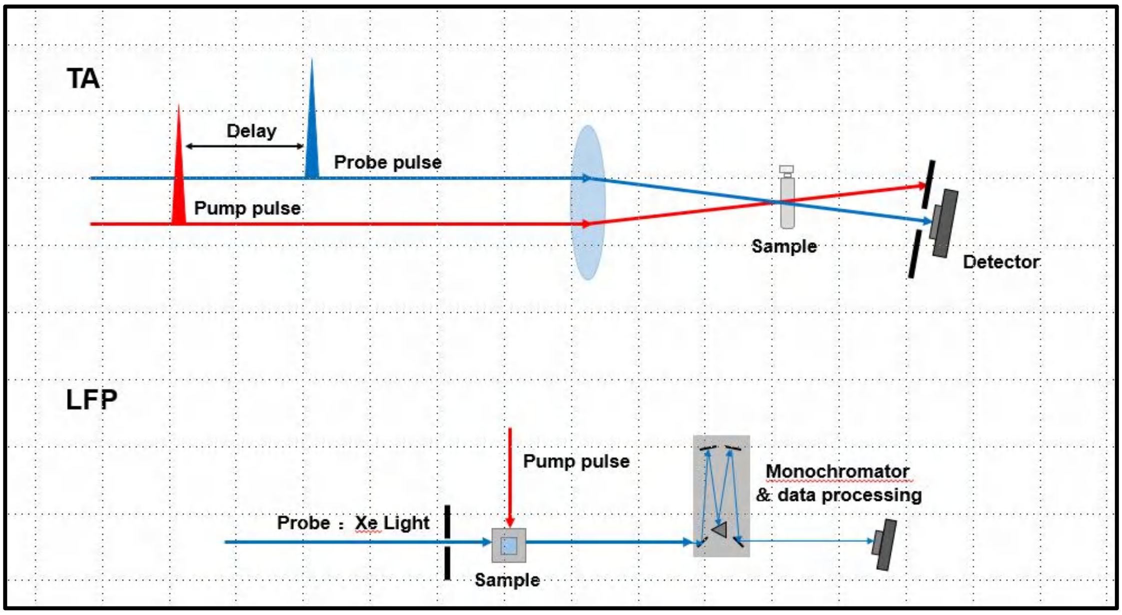

Supplementary Figure 10. Structural diagram of Transient absorption experiments

TA experiment set-ups

Femtosecond TA. The femtosecond pump-probe TA measurements were performed using a regenerative amplified Ti:sapphire laser system (Coherent; 800 nm, 70 fs, 6 mJ/pulse, and 1 kHz repetition rate) as the laser source and a femto-TA100 spectrometer (Time-Tech Spectra). Briefly, the 800 nm output pulse from the regenerative amplifier was split in two parts with a 50% beam splitter. The transmitted part was used to pump a TOPAS Optical Parametric Amplifier (OPA) which generated a wavelength-tunable laser pulse from 250 nm to 2.5 μm as pump beam. The reflected 800 nm beam was split again into two parts. One part with less than 10% was attenuated with a neutral density filter and focused into a 2 mm thick sapphire window to generate a white light continuum (WLC) used for probe beam. The probe beam was focused with an Al parabolic reflector onto the sample. After the sample, the probe beam was collimated and then focused into a fiber-coupled spectrometer with CMOS sensors and detected at a frequency of 1 KHz. The intensity of the pump pulse used in the experiment was controlled by a variable neutral-density filter wheel. The delay between the pump and probe pulses was controlled by a motorized delay stage. The samples were placed in 1 mm airtight cuvettes in a N_2-filled glove box and measured under ambient conditions.

Nanosecond Laser Flash Photolysis (LFP). Nanosecond time-resolved transient absorption measurements were collected on a platform from Beijing optically century instruments where ΔA remained linear up to 0.8 O.D. in single wavelength acquisition mode detecting at 430 nm. The sample solutions were prepared to possess optical densities of 0.1 at 355 nm. Excitation of the samples in these experiments was

accomplished using a Nd:YAG laser system from Beamtech Optronics Co., Ltd. (Nimama-900) operating at 1 Hz. The incident laser power was varied using a series of neutral density filters along with appropriate long pass and short pass filters to clean the excitation pulse. The same spectrometer was also equipped with an CCD detector from Beijing optically century instruments that permitted the collection of time-resolved absorption and emission spectra.

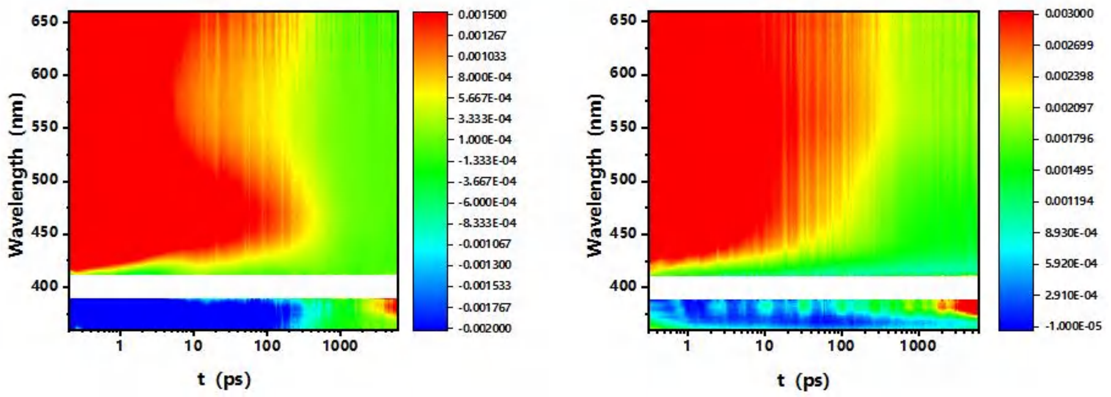

Supplementary Figure 11. Summary of Two-dimensional pseudo-colour TA spectra of in-situ generated **Cu-1** complex (λ_{pump} = 400 nm) (left) and in presence of MTBD (right) measured by ultrafast transient absorption (TA)

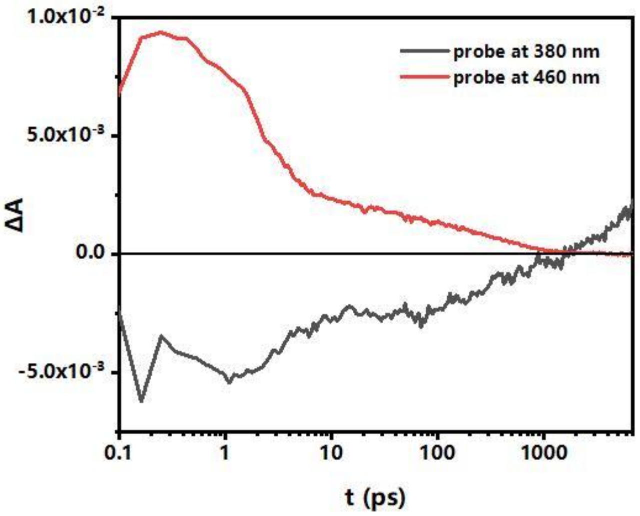

Supplementary Figure 12. Single-wavelength TA (ΔA) kinetics study (λ_{probe} = 460 nm and λ_{probe} = 380 nm)

Discussion: Ultrafast transient absorption data revealed the initial short-lived ^{1}MLCT excited-state absorption of in situ generated **Cu-1** complex, whereas the broadband from ~420 nm to > 650 nm. Single-wavelength TA (ΔA) kinetics data revealed the rapid decay of the short-lived ^{1}MLCT excited-state absorption feature at 460 nm (red) , which is accompanied by the generation of a new long-live transient state feature at 380 nm (black).

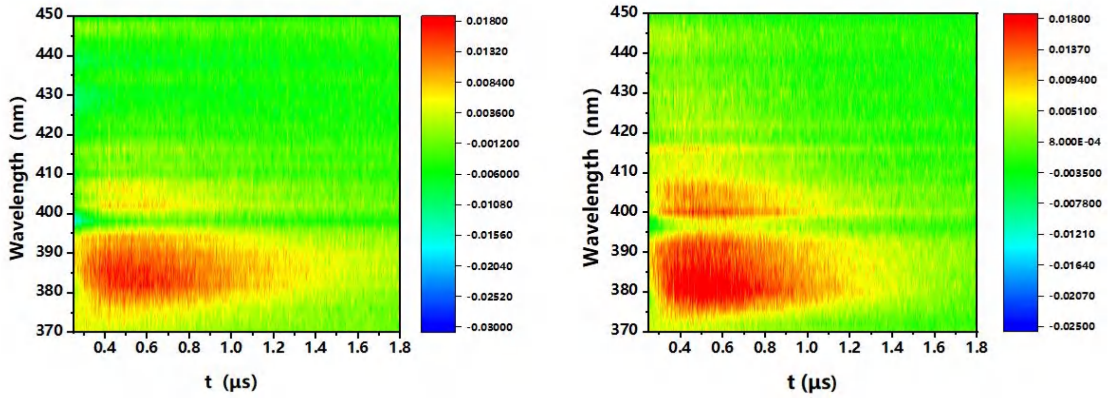

Supplementary Figure 13. Summary of Two-dimensional pseudo-colour TA spectra of in-situ generated **Cu-I** complex (λ_{pump} = 355 nm) (left) and in presence of MTBD (right) measured by Laser flash photolysis (LFP)

Discussion: Nanosecond Laser flash photolysis (LFP) data suggested that initial short-lived ^{1}MLCT state of in-situ generated **Cu-1** converts to a long-lived ^{3}MLCT state (τ ~2.1 μs)) through fast intersystem crossing (ISC) (τ ~ 260 ps)

4.12 Lifetime quenching experiments

Experimental Procedure: Rigorously degassed solutions of each component were prepared under atmosphere prior to each set of experiments. In a typical experiments, a 0.5 mM solution of in situ generated **Cu-1** complex: CuOAc (1.0 eq), L1 (1.0 eq) in MeCN was added in increasing amounts (0 mM, 20 mM, 50 mM, 10 mM, 15 mM) of quencher (iodocyclohexane) in a 1 mm quartz cuvette. The lifetime of non-emissive excited state of Cu-1 complex as function of electrophile concentration was measured at room temperature by transient absorption spectroscopy

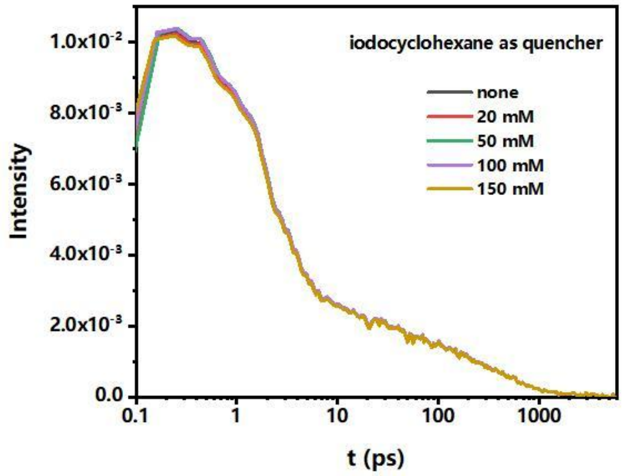

Supplementary Figure 14. TA lifetime of in situ generated **Cu-1** complex (^{1}MLCT) in the presence of various concentrations of iodocylohexane (λ_{pump} = 400 nm, λ_{probe} = 460 nm)

Discussion: no obvious data changes in the decay of short-lived ^{1}MLCT absorption at 460 nm indicated that charge transfer did not occur for singlet state.

Experimental Procedure: Rigorously degassed solutions of each component were prepared under atmosphere prior to each set of experiments. In a typical experiments, a 0.2 mM solution of in situ generated **Cu-1** complex: CuOAc (1.0 eq), L1 (1.0 eq), with/wihout MTBD (1.2 eq) in MeCN was added in increasing amounts (0 mM, 2.0 mM, 4.0 mM, 8 mM, 15.0 mM, 16.0 mM) of quencher (iodocyclohexane) in a 10 mm quartz cuvette. The lifetime of non-emissive excited state of **Cu-1** complex as function of electrophile concentration was measured at room temperature by Laser flash photolysis (LFP).

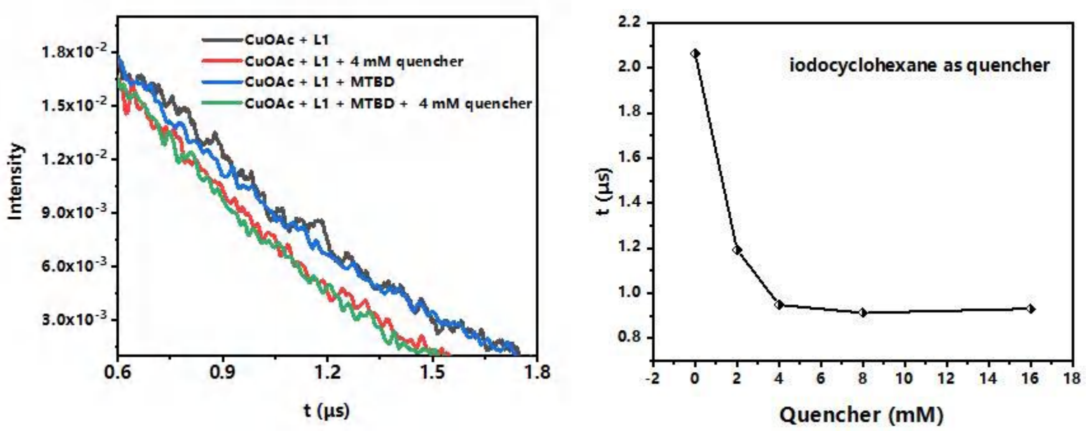

Supplementary Figure 15. LFP lifetime of of **Cu-1** (^{3}MLCT) in the presence of iodocylohexane (quencher) and/or MTBD (λ_{pump} = 355 nm, λ_{probe} = 385 nm).

Discussion: The addition of 4 mM iodocyclohexane resulted the life time of in the long-lived ^{3}MLCT absorption is shorted from 2.1 μs to 0.9 μs. This suggests that the dominant electron transfer process for the photoinduced reduction of the electrophiles occur for triplet state of **Cu-1**

4.13 Quenching analysis

The non-linear quenching behavior revealed in Figure S15 suggests that it does not follow a typical Stern-Volmer relationship. Instead, it should be analyzed using a Langmuir adsorption kinetic model. According to a proposed inner-sphere electron transfer (ISET) mechanism, we postulated that alkyl iodine first coordinated to the MLCT excited copper center to form exciplex $\mathrm{Q}Cu^*$ via halide bonding and then electron transfer occurred in the exciplex.

$$\text{Quencher (aq)} + \text{Excited } Cu^* \text{ site (aq)} \leftrightarrow \mathrm{Q}Cu^* \text{ (aq)}$$

$$\theta = \frac{K[Q]}{1+K[Q]} \qquad (1)$$

where θ is the equilibrium site occupancy, $[Q]$ is the concentration of Alkyl iodine in

solution, and K is the equilibrium constant.

From this model, a non-linear equation of the quenching ratio is obtained.

$$\frac{I_0}{I} = \frac{\tau_0}{\tau} = 1 + C\theta = 1 + C\frac{K[Q]}{1+K[Q]} = (C+1) - \frac{C}{1+K[Q]} \qquad (2)$$

where τ_0 is the initial life time of ^{3}MLCT excited Cu complex in the absence of quencher, τ is life time of ^{3}MLCT excited Cu complex in the presence of quencher, and C is as the modified inner-sphere quenching rate constant.

As can be seen in Figure S16, Experimental data can be good fitted using Eq.2.

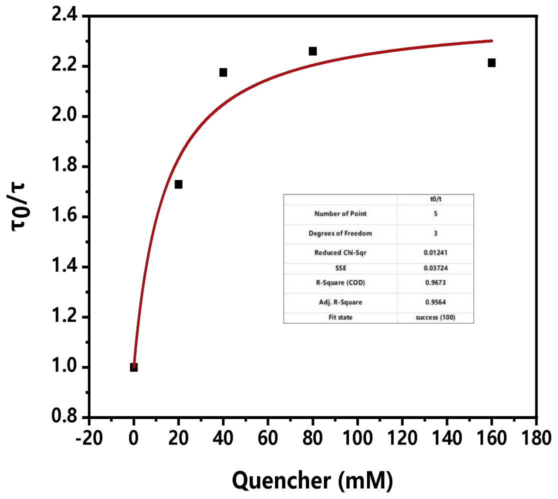

`

Supplementary Figure 16. Lifetime quenching as function of the concentration of quencher and data fitting using a Langmuir adsorption kinetic model.

4.14 Estimated excited-state oxidation potential of in situ generated Cu-1 complex

Cyclic voltammograms were recorded using a CH Instruments 656E potentiostat, a glassy carbon working electrode, a Ag/AgCl reference electrode, and a Pt counter electrode. The voltammograms were recorded at room temperature under nitrogen atmosphere in 0.1 M Bu_4NPF_6 in MeCN containing in situ generated **Cu-1** (1 mM), All potentials are reported in V vs SCE. The $E_{1/2}$ of the Fc/Fc^+ redox couple used as a standard is 0.42 V vs SCE in MeCN.

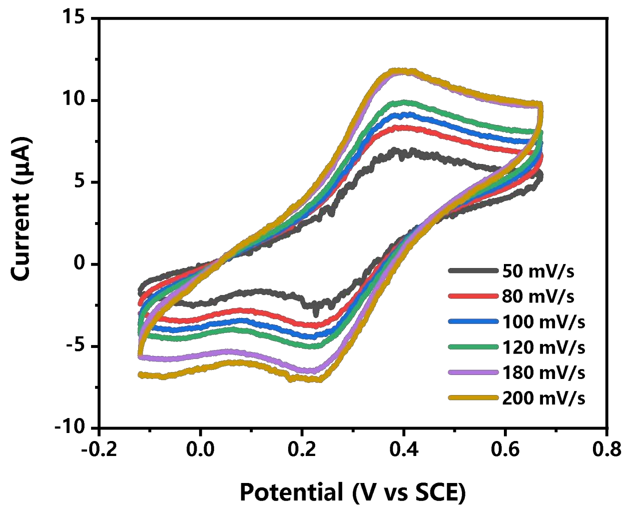

Supplementary Figure 17. Cyclic voltammogram of in situ generated **Cu-1** with scan rates ranging from 50 mV/s to 200 mV/s

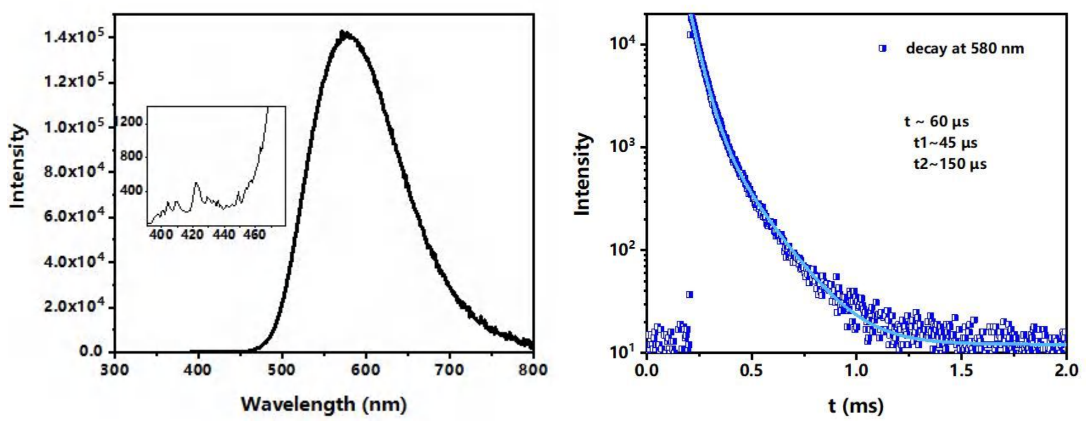

Supplementary Figure 18. Steady state emission of in situ generated **Cu-1** in MeCN at 80 K (excitation at 375 nm) (left) and Long-lived luminescence decay for in-situ generated Cu-1 complex measure at 80 K (excitation at 375 nm) (right)

Discussion: The λ_{tail} of emission of in situ generated **Cu-1** complex is ca.500 nm, together with the ground-state potential ($E^0(Cu^{II/I})$ = 0.29 V (vs SCE)), excited state potential is estimated to be $E^*(Cu^{II/I*}) = –2.48 + 0.29 = -2.19$ V (vs SCE).

4.15 The exploration of interactions between in-situ generated Cu-1 complex with alkyl iodides on ground state with ^{1}H NMR and UV-vis spectra

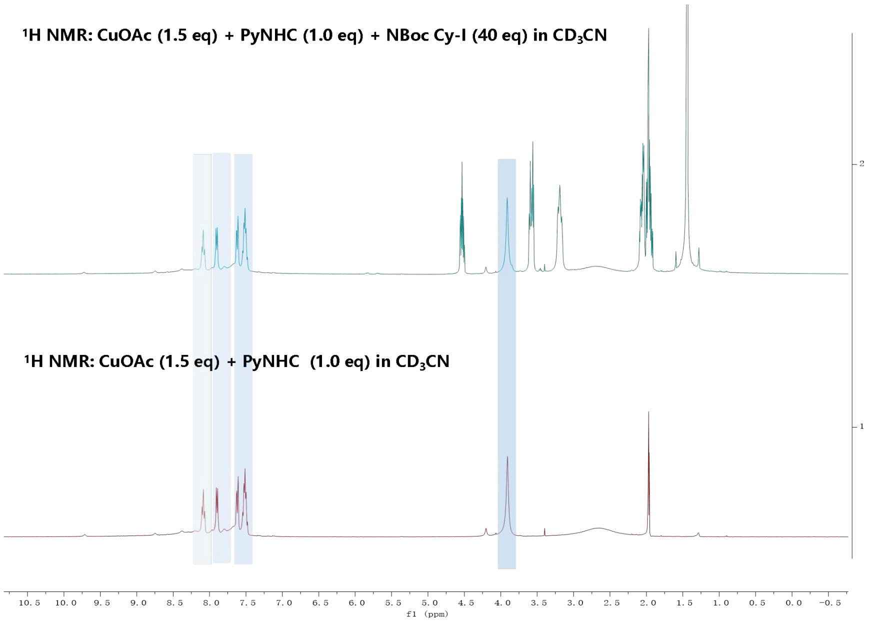

Supplementary Figure 19. ^{1}H NMR spectra of in situ generated **Cu-1** complex with/without alkyl iodide in CD_3CN

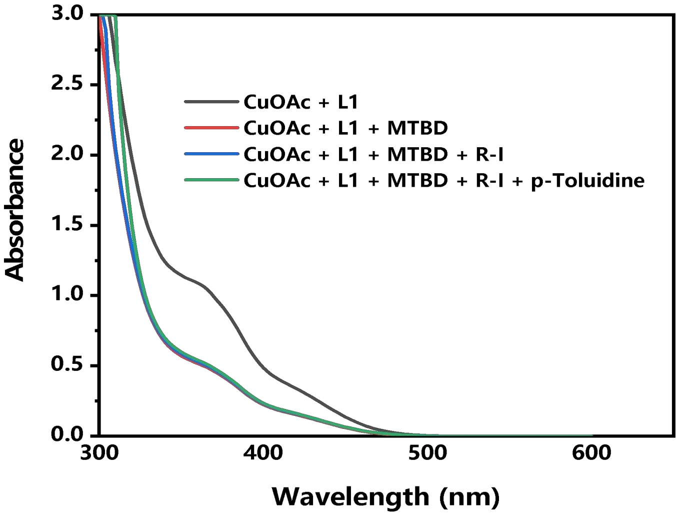

Supplementary Figure 20. UV-vis spectra of copper complexes

Discussion: The UV-vis and ^{1}H NMR spectra did not give any information about the association with alkyl iodides with in-situ generated copper complexes on ground state.

E. DFT calculation

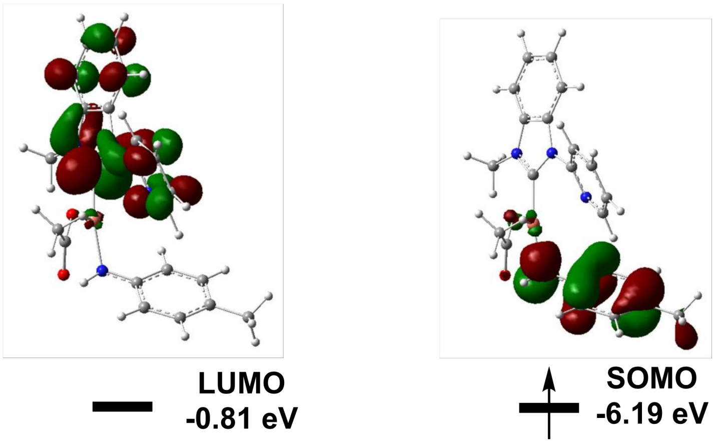

Supplementary Figure 21. The lowest unoccupied molecular orbitals and singly occupied molecular orbitals of Cu^{II}(Py-NHC)(OAc)(NH-*p*-Tol), with isovalues of 0.04 a.u.

4.16 Computational details

The optimizations were carried out using the Gaussian 16 software package[17], with the structures optimized at the B3LYP-D3(BJ) level of density functional theory (DFT) and a def2-TZVP basis set employed for all atoms. (For more detail, see supplementary data source)

F Other attempted couplings

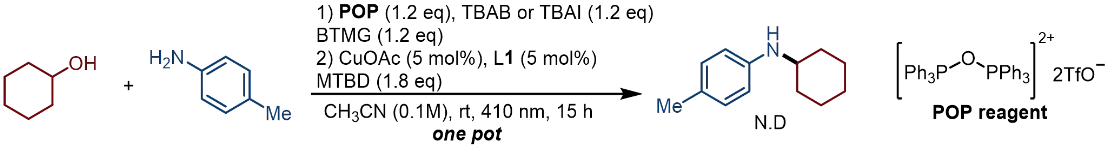

Supplementary Figure 22. Direct amination of alcohol in one-pot procedure.

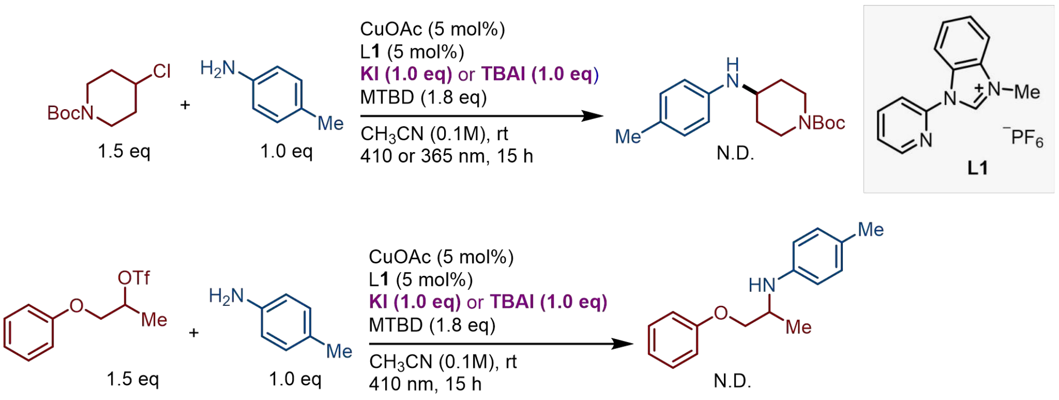

Supplementary Figure 23. Amination of alkyl chlorides/triflate in the presence of KI, TBAI

.

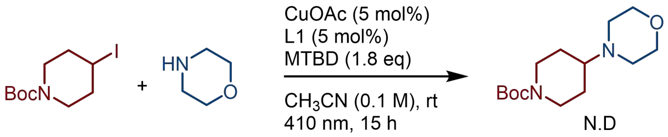

Supplementary Figure 24. Secondary amine as N-nucleophiles

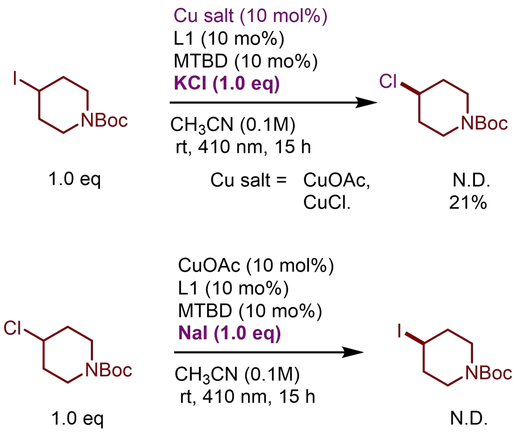

Supplementary Figure 25. halogen exchange reaction

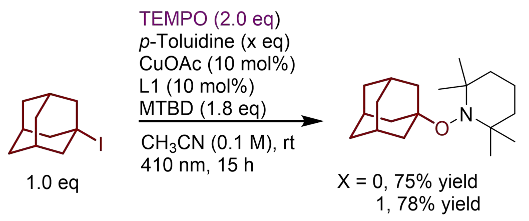

Supplementary Figure 26. Teritary alkyl radical was captured by TEMPO

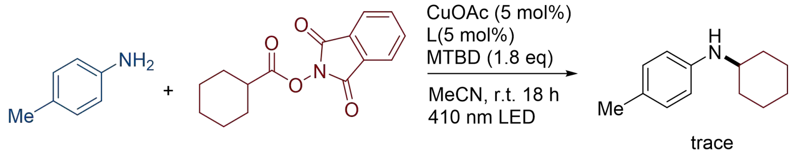

Supplementary Figure 27. *N*-Hydroxyphthalimide Esters as alkyl radical precursor

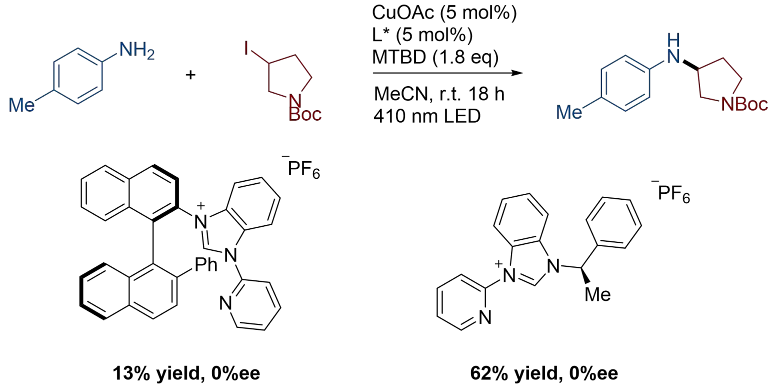

Supplementary Figure 28. Asymmetric copper catalyzed C-N couplings was attempted used chiral PyNHC ligands

5. Copies of NMR Spectra

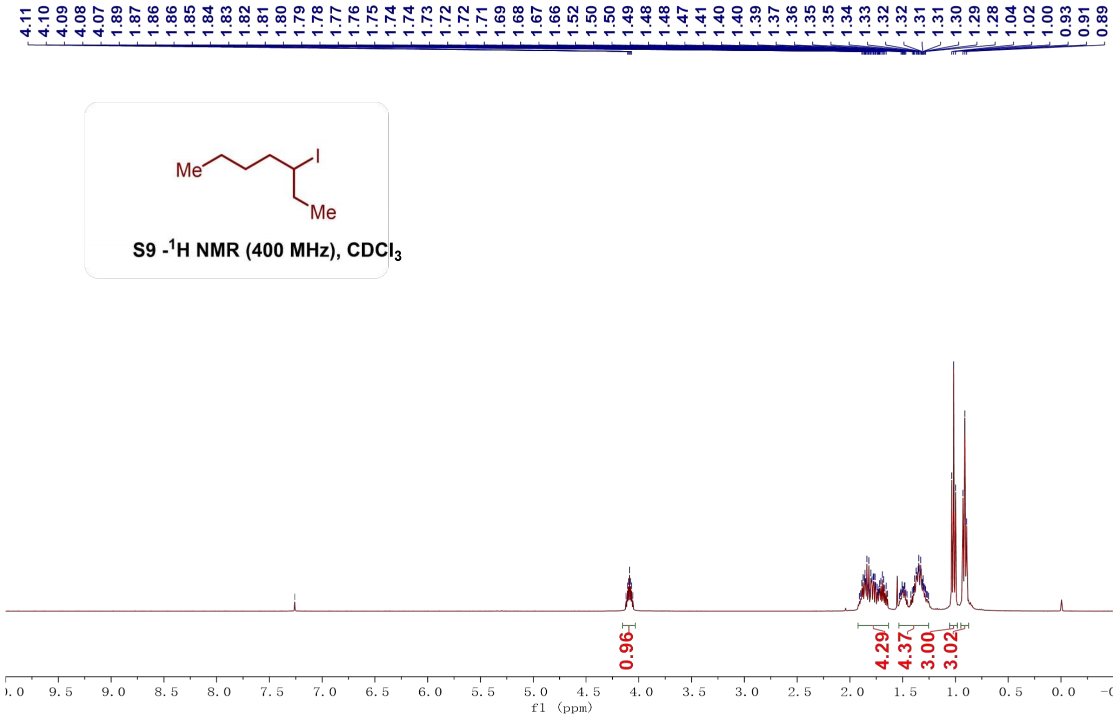

Supplementary Figure 29. ^{1}H NMR (400 MHz, $CDCl_3$) spectrum of compound **S9**

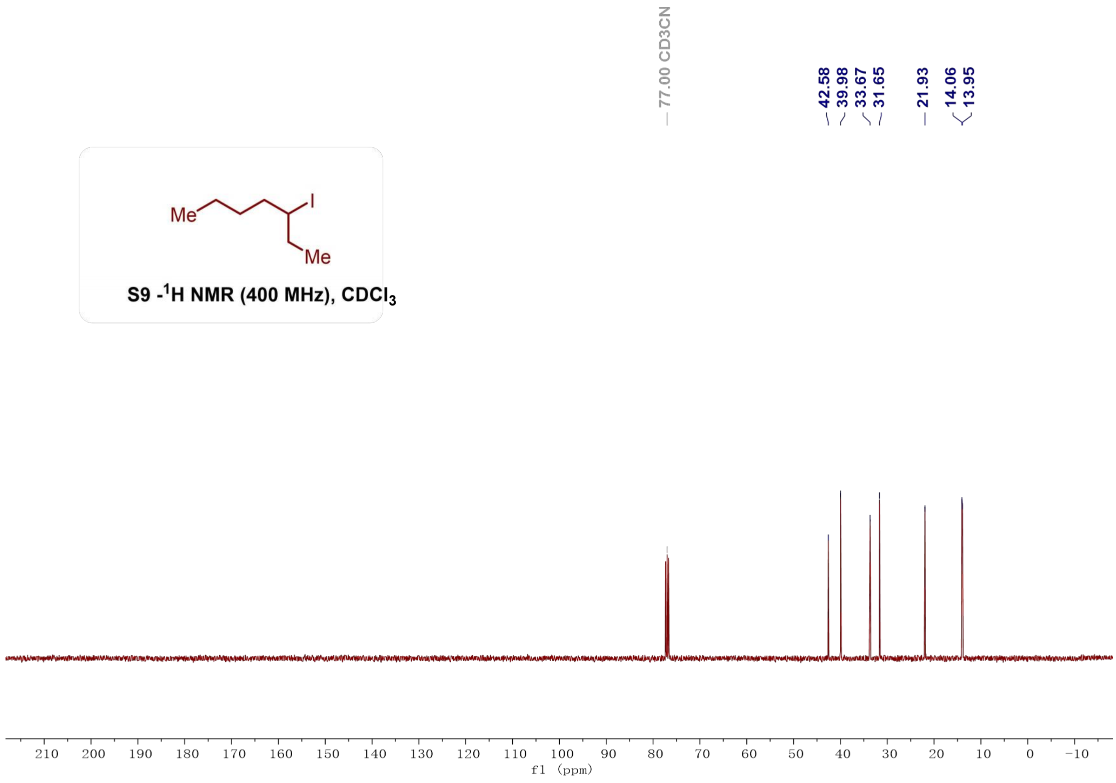

Supplementary Figure 30. ^{13}C NMR (101 MHz, $CDCl_3$) spectrum of compound **S9**

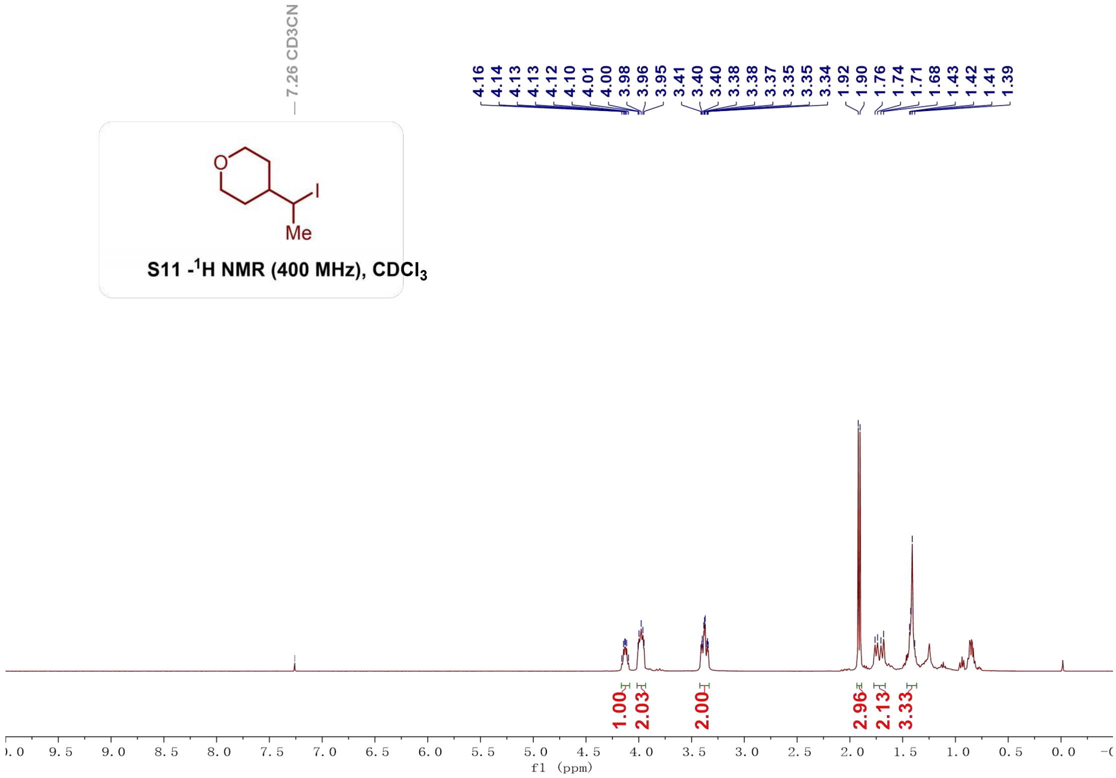

Supplementary Figure 31. ^{1}H NMR (400 MHz, $CDCl_3$) spectrum of compound **S11**

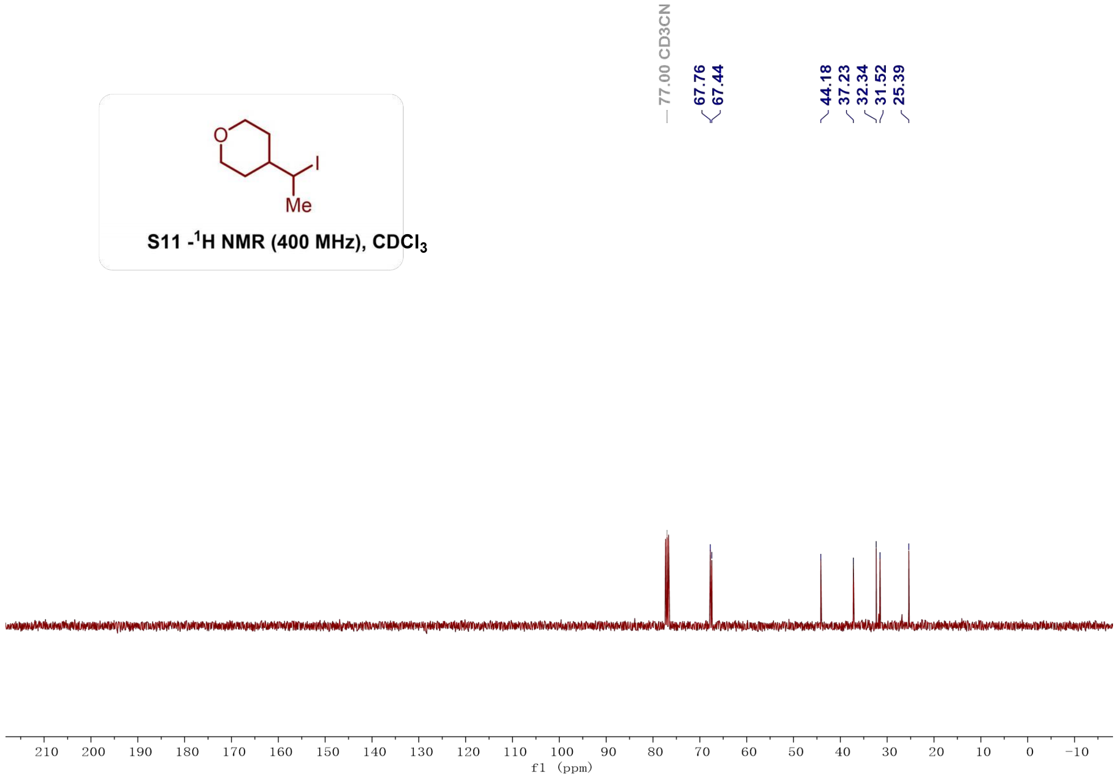

Supplementary Figure 32. ^{13}C NMR (101 MHz, $CDCl_3$) spectrum of compound **S11**

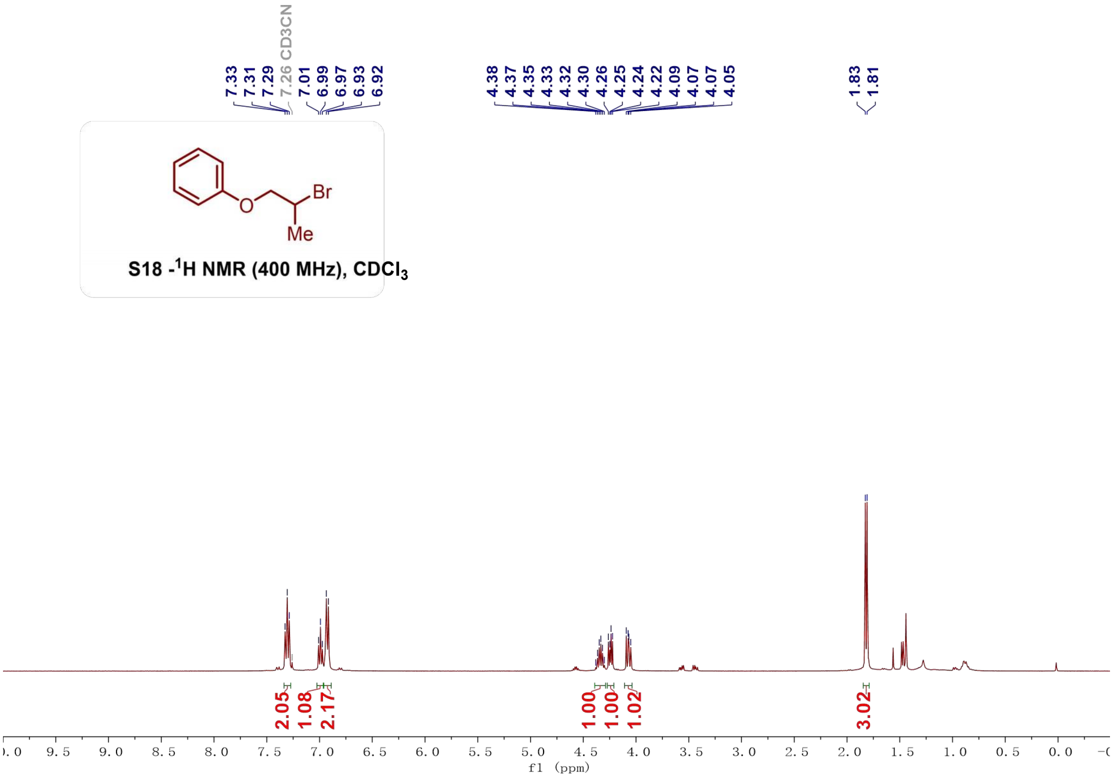

Supplementary Figure 33. ^{1}H NMR (400 MHz, $CDCl_3$) spectrum of compound **S18**

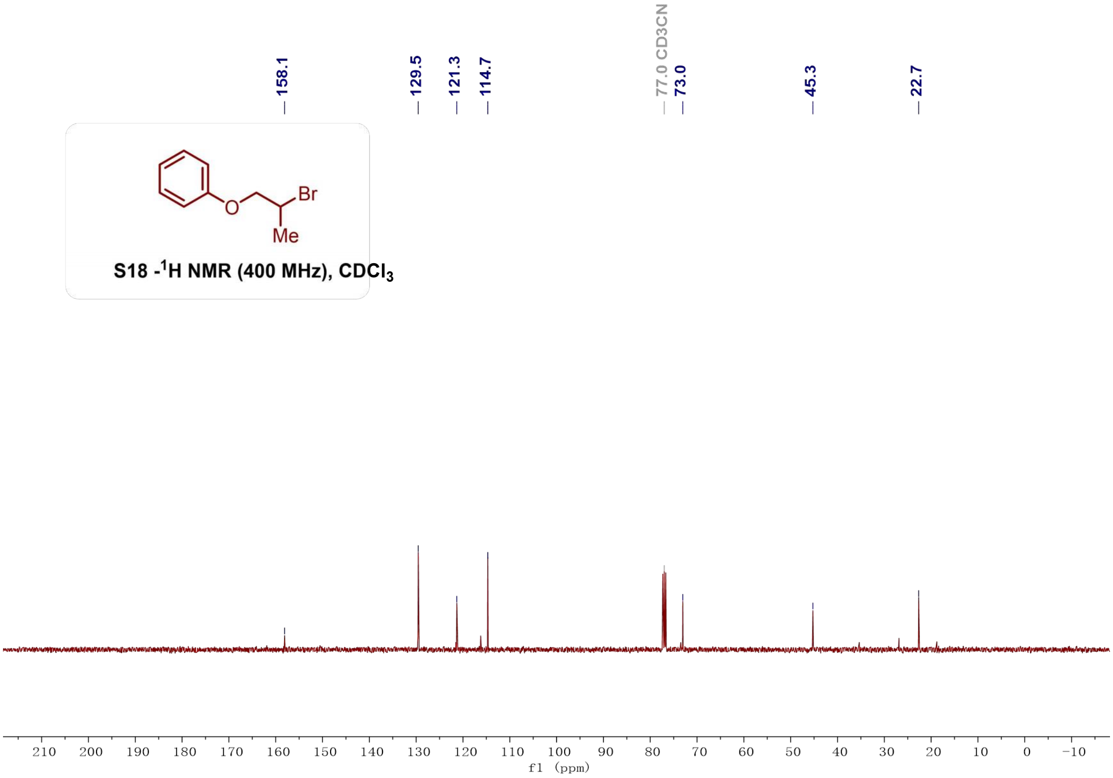

Supplementary Figure 34. ^{13}C NMR (101 MHz, $CDCl_3$) spectrum of compound **S18**

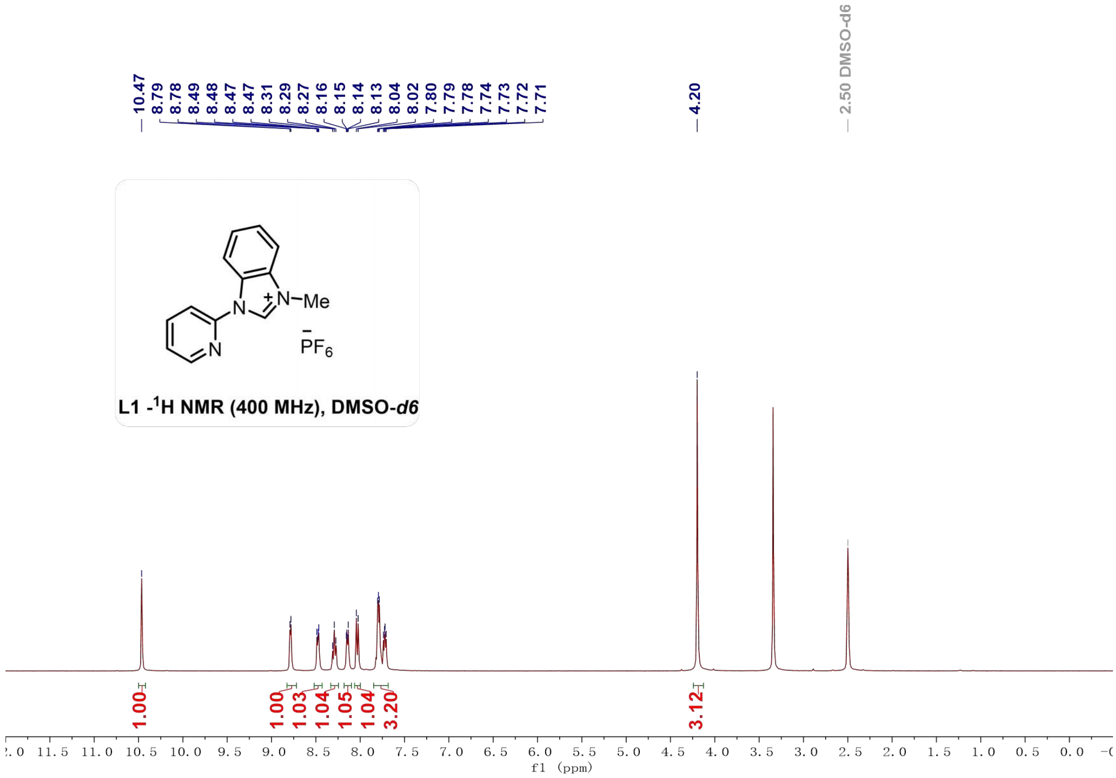

Supplementary Figure 35. ^{1}H NMR (400 MHz, DMSO-*d6*) spectrum of compound **L1**

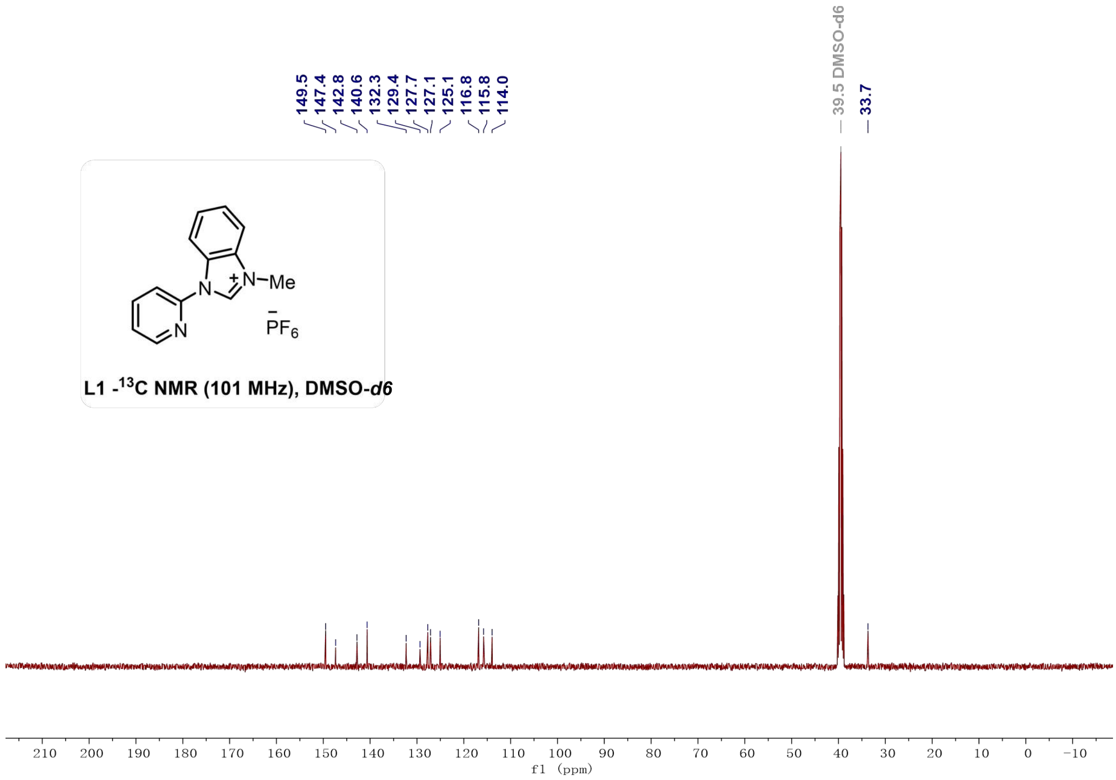

Supplementary Figure 36. ^{13}C NMR (101 MHz, DMSO-*d6*) spectrum of compound **L1**

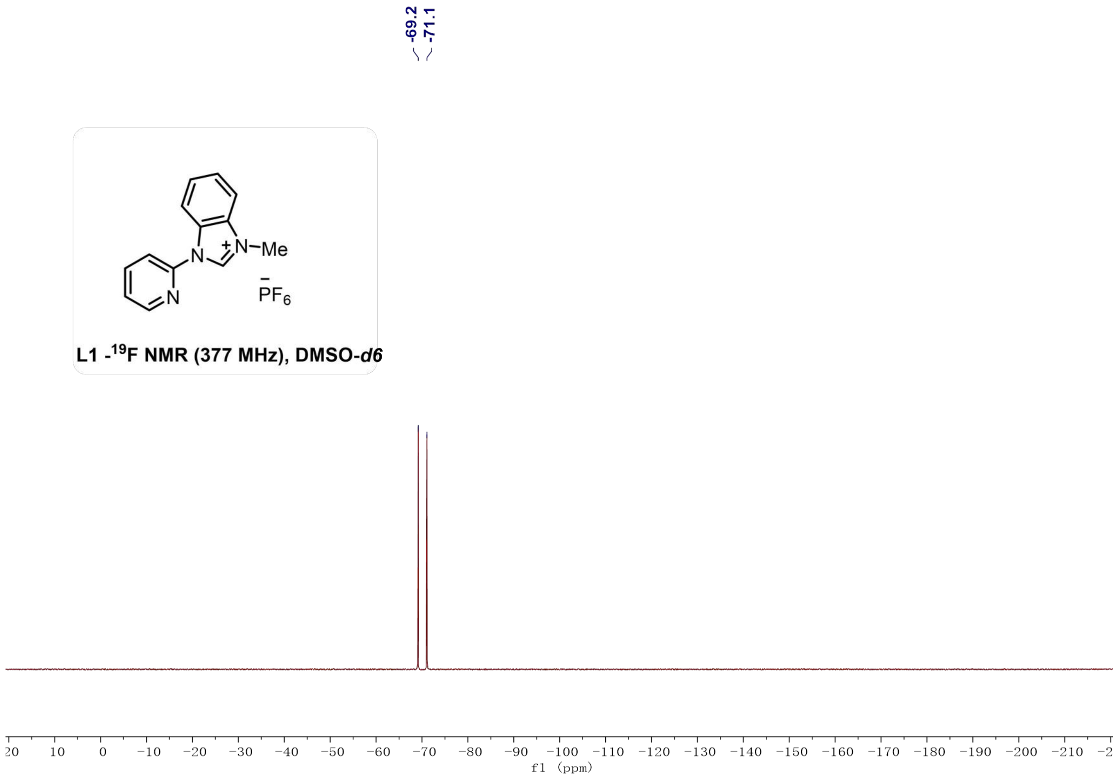

Supplementary Figure 37. ^{19}F NMR (377 MHz, DMSO-d_6) spectrum of compound **L1**

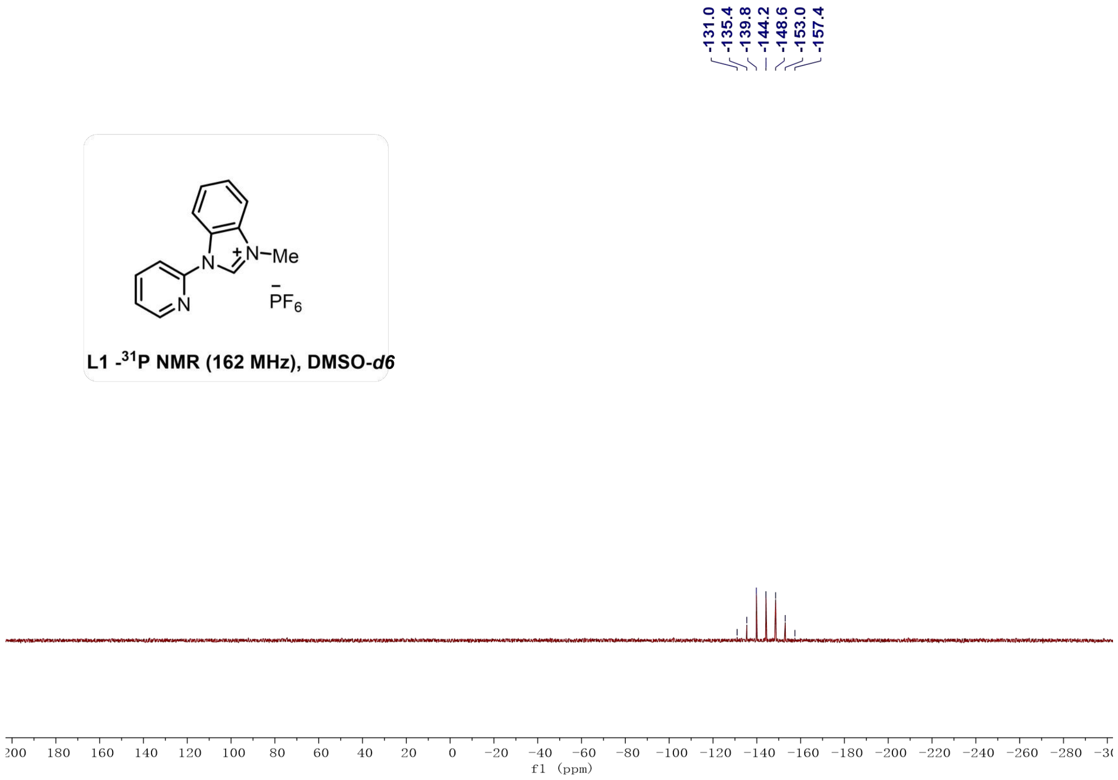

Supplementary Figure 38. ^{31}P NMR (162 MHz, DMSO-d_6) spectrum of compound **L1**

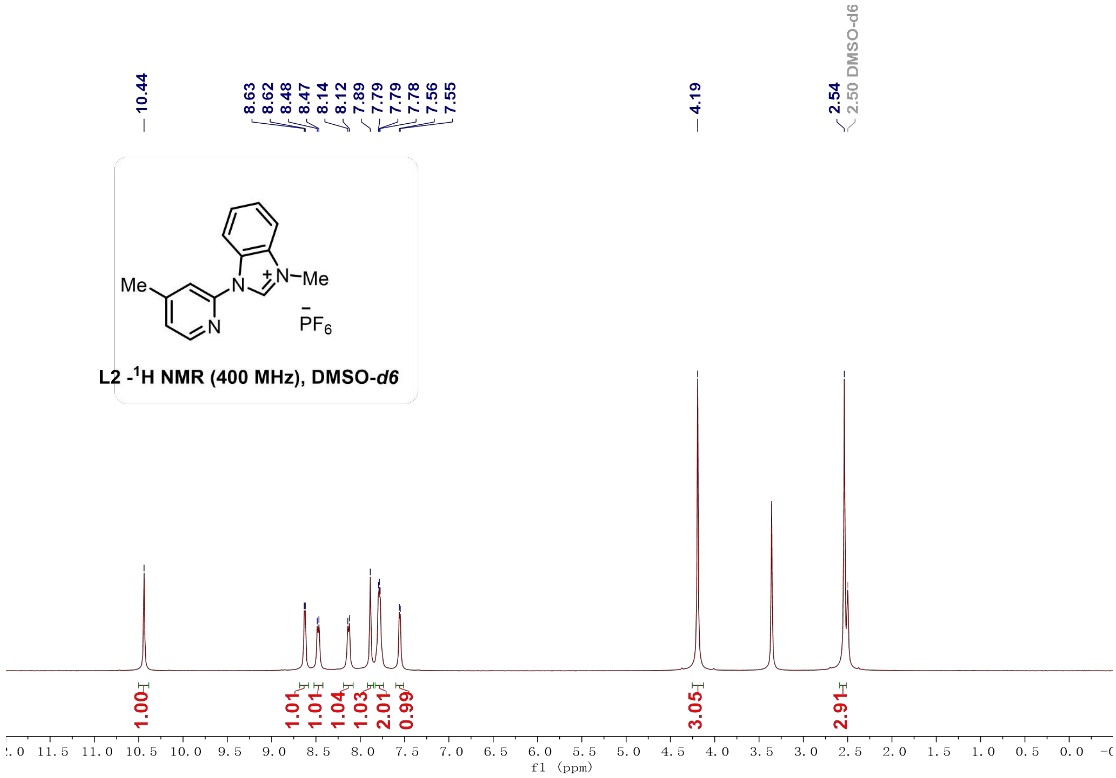

Supplementary Figure 39. ^{1}H NMR (400 MHz, DMSO-*d*6) spectrum of compound **L2**

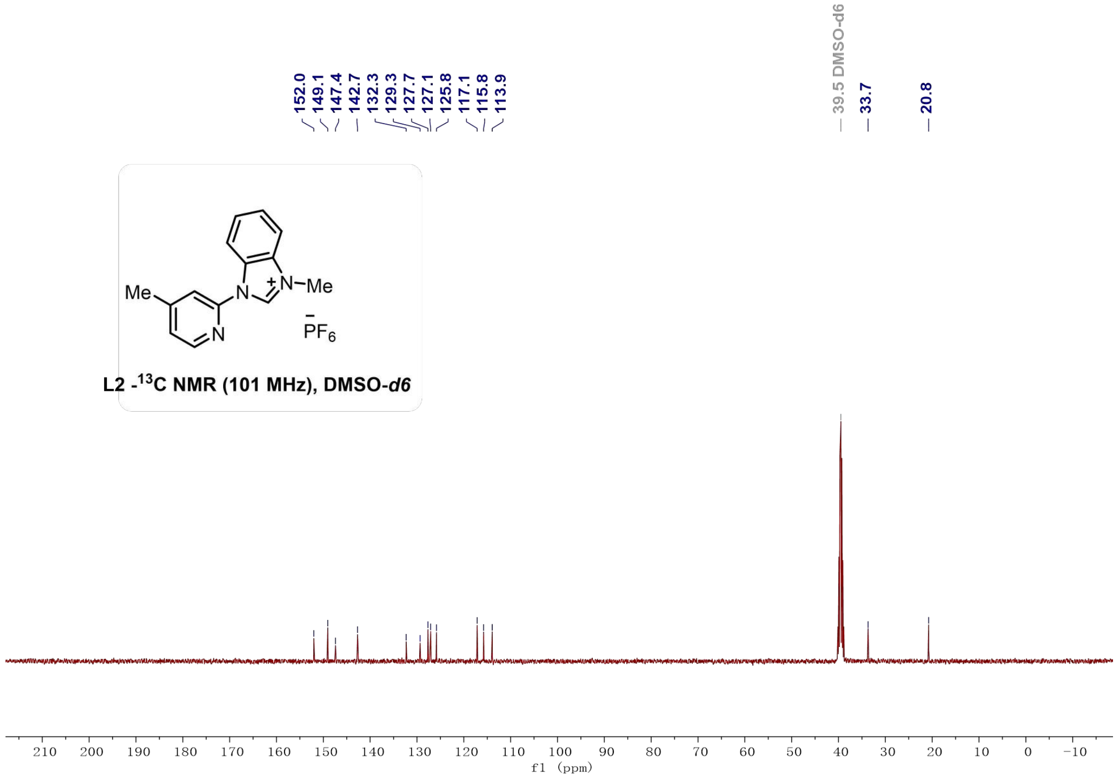

Supplementary Figure 40. ^{13}C NMR (101 MHz, DMSO-*d*6) spectrum of compound **L2**

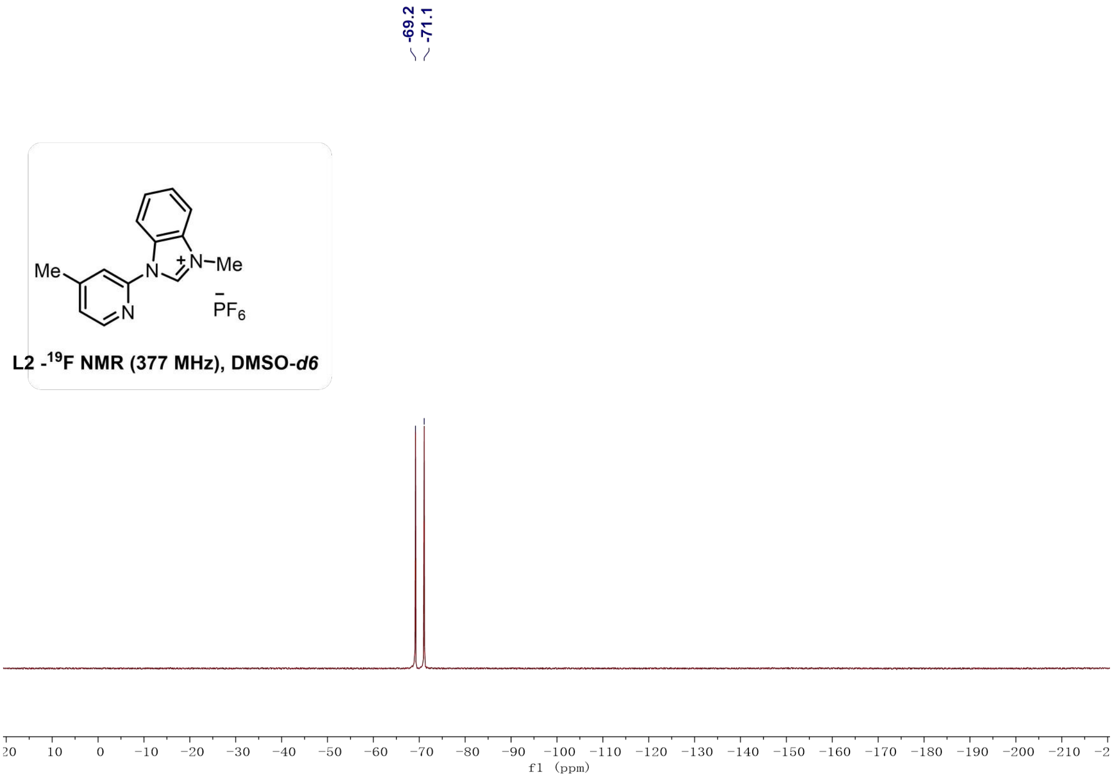

Supplementary Figure 41. ^{19}F NMR (377 MHz, DMSO-d_6) spectrum of compound **L2**

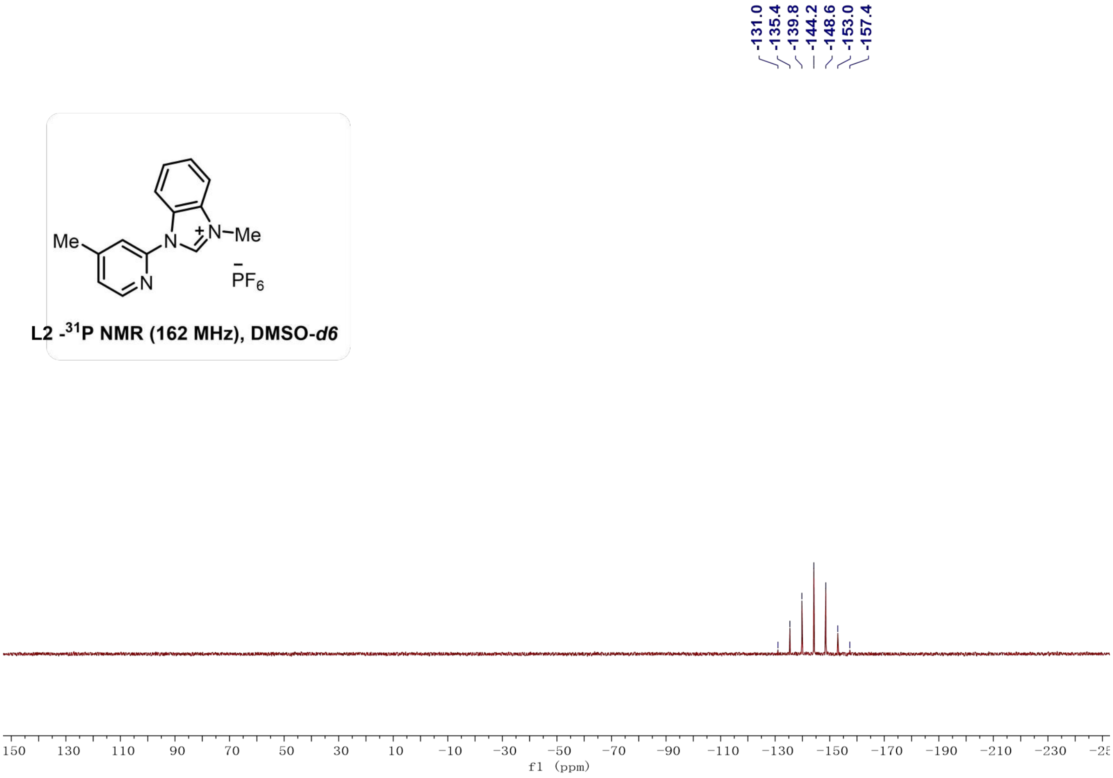

Supplementary Figure 42. ^{31}P NMR (162 MHz, DMSO-d_6) spectrum of compound **L2**

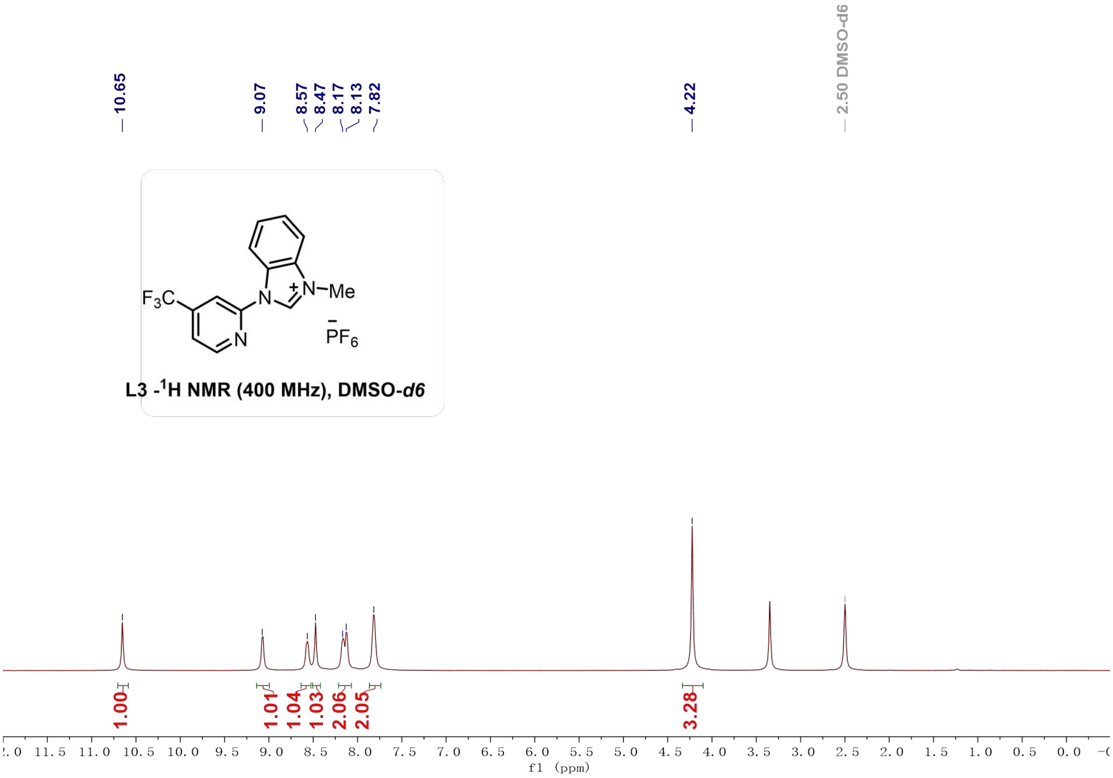

Supplementary Figure 43. ^{1}H NMR (400 MHz, DMSO-*d*6) spectrum of compound **L3**

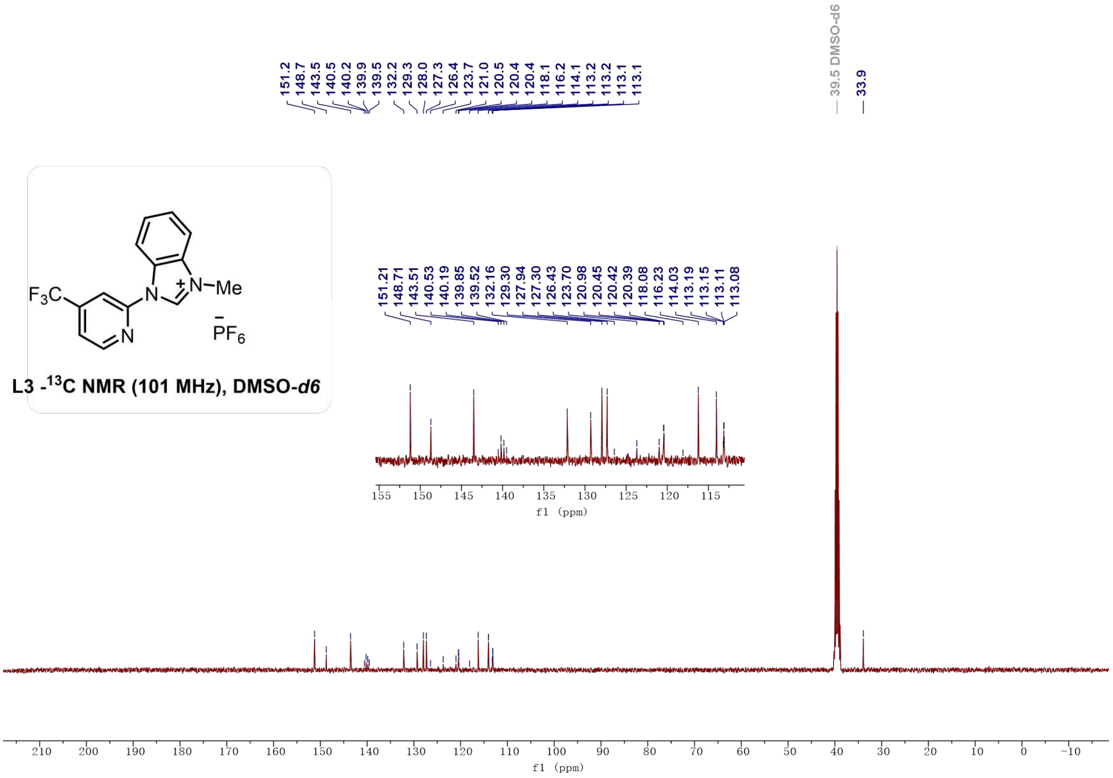

Supplementary Figure 44. ^{13}C NMR (101 MHz, DMSO-*d*6) spectrum of compound **L3**

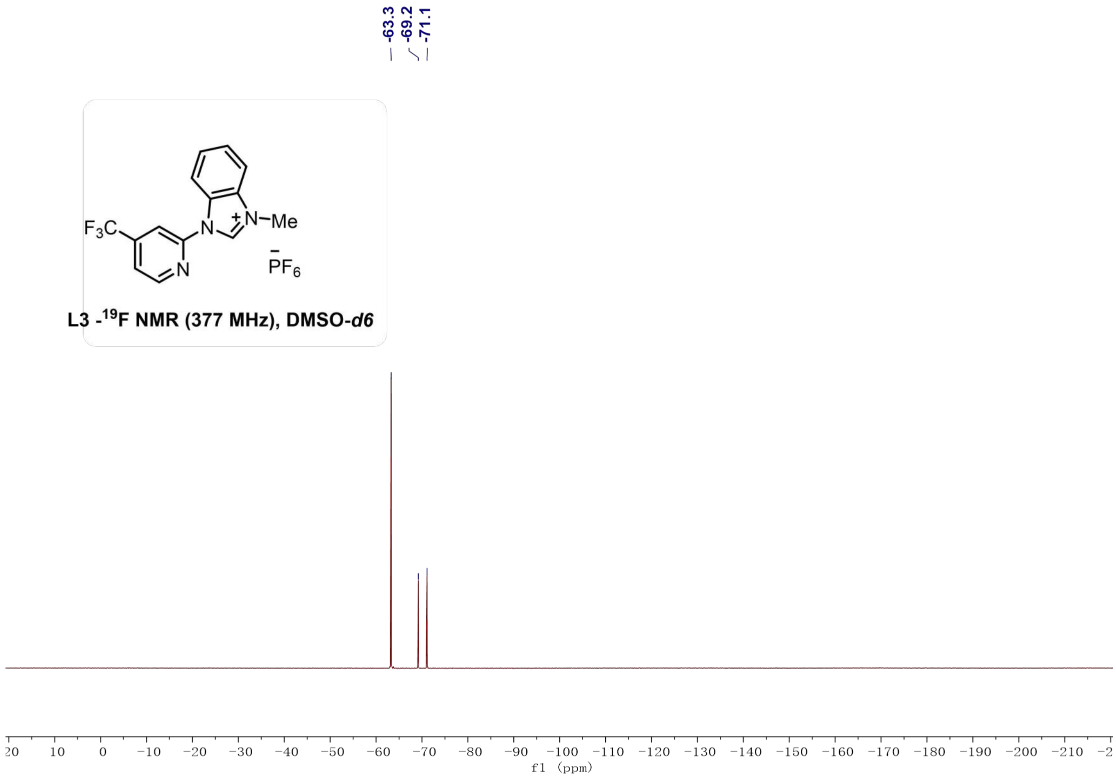

Supplementary Figure 45. ^{19}F NMR (377 MHz, DMSO-d_6) spectrum of compound **L3**

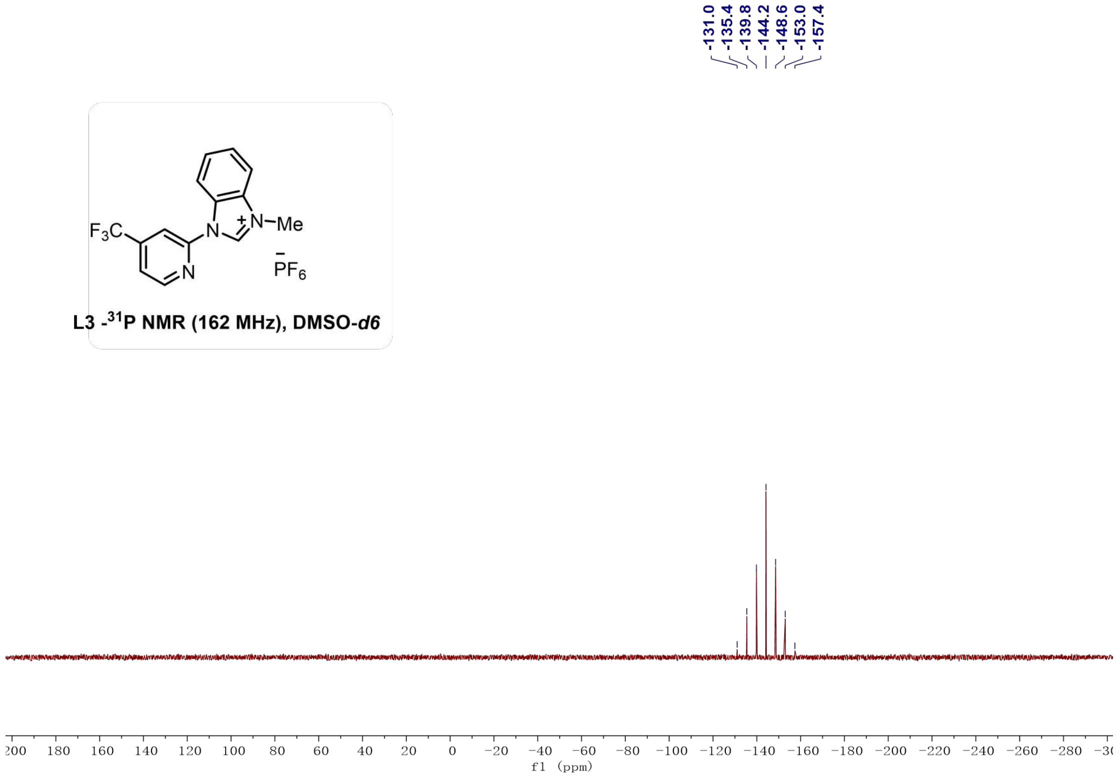

Supplementary Figure 46. ^{31}P NMR (162 MHz, DMSO-d_6) spectrum of compound **L3**

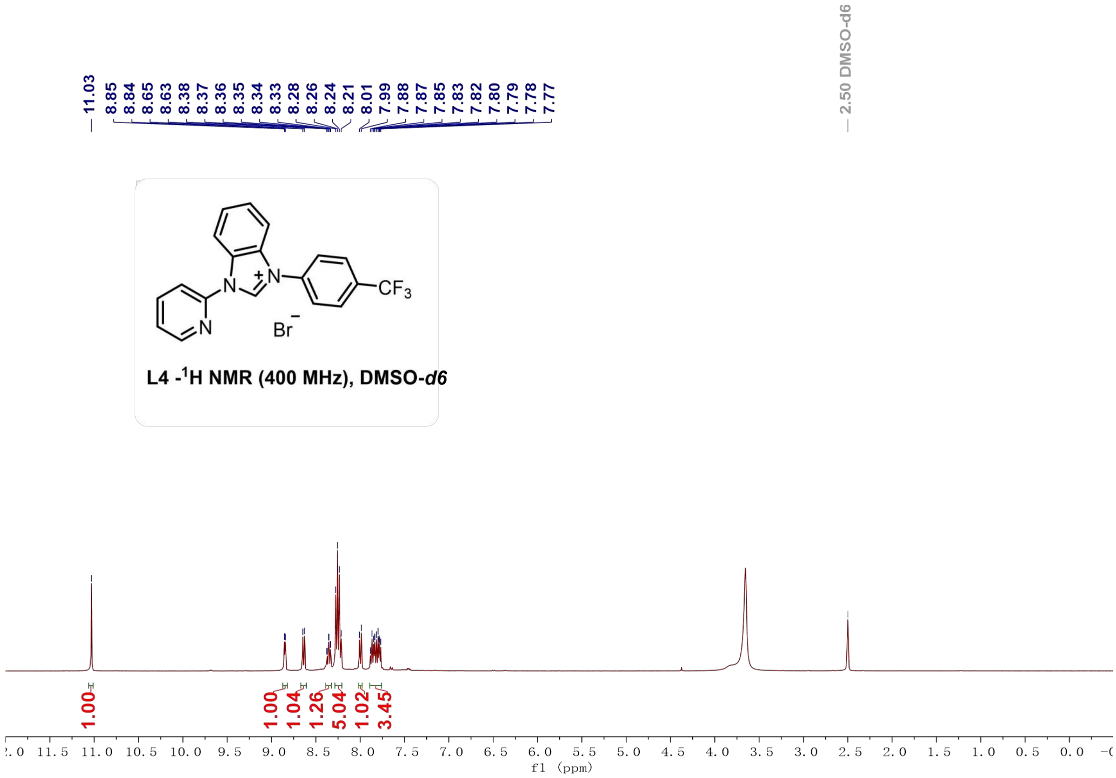

Supplementary Figure 47. ^{1}H NMR (400 MHz, DMSO-*d6*) spectrum of compound **L4**

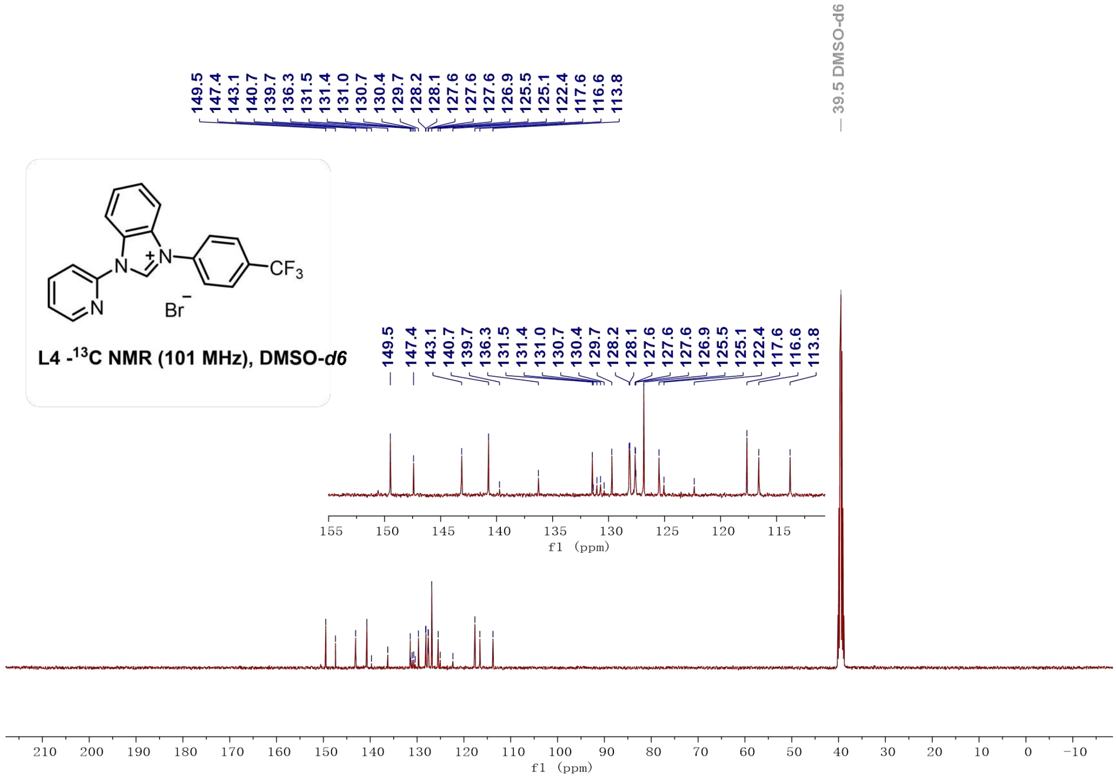

Supplementary Figure 48. ^{13}C NMR (101 MHz, DMSO-*d6*) spectrum of compound **L4**

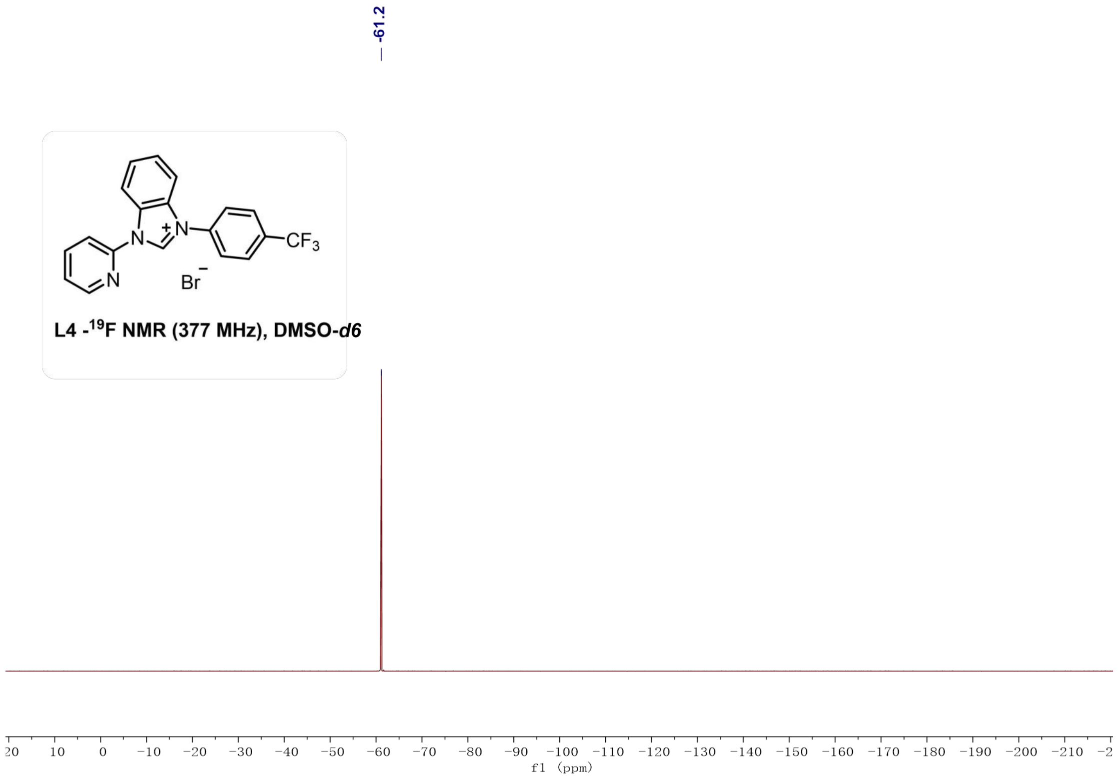

Supplementary Figure 49. ^{19}F NMR (377 MHz, DMSO-d_6) spectrum of compound **L4**

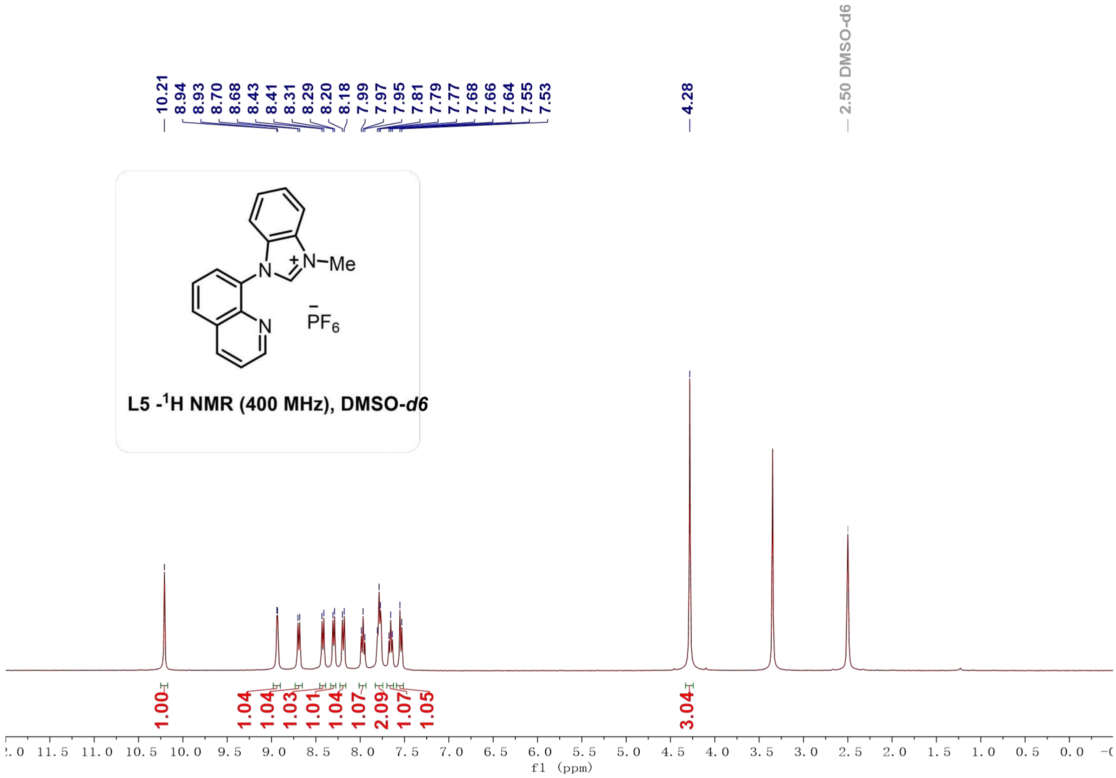

Supplementary Figure 50. ^{1}H NMR(400 MHz, DMSO-*d*6) spectrum of compound **L5**

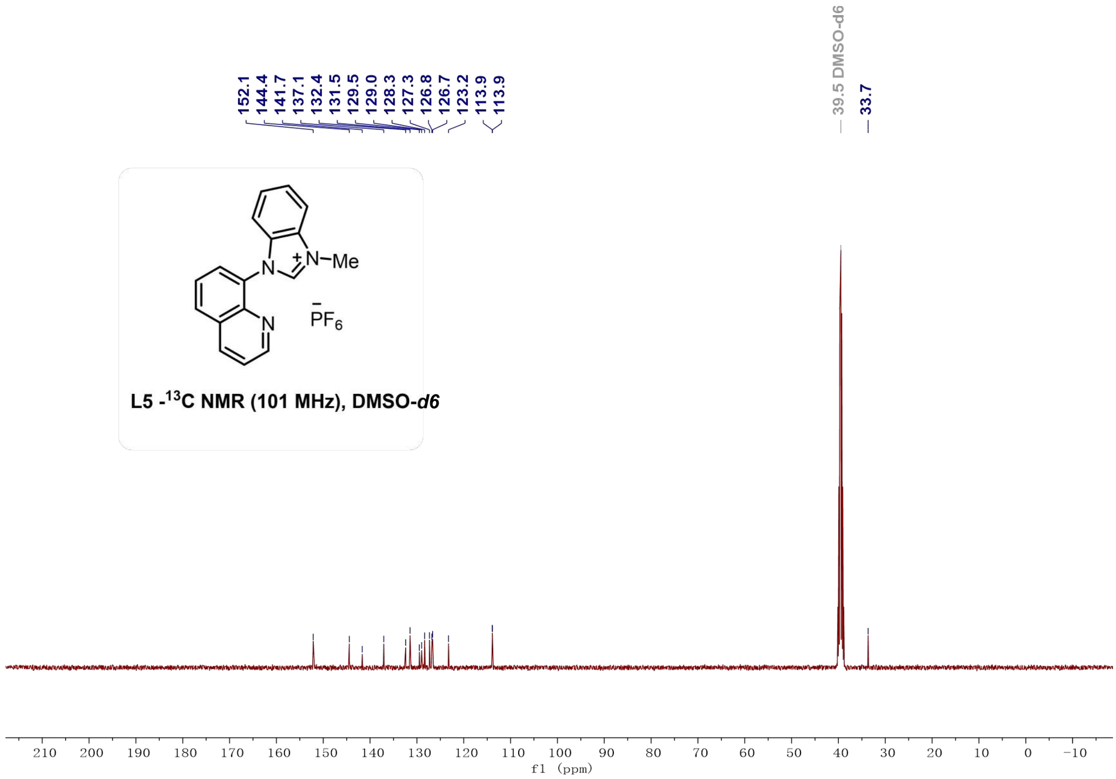

Supplementary Figure 51. ^{13}C NMR (101 MHz, DMSO-*d*6) spectrum of compound **L5**

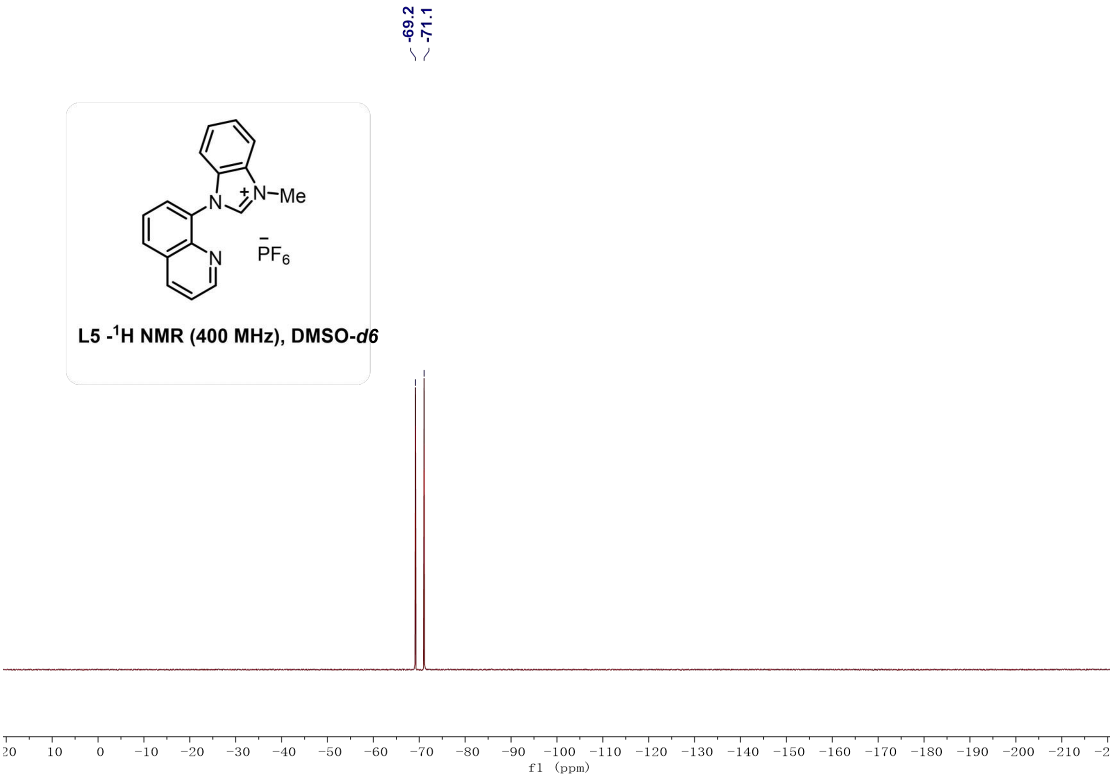

Supplementary Figure 52. ^{19}F NMR (377 MHz, DMSO-d_6) spectrum of compound **L5**

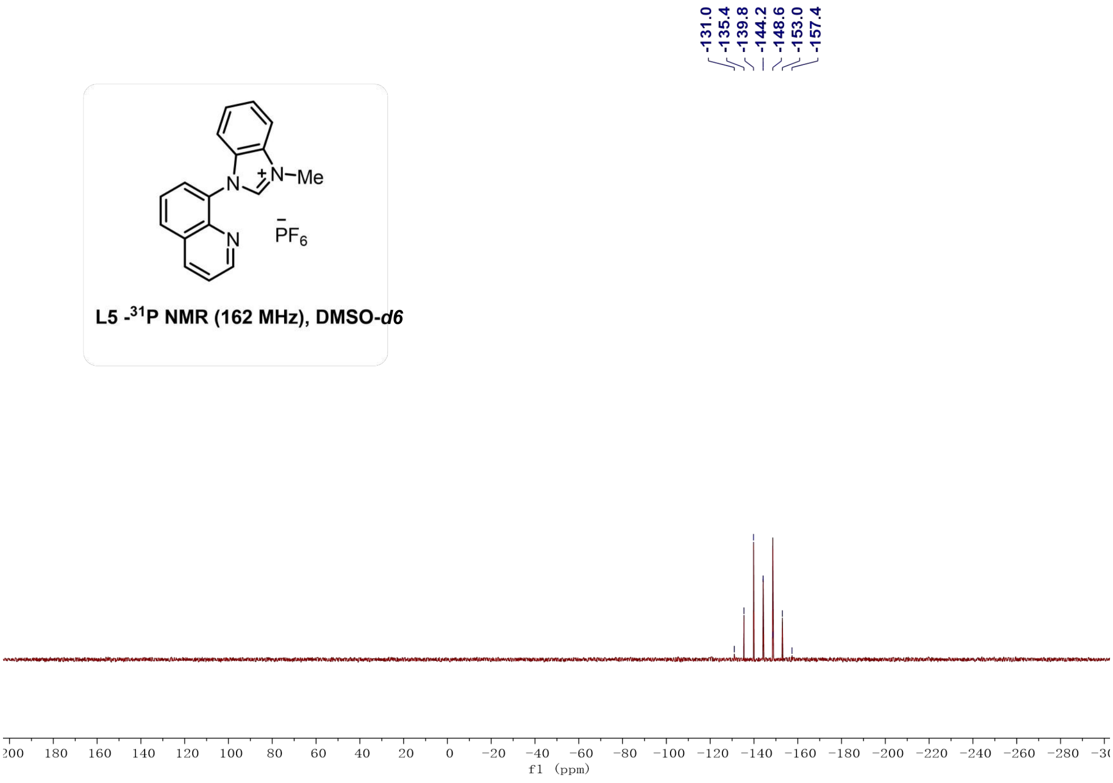

Supplementary Figure 53. ^{31}P NMR (162 MHz, DMSO-d_6) spectrum of compound **L6**

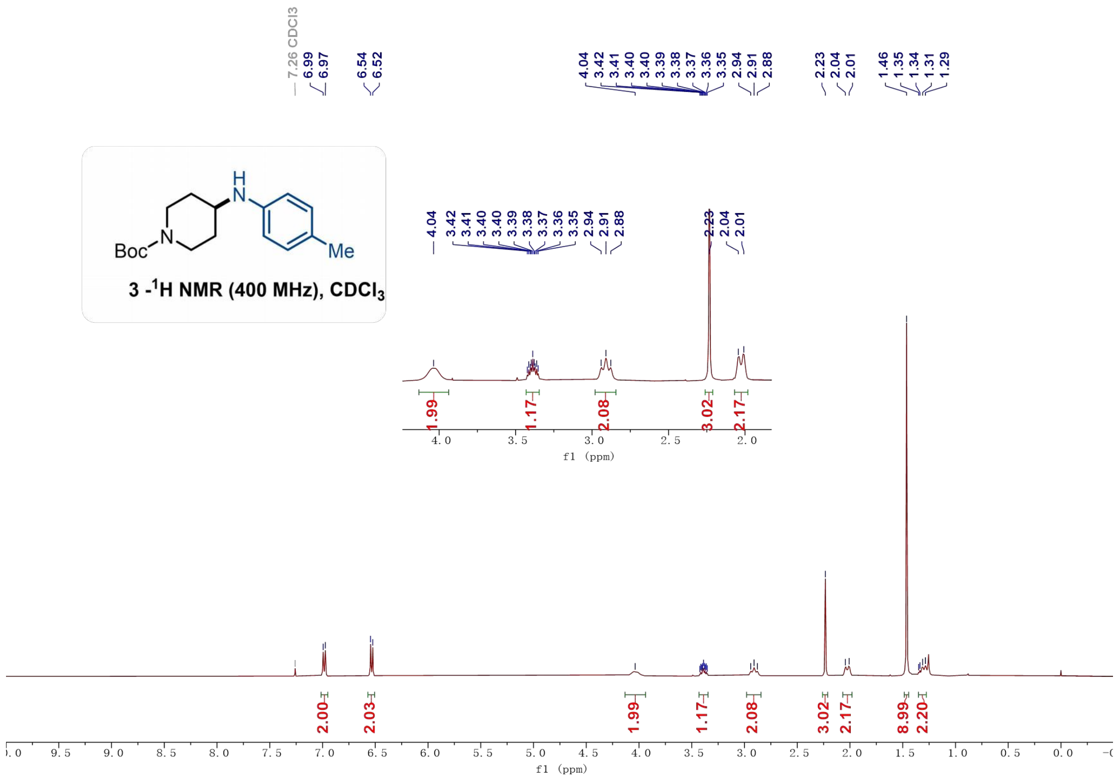

Supplementary Figure 54. ^{1}H NMR (400 MHz, $CDCl_3$) spectrum of compound **3**

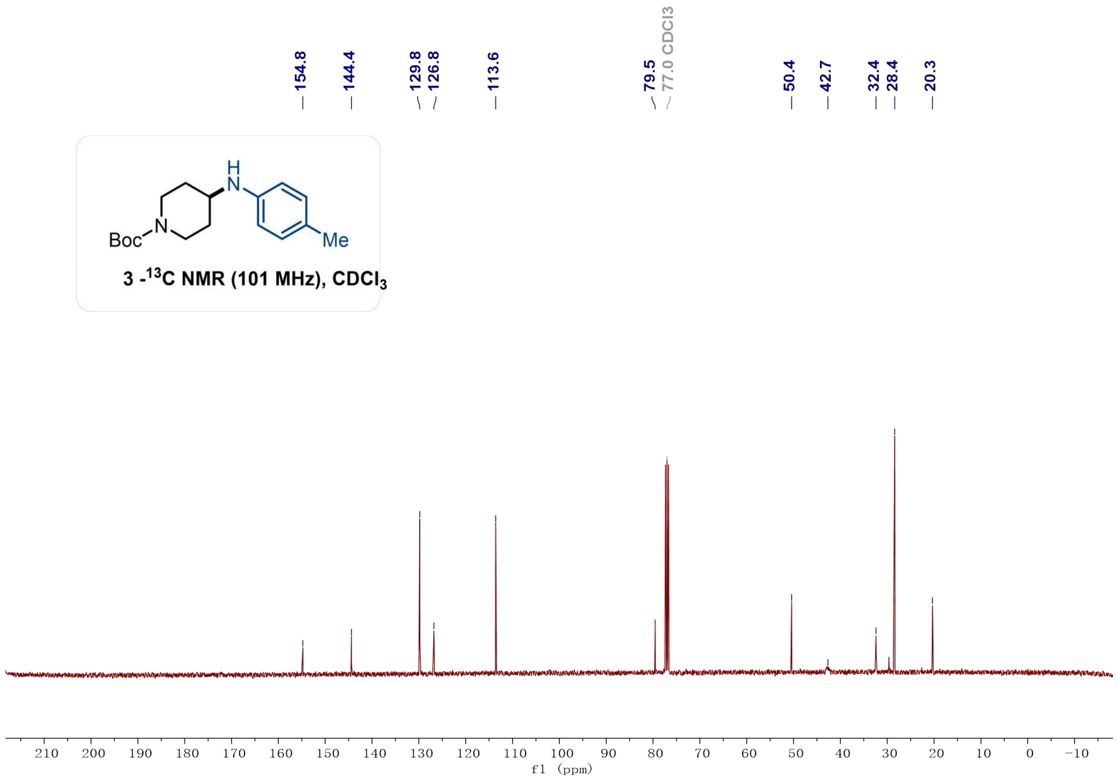

Supplementary Figure 55. ^{13}C NMR (101 MHz, $CDCl_3$) spectrum of compound **3**

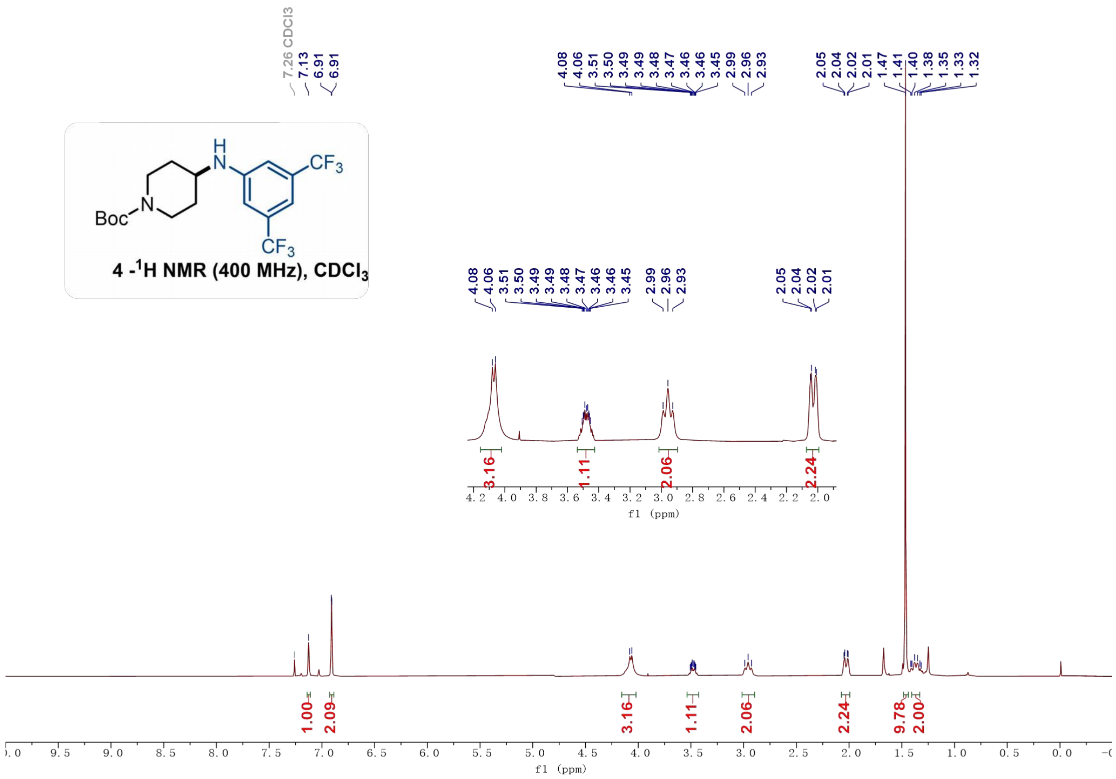

Supplementary Figure 56. ^{1}H NMR (400 MHz, $CDCl_3$) spectrum of compound **4**

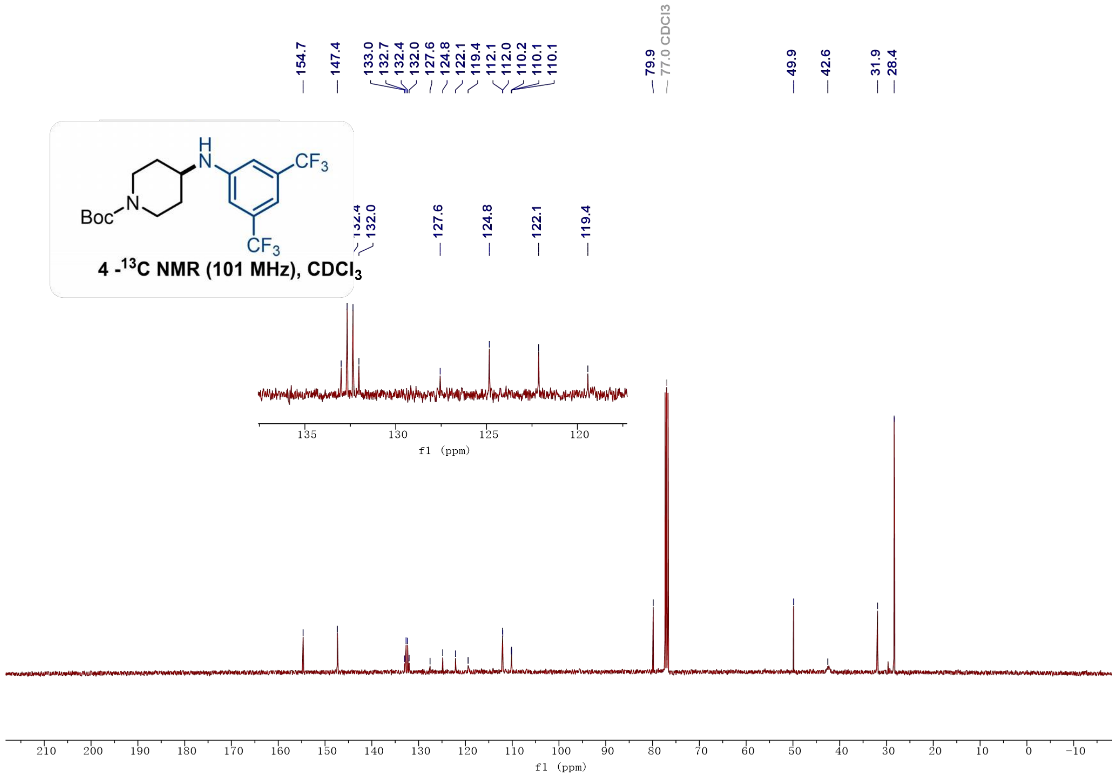

Supplementary Figure 57. ^{13}C NMR (101 MHz, $CDCl_3$) spectrum of compound **4**

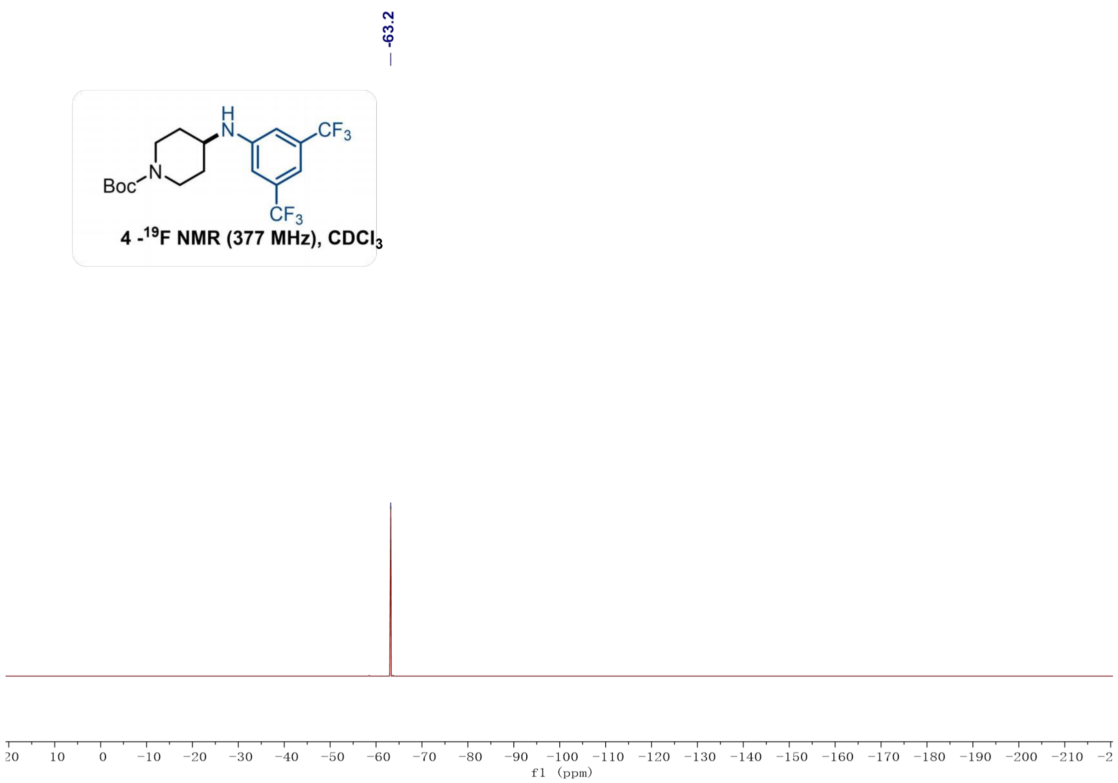

Supplementary Figure 58. ^{19}F NMR (377 MHz, $CDCl_3$) spectrum of compound **4**

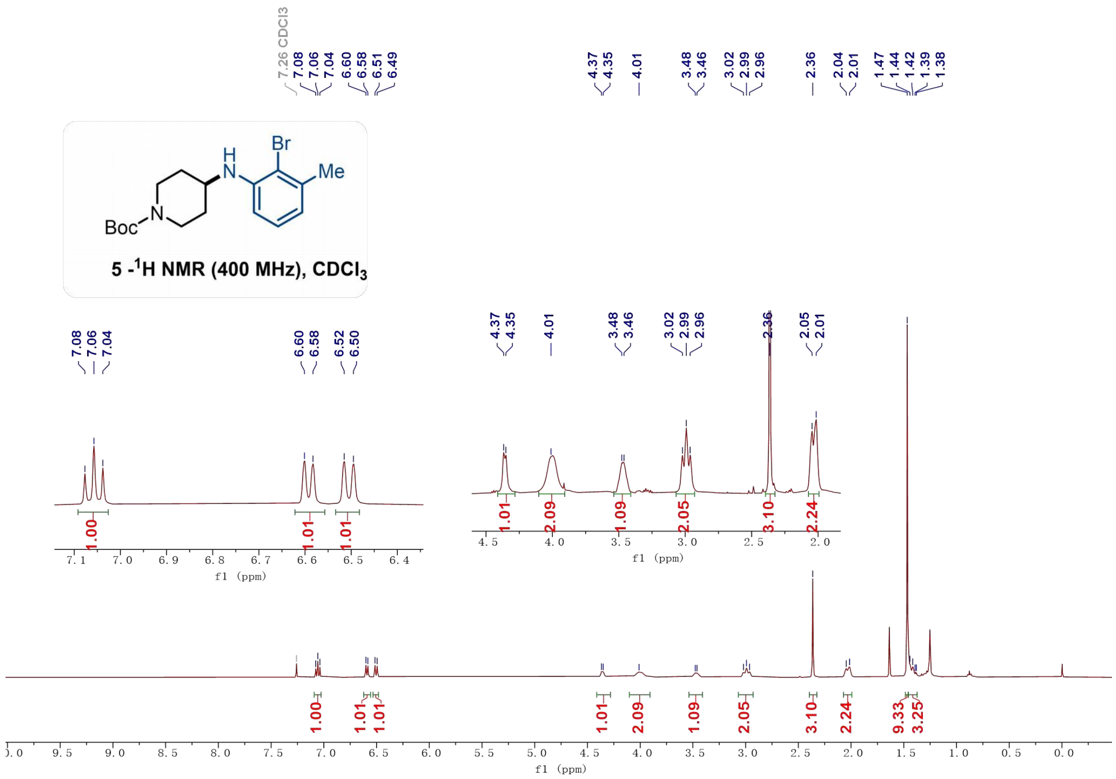

Supplementary Figure 59. ^{1}H NMR (400 MHz, $CDCl_3$) spectrum of compound **5**

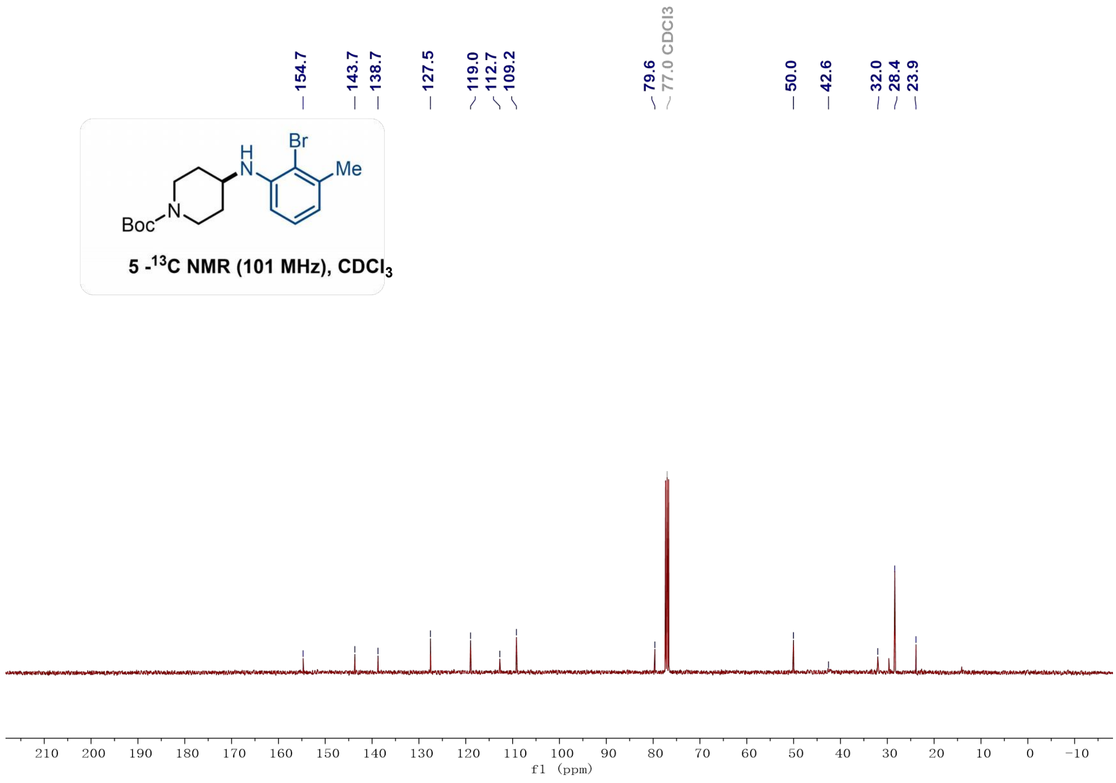

Supplementary Figure 60. ^{13}C NMR (101 MHz, $CDCl_3$) spectrum of compound **5**

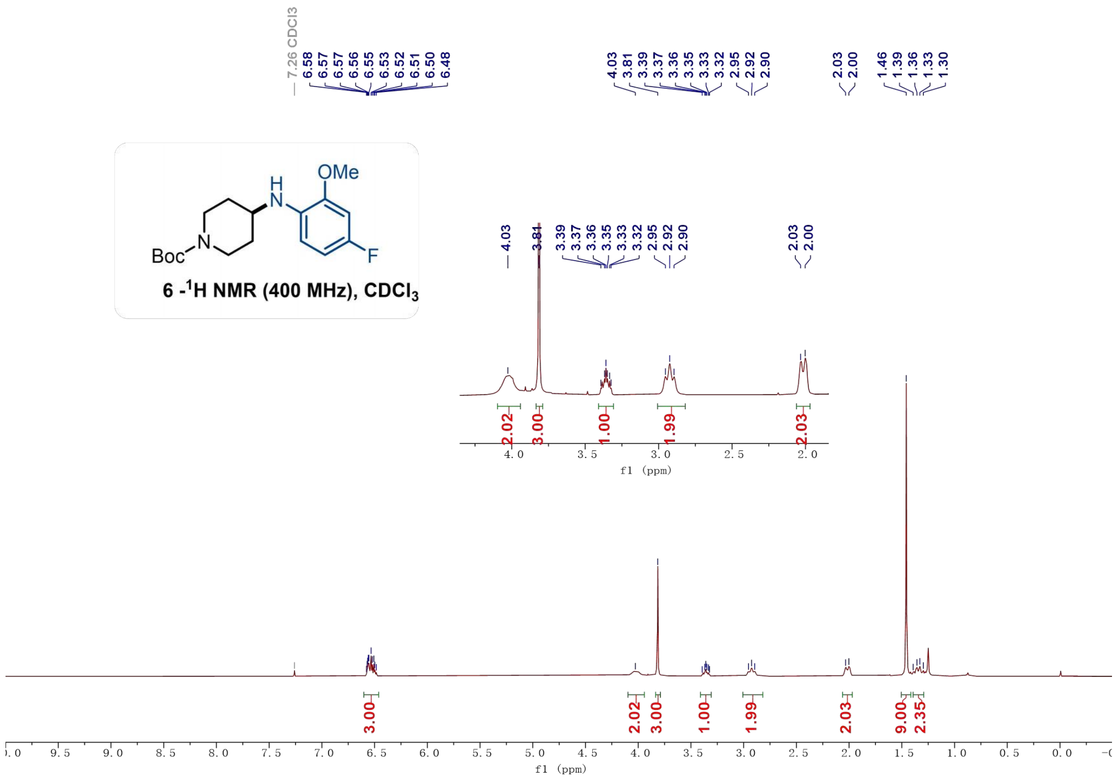

Supplementary Figure 61. ^{1}H NMR (400 MHz, $CDCl_3$) spectrum of compound **6**

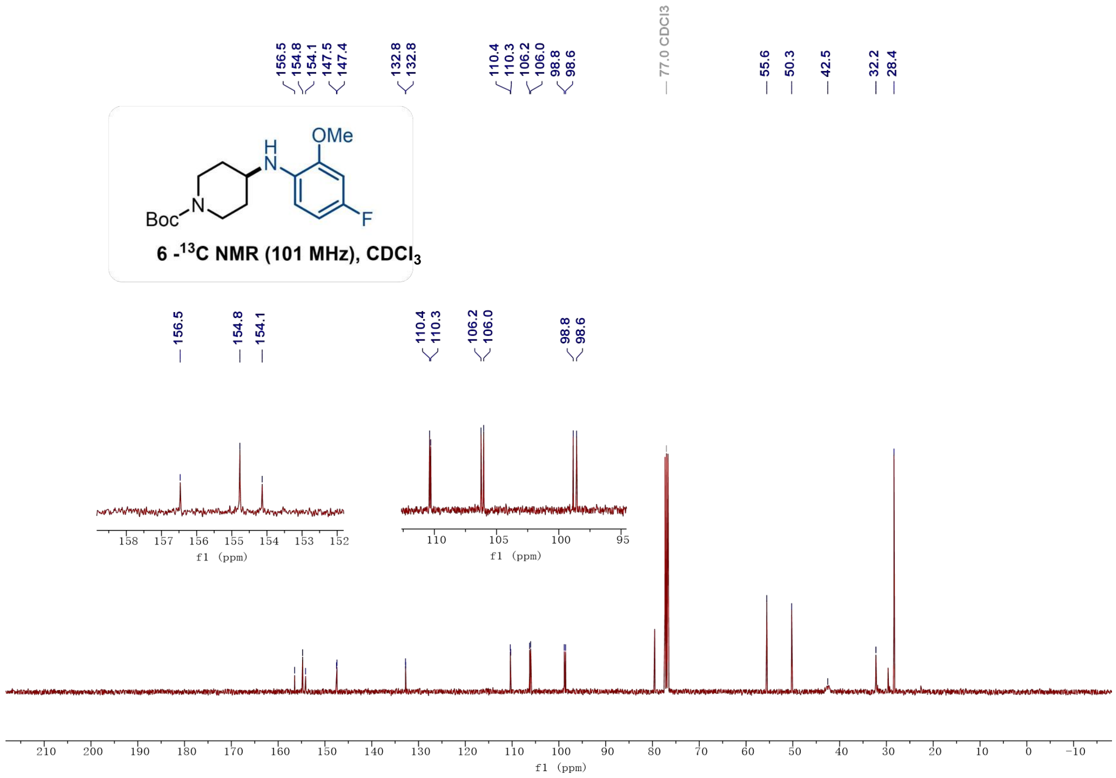

Supplementary Figure 62. ^{13}C NMR (101 MHz, $CDCl_3$) spectrum of compound **6**

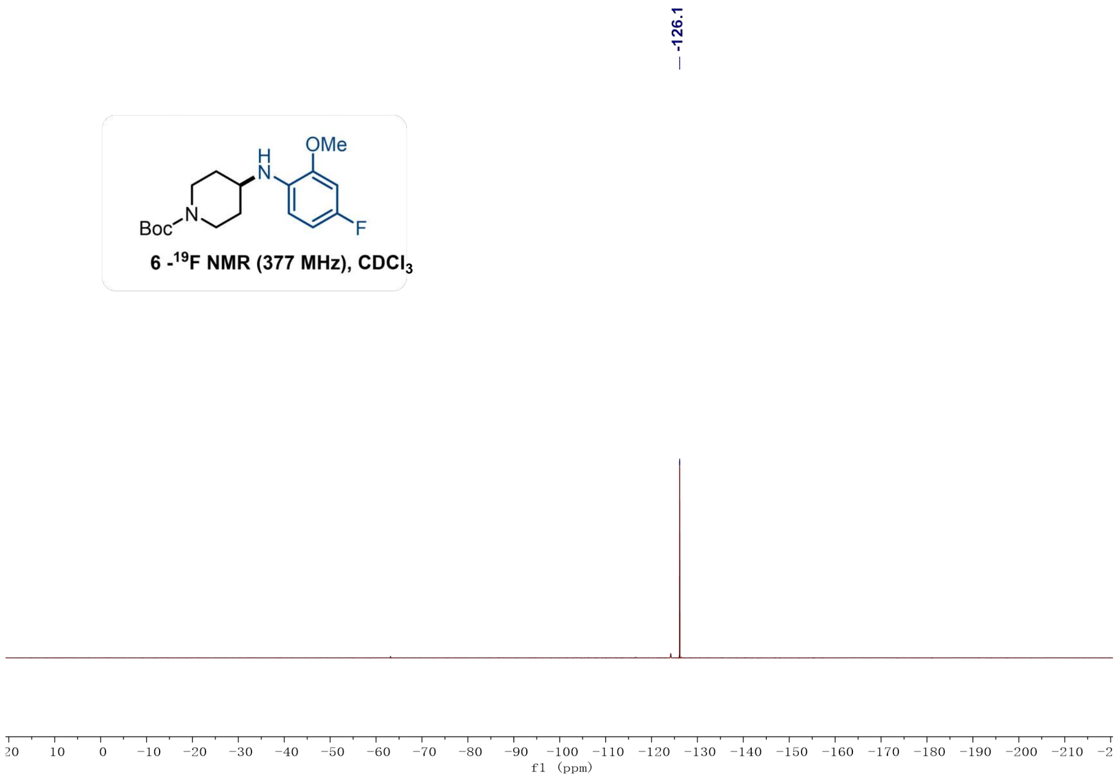

Supplementary Figure 63. ^{19}F NMR (377 MHz, $CDCl_3$) spectrum of compound **6**

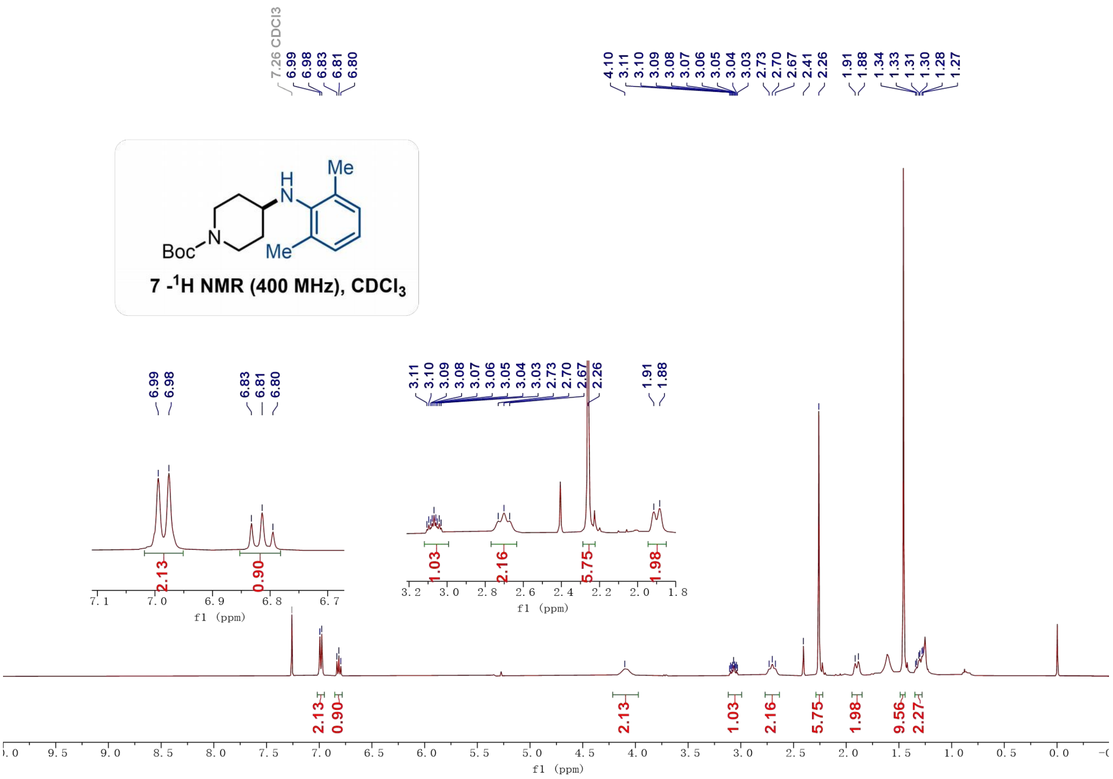

Supplementary Figure 64. ^{1}H NMR (400 MHz, $CDCl_3$) spectrum of compound **7**

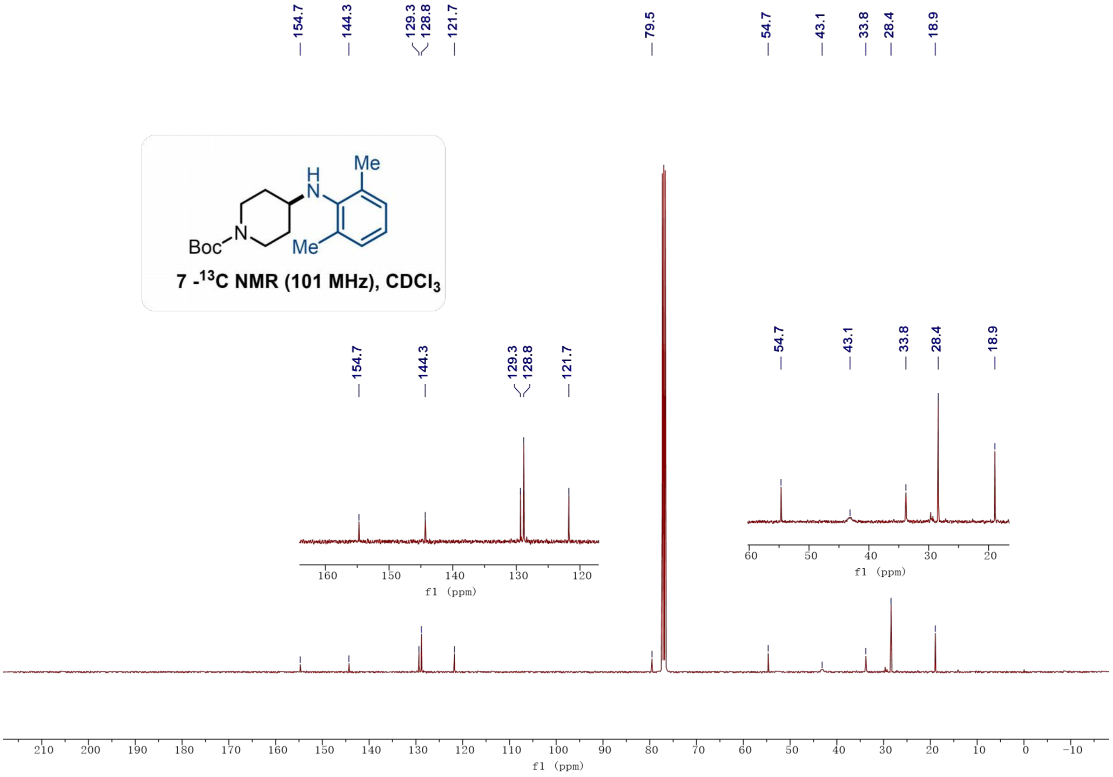

Supplementary Figure 65. ^{13}C NMR (101 MHz, $CDCl_3$) spectrum of compound **7**

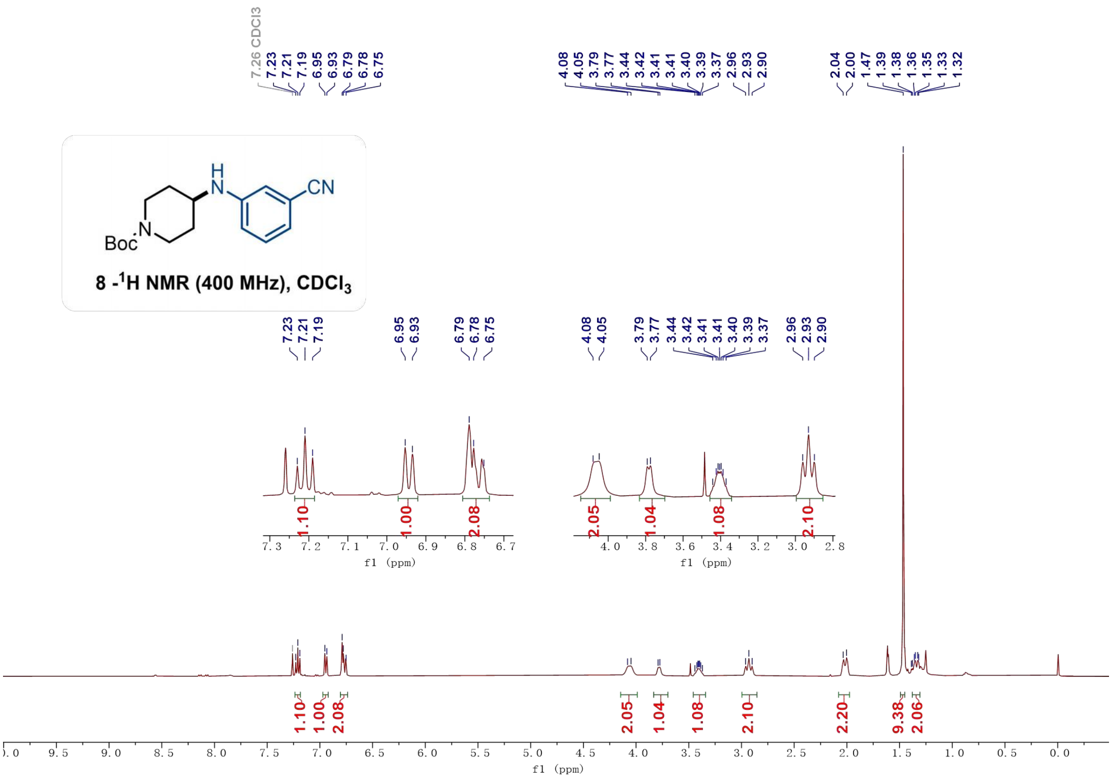

Supplementary Figure 66. ^{1}H NMR (400 MHz, $CDCl_3$) spectrum of compound **8**

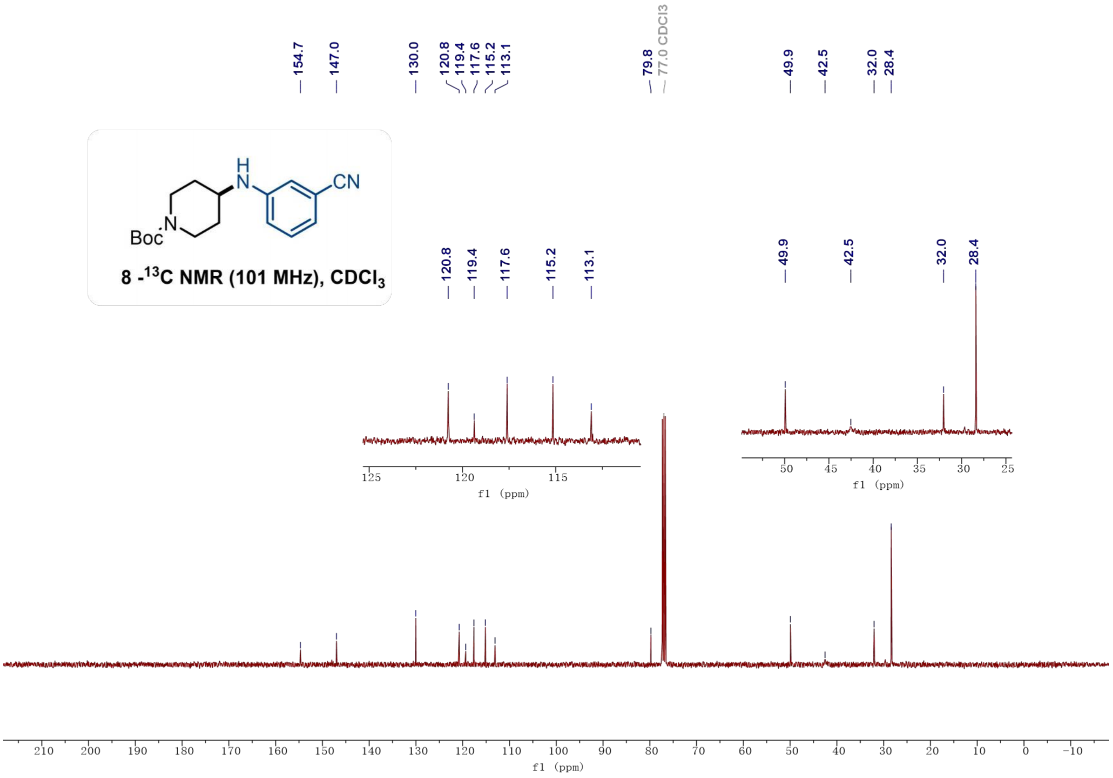

Supplementary Figure 67. ^{13}C NMR (101 MHz, $CDCl_3$) spectrum of compound **8**

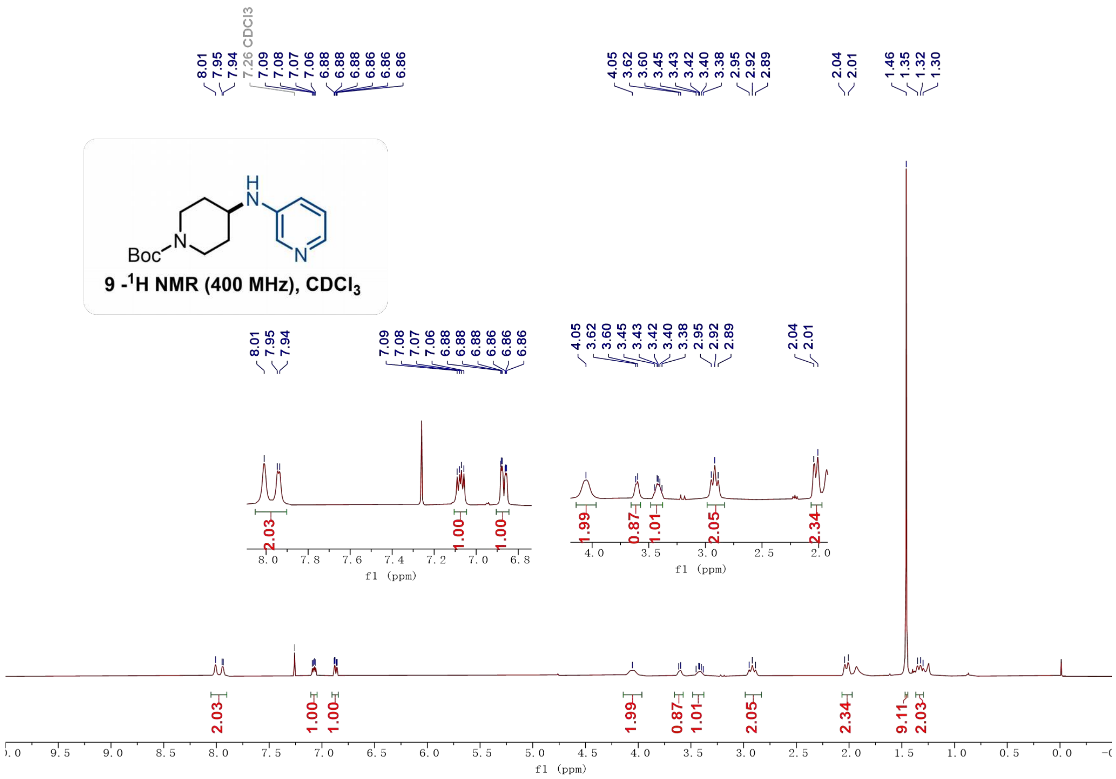

Supplementary Figure 68. ^{1}H NMR (400 MHz, $CDCl_3$) spectrum of compound **9**

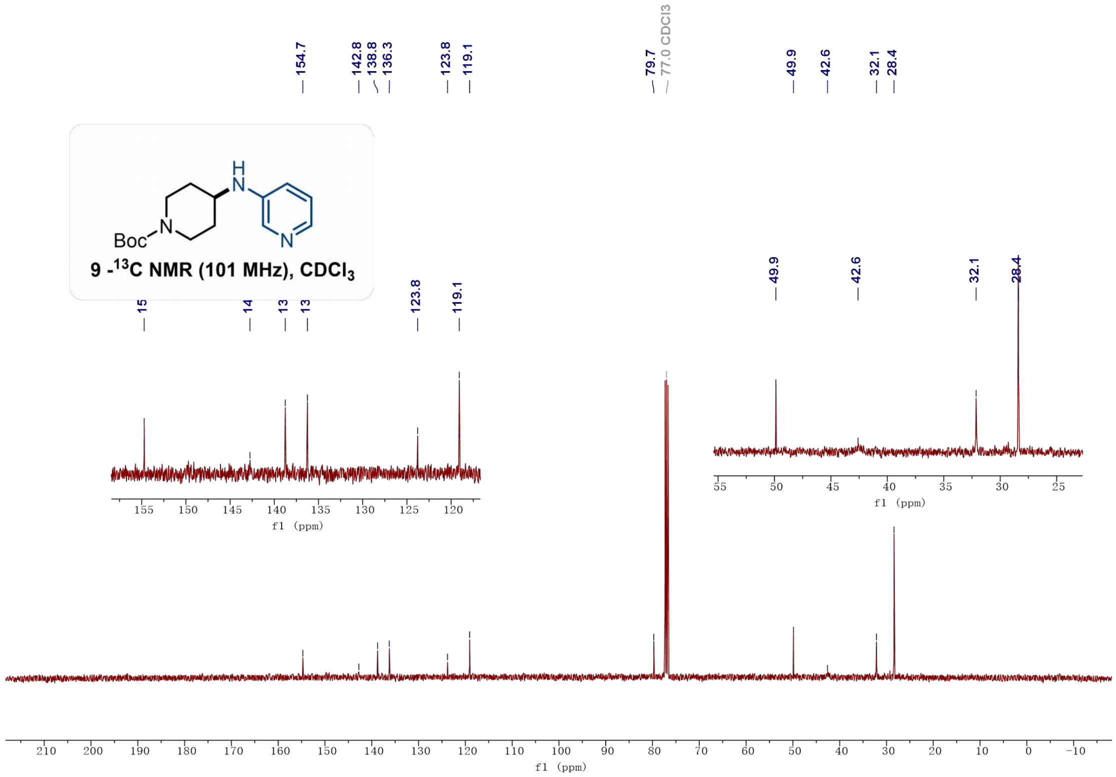

Supplementary Figure 69. ^{13}C NMR (101 MHz, $CDCl_3$) spectrum of compound **9**

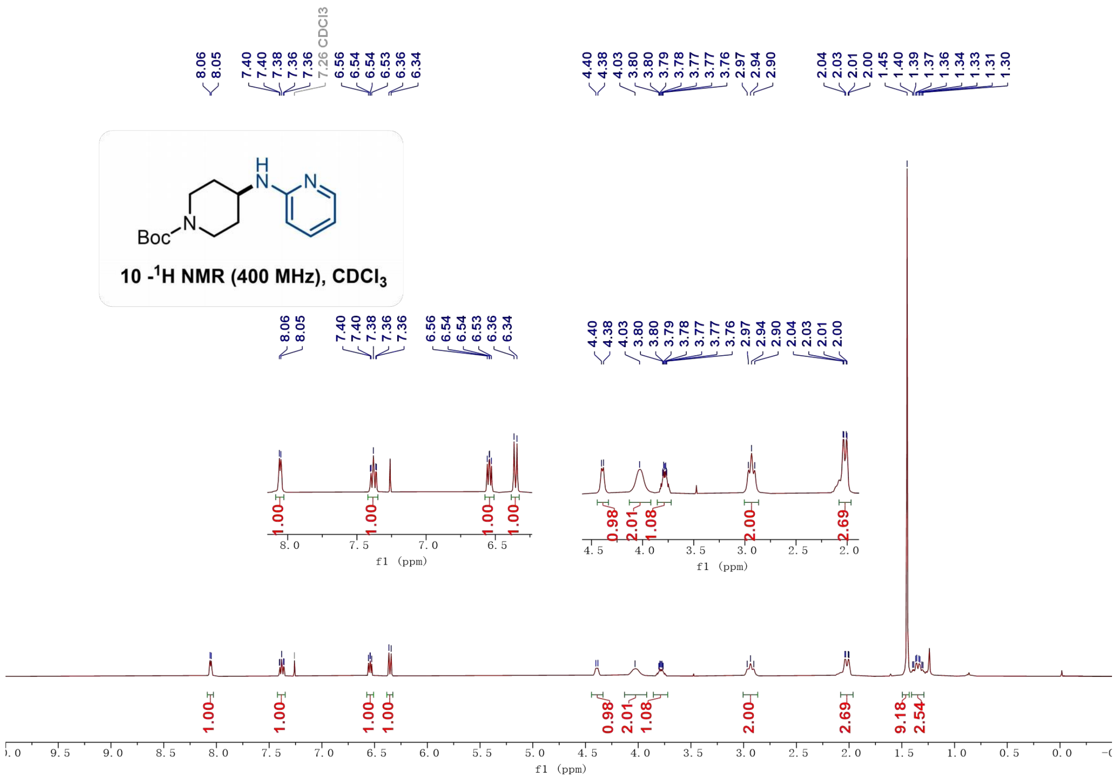

Supplementary Figure 70. ^{1}H NMR (400 MHz, $CDCl_3$) spectrum of compound **10**

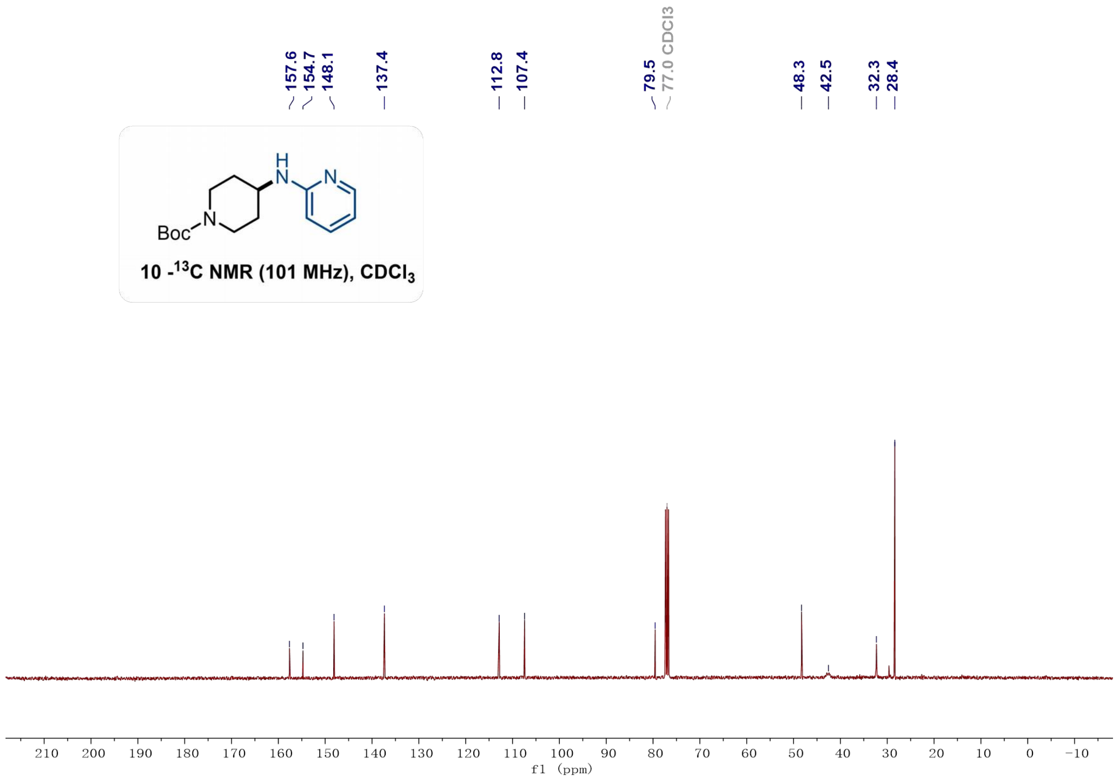

Supplementary Figure 71. ^{13}C NMR (101 MHz, $CDCl_3$) spectrum of compound **10**

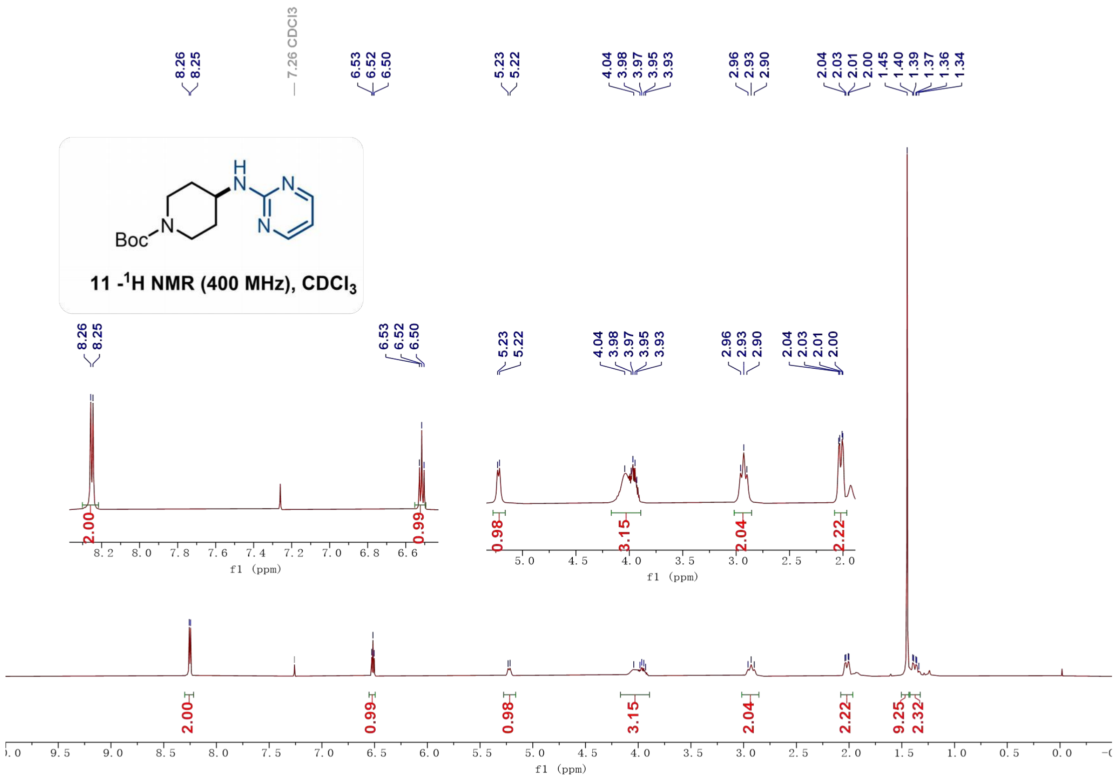

Supplementary Figure 72. ^{1}H NMR(400 MHz, $CDCl_3$) spectrum of compound **11**

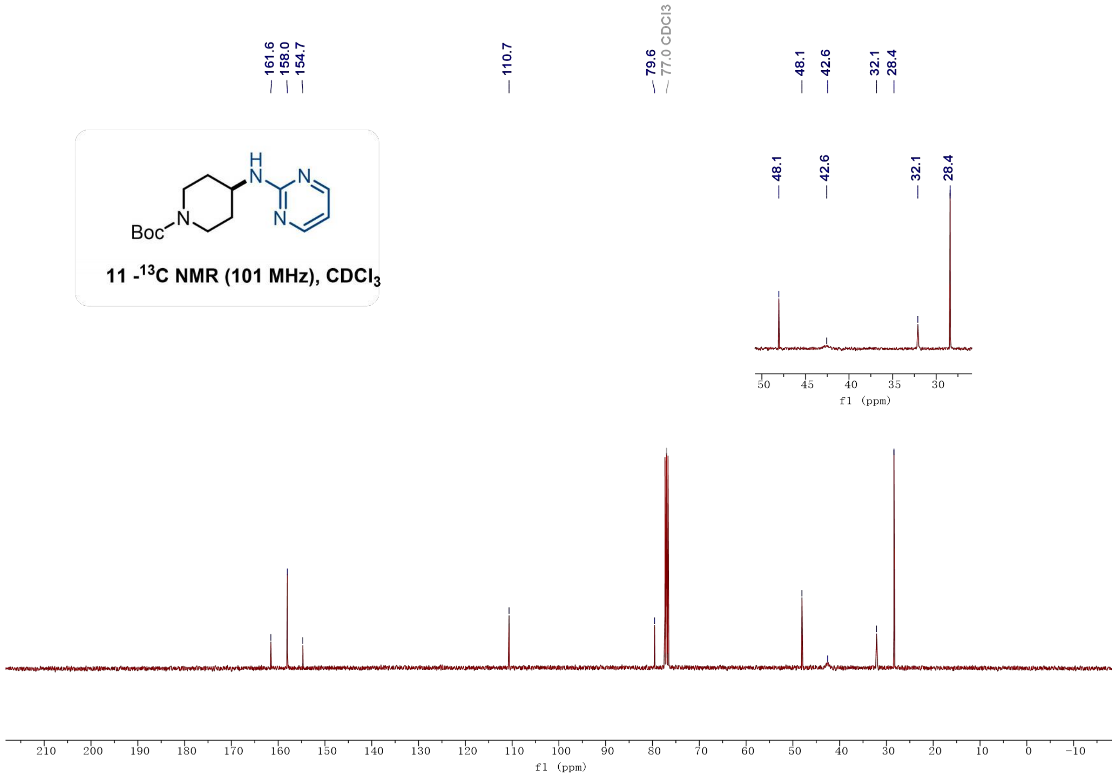

Supplementary Figure 73. ^{13}C NMR (101 MHz, $CDCl_3$) spectrum of compound **11**

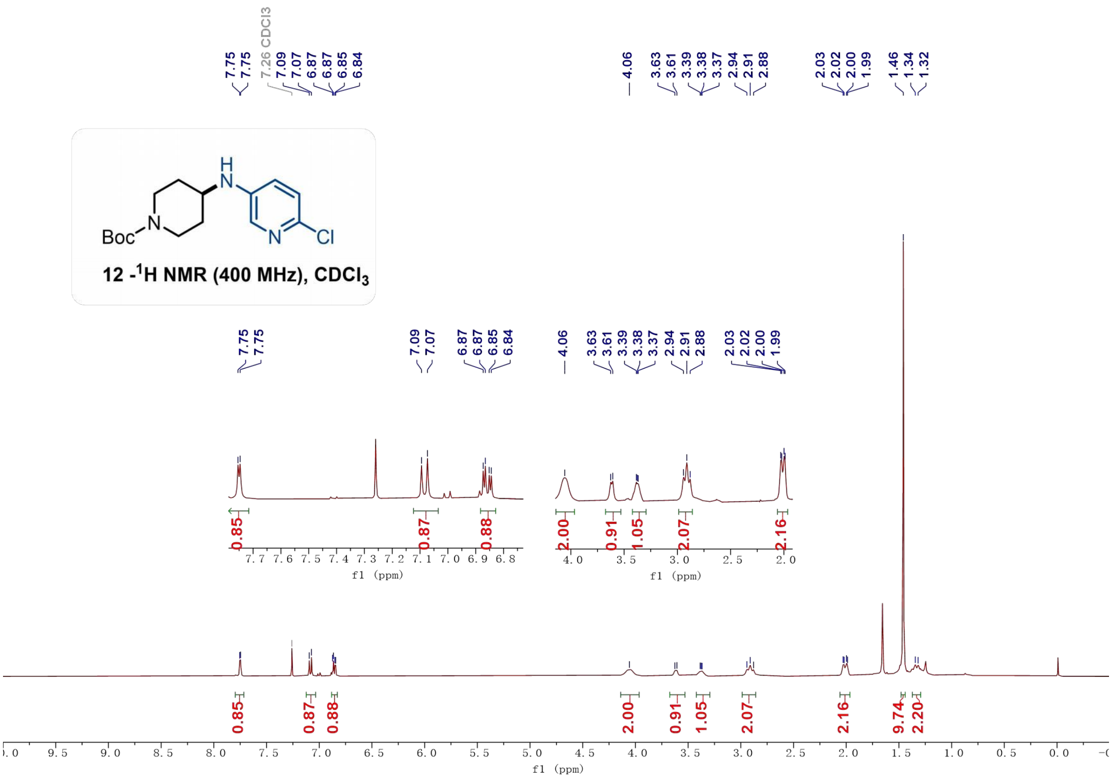

Supplementary Figure 74. ^{1}H NMR(400 MHz, $CDCl_3$) spectrum of compound **12**

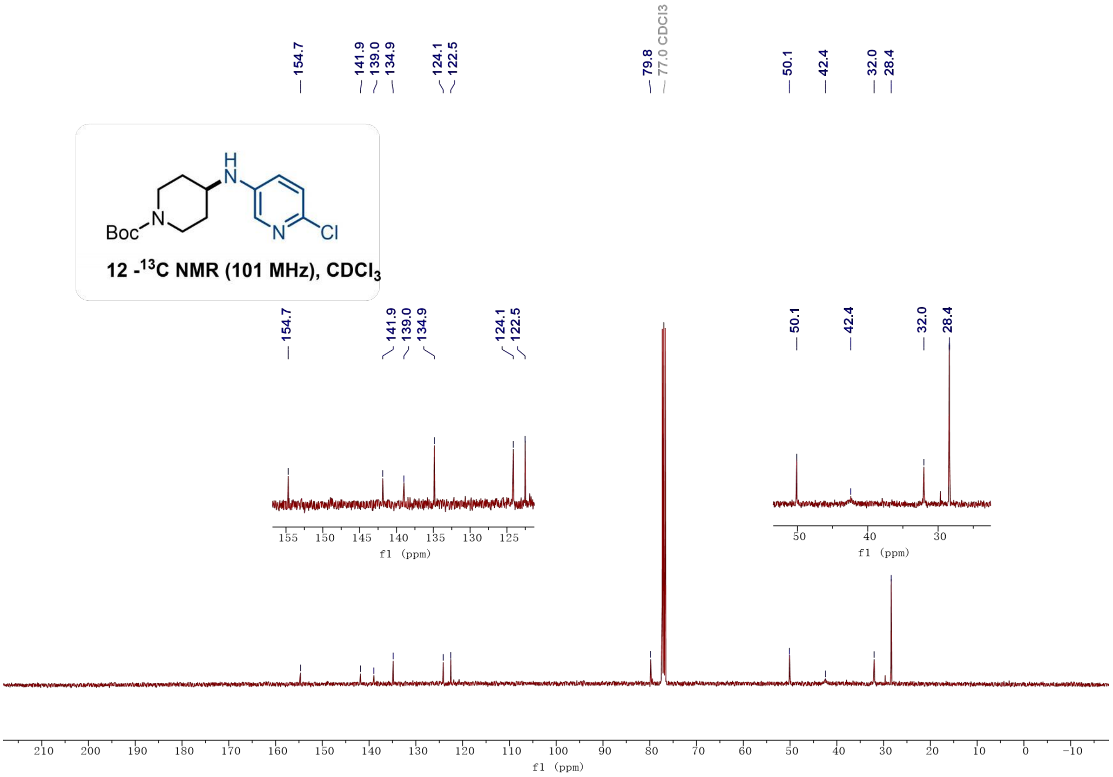

Supplementary Figure 75. ^{13}C NMR (101 MHz, $CDCl_3$) spectrum of compound **12**

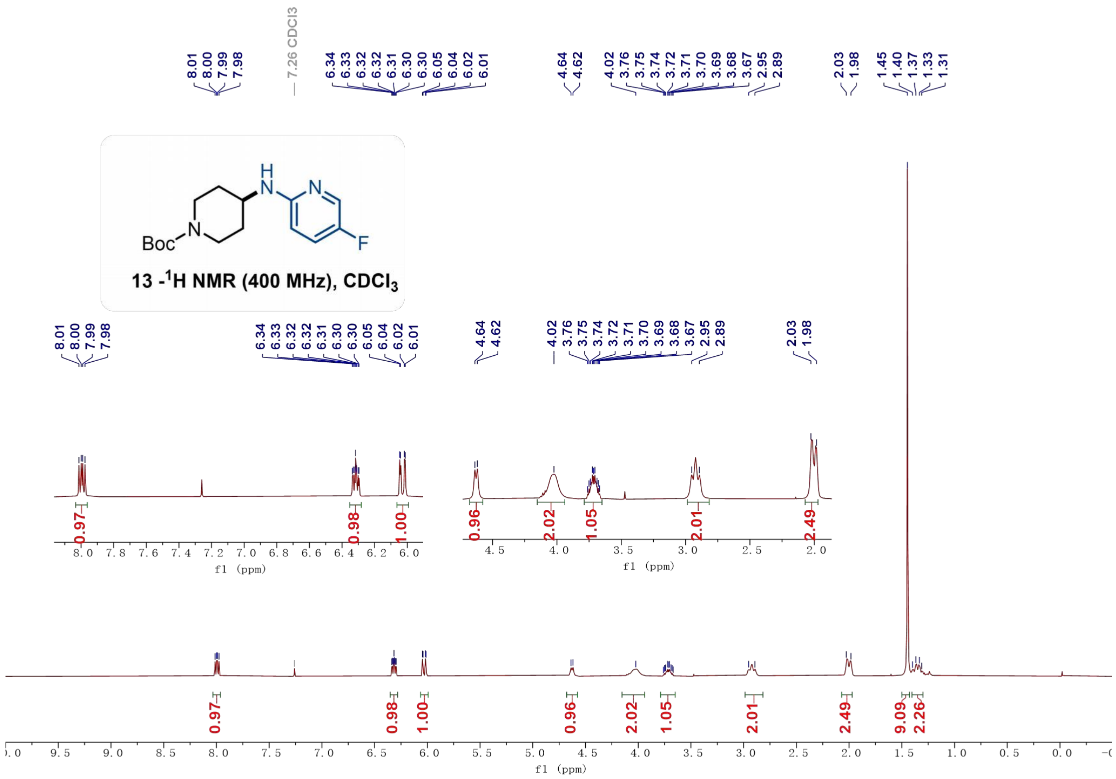

Supplementary Figure 76. ^{1}H NMR (400 MHz, $CDCl_3$) spectrum of compound 1**3**

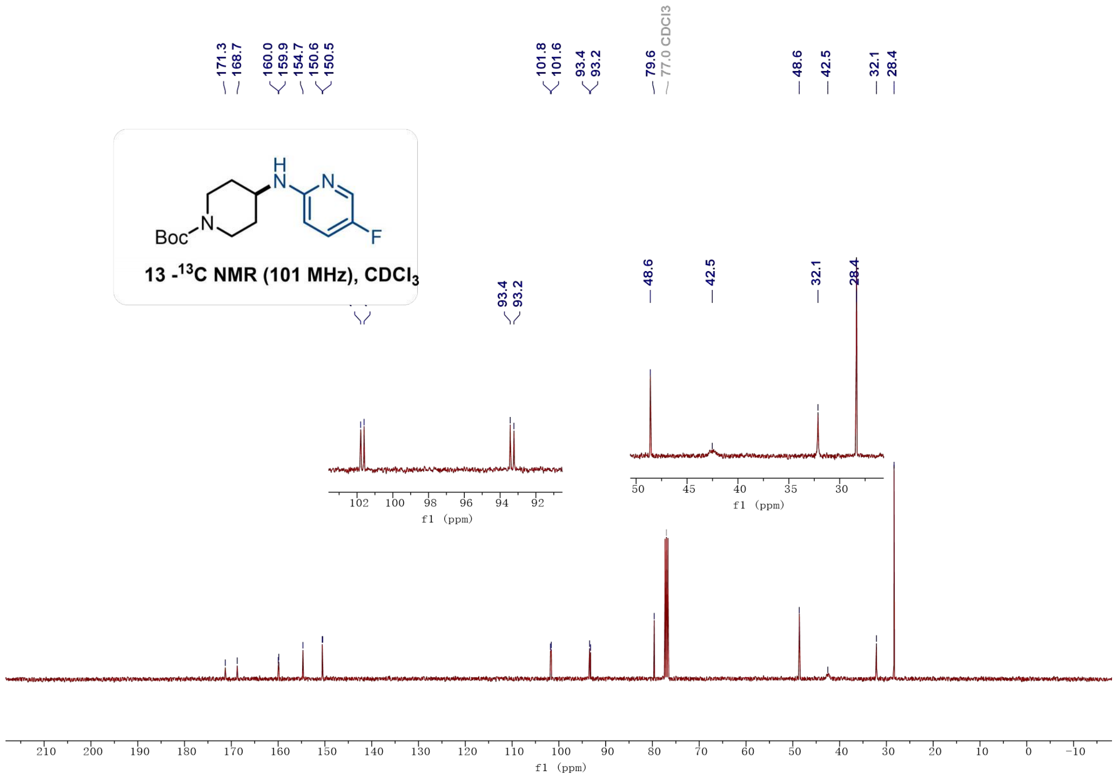

Supplementary Figure 77. ^{13}C NMR (101 MHz, $CDCl_3$) spectrum of compound **13**

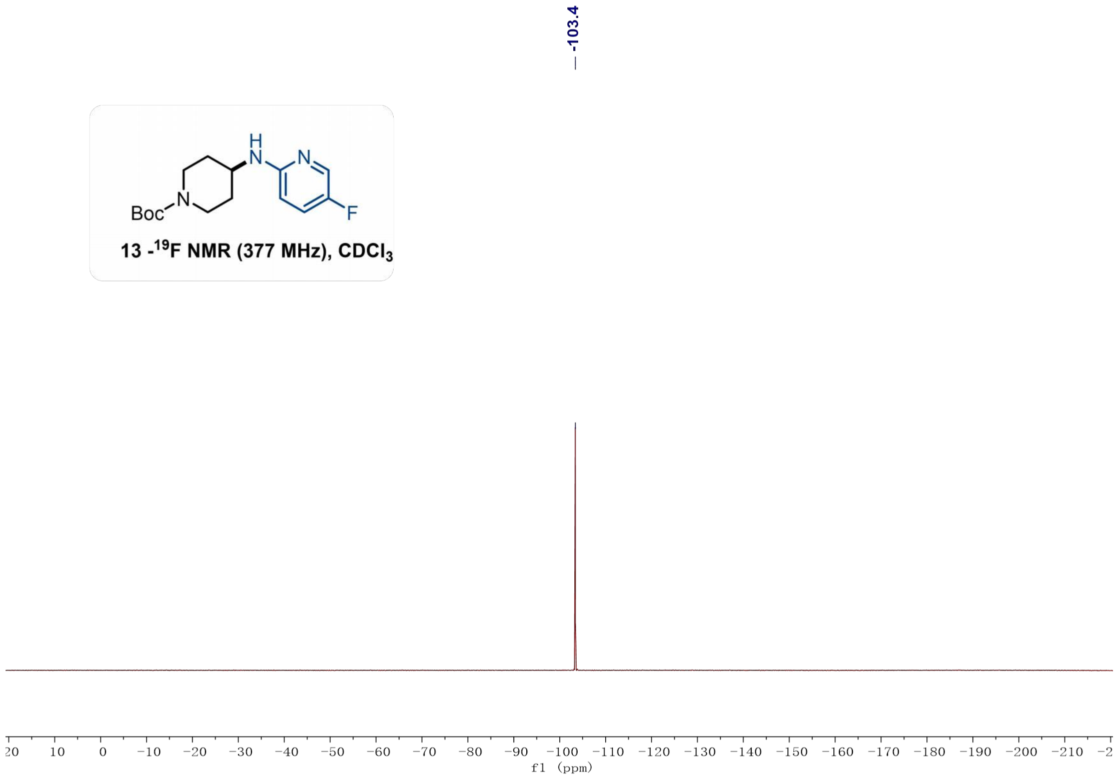

Supplementary Figure 78. ^{19}F NMR (377 MHz, $CDCl_3$) spectrum of compound **13**

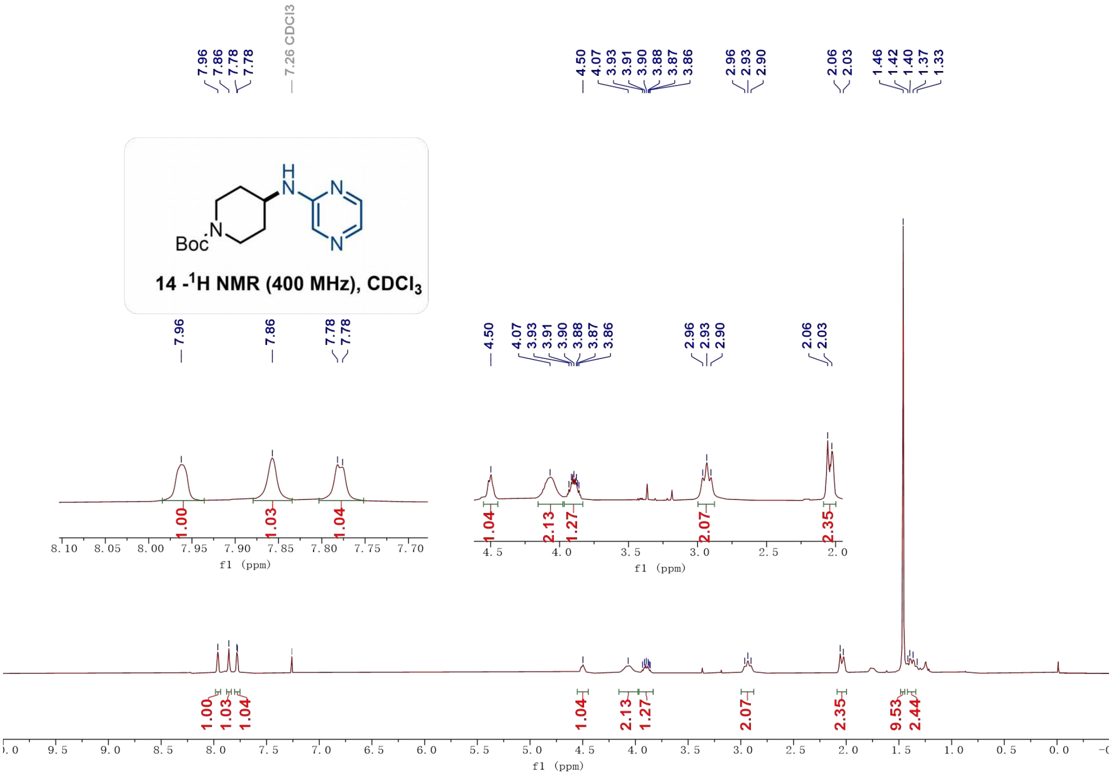

Supplementary Figure 79. ^{1}H NMR (400 MHz, $CDCl_3$) spectrum of compound **14**

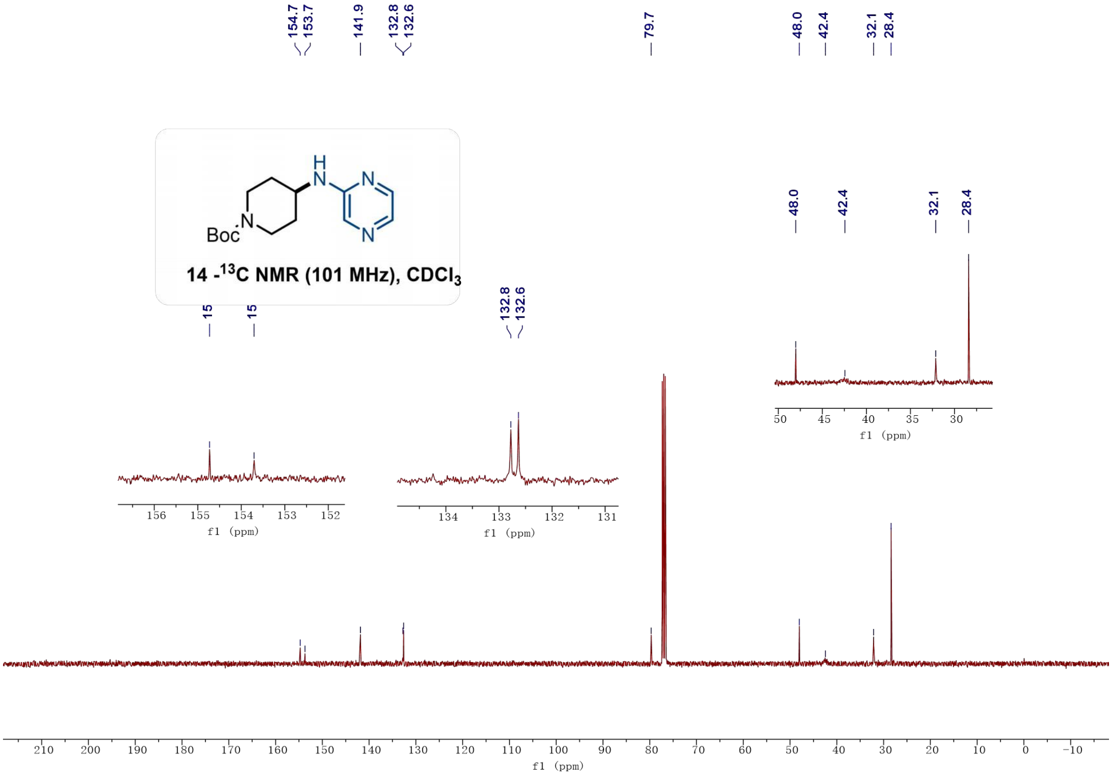

Supplementary Figure 80. ^{13}C NMR (101 MHz, $CDCl_3$) spectrum of compound **14**

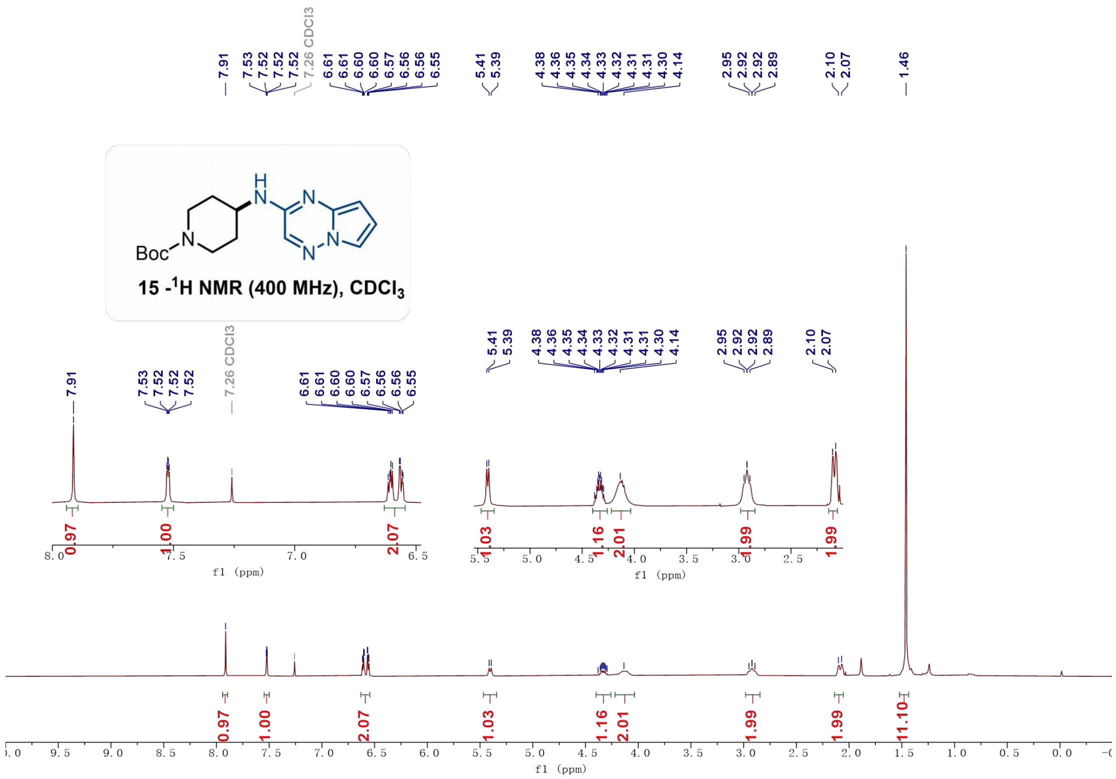

Supplementary Figure 81. ^{1}H NMR (400 MHz, $CDCl_3$) spectrum of compound **15**

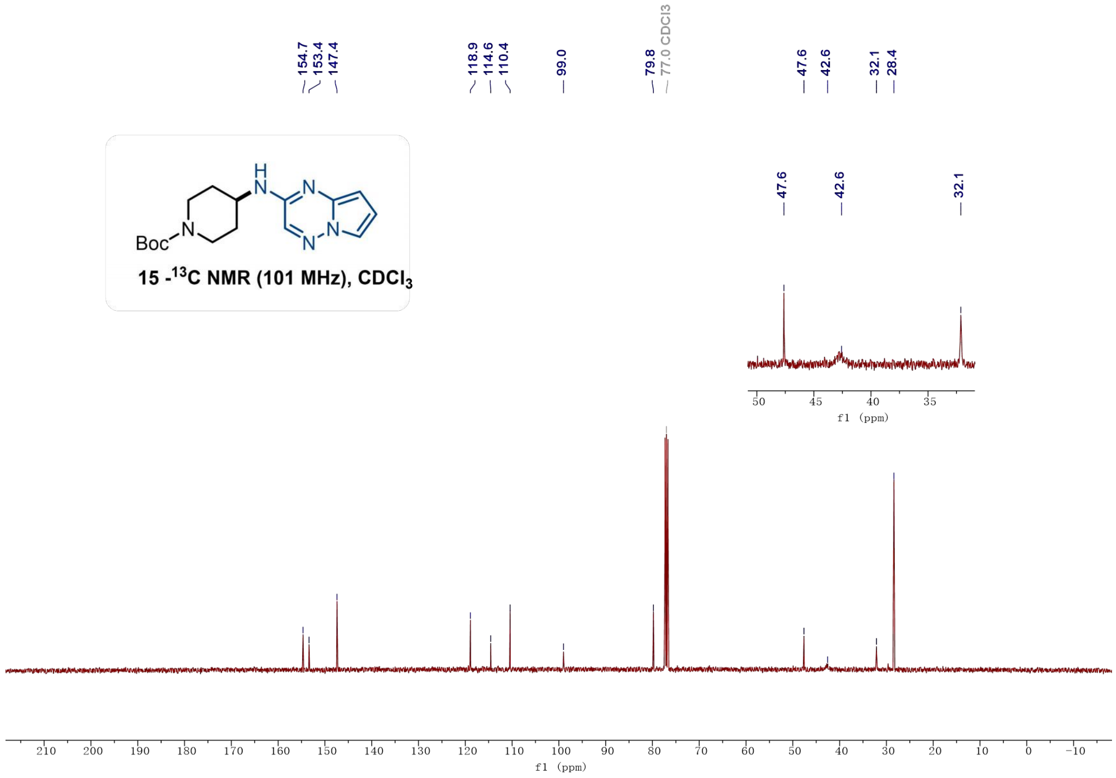

Supplementary Figure 82. ^{13}C NMR (101 MHz, $CDCl_3$) spectrum of compound **15**

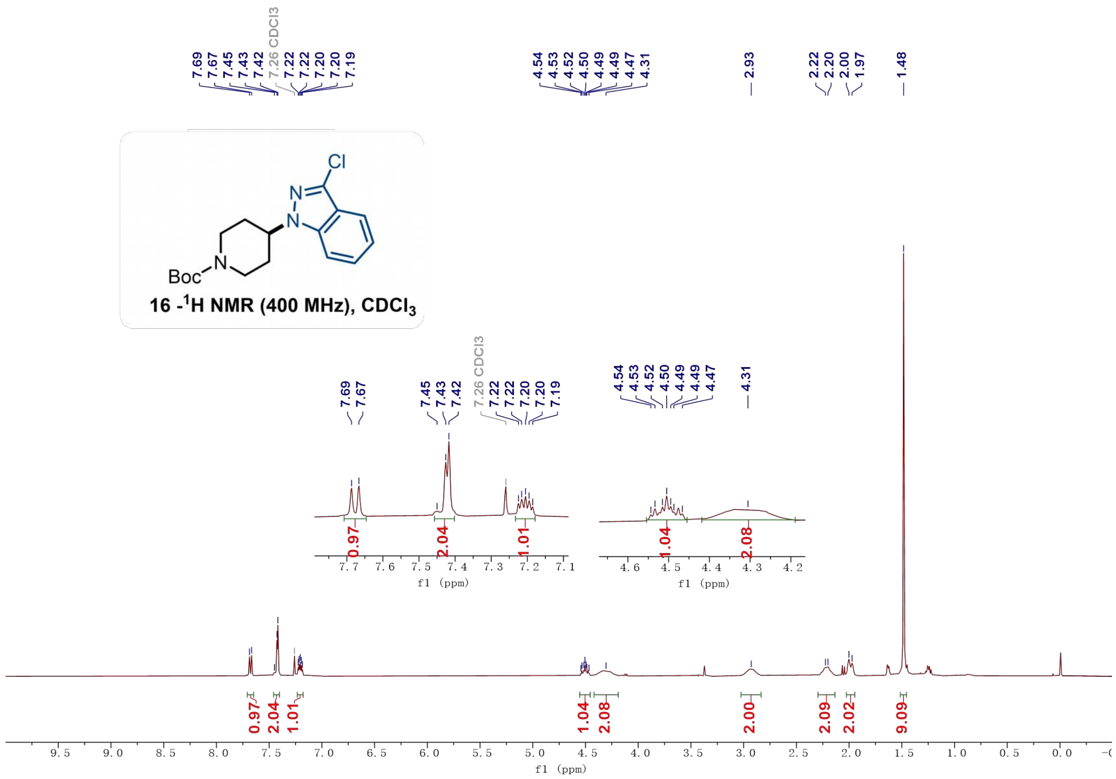

Supplementary Figure 83. ^{1}H NMR (400 MHz, $CDCl_3$) spectrum of compound **16**

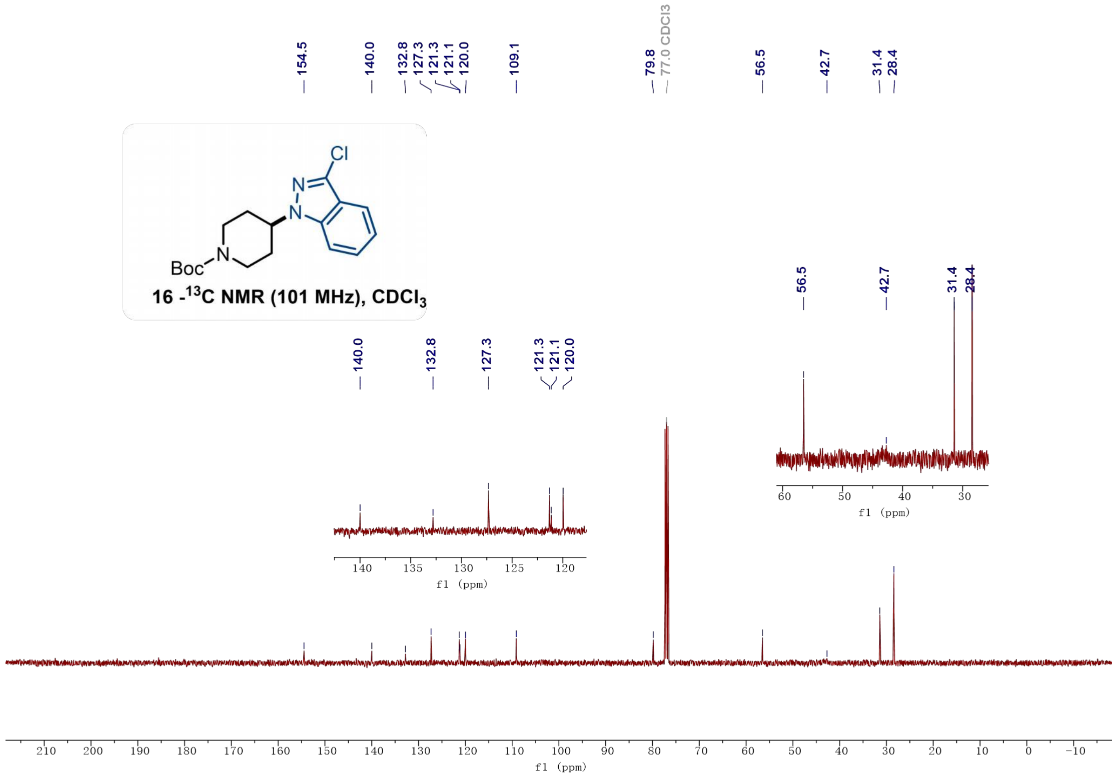

Supplementary Figure 84. ^{13}C NMR (101 MHz, $CDCl_3$) spectrum of compound **16**

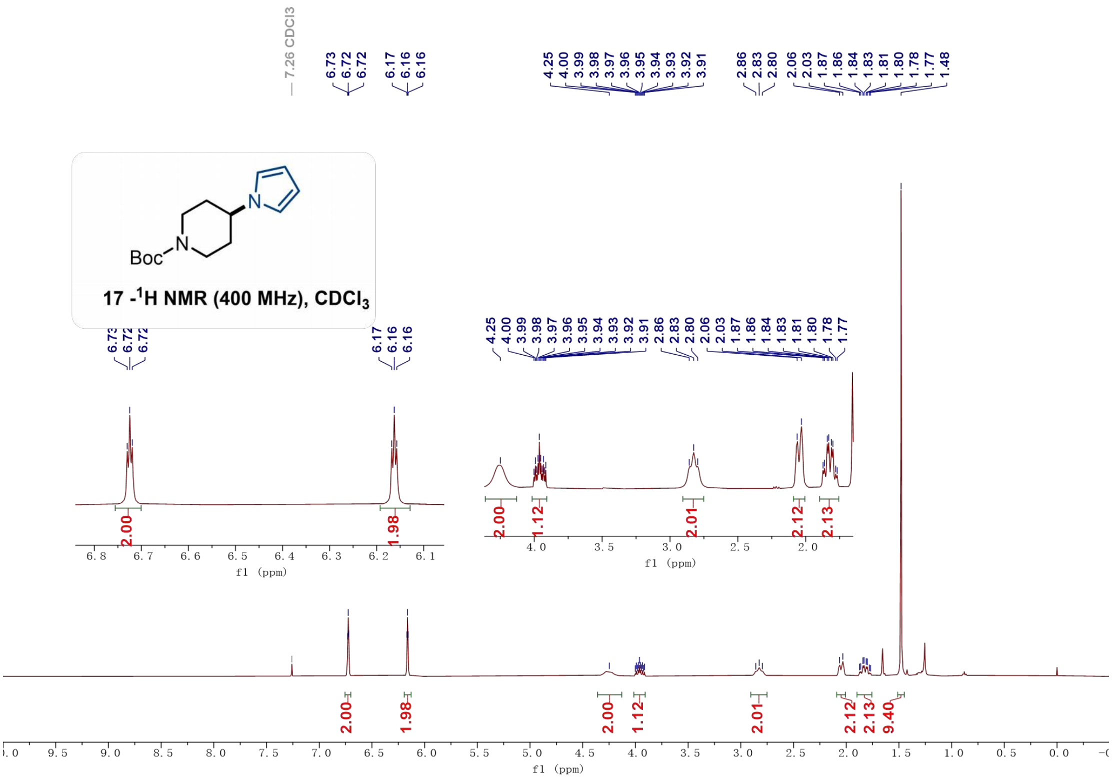

Supplementary Figure 85. ^{1}H NMR (400 MHz, $CDCl_3$) spectrum of compound **17**

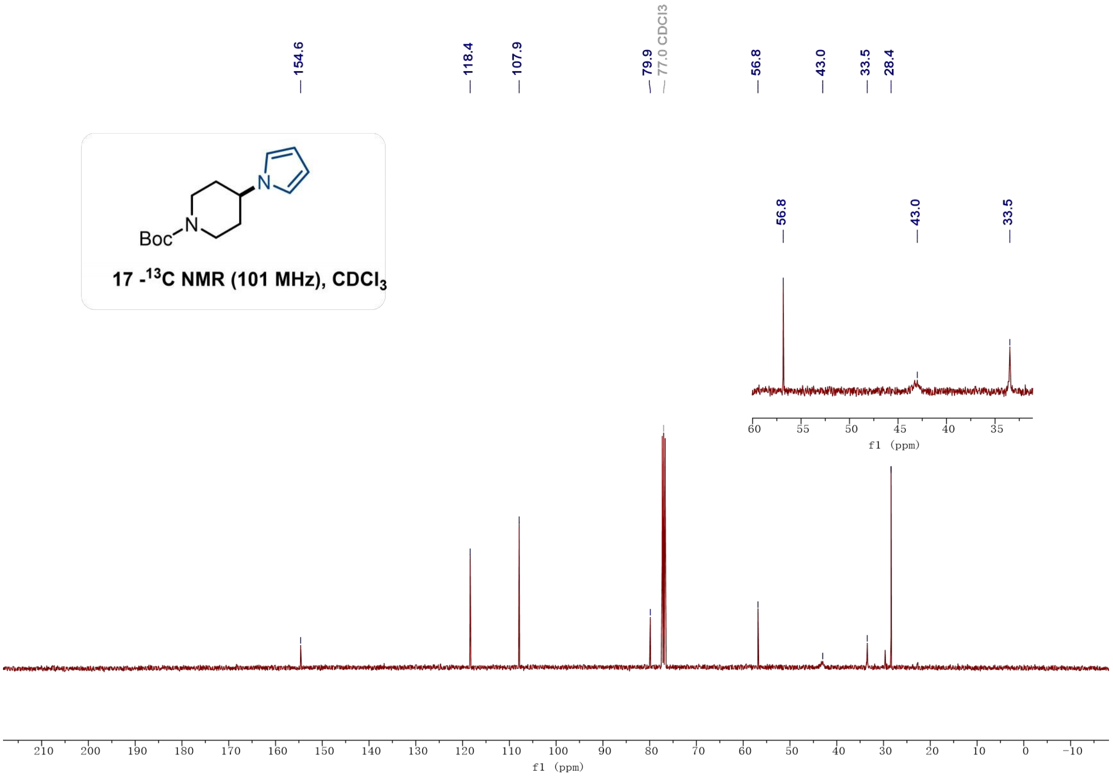

Supplementary Figure 86. ^{13}C NMR (101 MHz, $CDCl_3$) spectrum of compound **17**

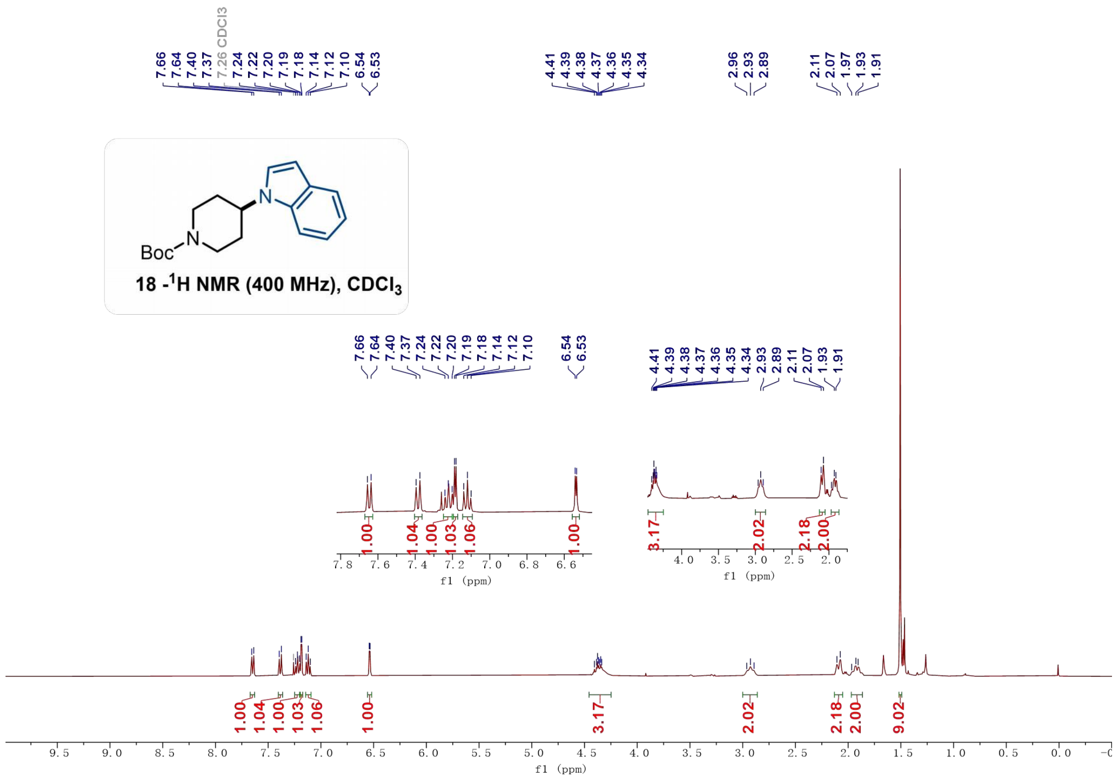

Supplementary Figure 87. ^{1}H NMR (400 MHz, $CDCl_3$) spectrum of compound **18**

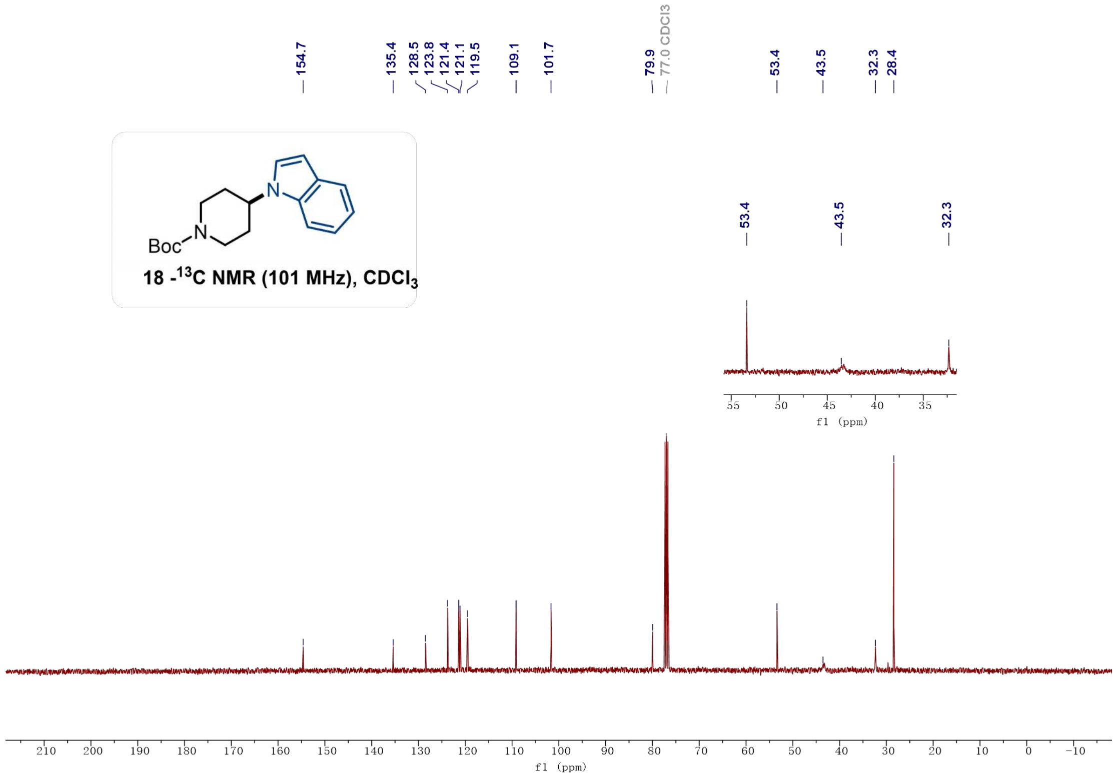

Supplementary Figure 88. ^{13}C NMR (101 MHz, $CDCl_3$) spectrum of compound **18**

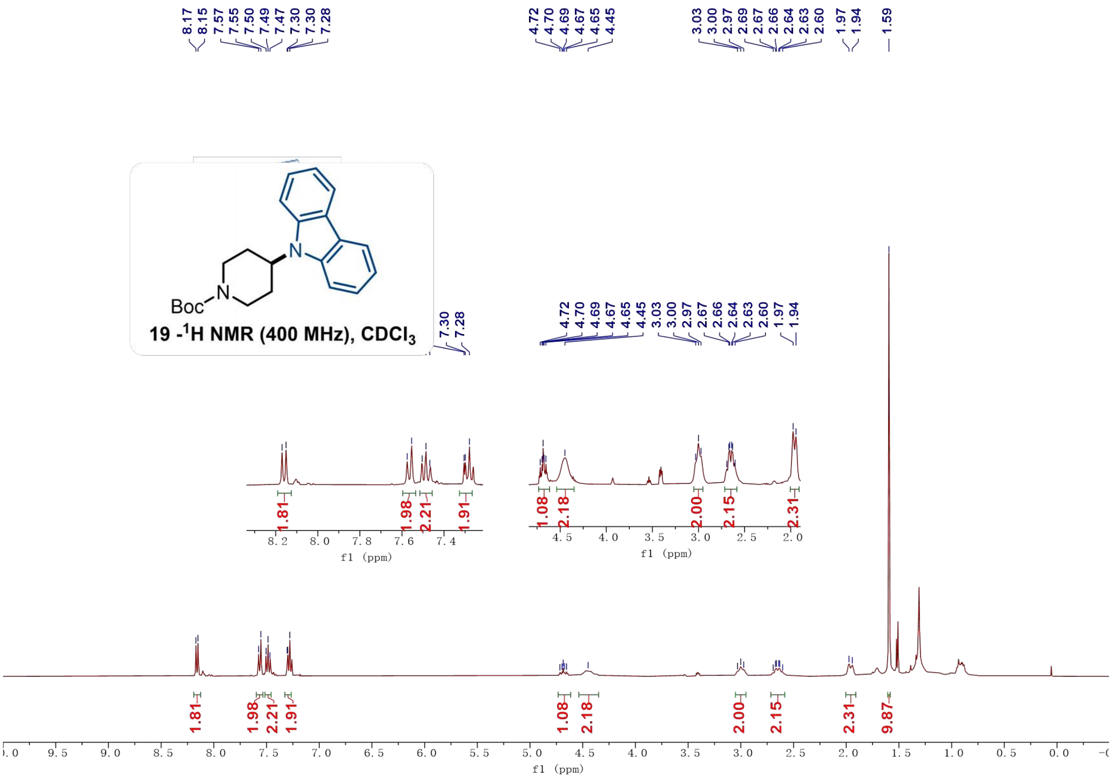

Supplementary Figure 89. ^{1}H NMR (400 MHz, $CDCl_3$) spectrum of compound **19**

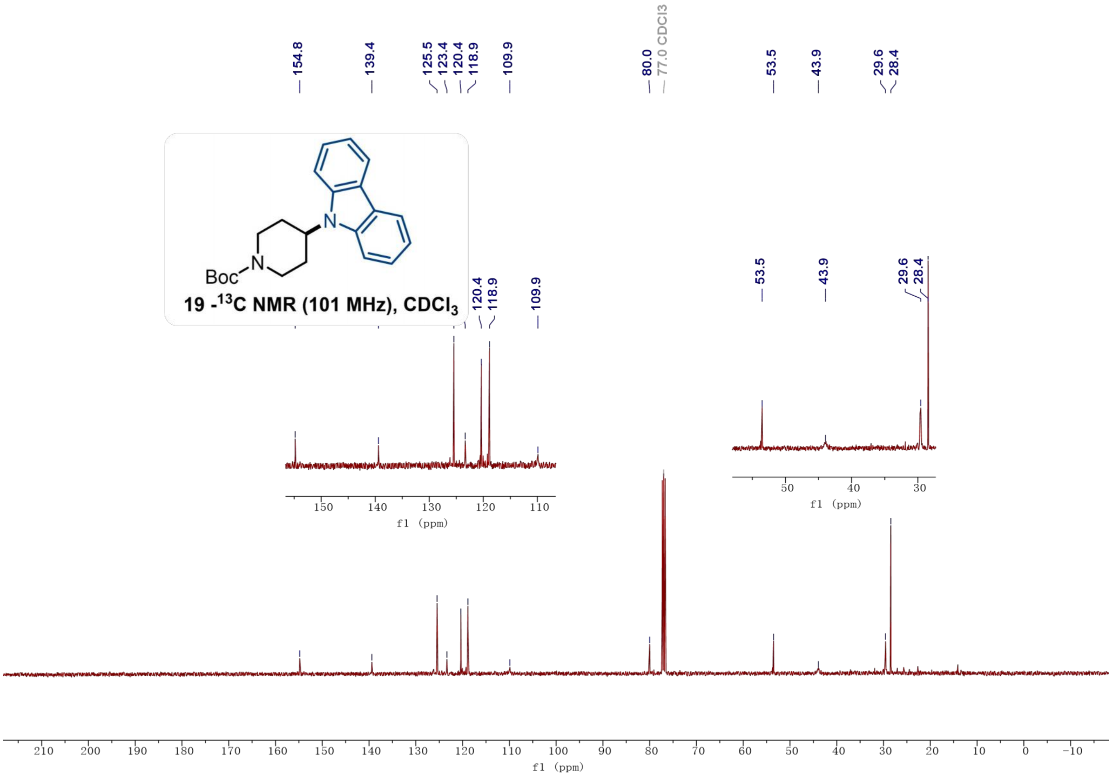

Supplementary Figure 90. ^{13}C NMR (101 MHz, $CDCl_3$) spectrum of compound **19**

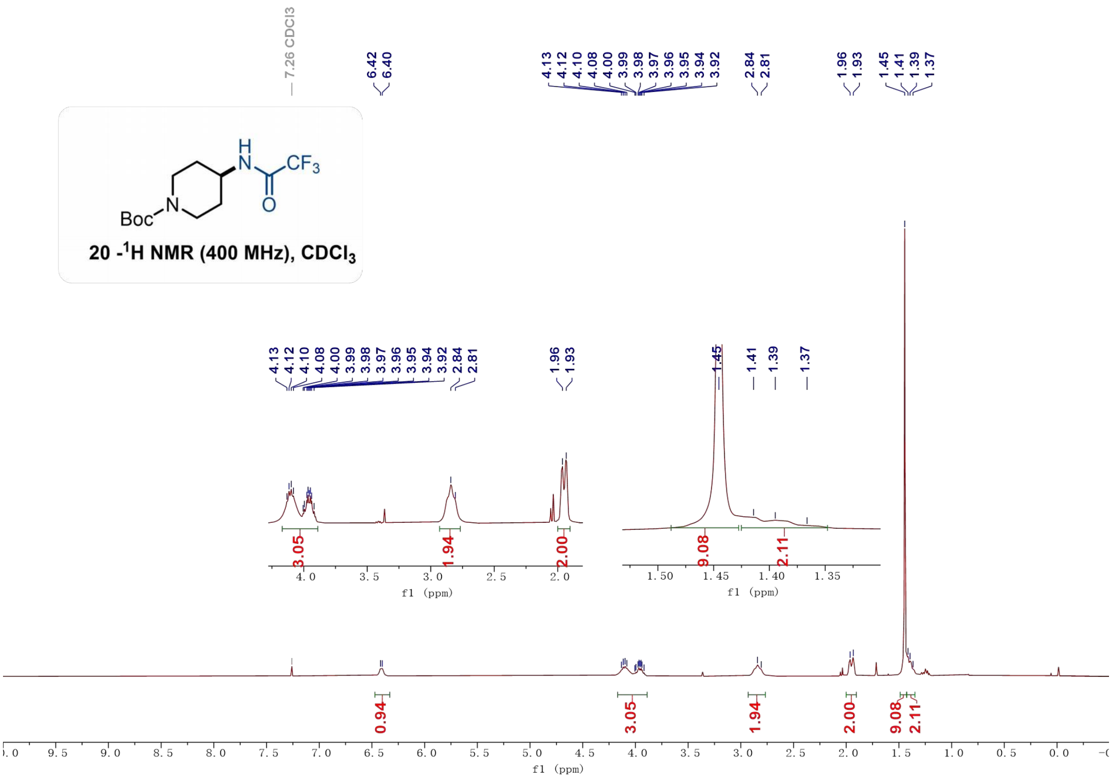

Supplementary Figure 91. ^{1}H NMR (400 MHz, $CDCl_3$) spectrum of compound **20**

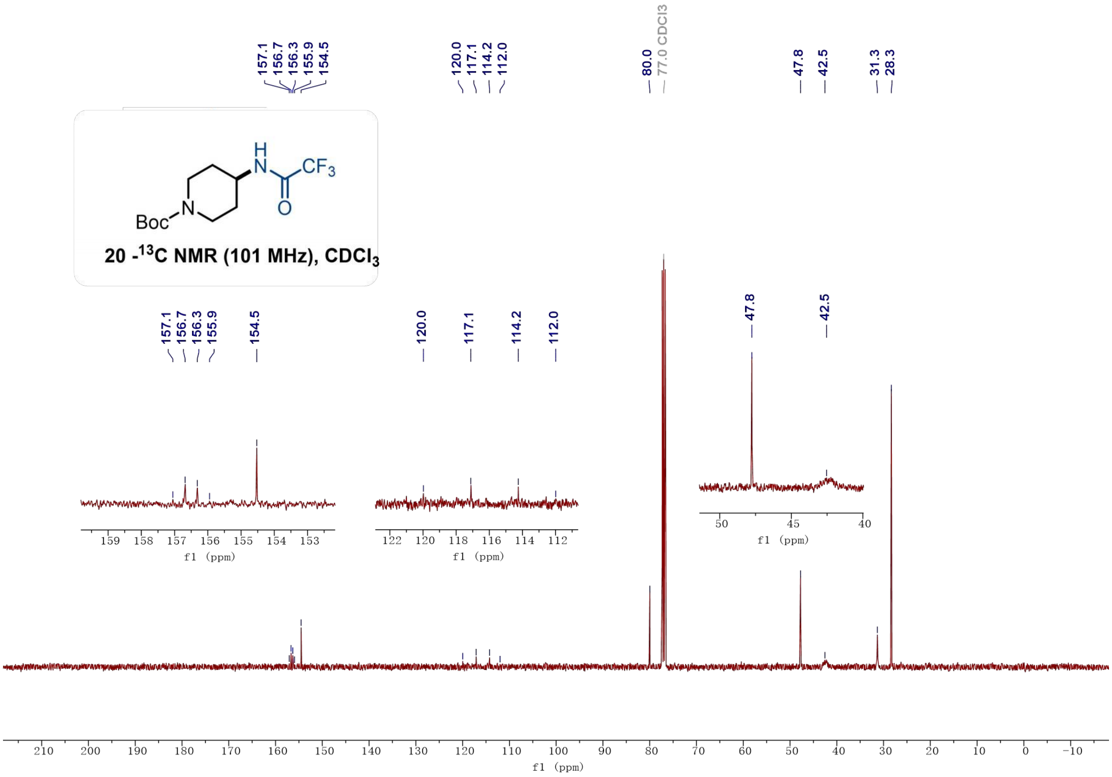

Supplementary Figure 92. ^{13}C NMR (101 MHz, $CDCl_3$) spectrum of compound **20**

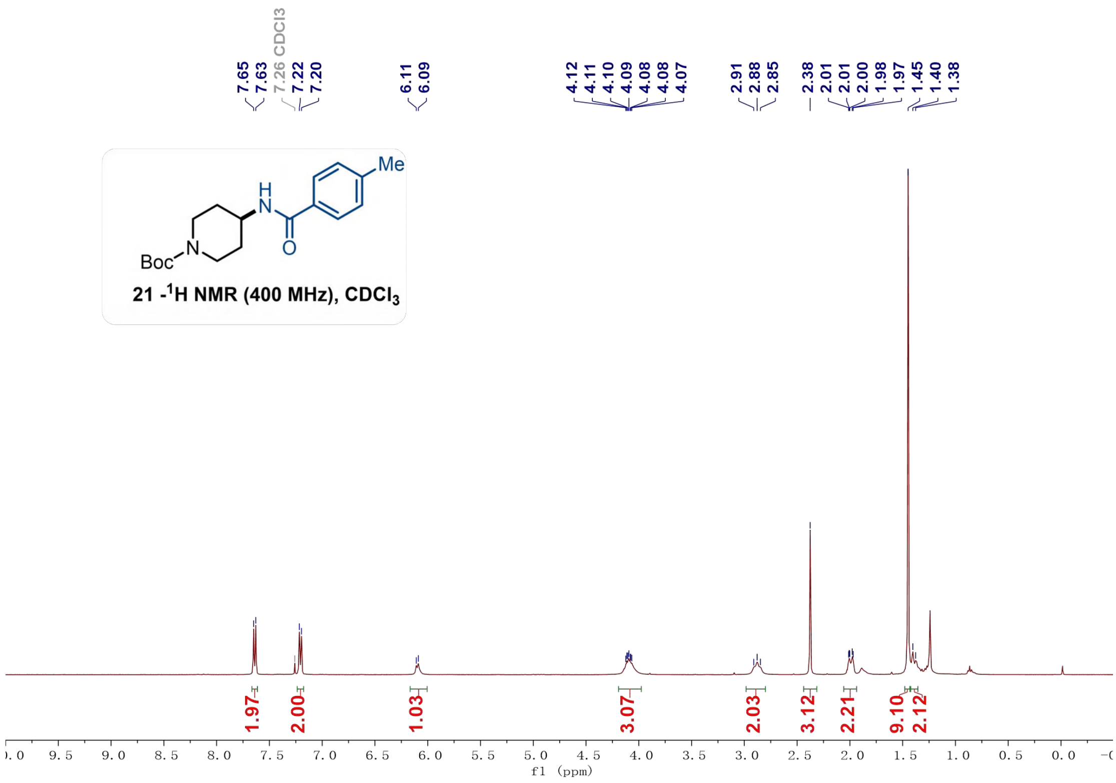

Supplementary Figure 93. ^{1}H NMR (400 MHz, $CDCl_3$) spectrum of compound **21**

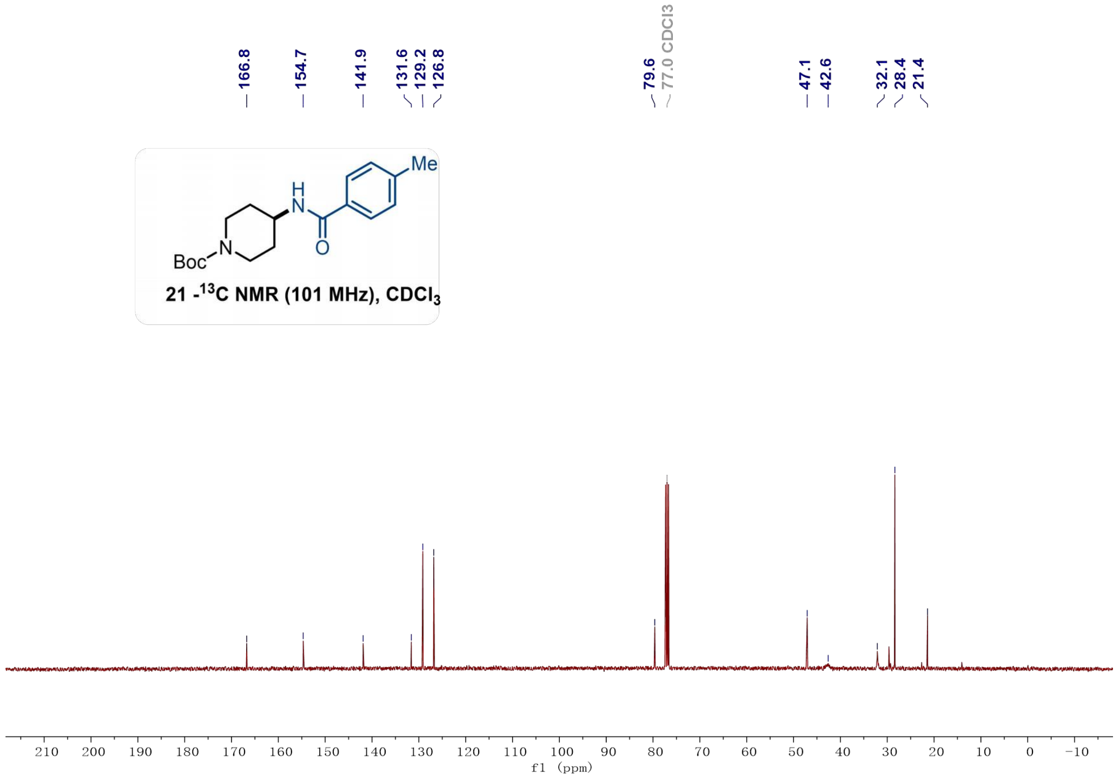

Supplementary Figure 94. ^{13}C NMR (101 MHz, $CDCl_3$) spectrum of compound **21**

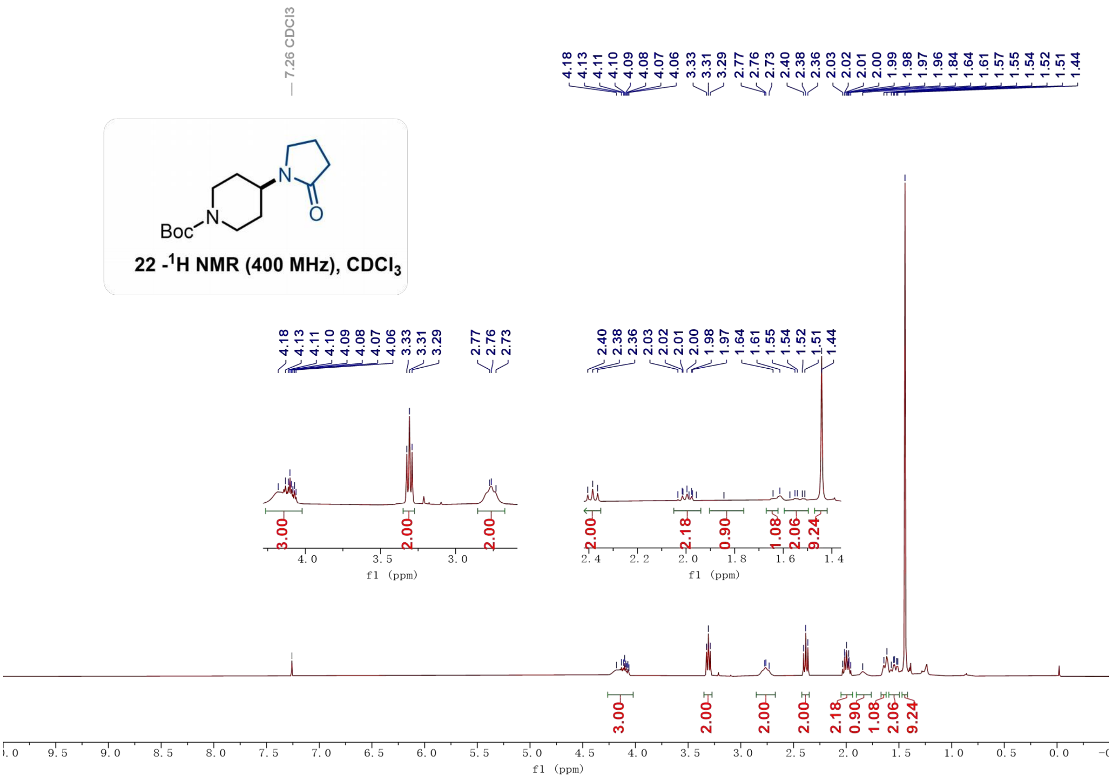

Supplementary Figure 95. ^{1}H NMR (400 MHz, $CDCl_3$) spectrum of compound **22**

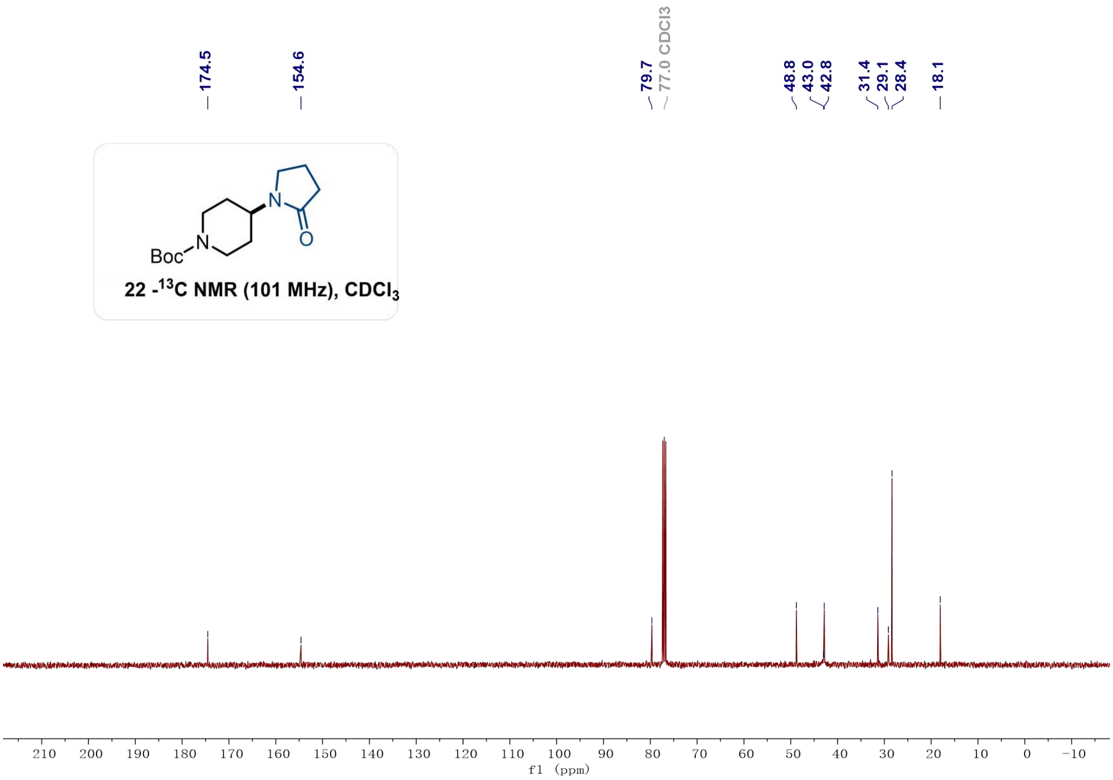

Supplementary Figure 96. ^{13}C NMR (101 MHz, $CDCl_3$) spectrum of compound **22**

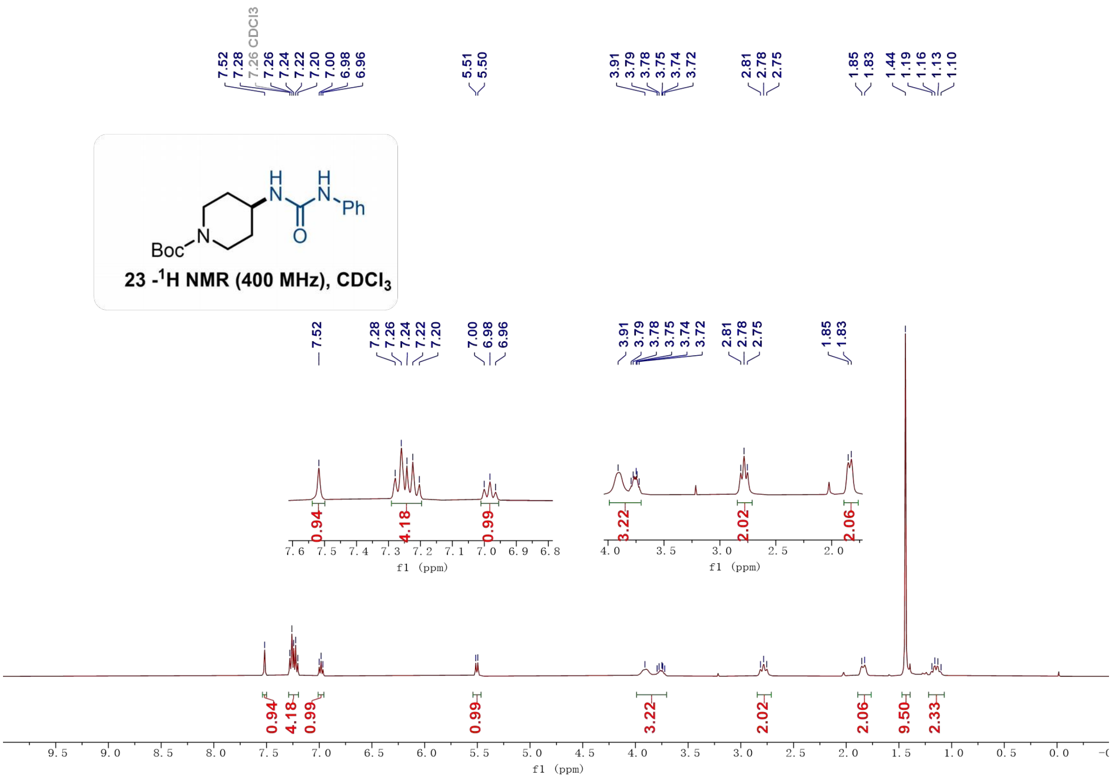

Supplementary Figure 97. ^{1}H NMR (400 MHz, $CDCl_3$) spectrum of compound **23**

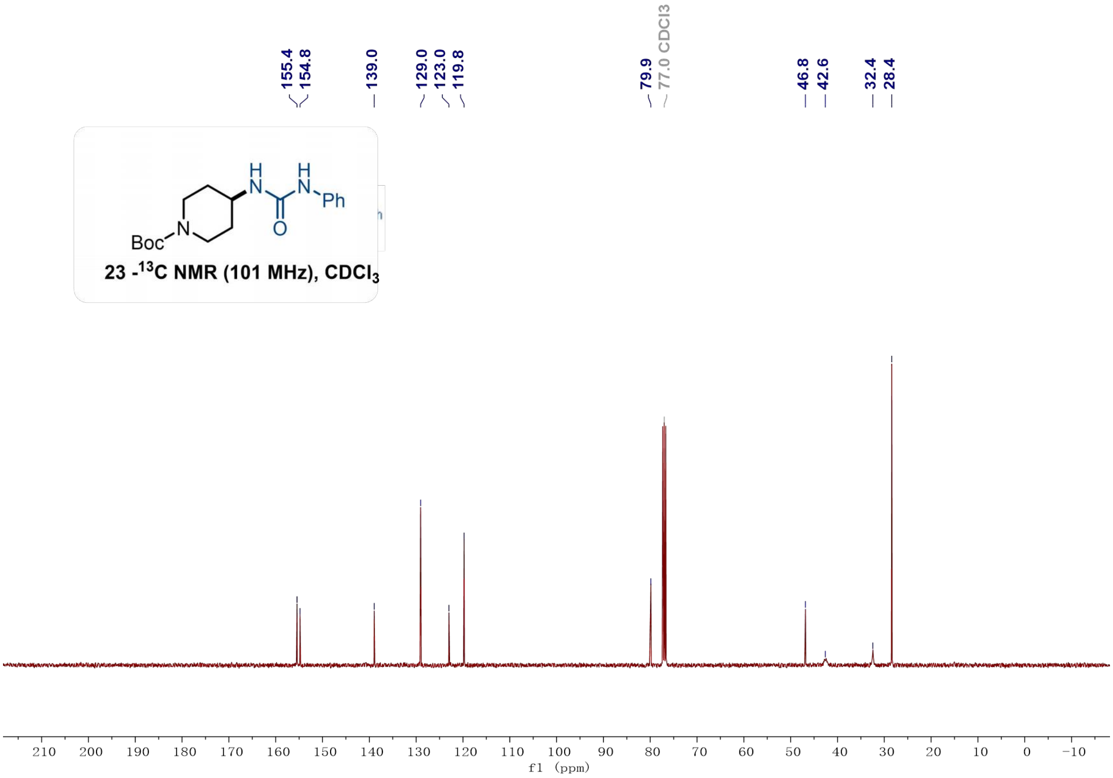

Supplementary Figure 98. ^{13}C NMR (101 MHz, $CDCl_3$) spectrum of compound **23**

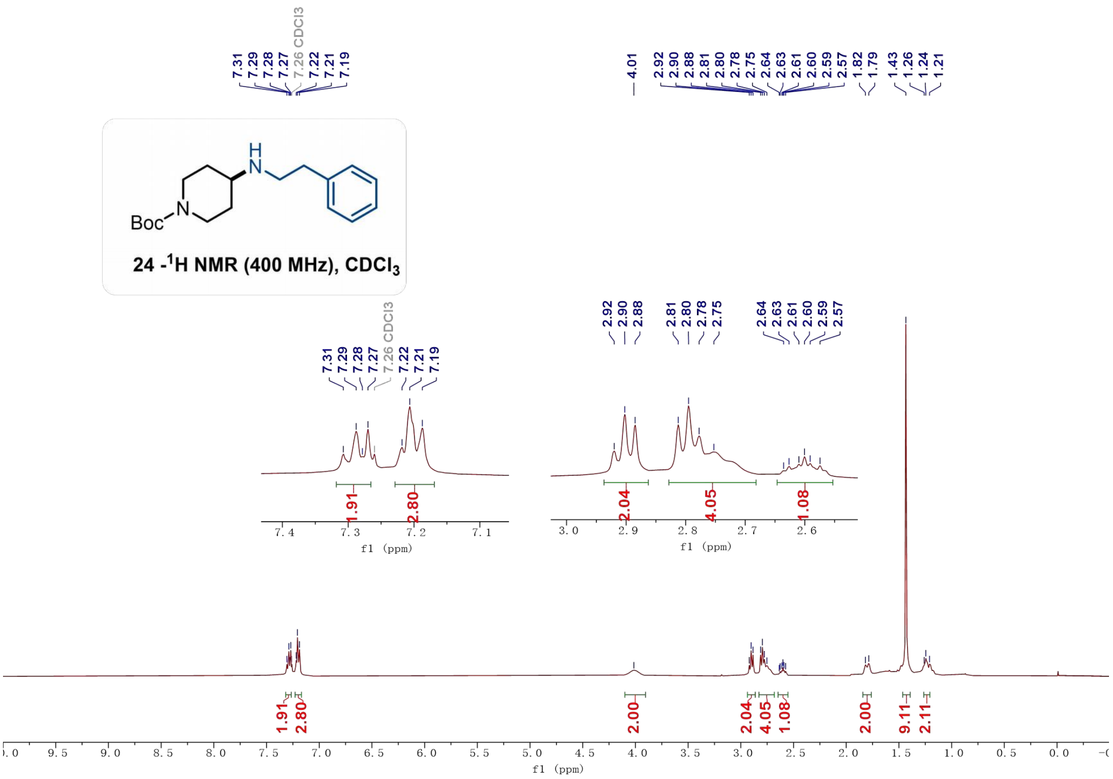

Supplementary Figure 99. ^{1}H NMR(400 MHz, $CDCl_3$) spectrum of compound **24**

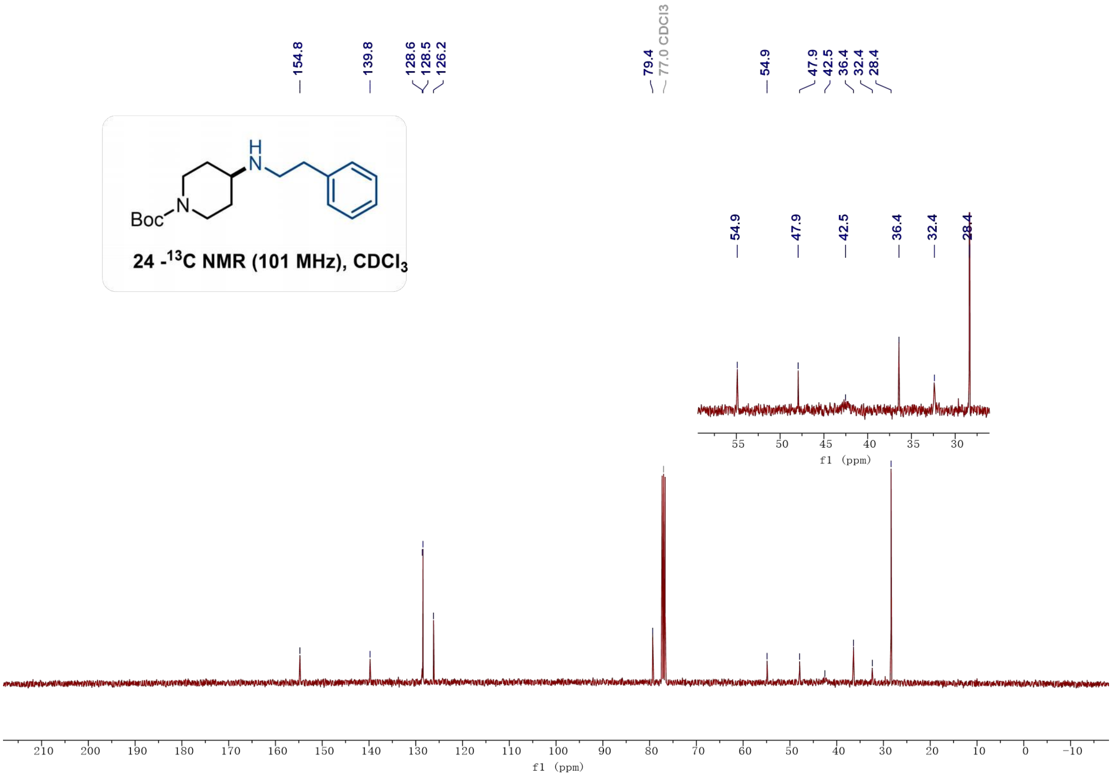

Supplementary Figure 100. ^{13}C NMR (101 MHz, $CDCl_3$) spectrum of compound **24**

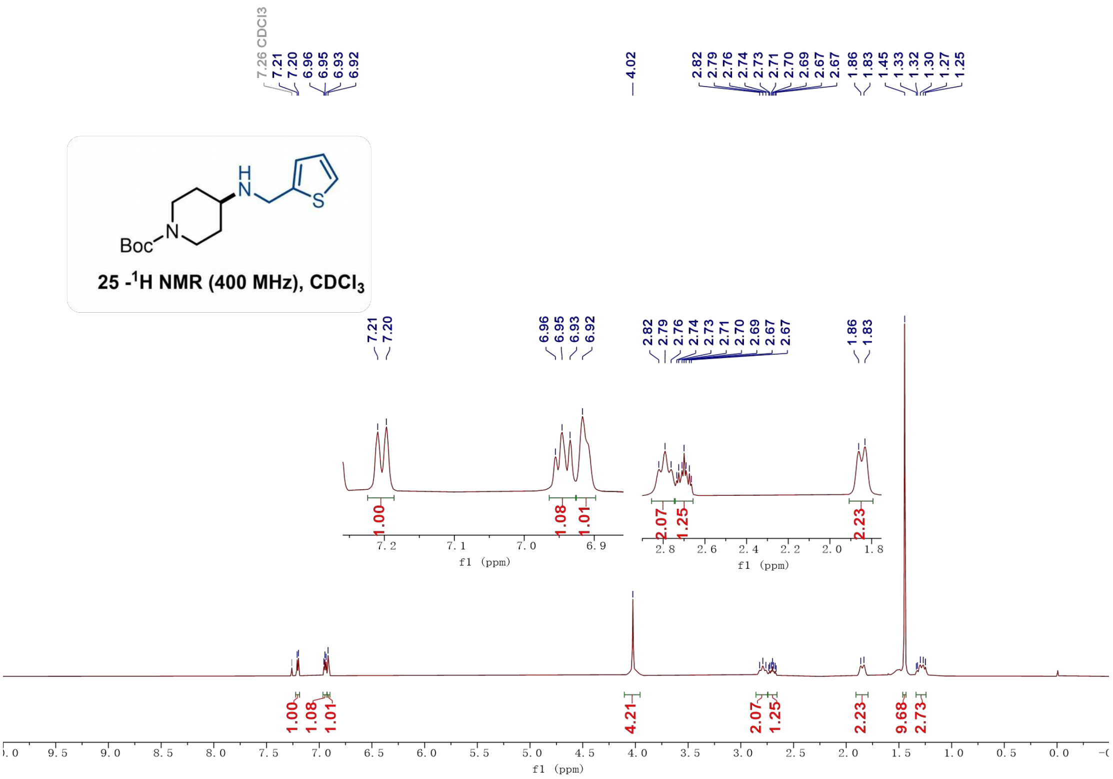

Supplementary Figure 101. ^{1}H NMR(400 MHz, $CDCl_3$) spectrum of compound **25**

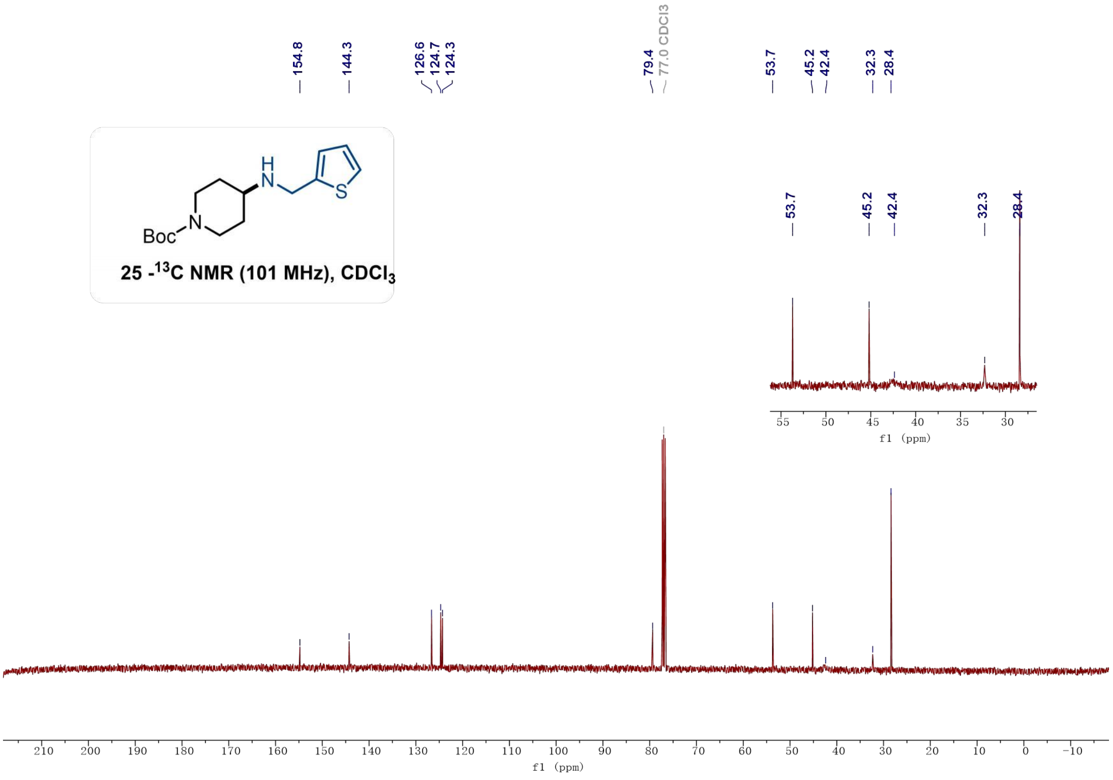

Supplementary Figure 102. ^{13}C NMR (101 MHz, $CDCl_3$) spectrum of compound **25**

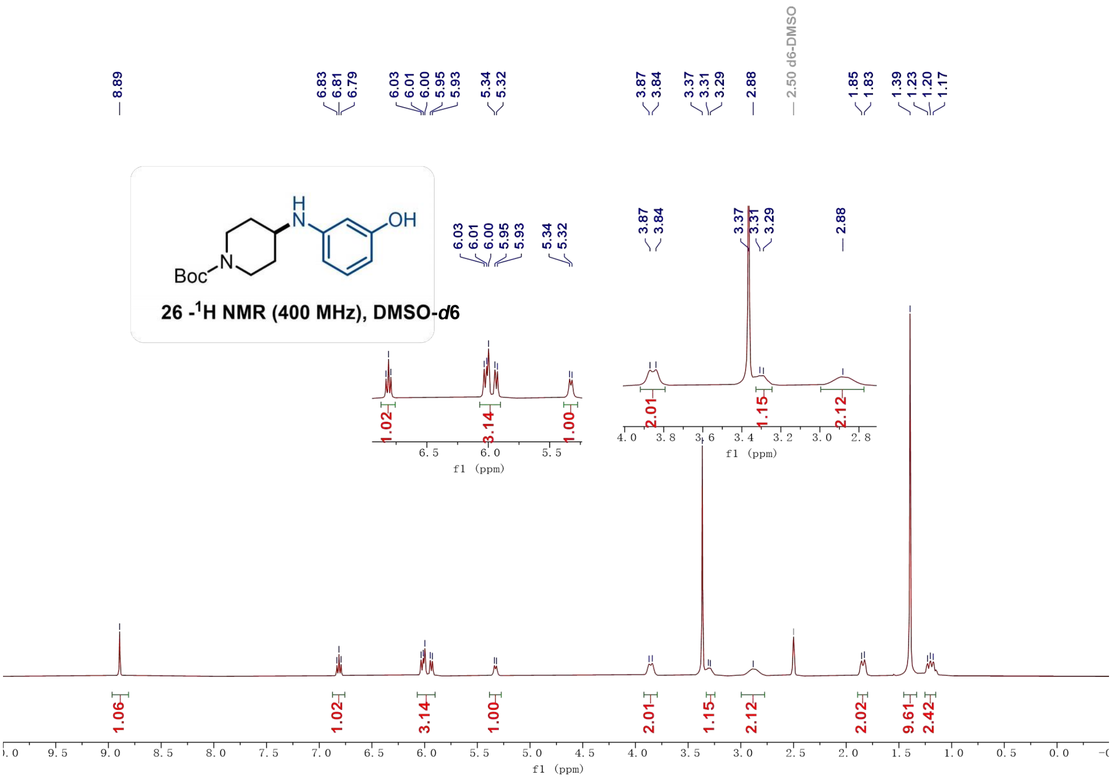

Supplementary Figure 103. ^{1}H NMR (400 MHz, $CDCl_3$) spectrum of compound **26**

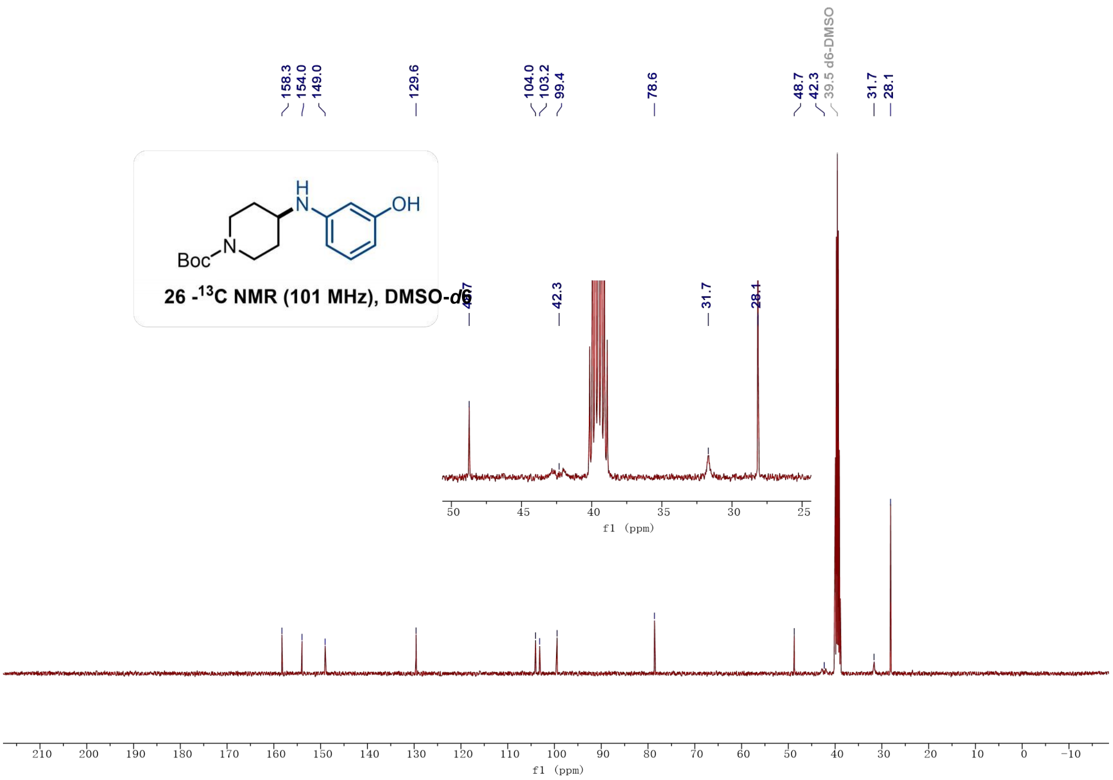

Supplementary Figure 104. ^{13}C NMR (101 MHz, $CDCl_3$) spectrum of compound **26**

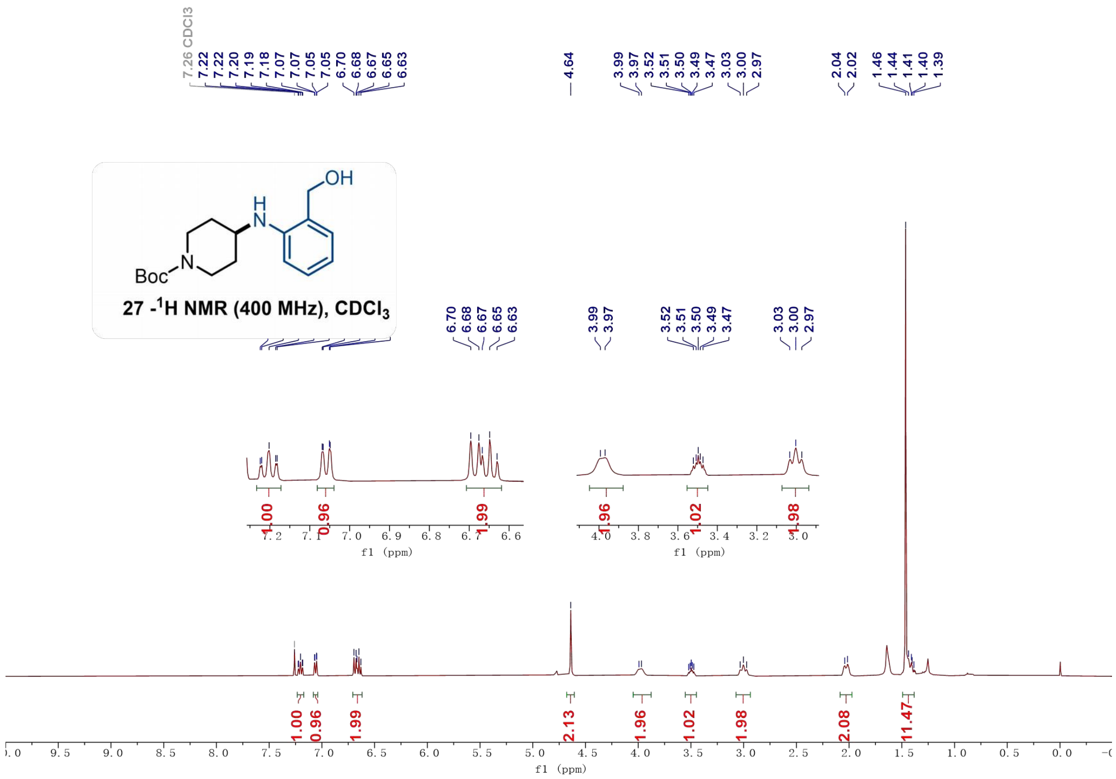

Supplementary Figure 105. ^{1}H NMR (400 MHz, $CDCl_3$) spectrum of compound **27**

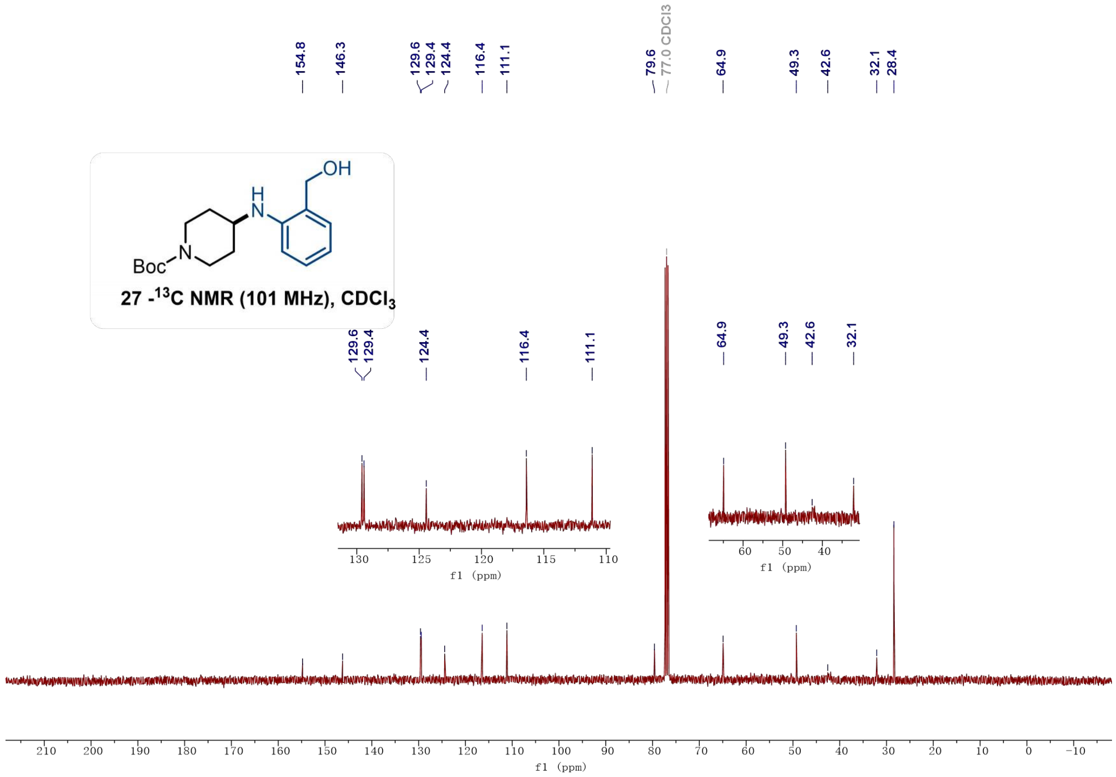

Supplementary Figure 106. ^{13}C NMR (101 MHz, $CDCl_3$) spectrum of compound **27**

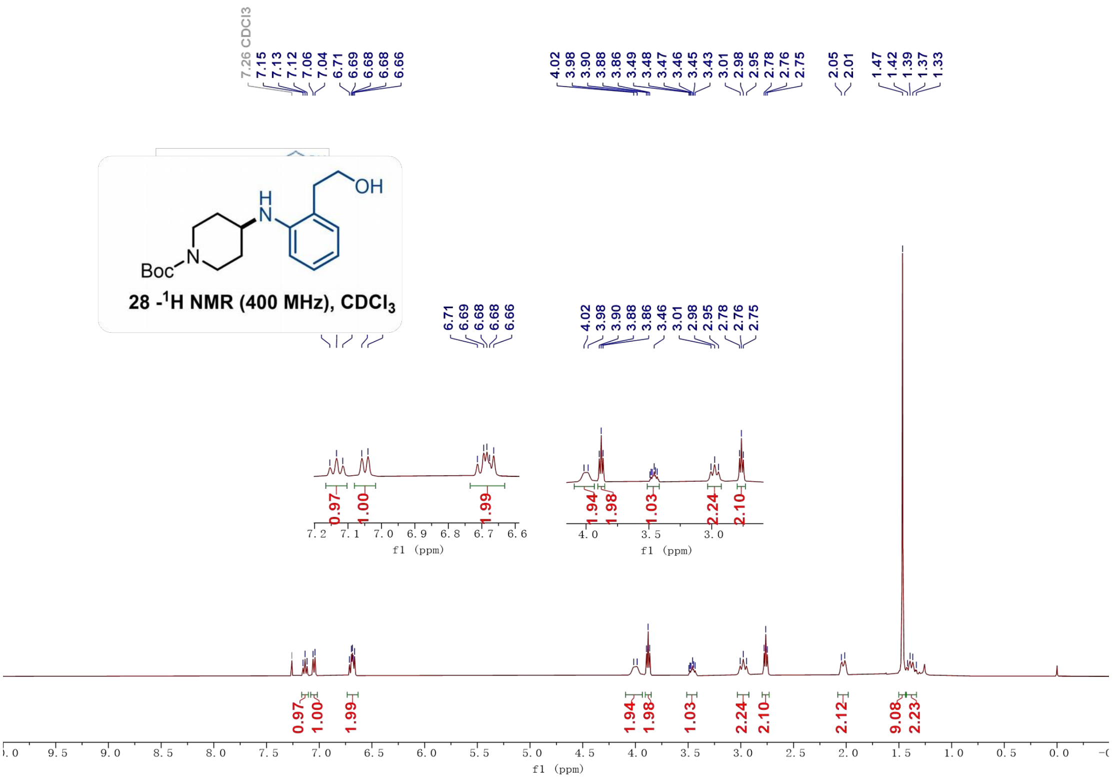

Supplementary Figure 107. ^{1}H NMR (400 MHz, $CDCl_3$) spectrum of compound **28**

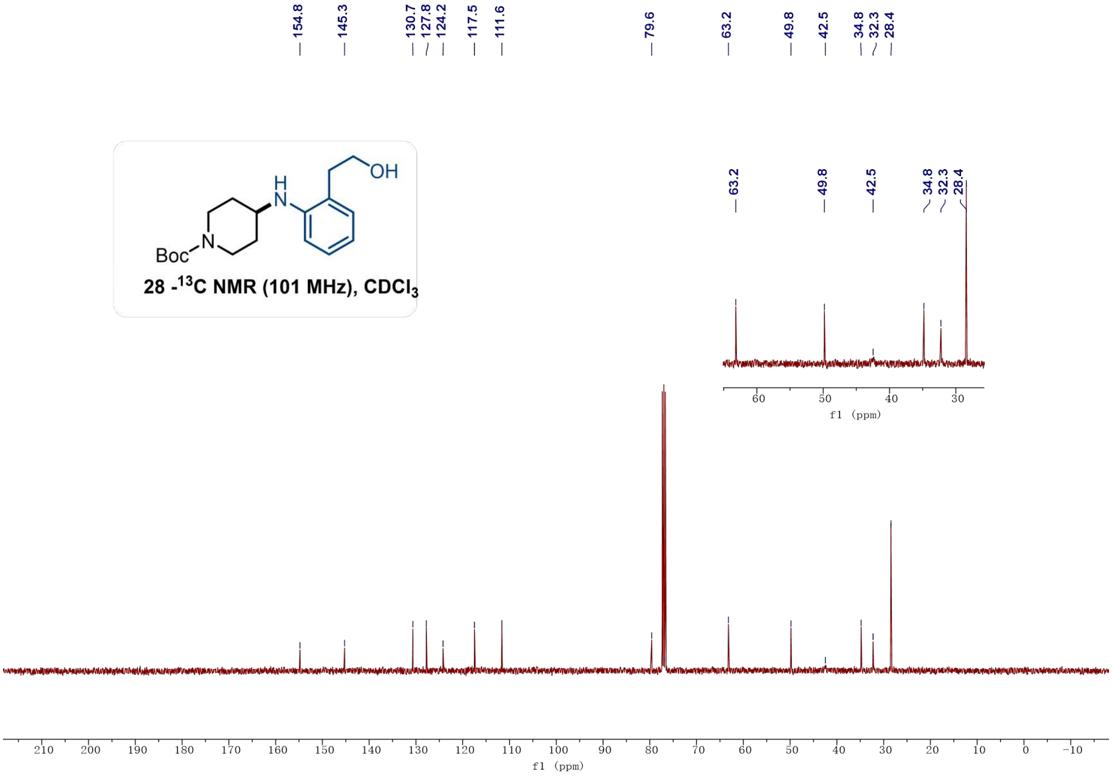

Supplementary Figure 108. ^{13}C NMR (101 MHz, $CDCl_3$) spectrum of compound **28**

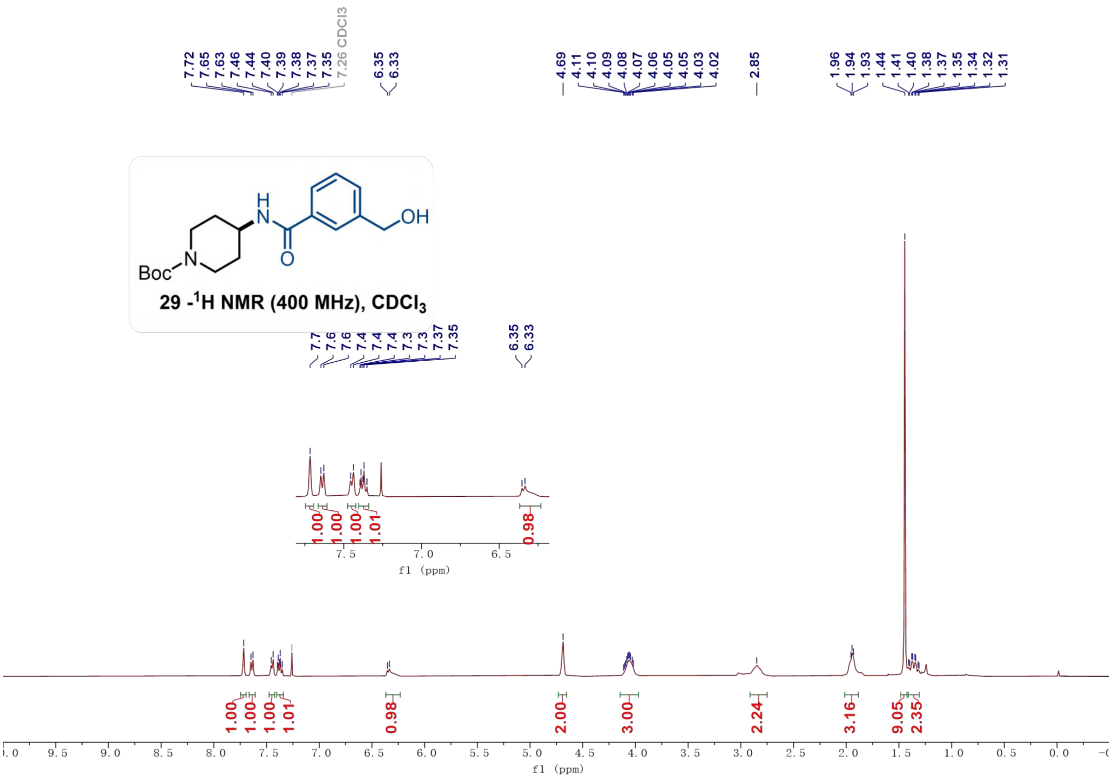

Supplementary Figure 109. ^{1}H NMR (400 MHz, $CDCl_3$) spectrum of compound **29**

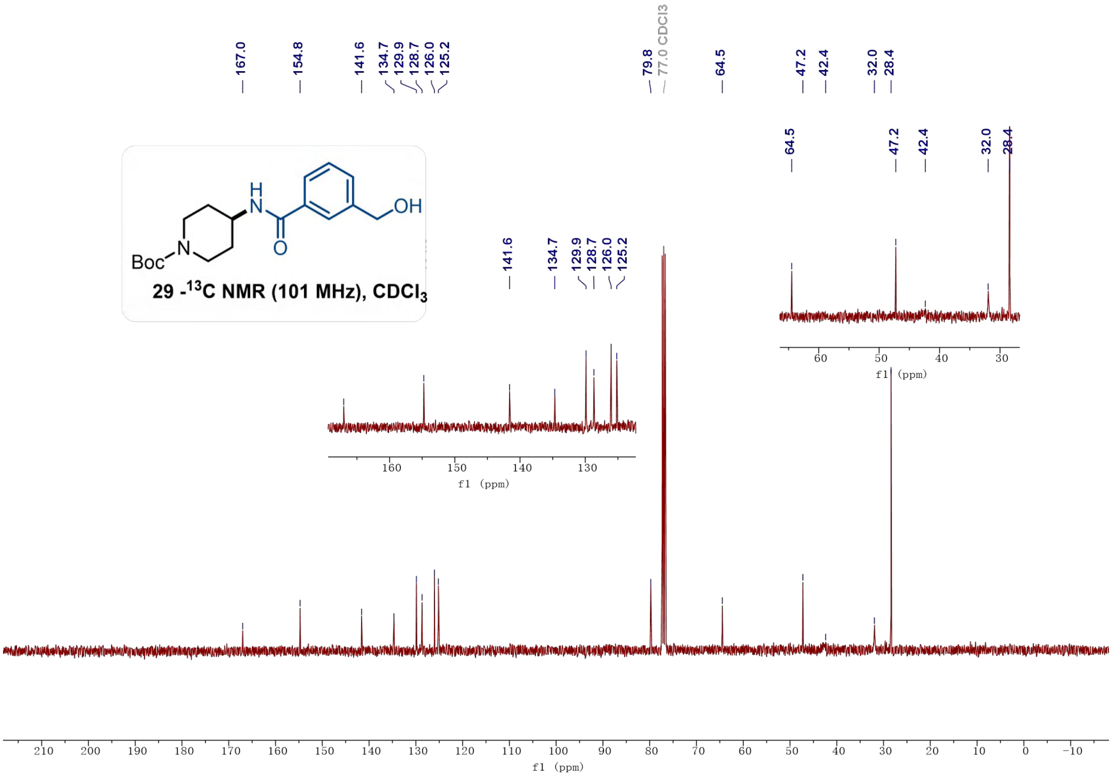

Supplementary Figure 110. ^{13}C NMR (101 MHz, $CDCl_3$) spectrum of compound **29**

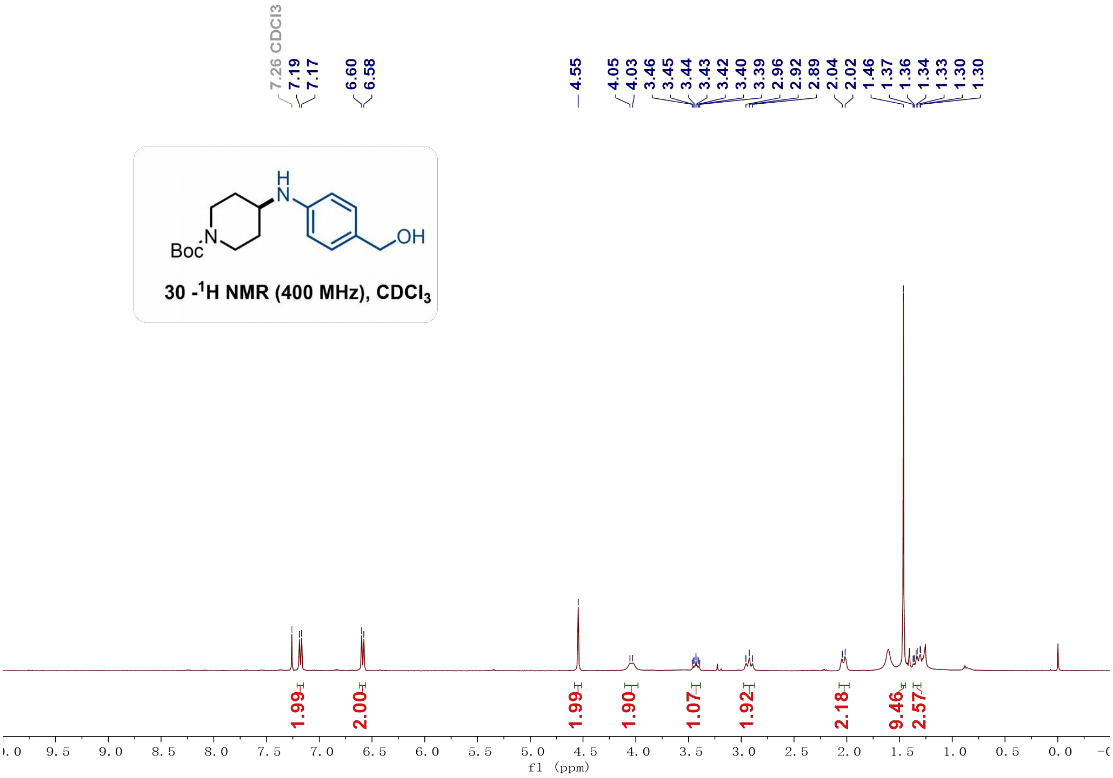

Supplementary Figure 111. ^{1}H NMR(400 MHz, $CDCl_3$) spectrum of compound **30**

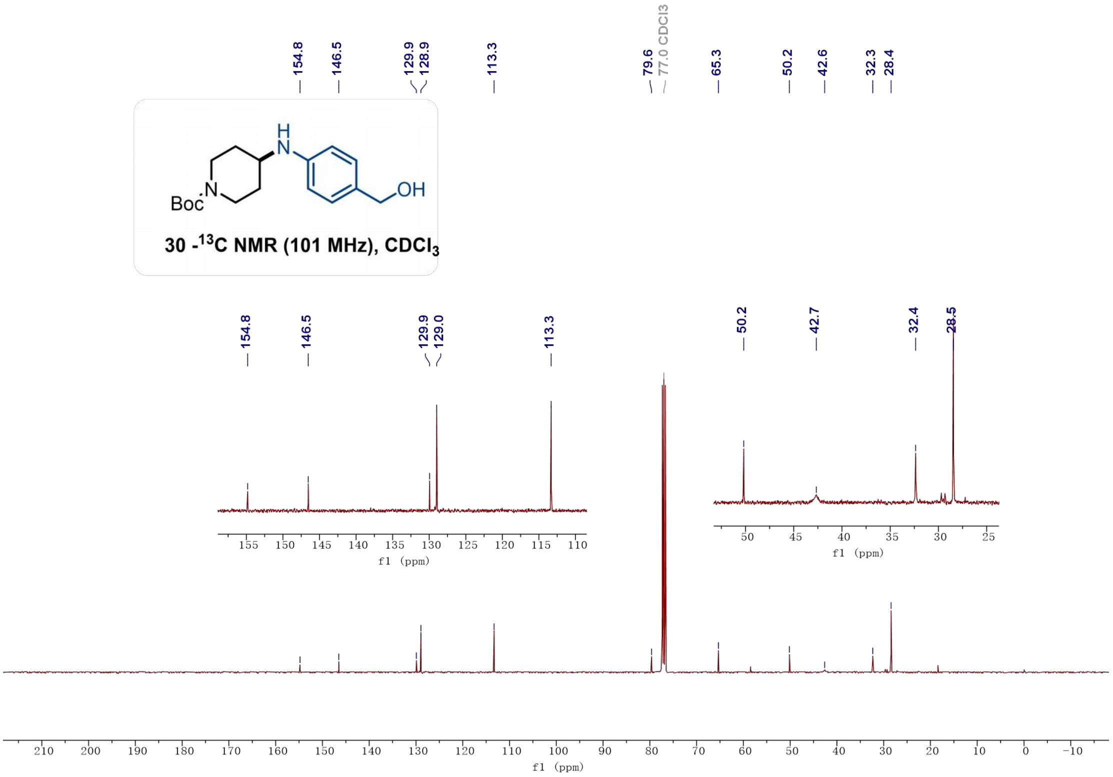

Supplementary Figure 112. ^{13}C NMR (101 MHz, $CDCl_3$) spectrum of compound **30**

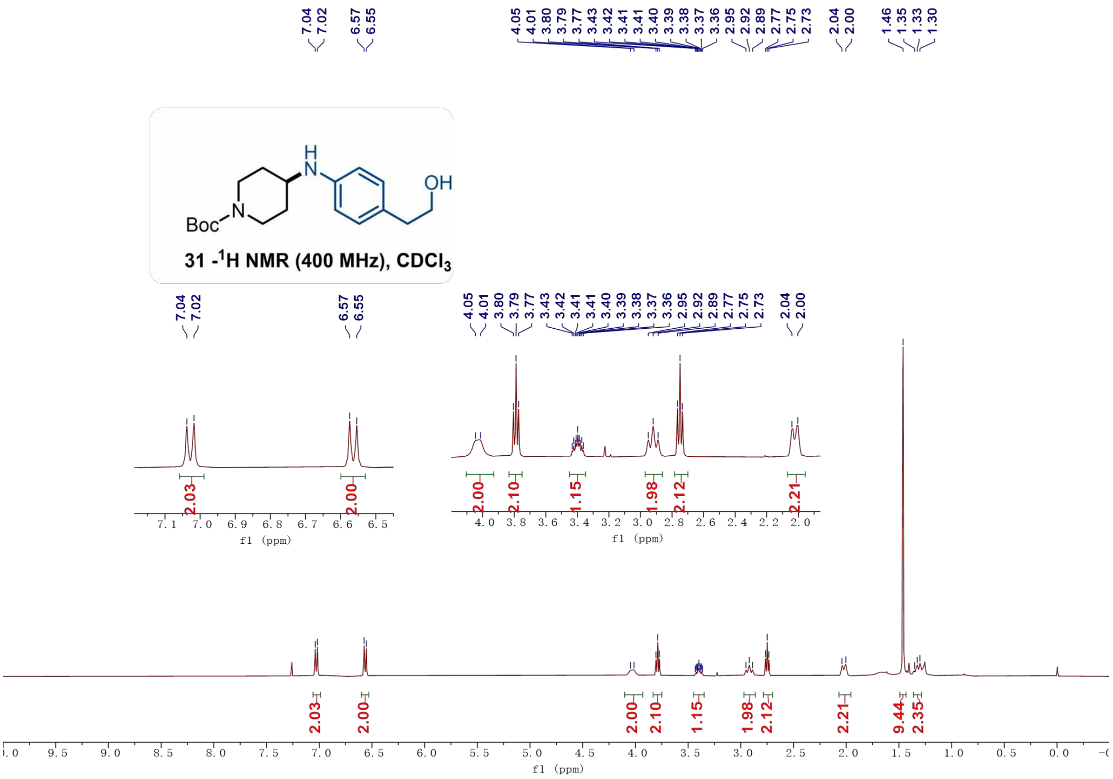

Supplementary Figure 113. ^{1}H NMR (400 MHz, $CDCl_3$) spectrum of compound **31**

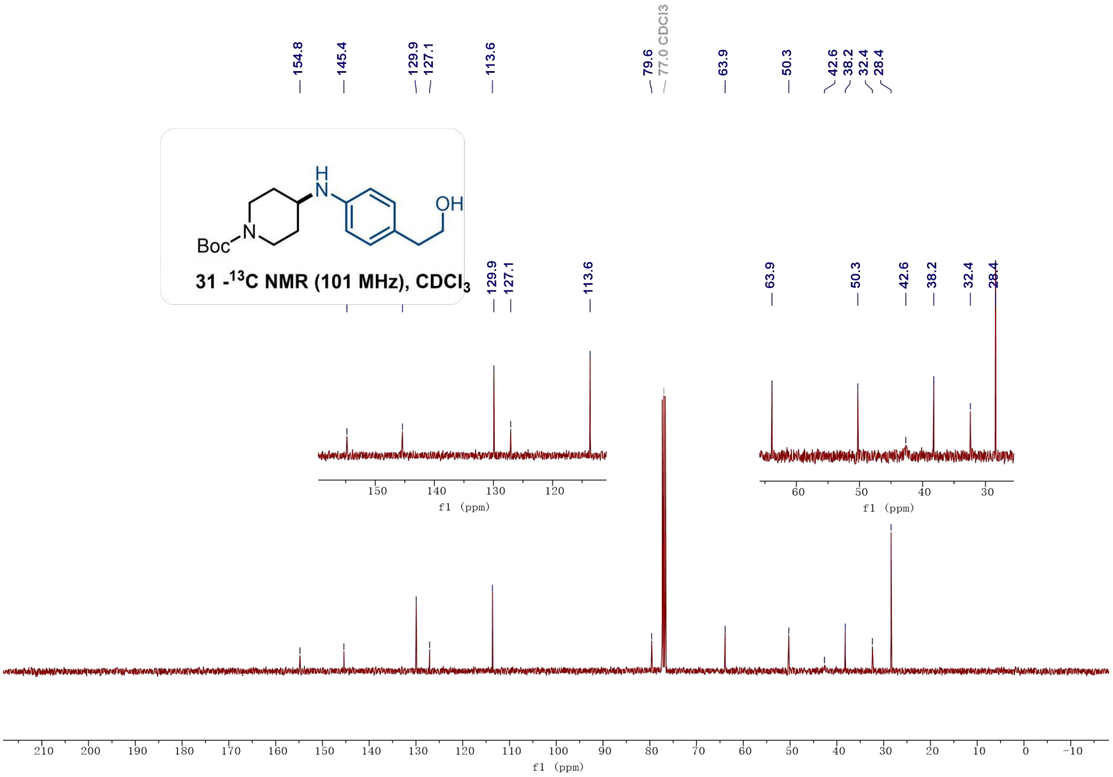

Supplementary Figure 114. ^{13}C NMR (101 MHz, $CDCl_3$) spectrum of compound **31**

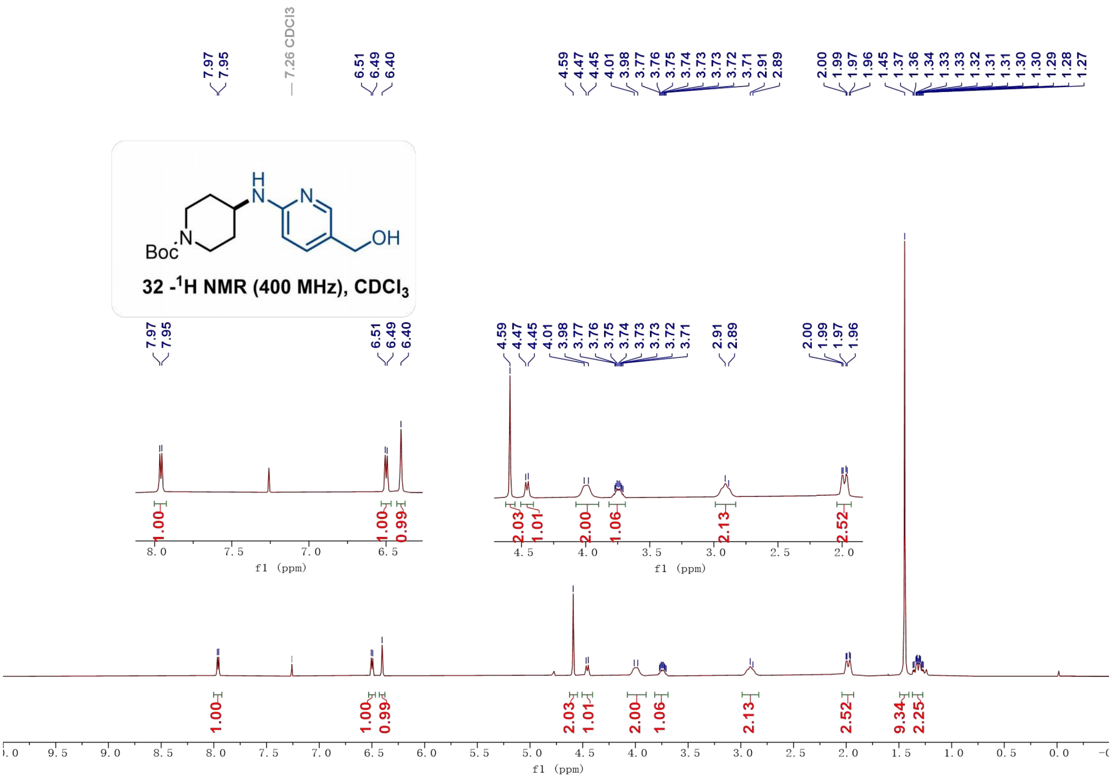

Supplementary Figure 115. ^{1}H NMR (400 MHz, $CDCl_3$) spectrum of compound **32**

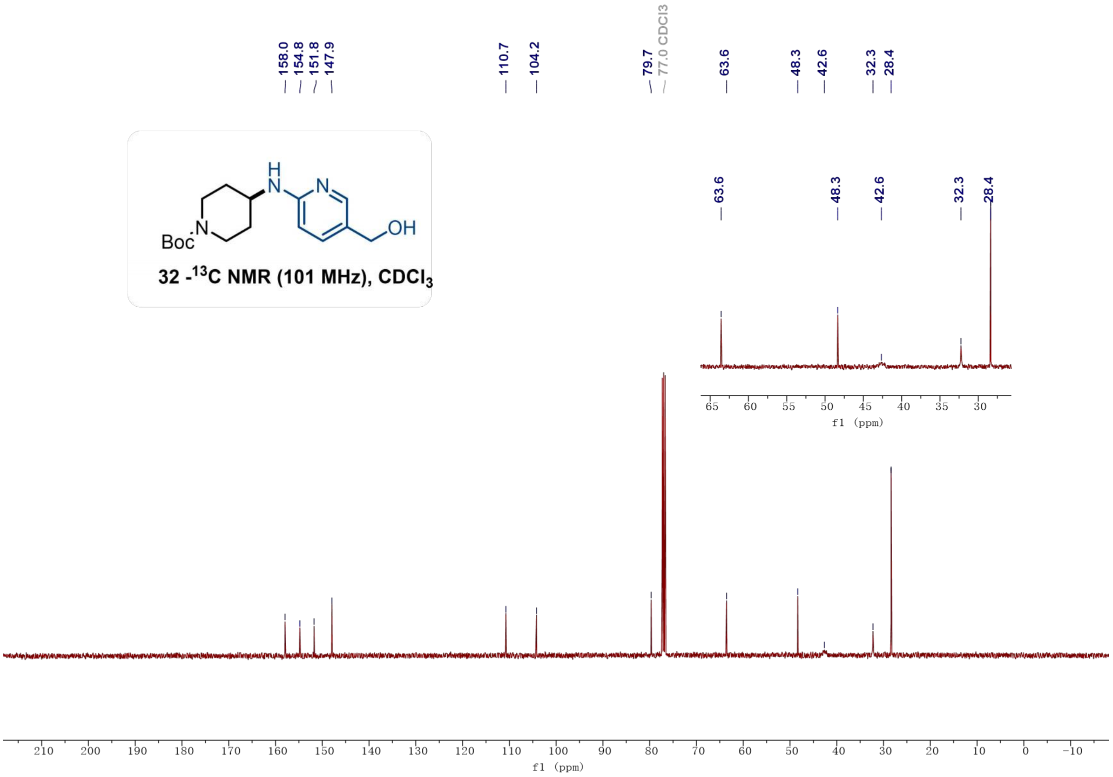

Supplementary Figure 116. ^{13}C NMR (101 MHz, $CDCl_3$) spectrum of compound **32**

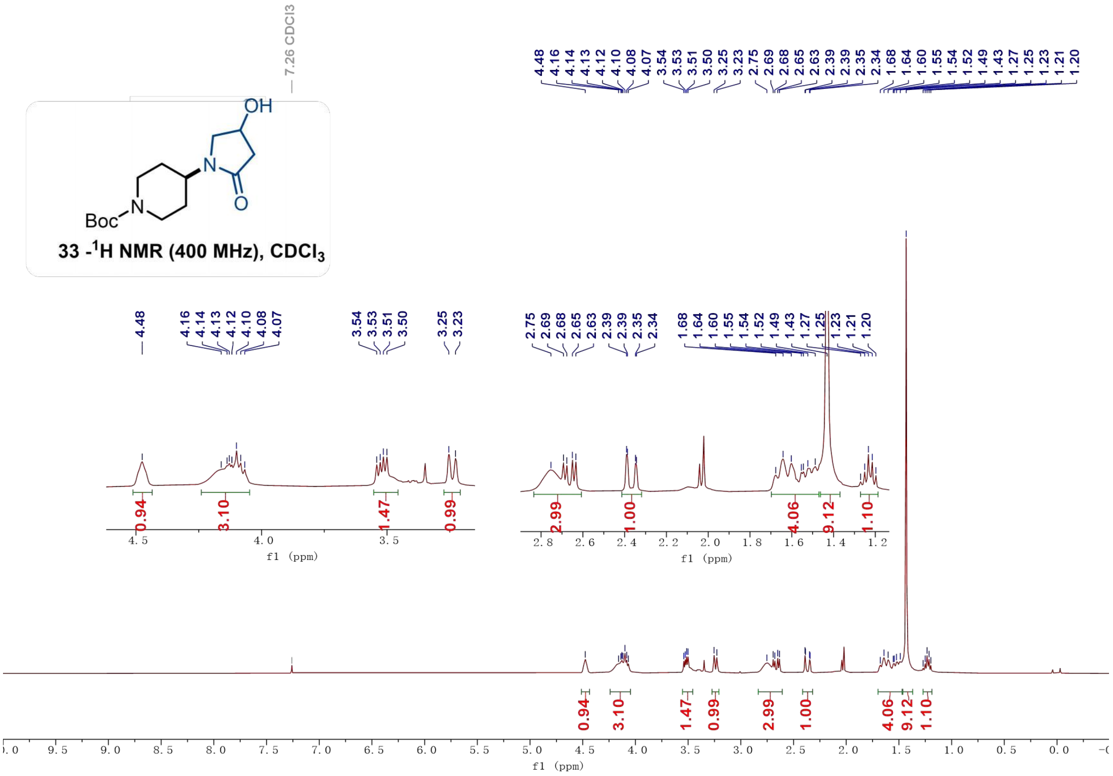

Supplementary Figure 117. ^{1}H NMR (400 MHz, $CDCl_3$) spectrum of compound **33**

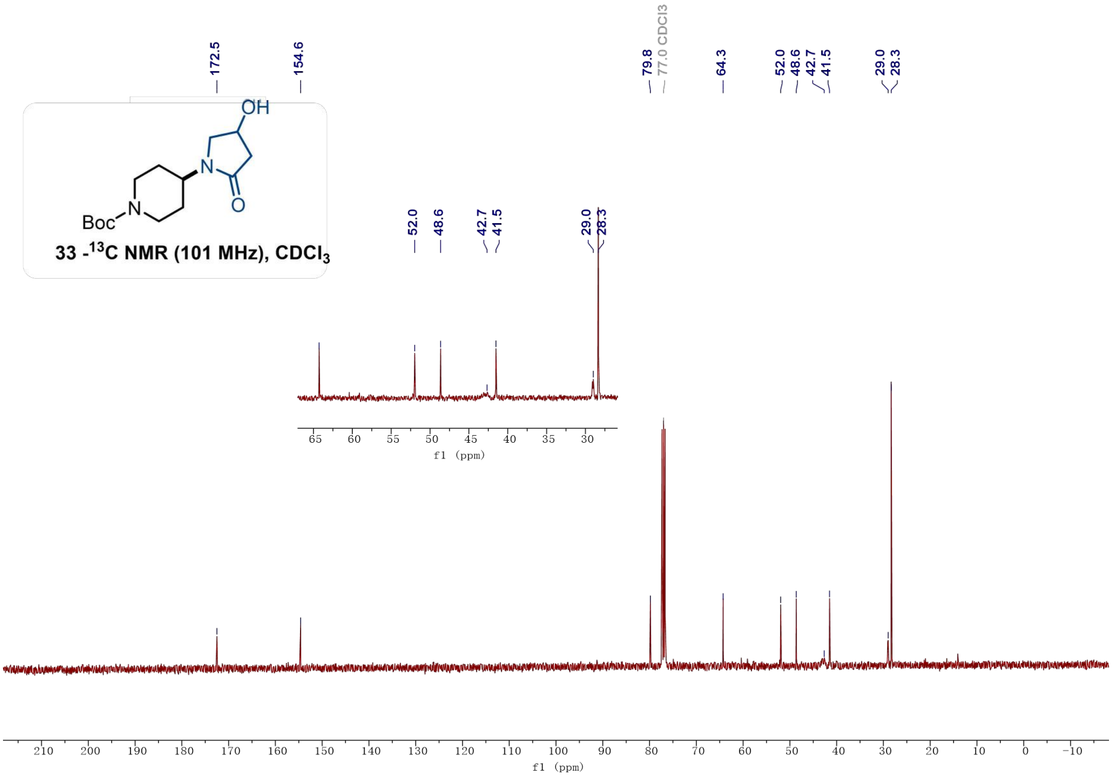

Supplementary Figure 118. ^{13}C NMR (101 MHz, $CDCl_3$) spectrum of compound **33**

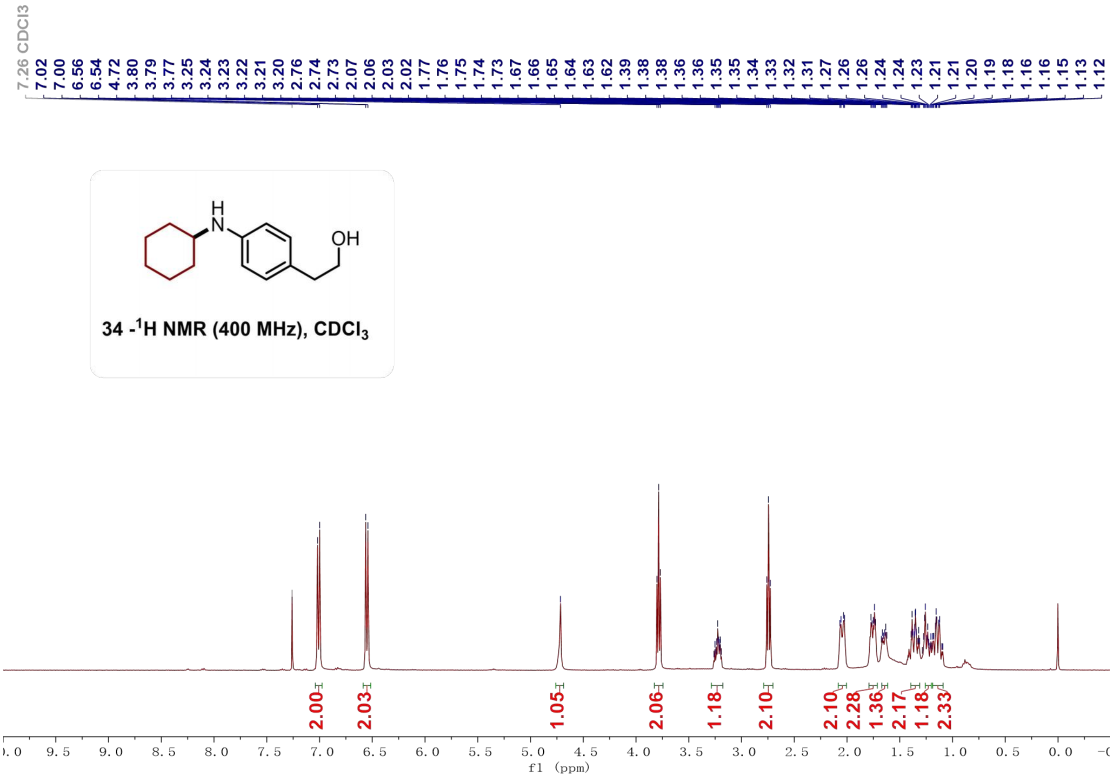

Supplementary Figure 119. ^{1}H NMR (400 MHz, $CDCl_3$) spectrum of compound **34**

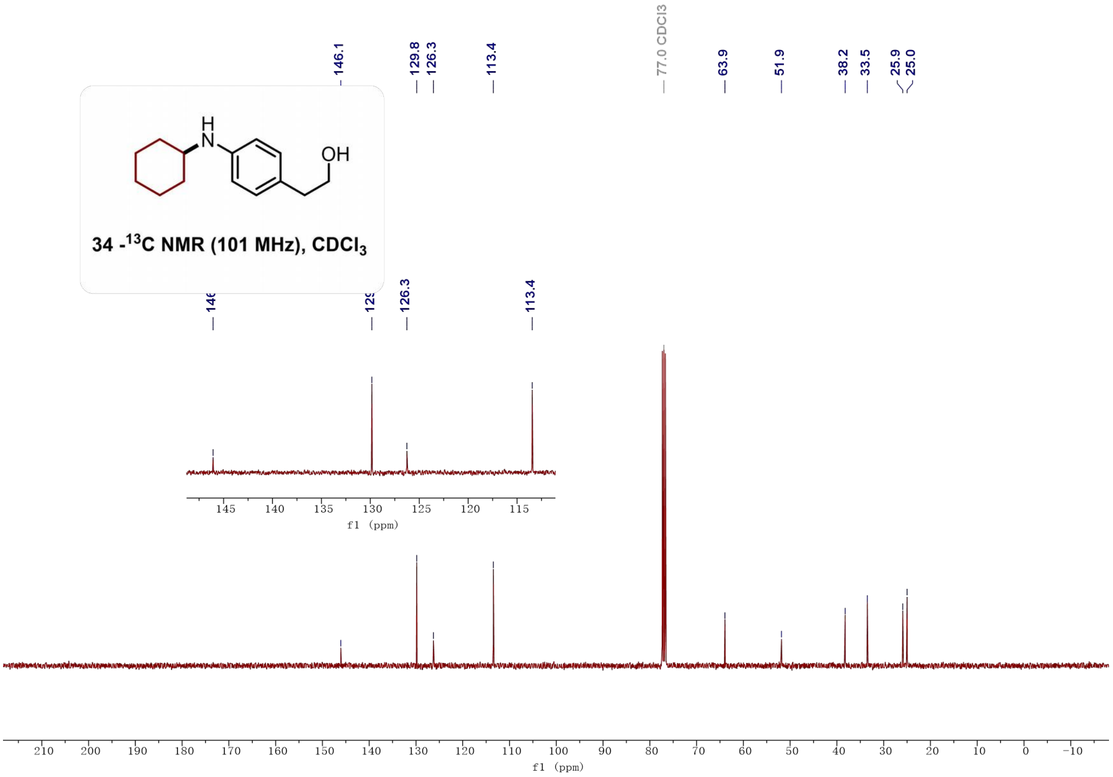

Supplementary Figure 120. ^{13}C NMR (101 MHz, $CDCl_3$) spectrum of compound **34**

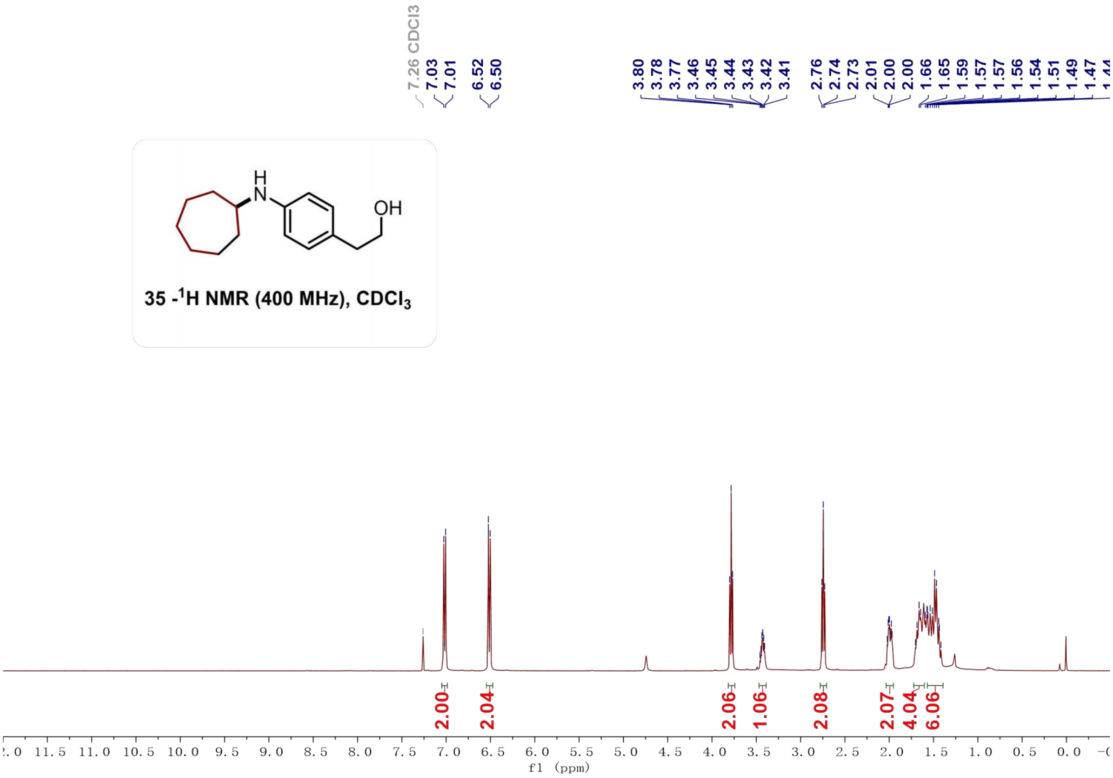

Supplementary Figure 121. ^{1}H NMR(400 MHz, $CDCl_3$) spectrum of compound **35**

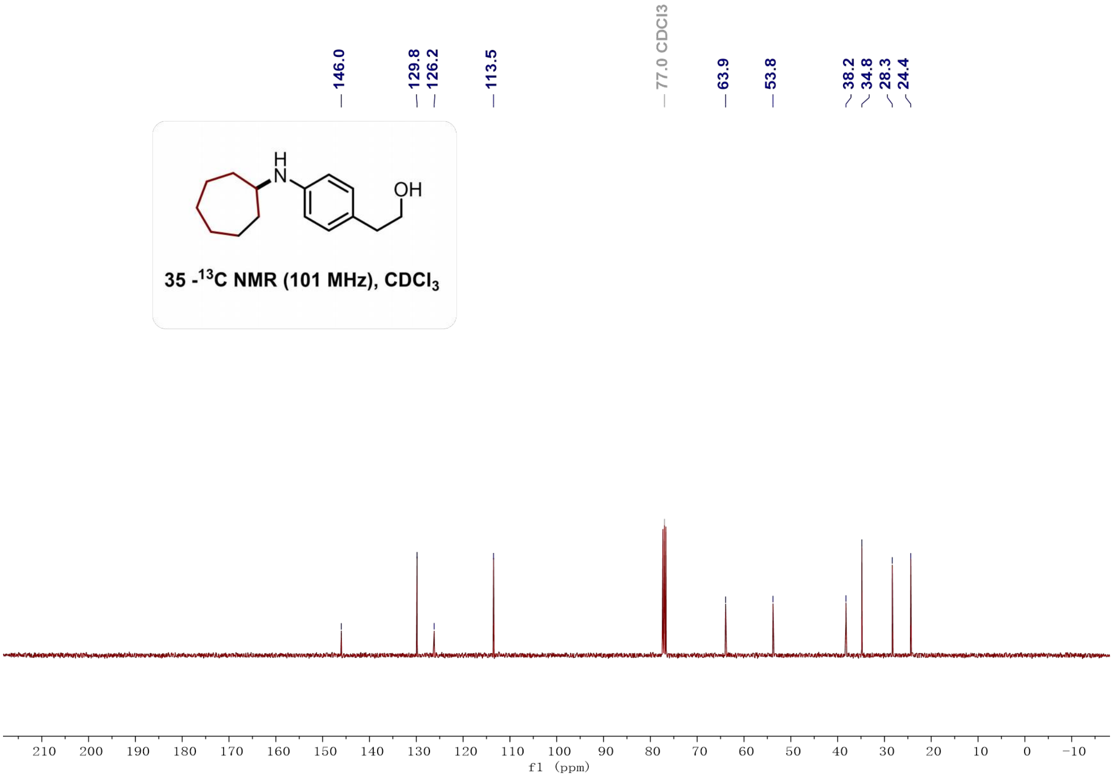

Supplementary Figure 122. ^{13}C NMR (101 MHz, $CDCl_3$) spectrum of compound **35**

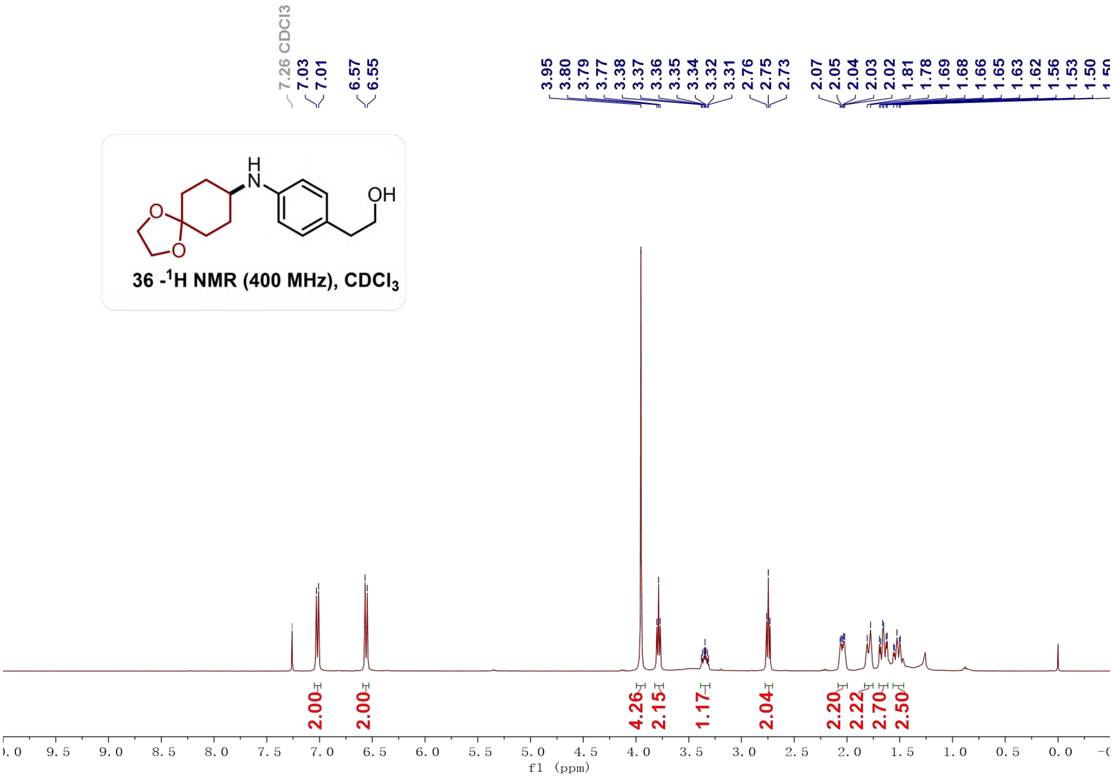

Supplementary Figure 123. ^{1}H NMR (400 MHz, $CDCl_3$) spectrum of compound **36**

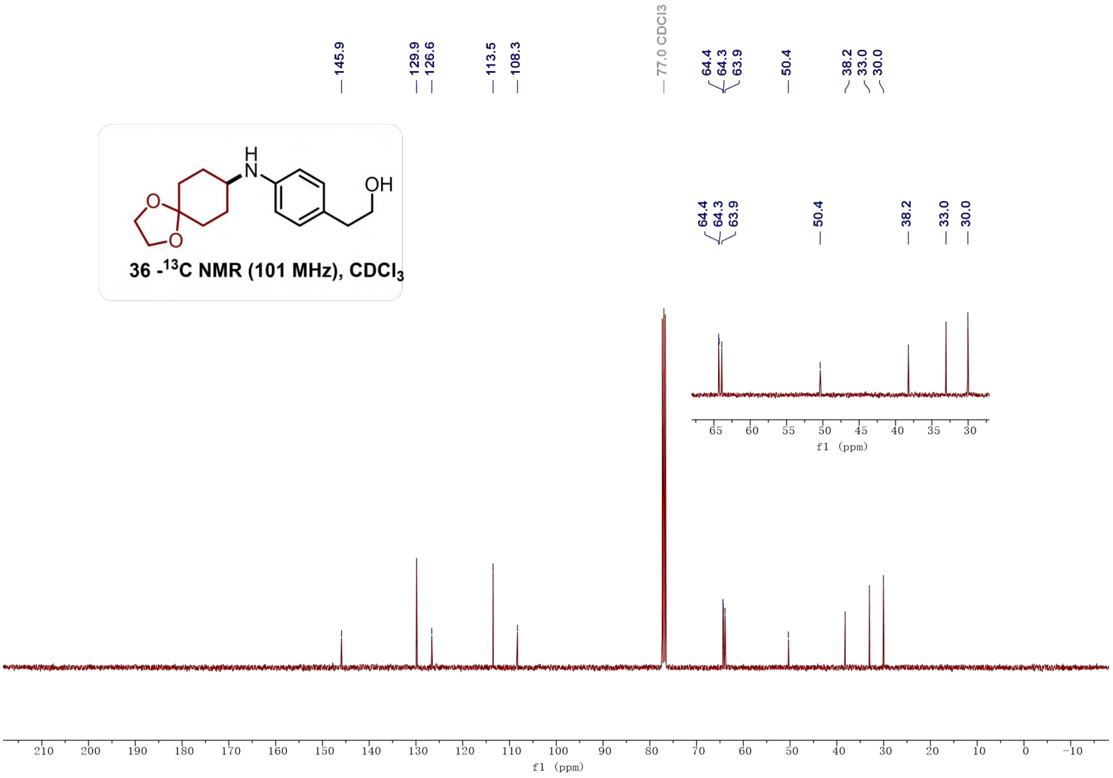

Supplementary Figure 124. ^{13}C NMR (101 MHz, $CDCl_3$) spectrum of compound **36**

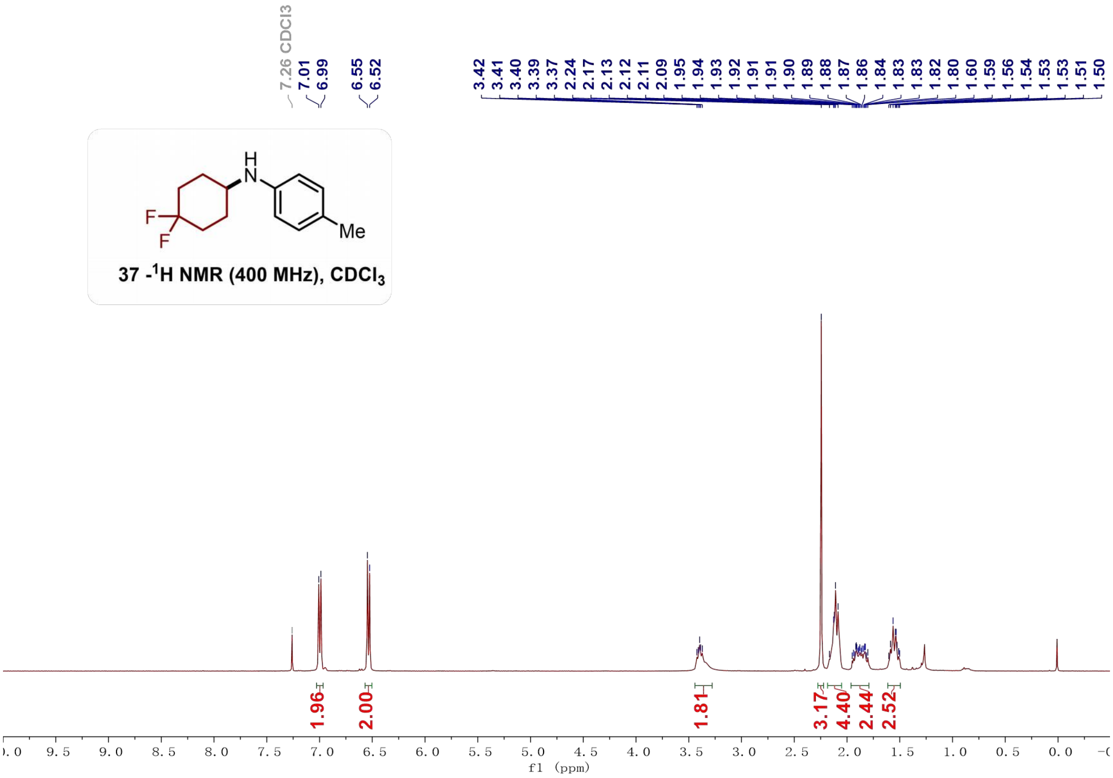

Supplementary Figure 125. ^{1}H NMR (400 MHz, $CDCl_3$) spectrum of compound **37**

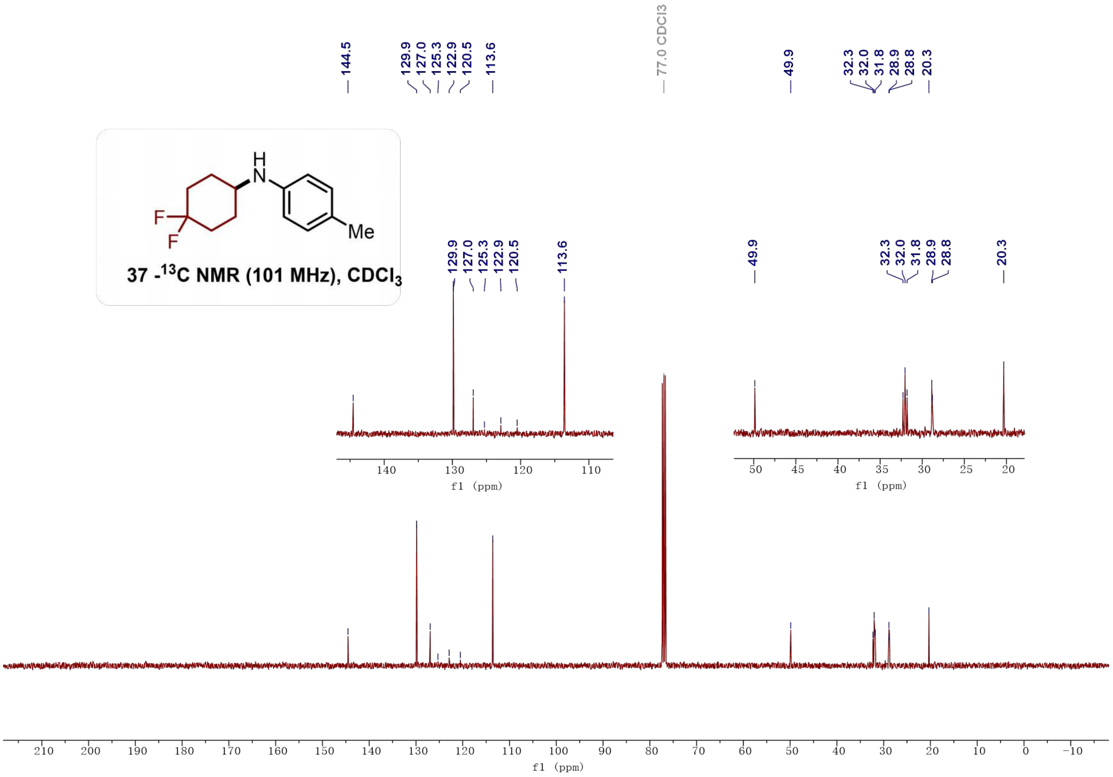

Supplementary Figure 126. ^{13}C NMR (101 MHz, $CDCl_3$) spectrum of compound **37**

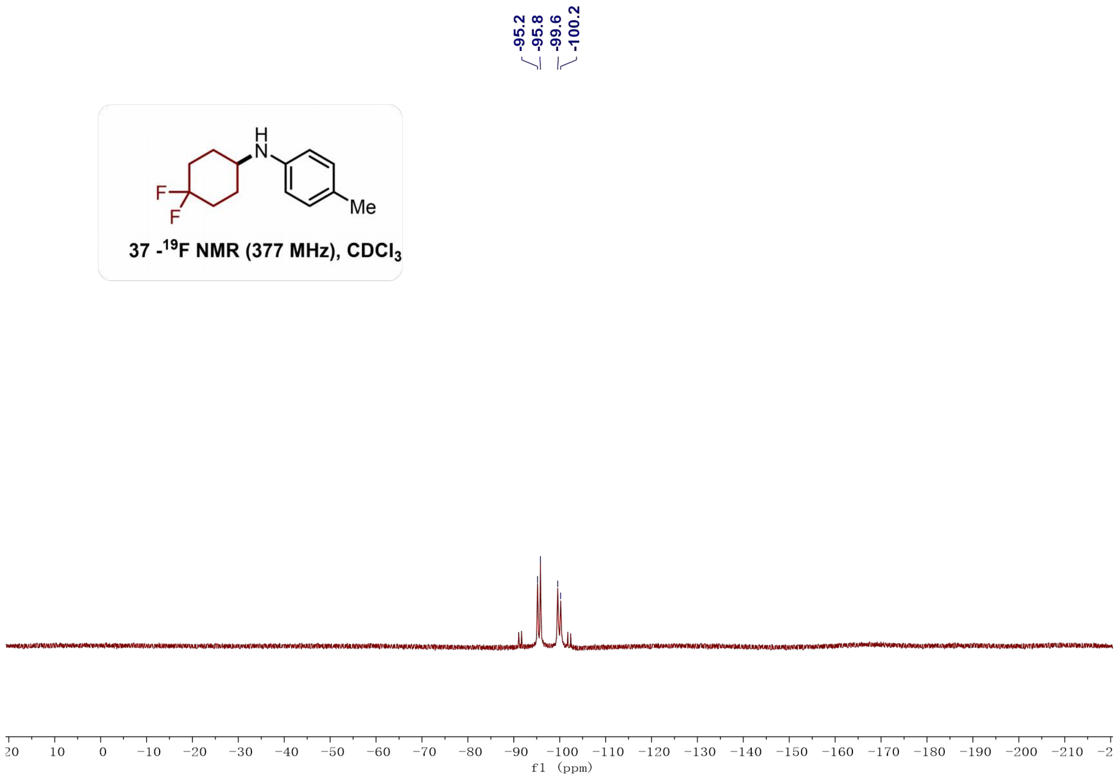

Supplementary Figure 127. ^{19}F NMR (377 MHz, $CDCl_3$) spectrum of compound **37**

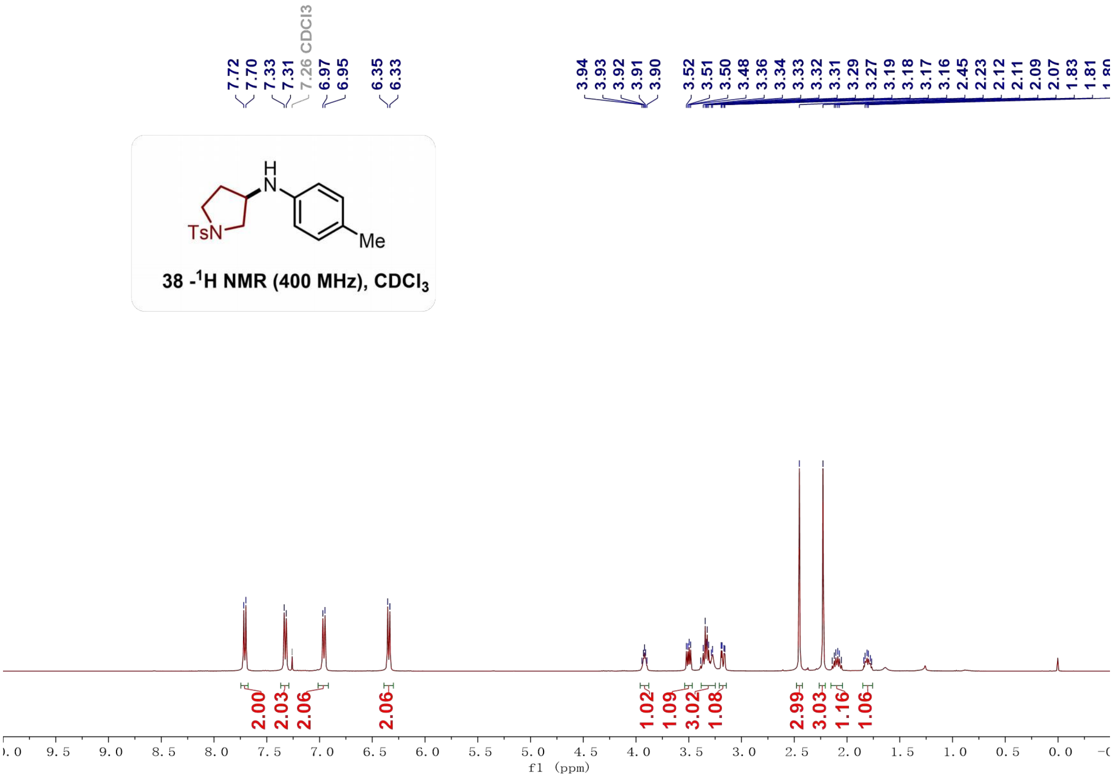

Supplementary Figure 128. ^{1}H NMR (400 MHz, $CDCl_3$) spectrum of compound **38**

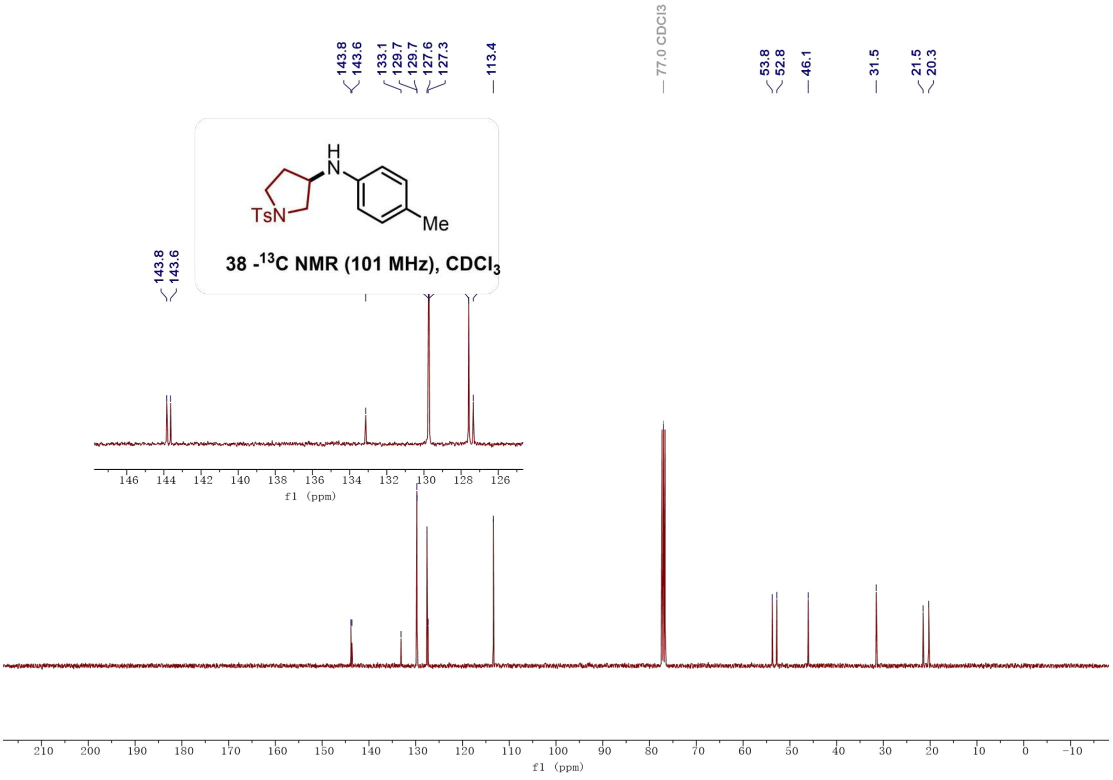

Supplementary Figure 129. ^{13}C NMR (101 MHz, $CDCl_3$) spectrum of compound **38**

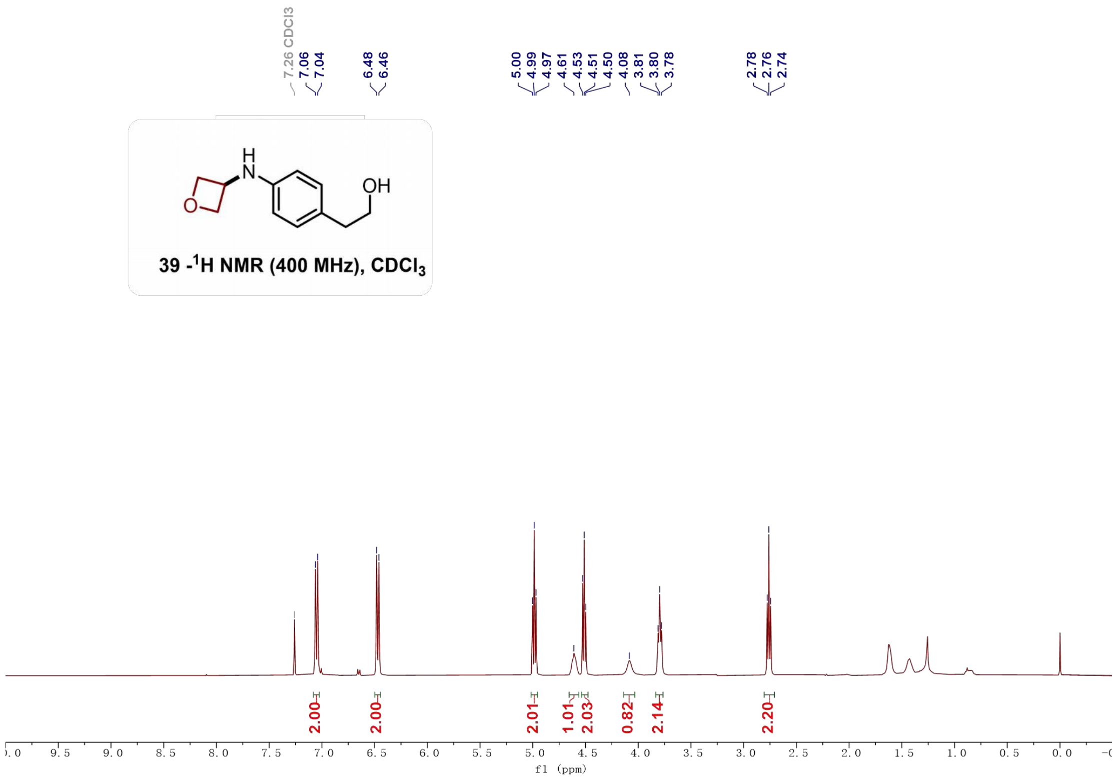

Supplementary Figure 130. ^{1}H NMR (400 MHz, $CDCl_3$) spectrum of compound **39**

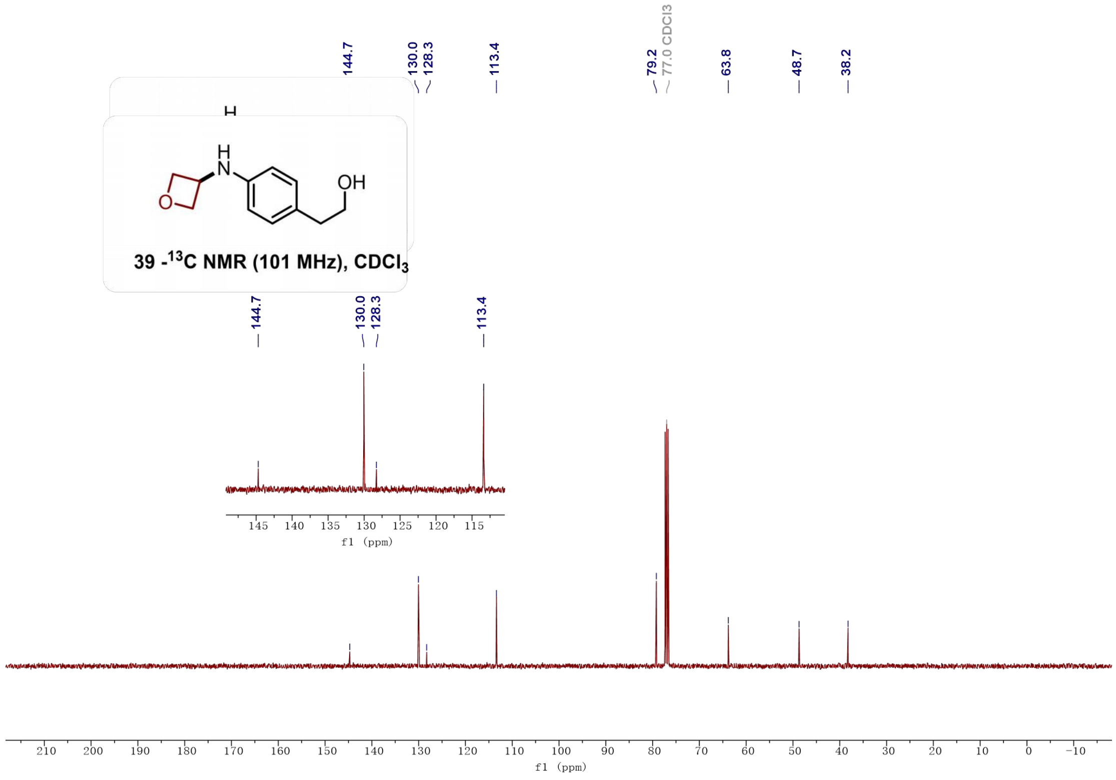

Supplementary Figure 131. ^{13}C NMR (101 MHz, $CDCl_3$) spectrum of compound **39**

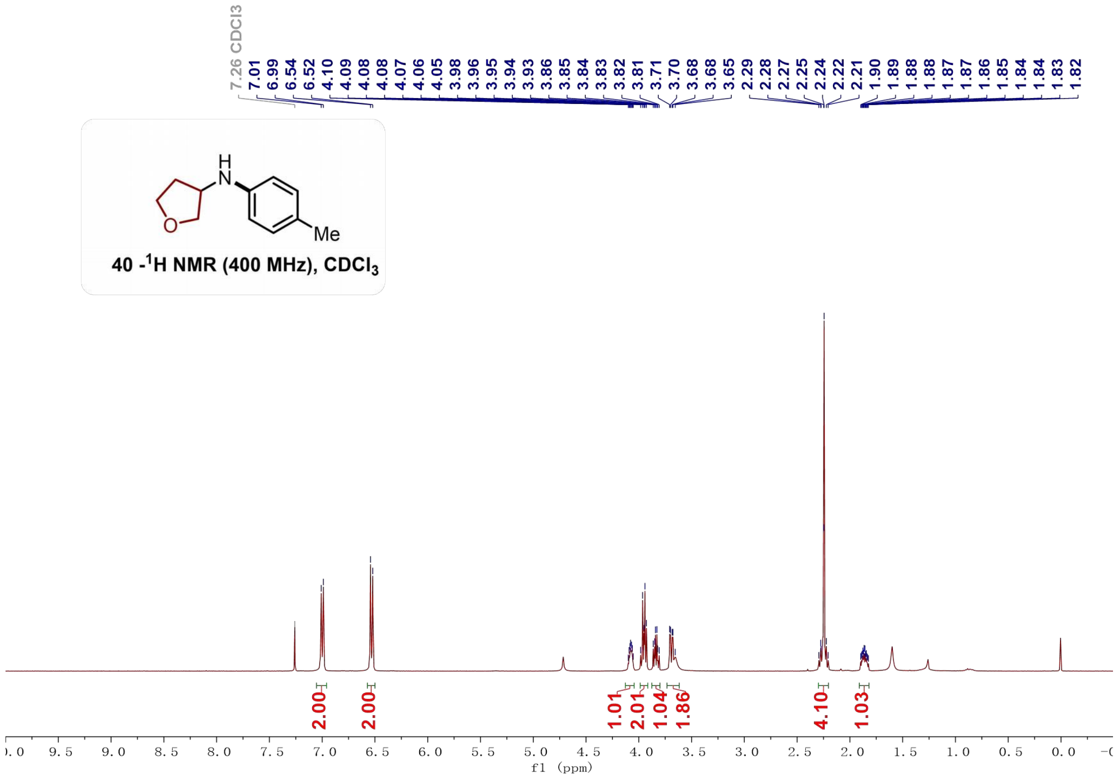

Supplementary Figure 132. ^{1}H NMR (400 MHz, $CDCl_3$) spectrum of compound **40**

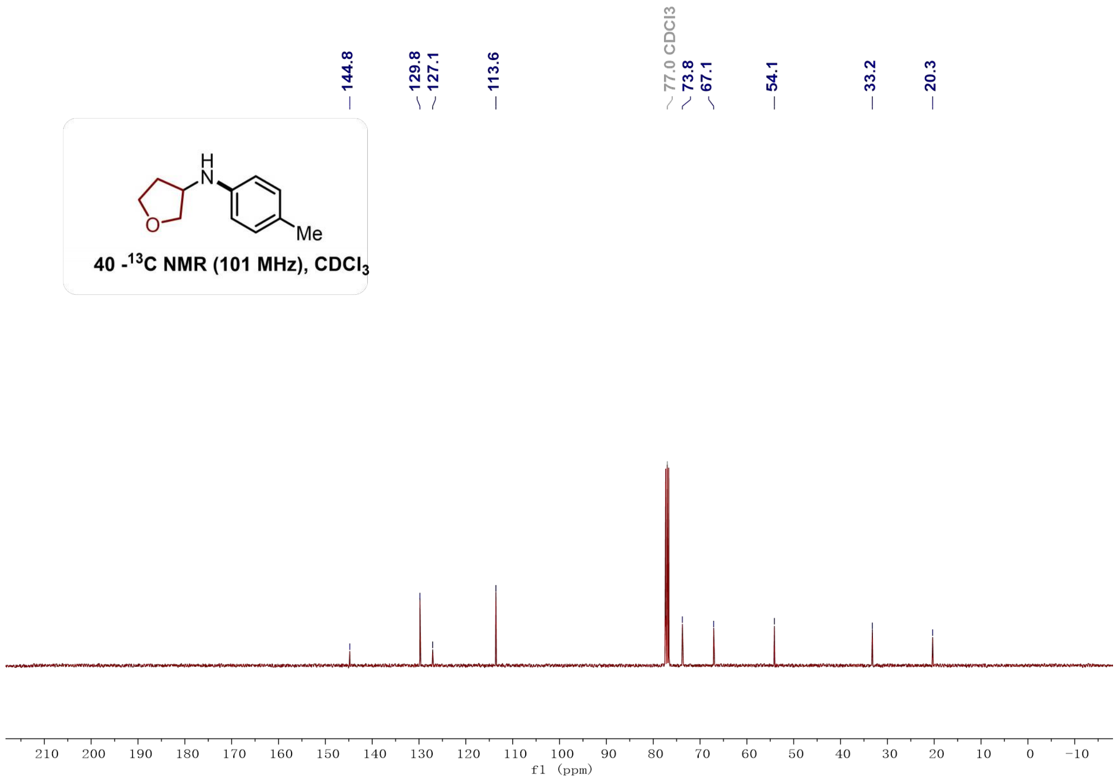

Supplementary Figure 133. ^{13}C NMR (101 MHz, $CDCl_3$) spectrum of compound **40**

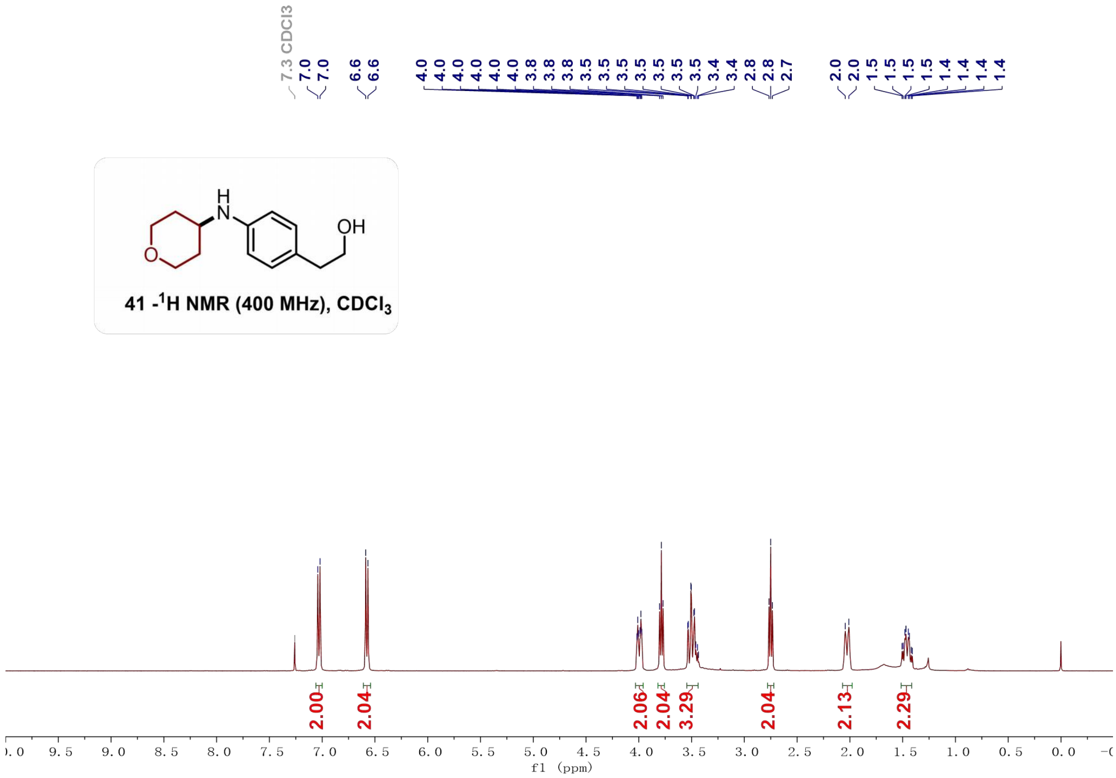

Supplementary Figure 134. ^{1}H NMR (400 MHz, $CDCl_3$) spectrum of compound **41**

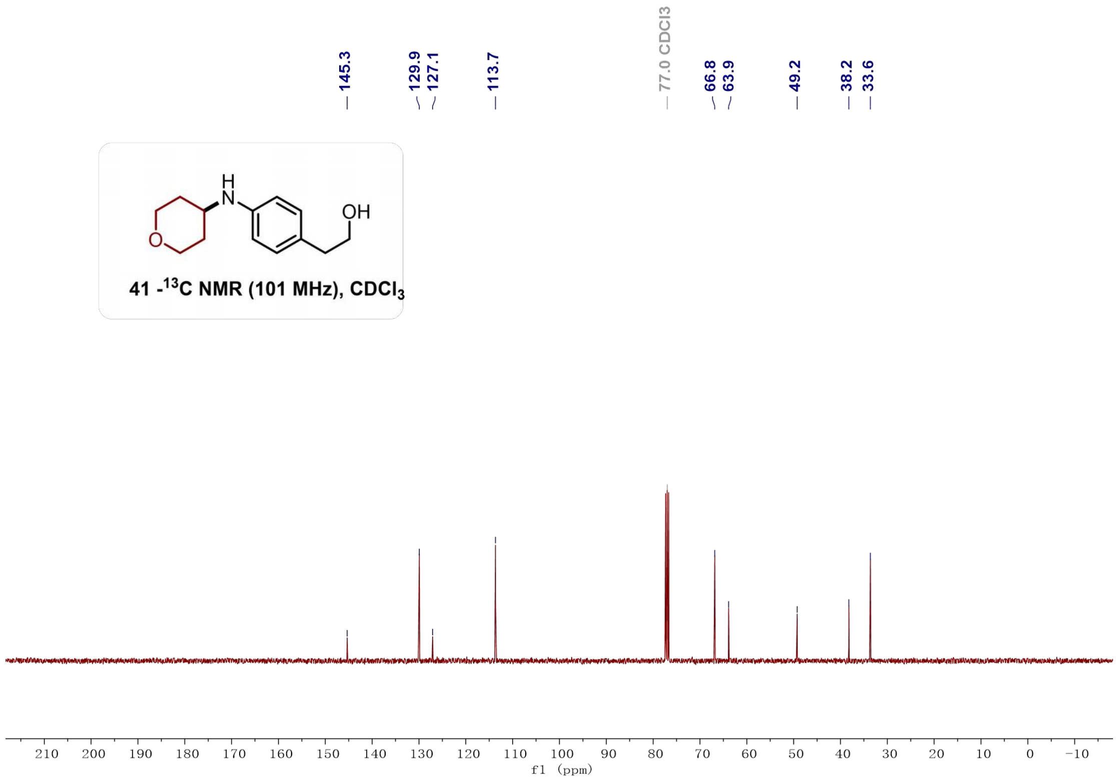

Supplementary Figure 135. ^{13}C NMR (101 MHz, $CDCl_3$) spectrum of compound **41**

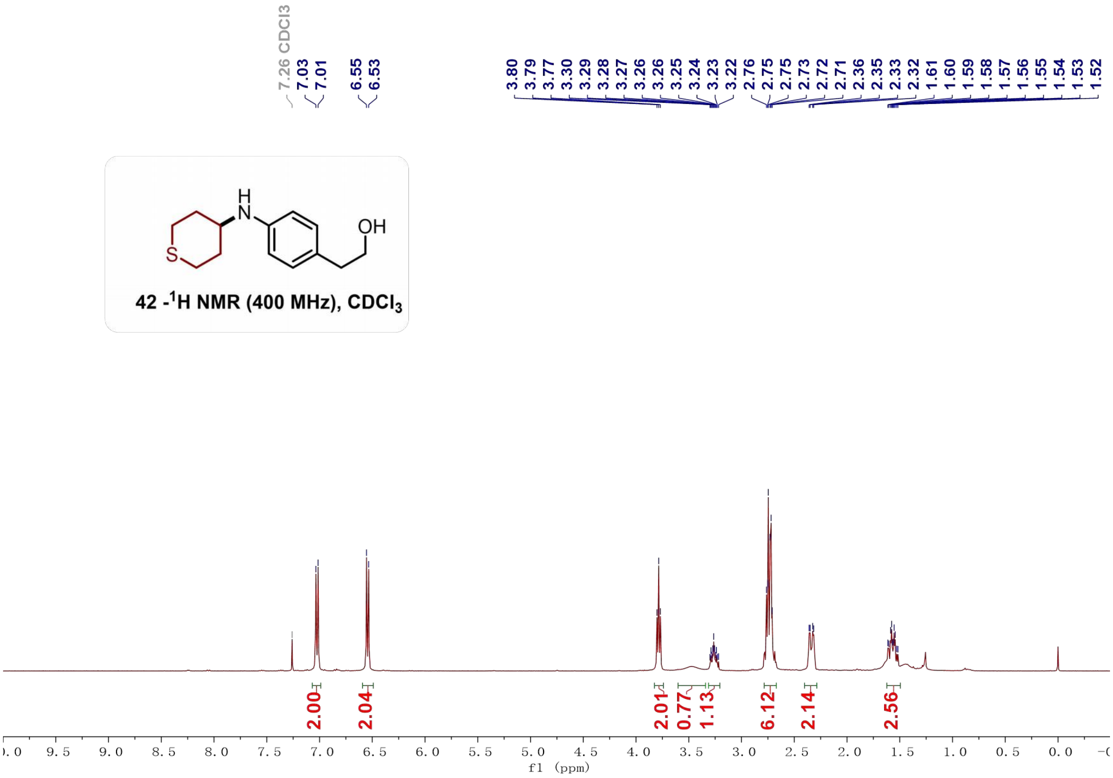

Supplementary Figure 136. ^{1}H NMR (400 MHz, $CDCl_3$) spectrum of compound **42**

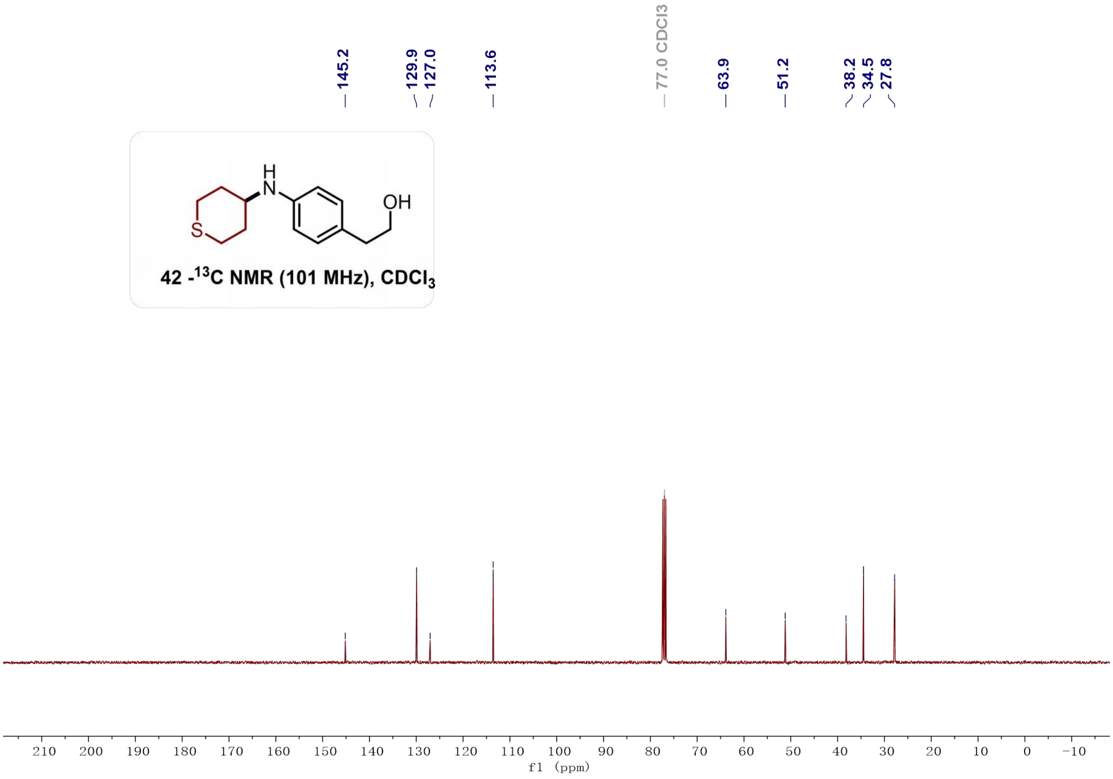

Supplementary Figure 137. ^{13}C NMR (101 MHz, $CDCl_3$) spectrum of compound **42**

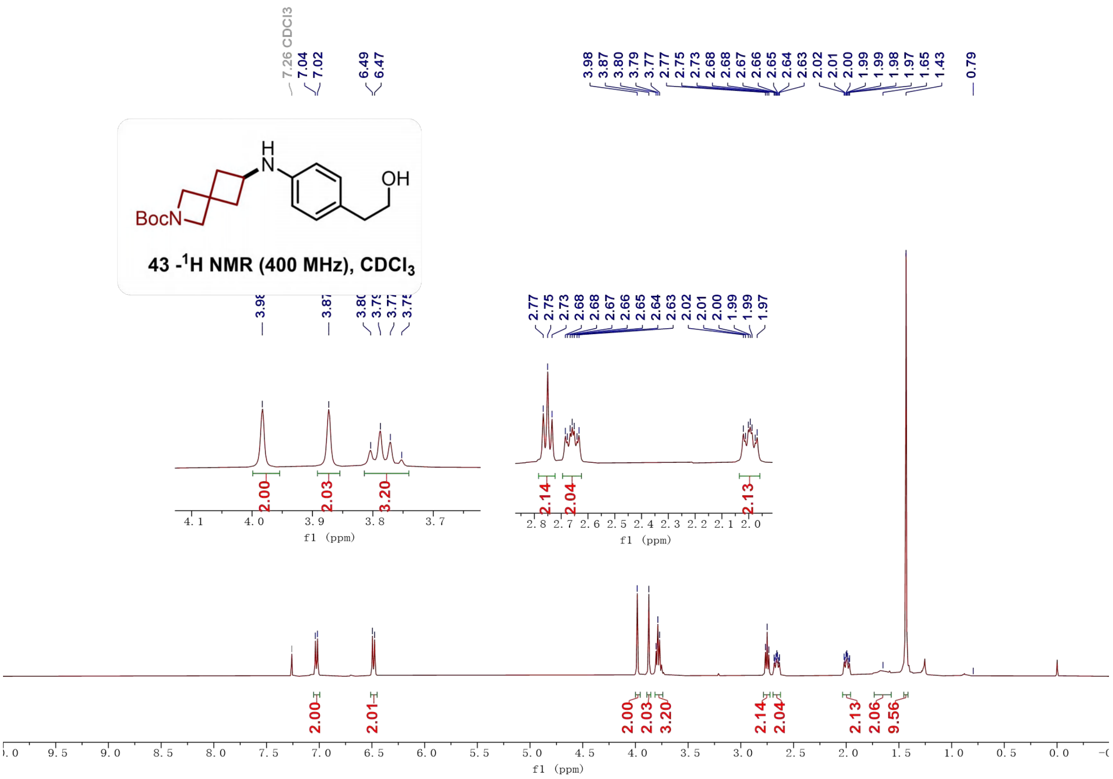

Supplementary Figure 138. ^{1}H NMR (400 MHz, $CDCl_3$) spectrum of compound **43**

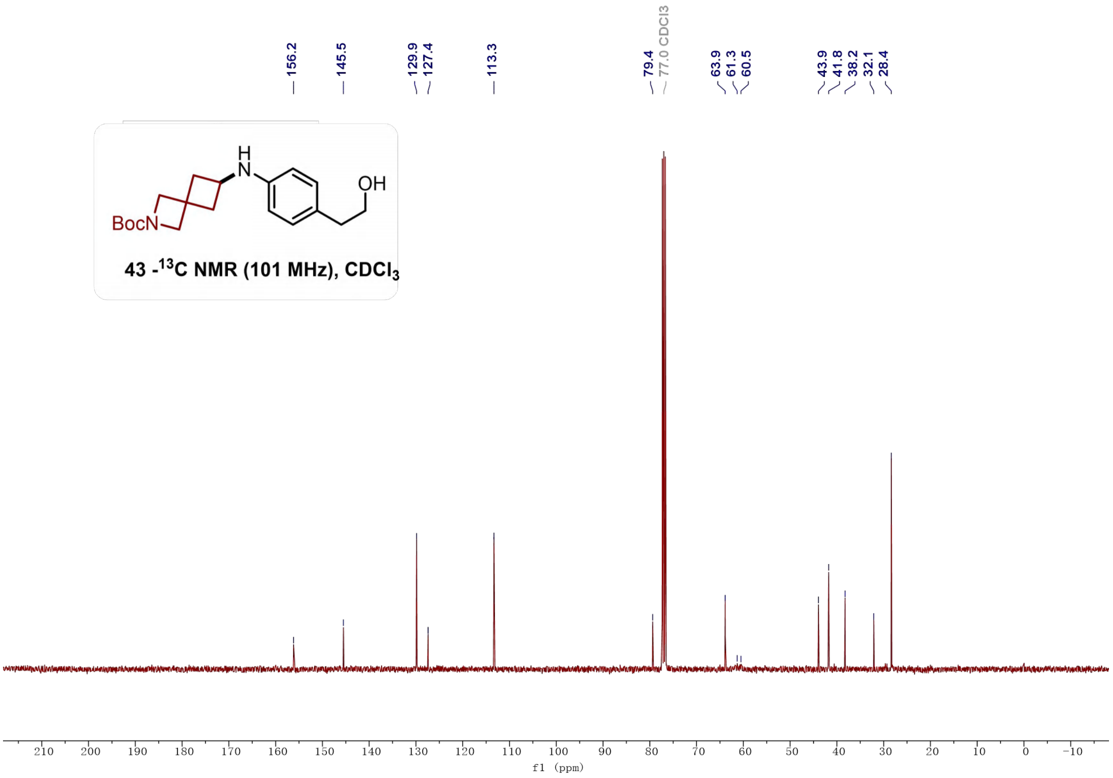

Supplementary Figure 139. ^{13}C NMR (101 MHz, $CDCl_3$) spectrum of compound **43**

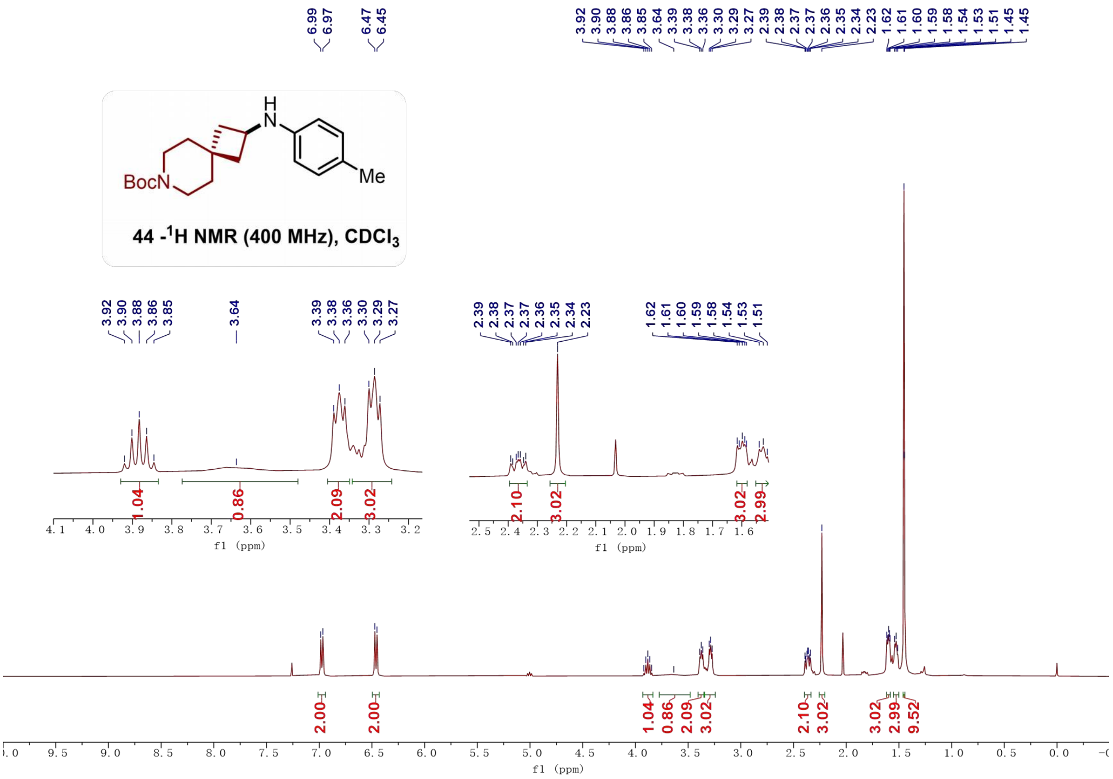

Supplementary Figure 140. ^{1}H NMR (400 MHz, $CDCl_3$) spectrum of compound **44**

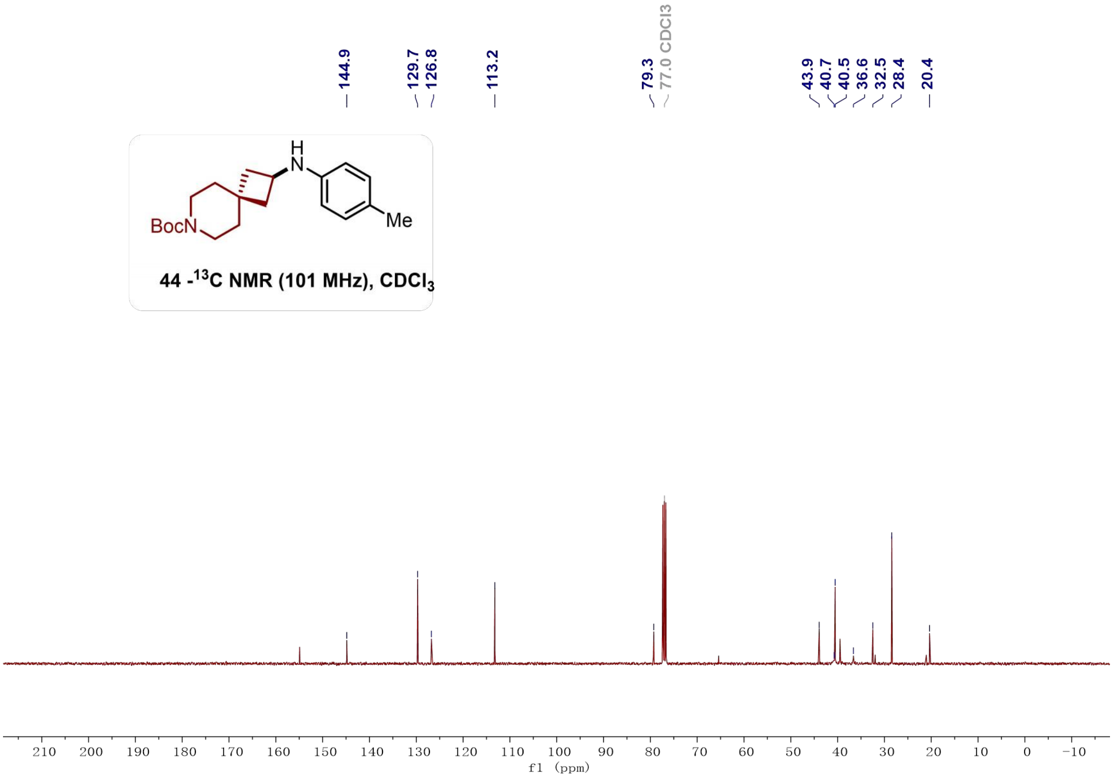

Supplementary Figure 141. ^{13}C NMR (101 MHz, $CDCl_3$) spectrum of compound **44**

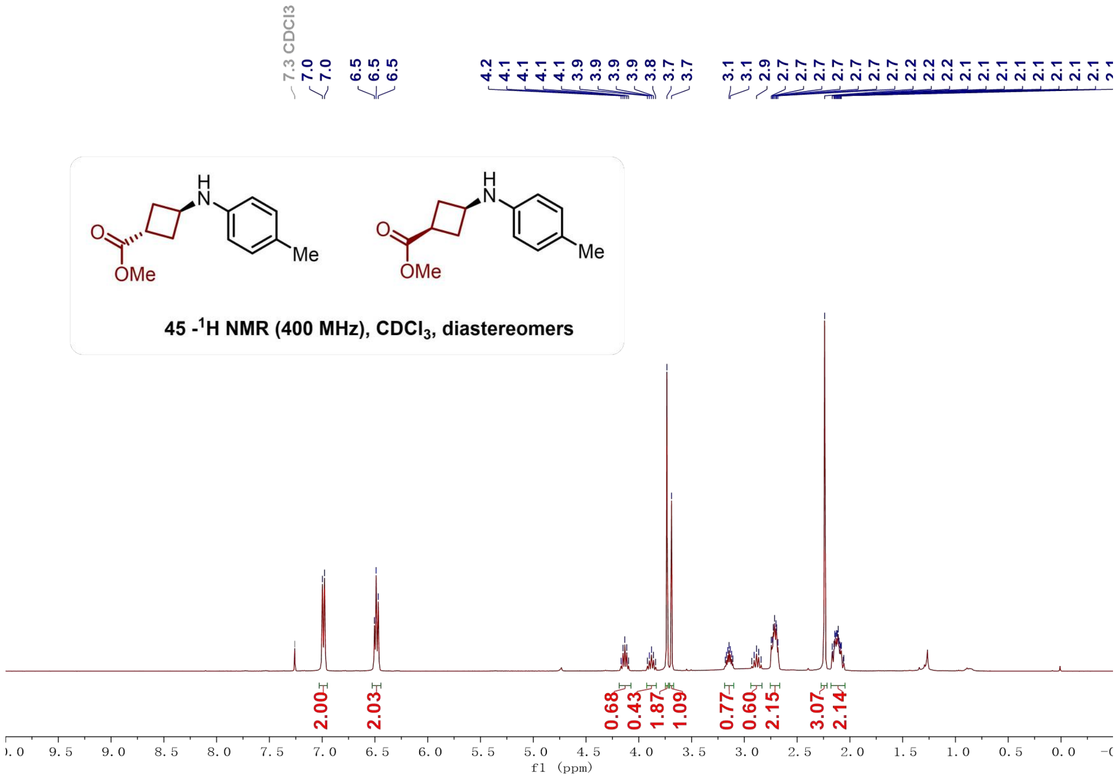

Supplementary Figure 142. ^{1}H NMR (400 MHz, $CDCl_3$) spectrum of compound **45**

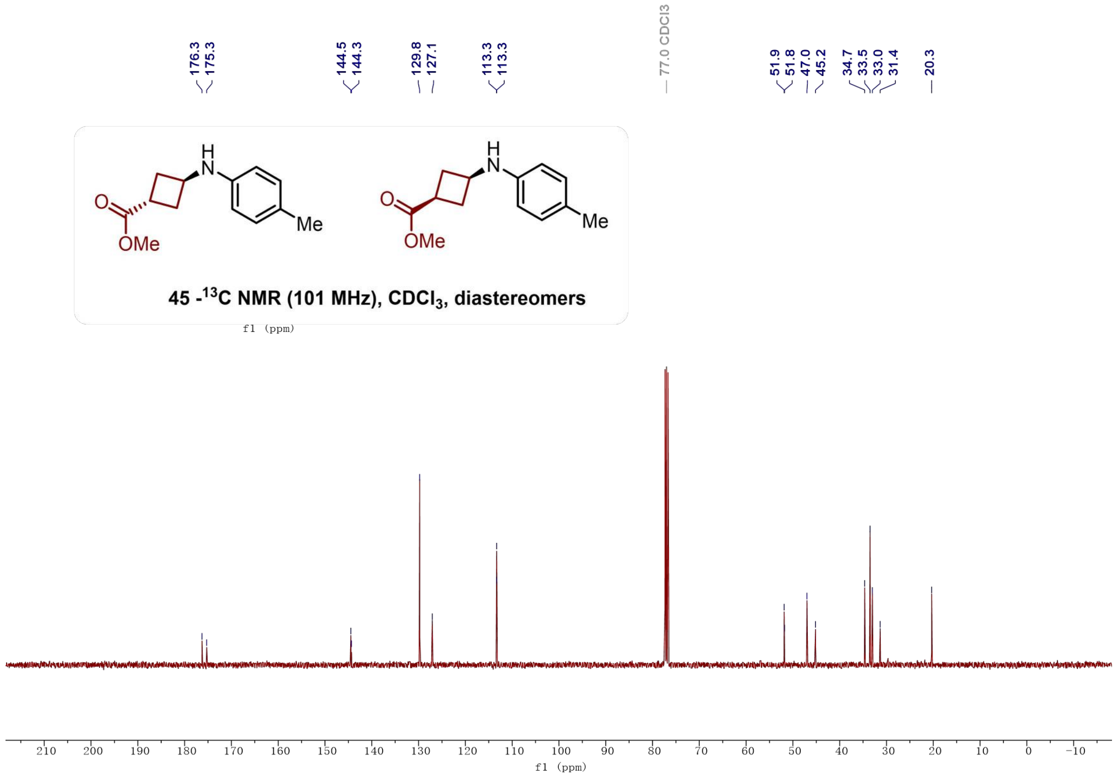

Supplementary Figure 143. ^{13}C NMR (101 MHz, $CDCl_3$) spectrum of compound **45**

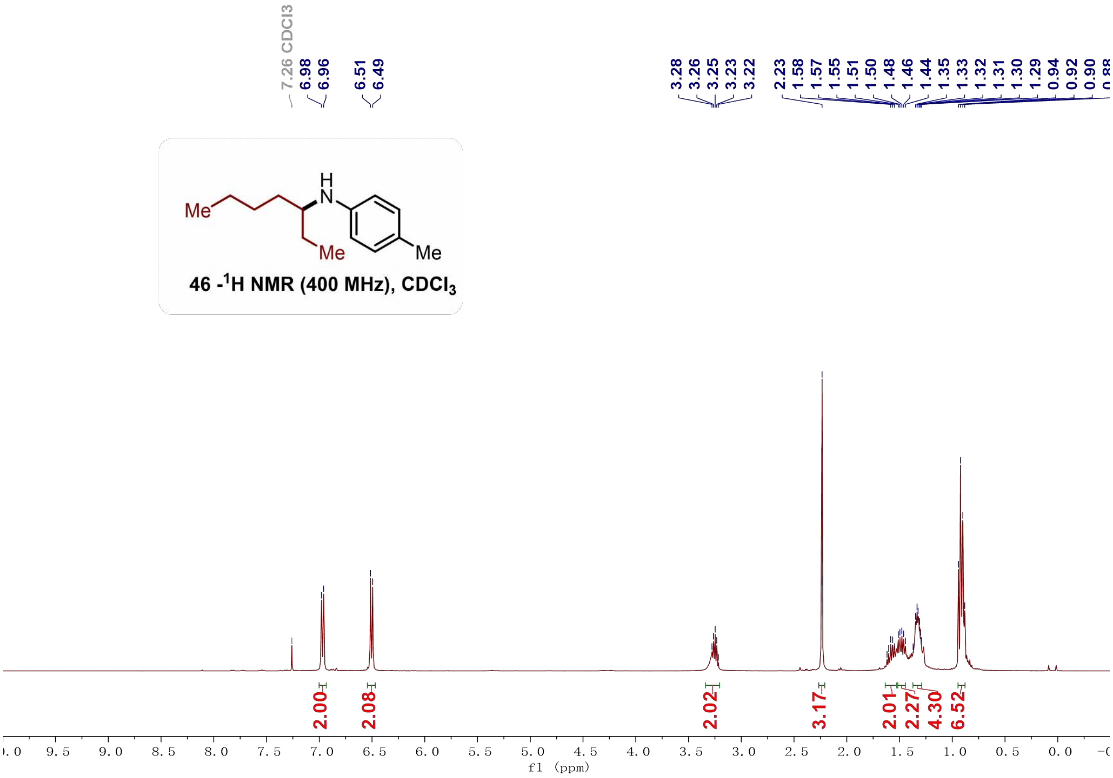

Supplementary Figure 144. ^{1}H NMR (400 MHz, $CDCl_3$) spectrum of compound **46**

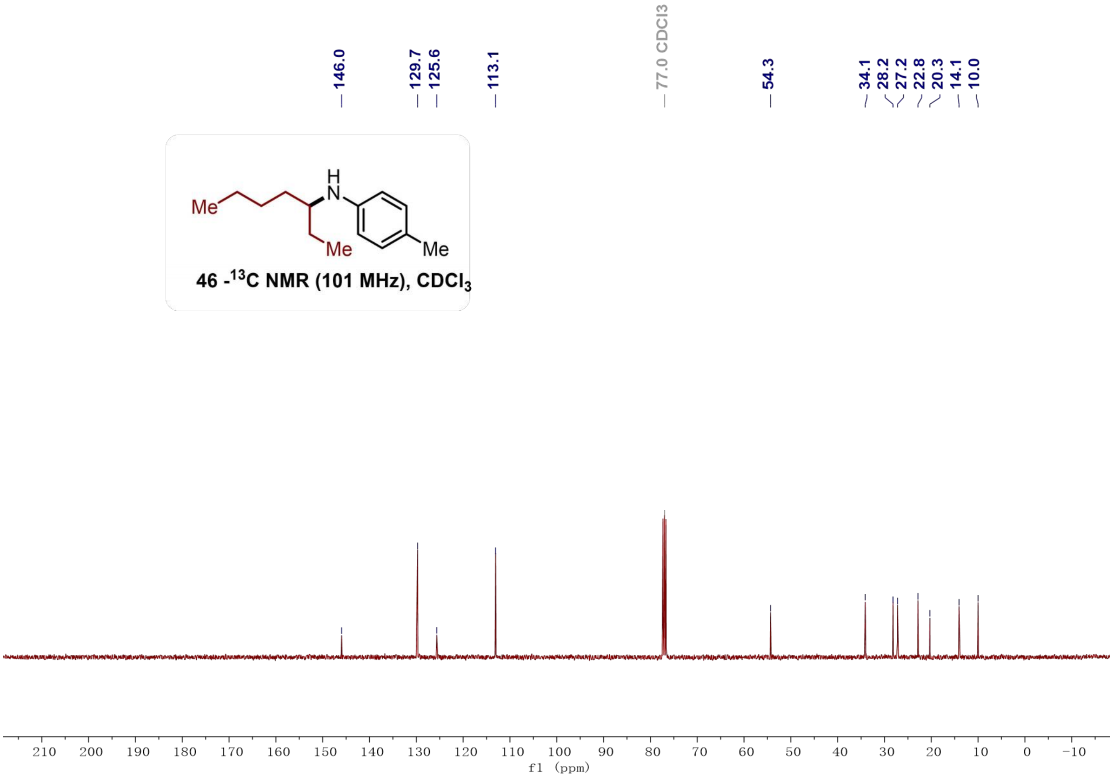

Supplementary Figure 145. ^{13}C NMR (101 MHz, $CDCl_3$) spectrum of compound **46**

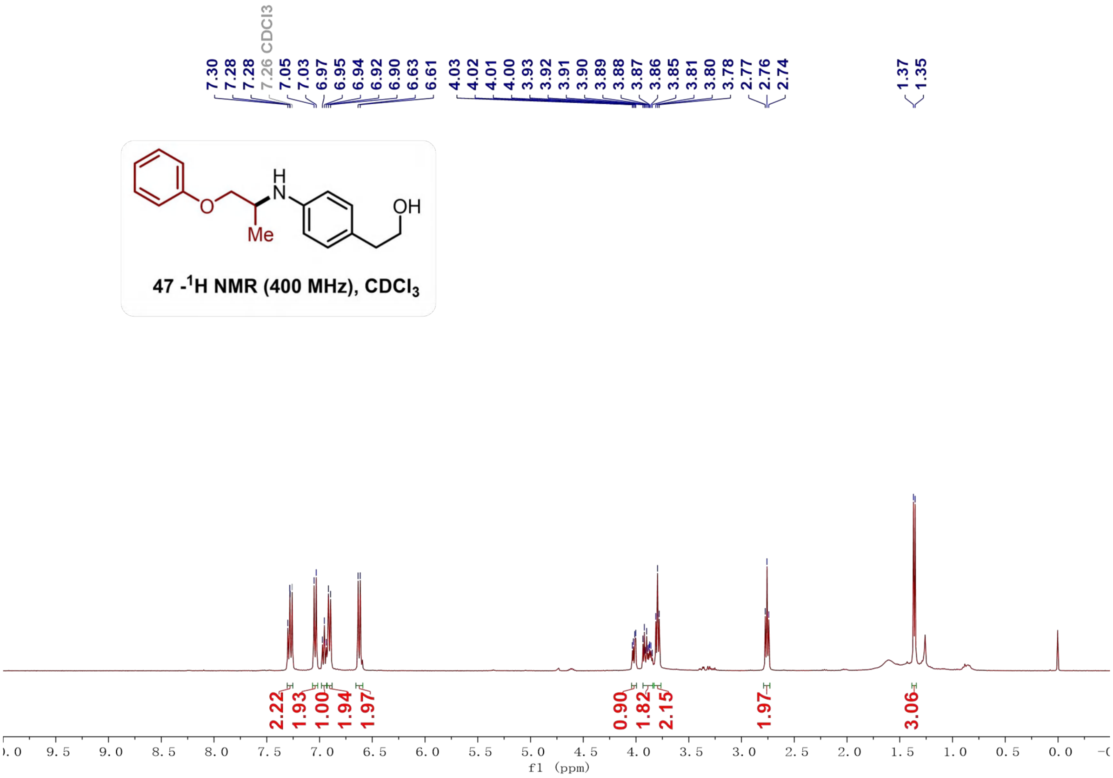

Supplementary Figure 146. ^{1}H NMR (400 MHz, $CDCl_3$) spectrum of compound **47**

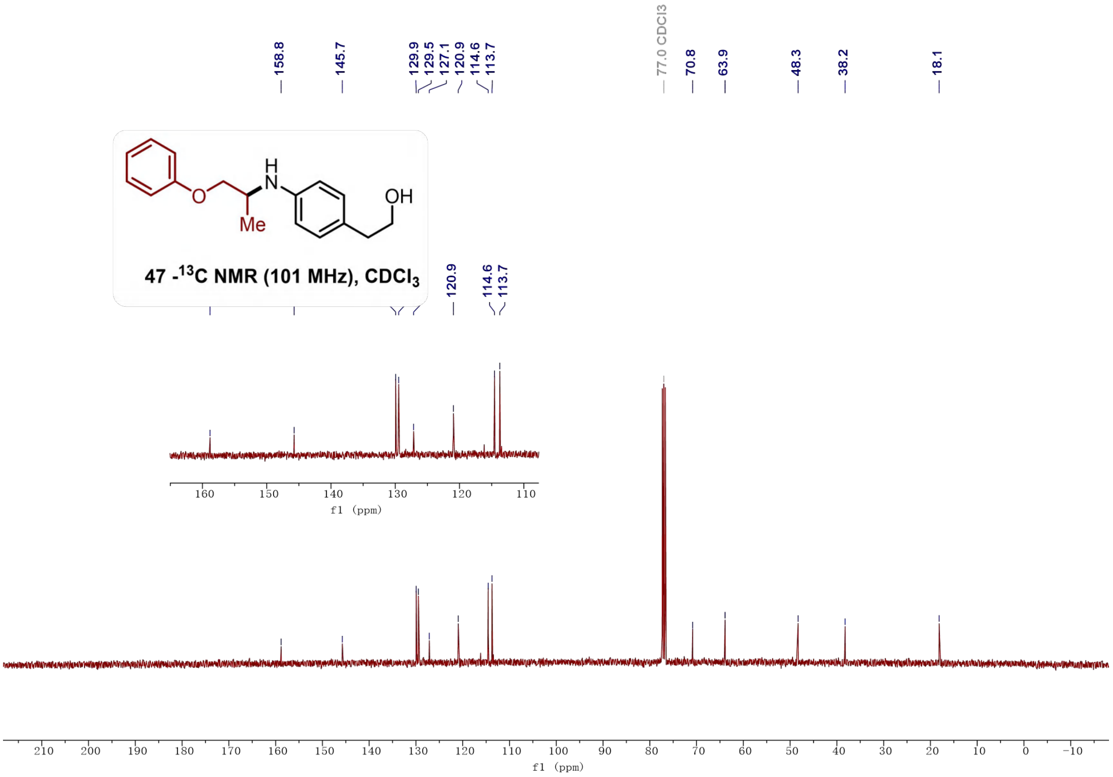

Supplementary Figure 147. ^{13}C NMR (101 MHz, $CDCl_3$) spectrum of compound **47**

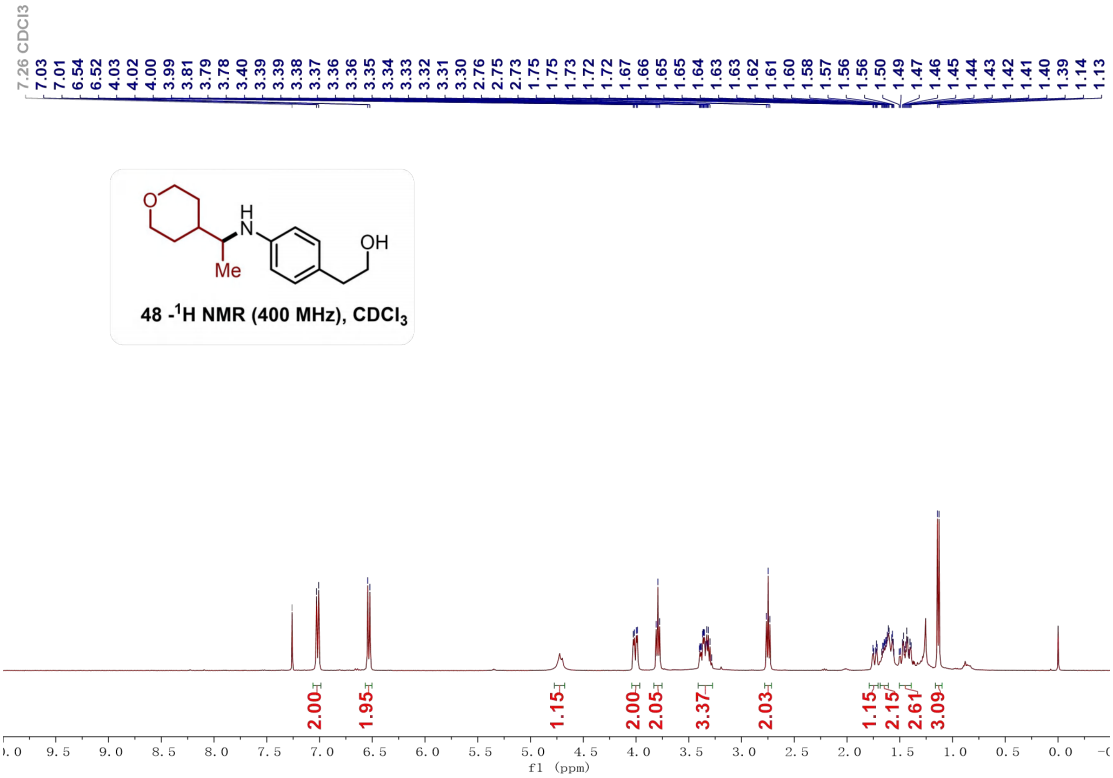

Supplementary Figure 148. ^{1}H NMR (400 MHz, $CDCl_3$) spectrum of compound **48**

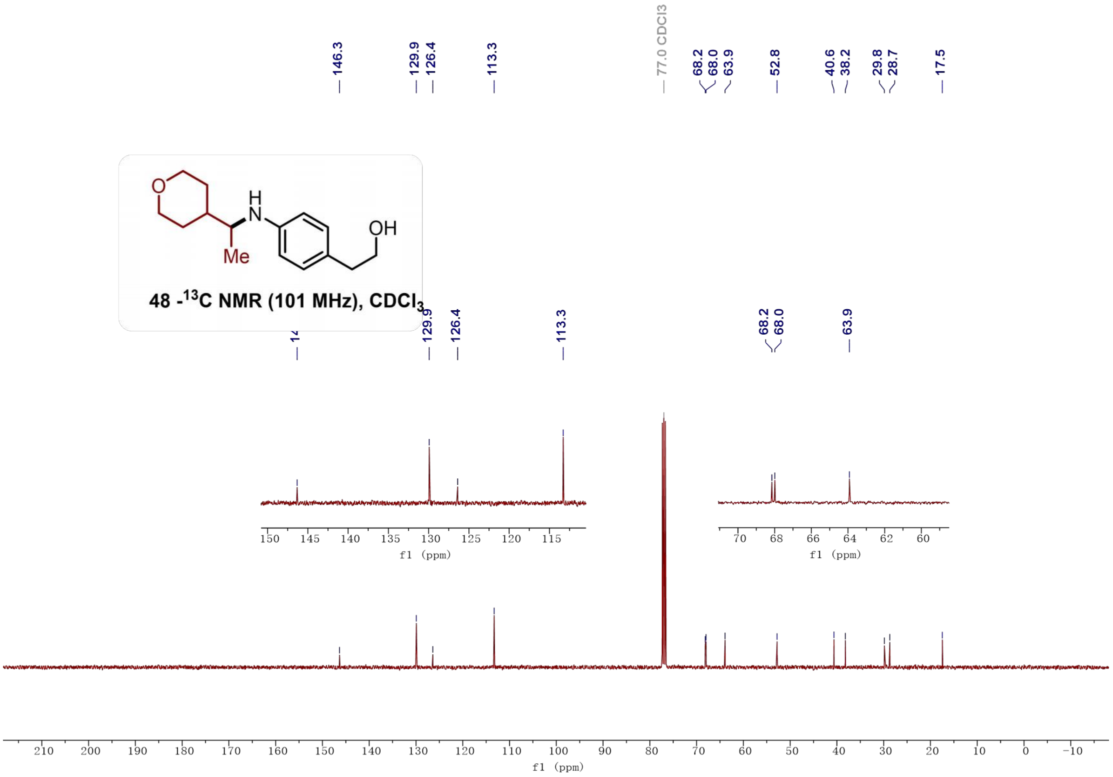

Supplementary Figure 149. ^{13}C NMR (101 MHz, $CDCl_3$) spectrum of compound **48**

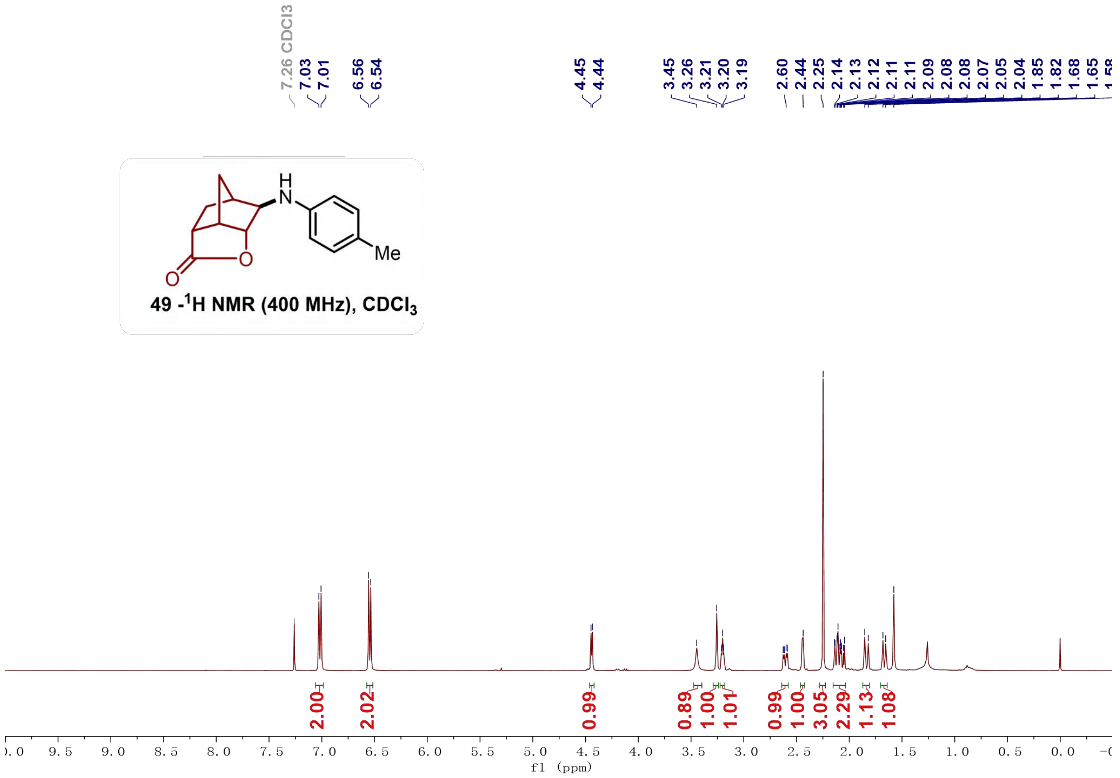

Supplementary Figure 150. ^{1}H NMR (400 MHz, $CDCl_3$) spectrum of compound **49**

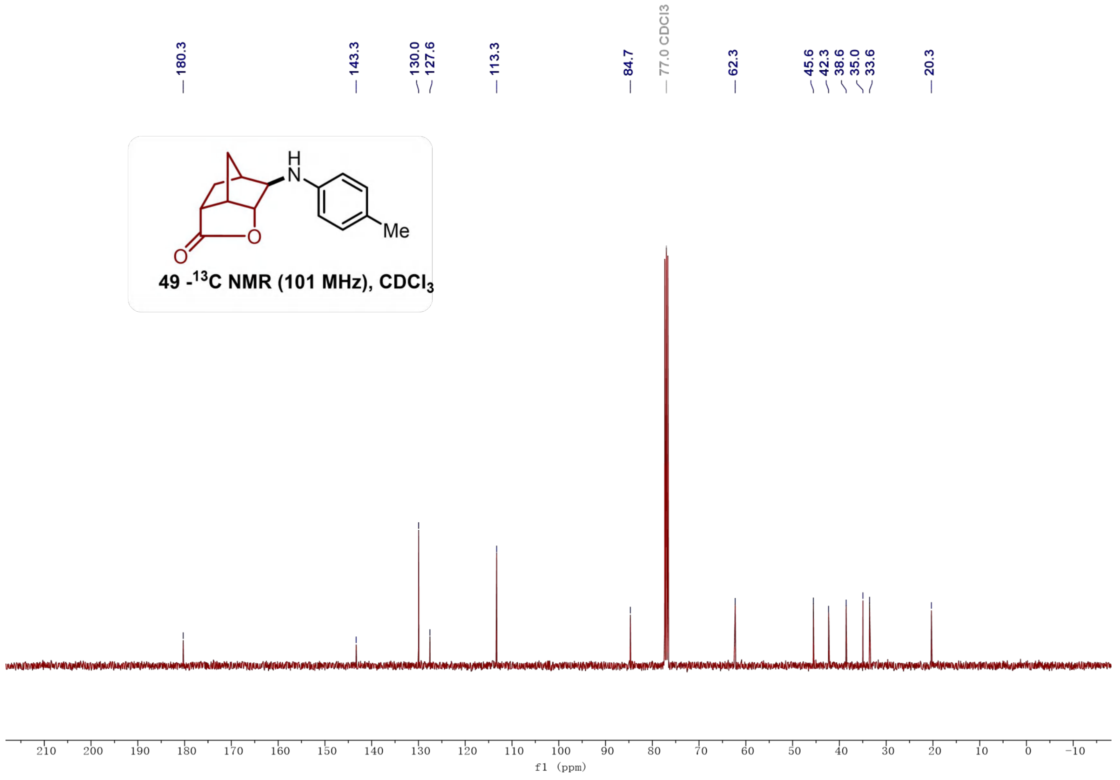

Supplementary Figure 151. ^{13}C NMR (101 MHz, $CDCl_3$) spectrum of compound **49**

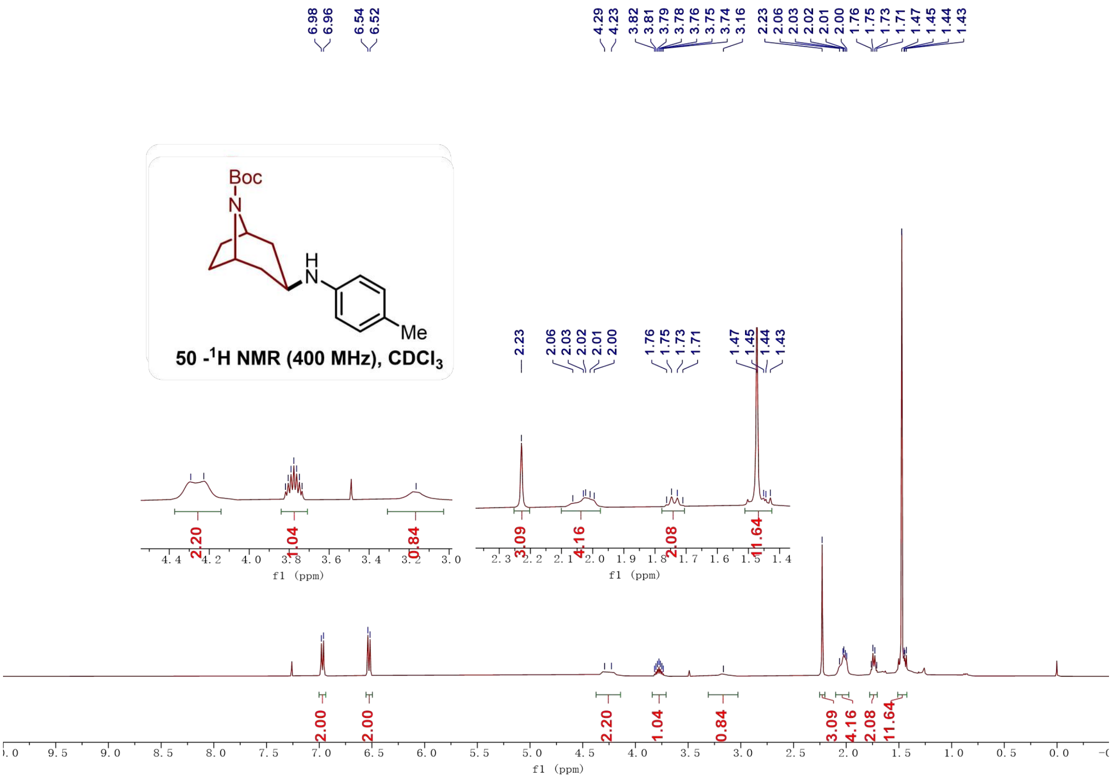

Supplementary Figure 152. ^{1}H NMR (400 MHz, $CDCl_3$) spectrum of compound **50**

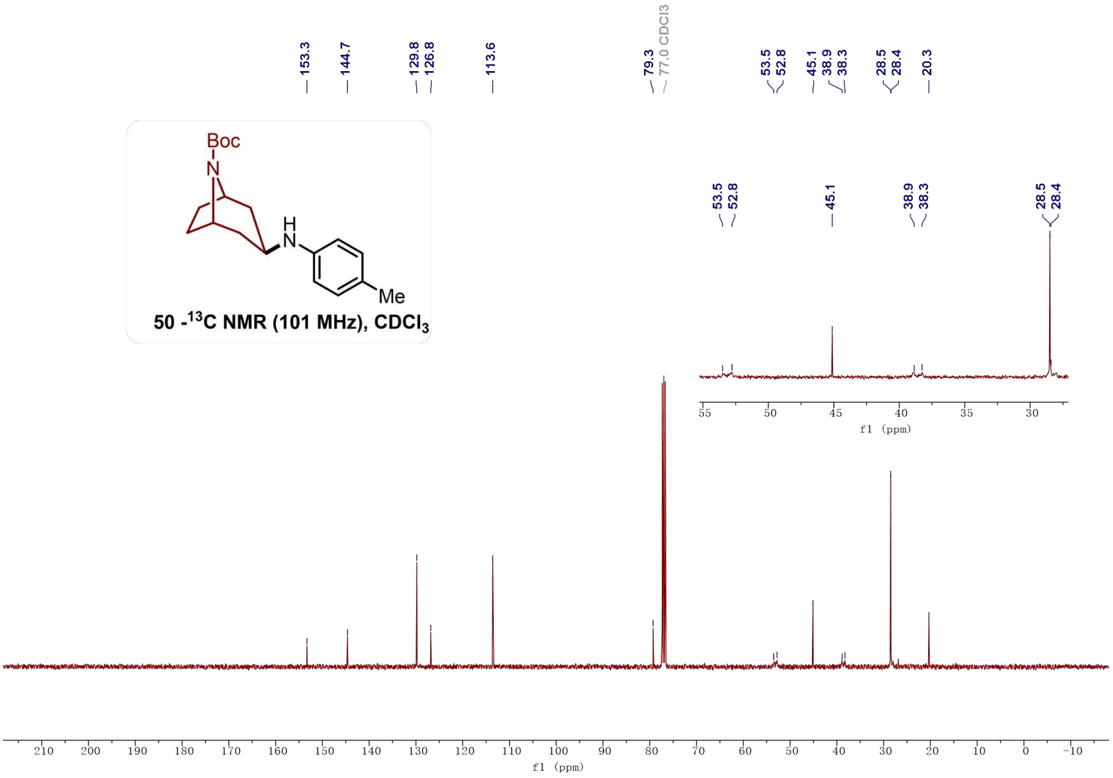

Supplementary Figure 153. ^{13}C NMR (101 MHz, $CDCl_3$) spectrum of compound **49**

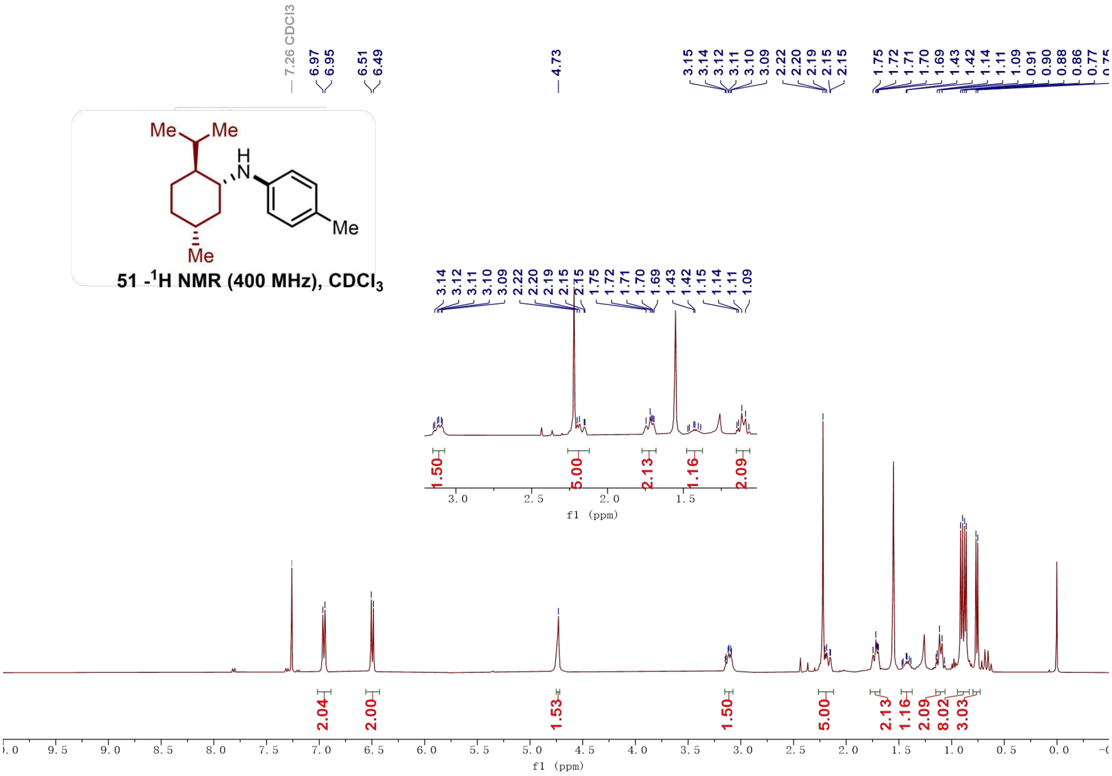

Supplementary Figure 154. ^{1}H NMR (400 MHz, $CDCl_3$) spectrum of compound **51**

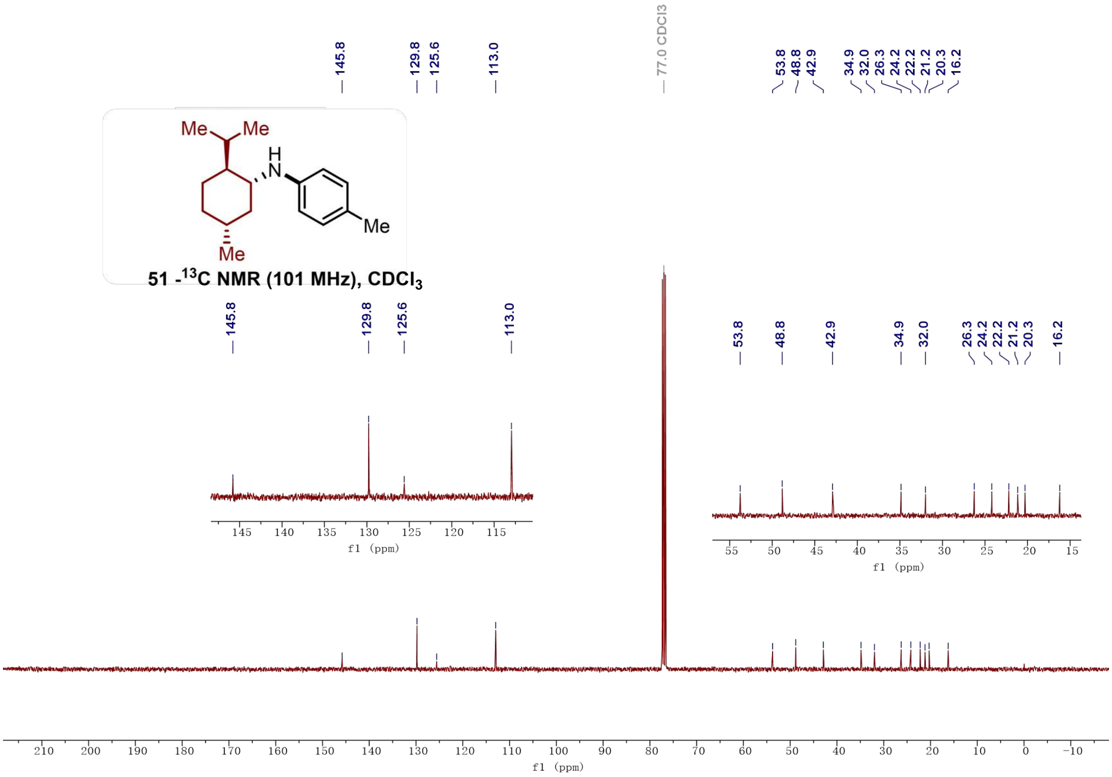

Supplementary Figure 155. ^{13}C NMR (101 MHz, $CDCl_3$) spectrum of compound **51**

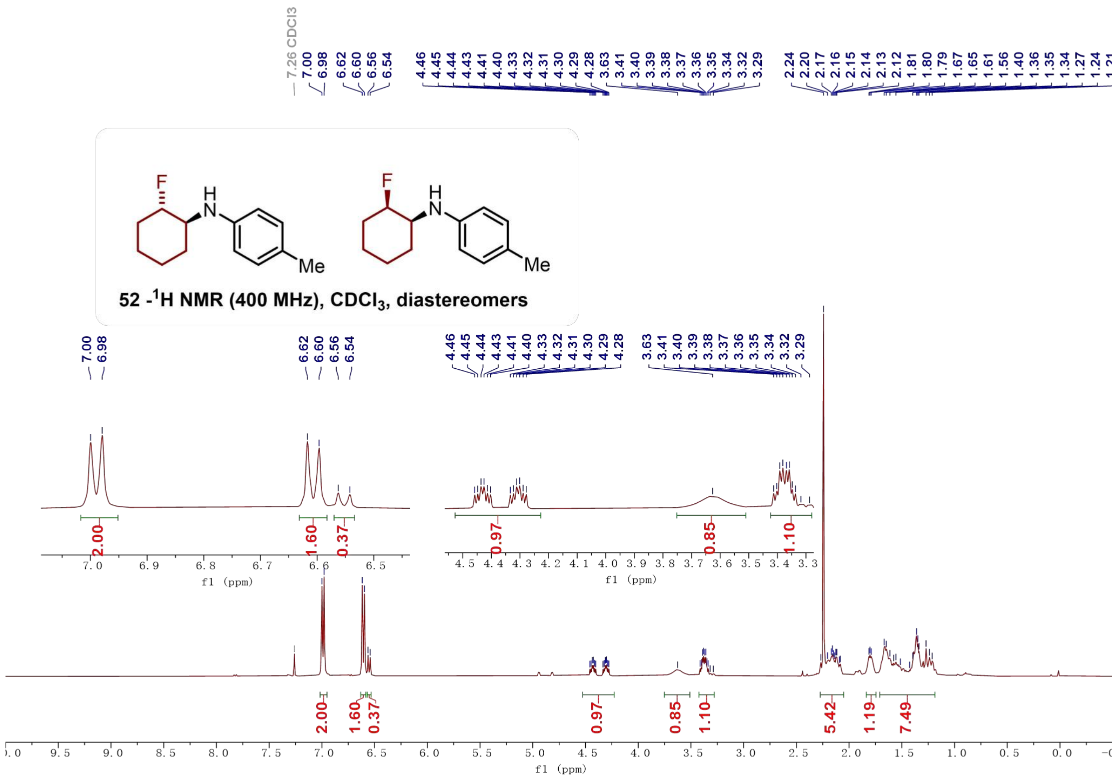

Supplementary Figure 156. ^{1}H NMR (400 MHz, $CDCl_3$) spectrum of compound **52**

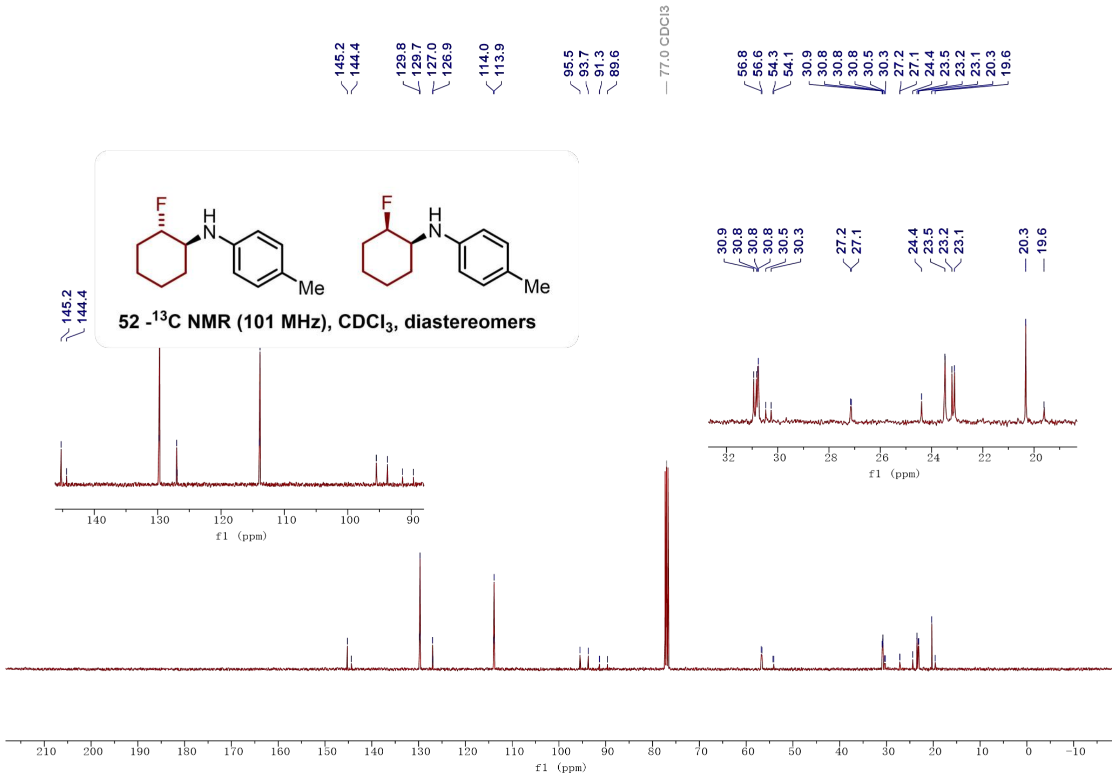

Supplementary Figure 157. ^{13}C NMR (101 MHz, $CDCl_3$) spectrum of compound **52**

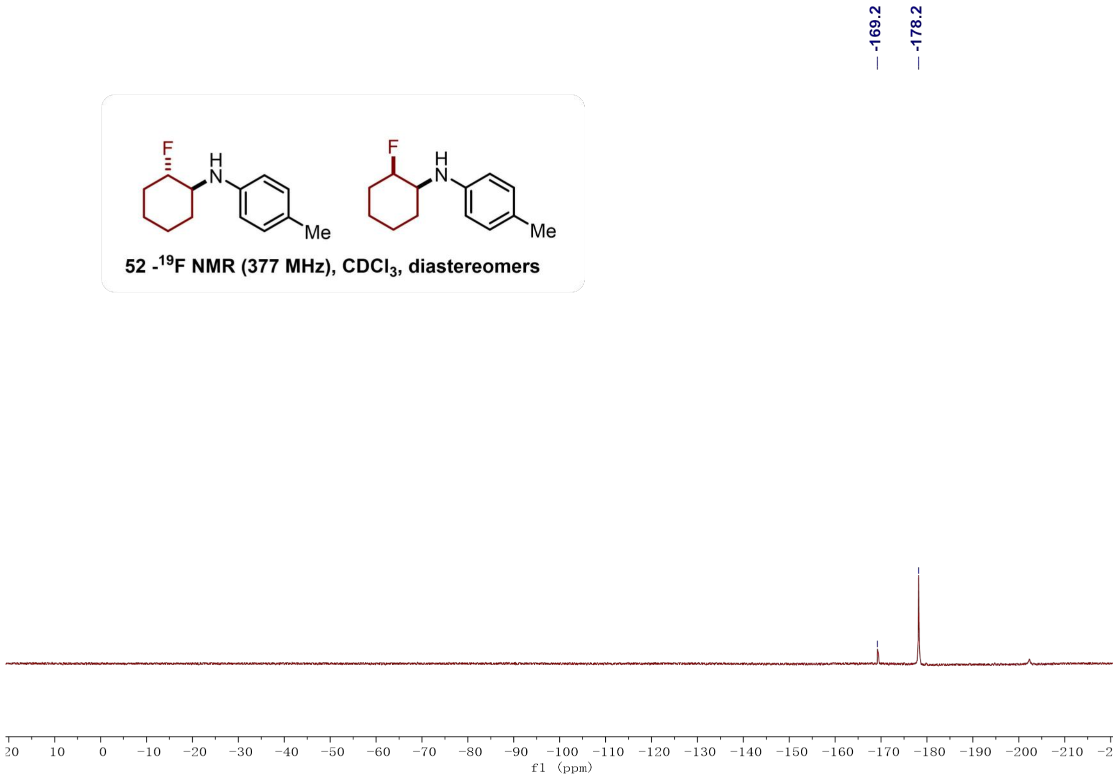

Supplementary Figure 158. ^{19}F NMR (377 MHz, $CDCl_3$) spectrum of compound **52**

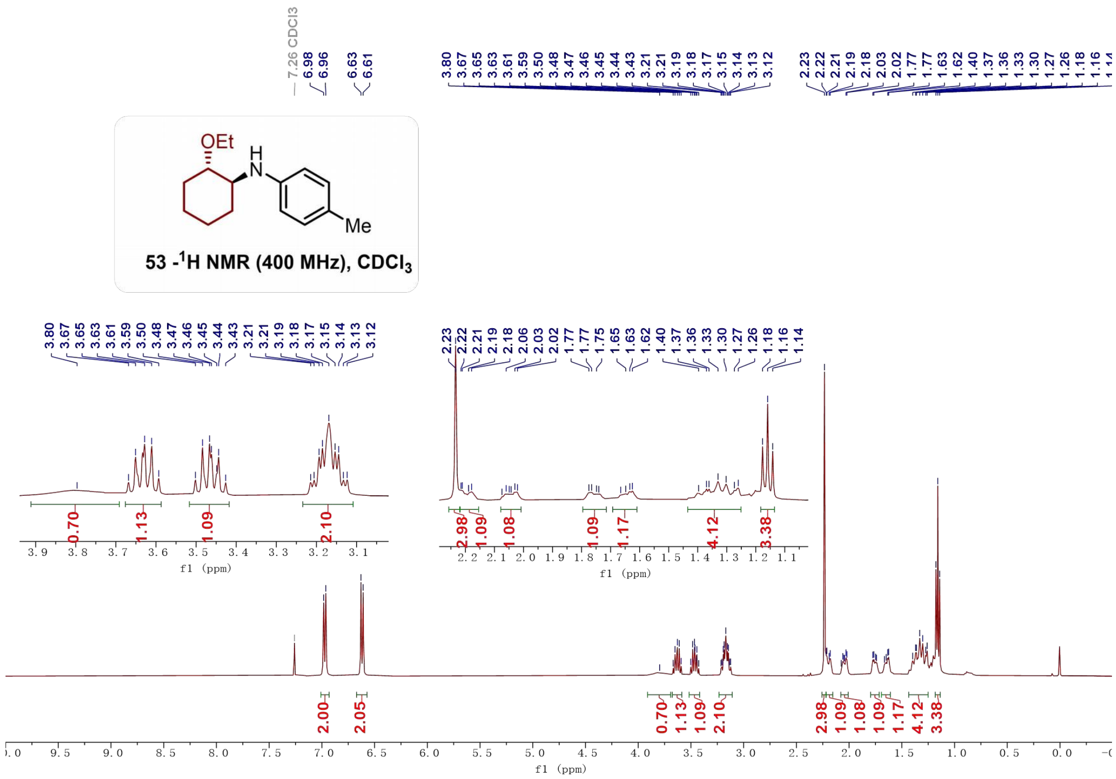

Supplementary Figure 159. ^{1}H NMR (400 MHz, $CDCl_3$) spectrum of compound **53**

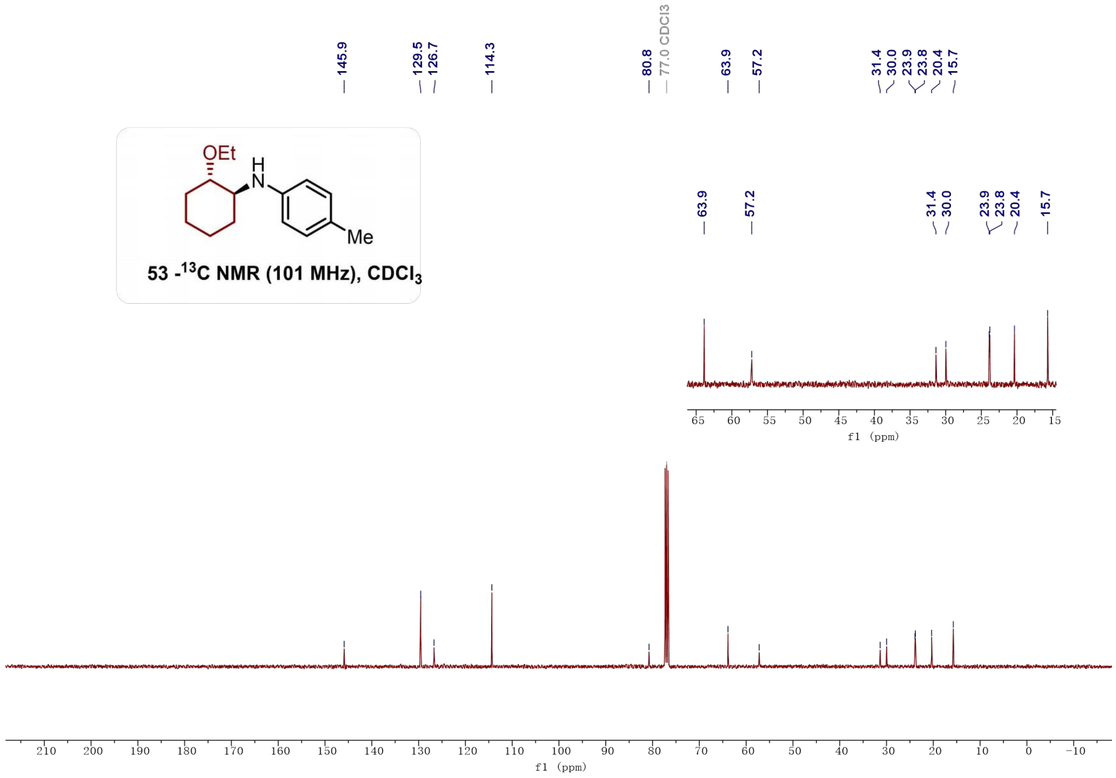

Supplementary Figure 160. ^{13}C NMR (101 MHz, $CDCl_3$) spectrum of compound **53**

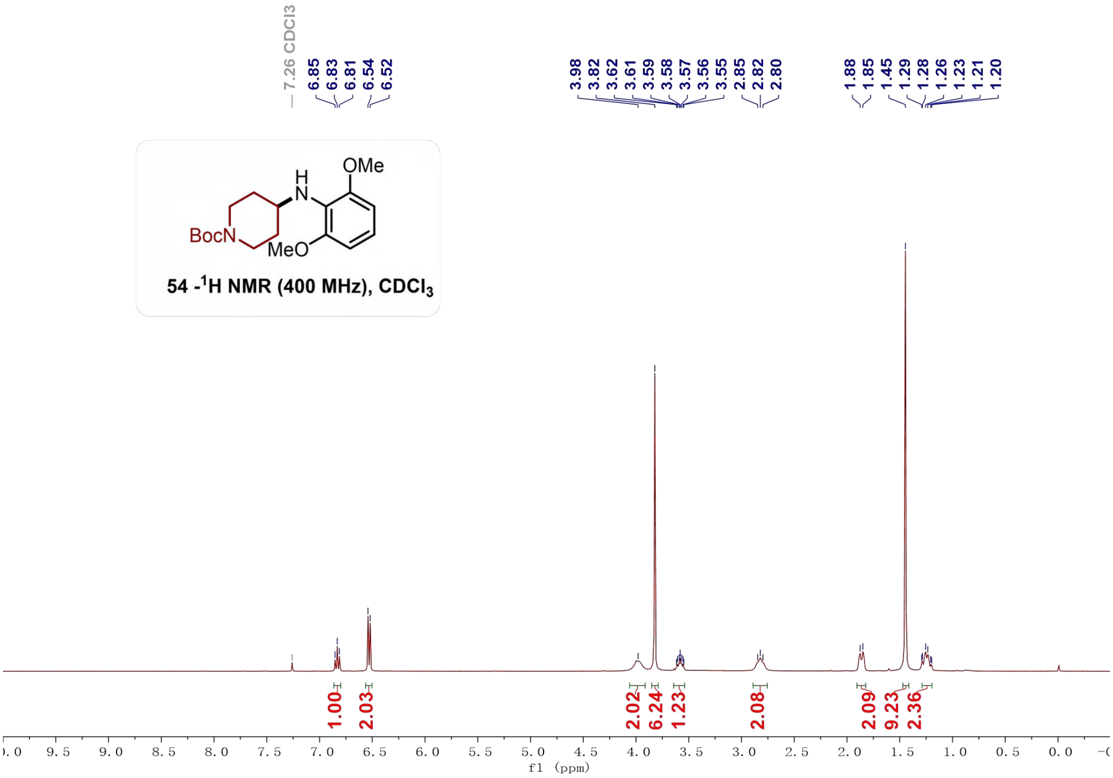

Supplementary Figure 161. ^{1}H NMR (400 MHz, $CDCl_3$) spectrum of compound **54**

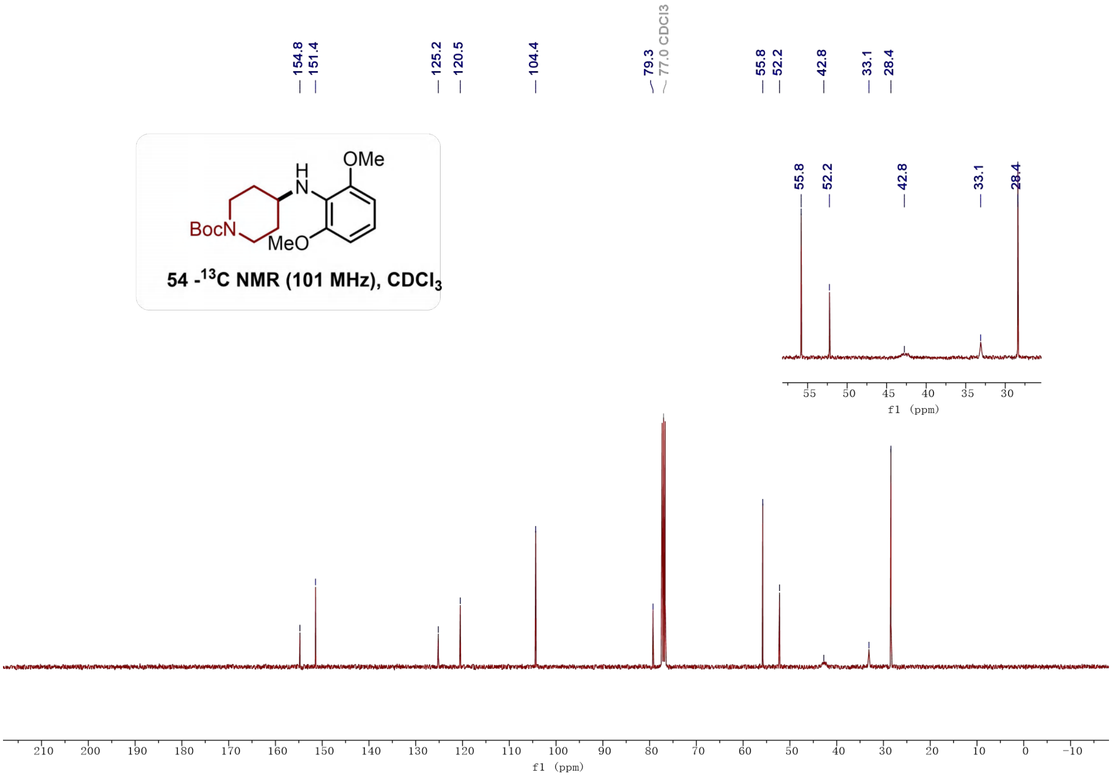

Supplementary Figure 162. ^{13}C NMR (101 MHz, $CDCl_3$) spectrum of compound **54**

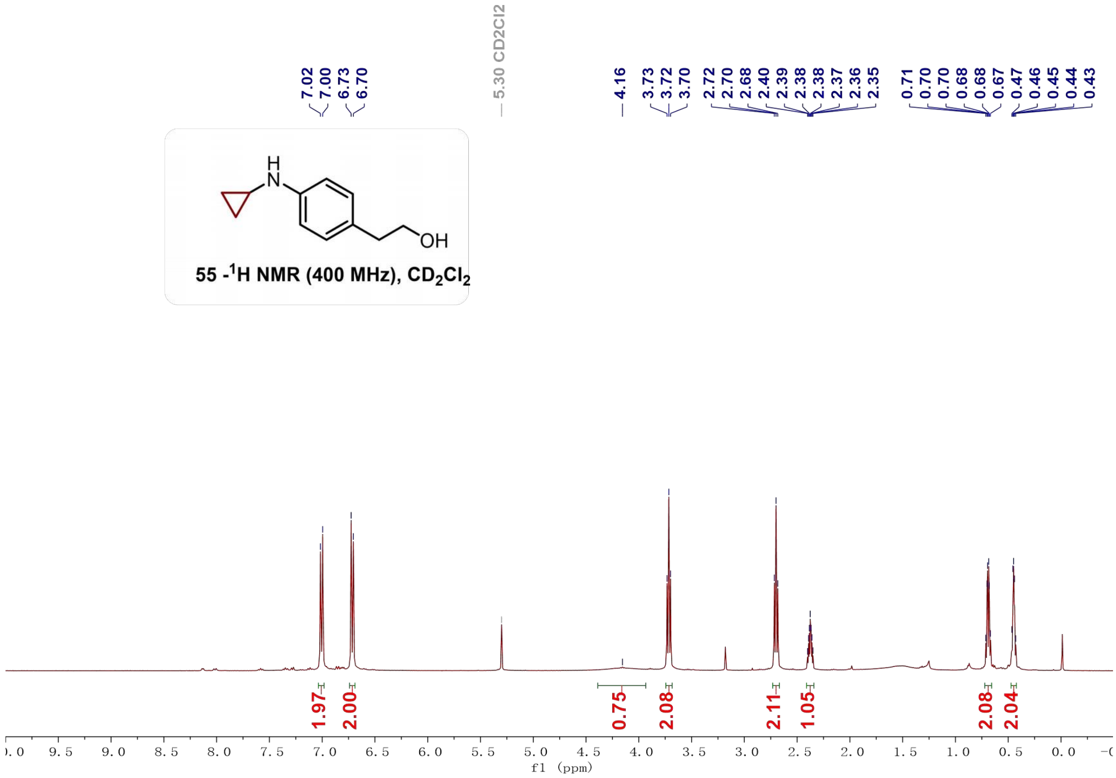

Supplementary Figure 163. ^{1}H NMR (400 MHz, CD_2Cl_2) spectrum of compound **55**

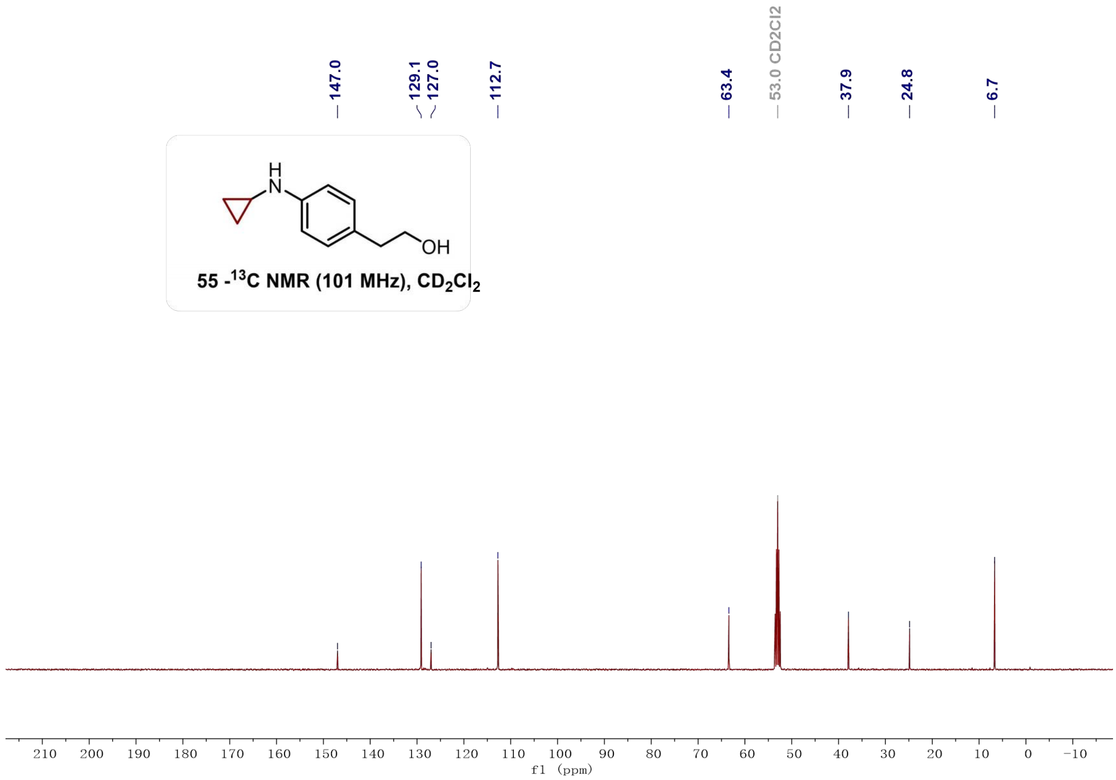

Supplementary Figure 164. ^{13}C NMR (101 MHz, CD_2Cl_2) spectrum of compound **55**

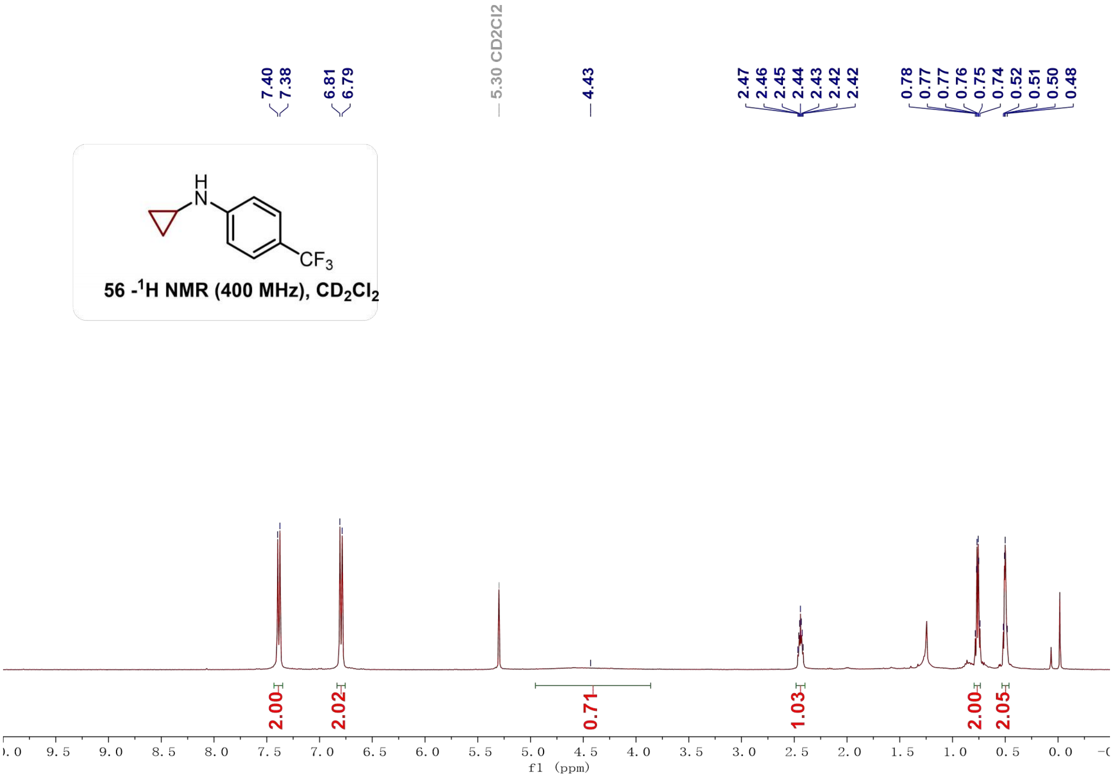

Supplementary Figure 165. ^{1}H NMR (400 MHz, CD_2Cl_2) spectrum of compound **56**

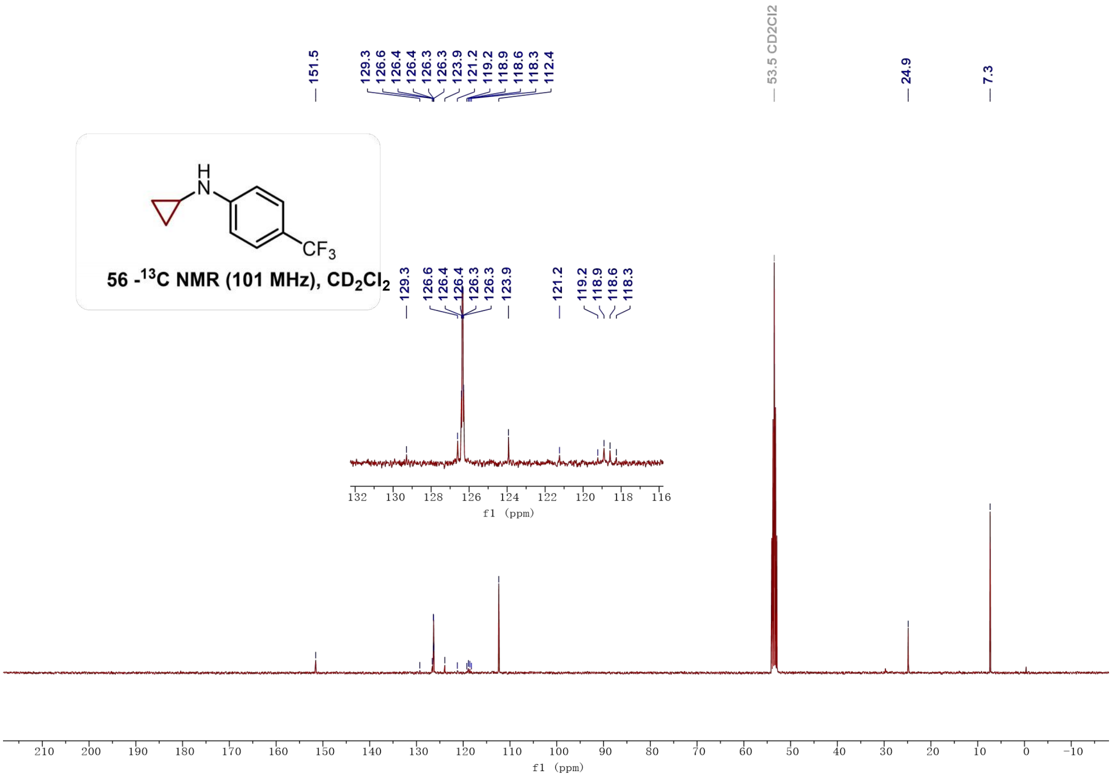

Supplementary Figure 166. ^{13}C NMR (101 MHz, CD_2Cl_2) spectrum of compound **56**

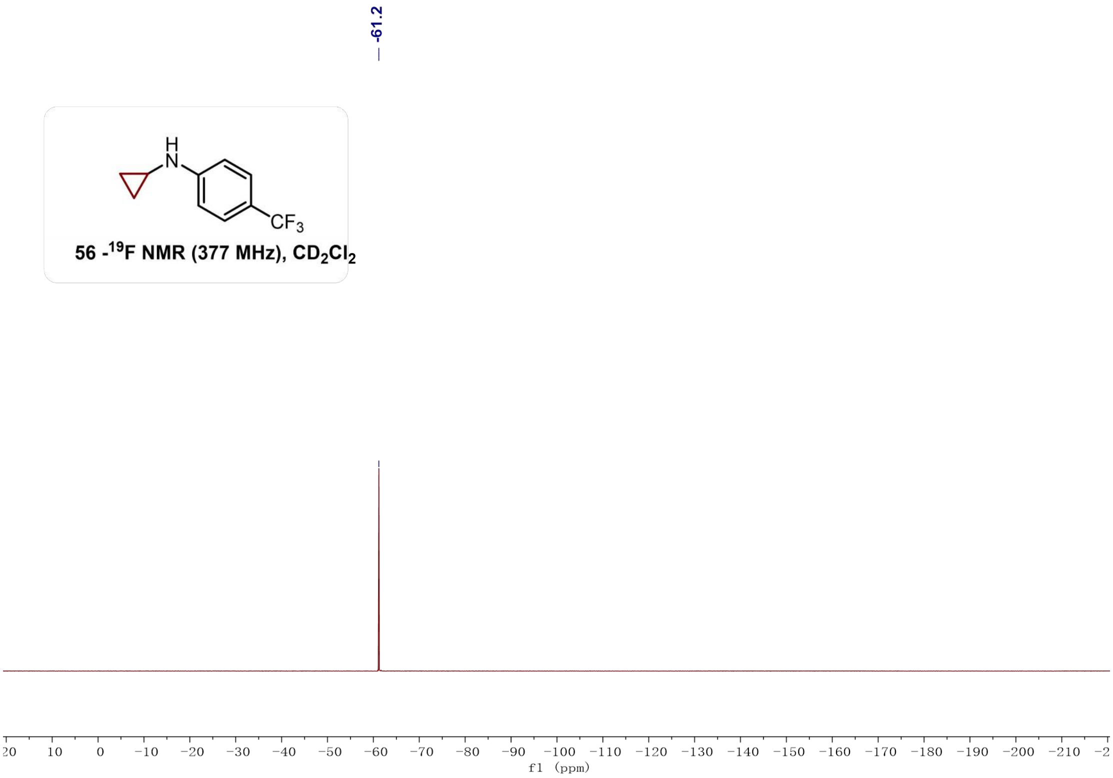

Supplementary Figure 167. ^{19}F NMR (377 MHz, CD_2Cl_2) spectrum of compound **56**

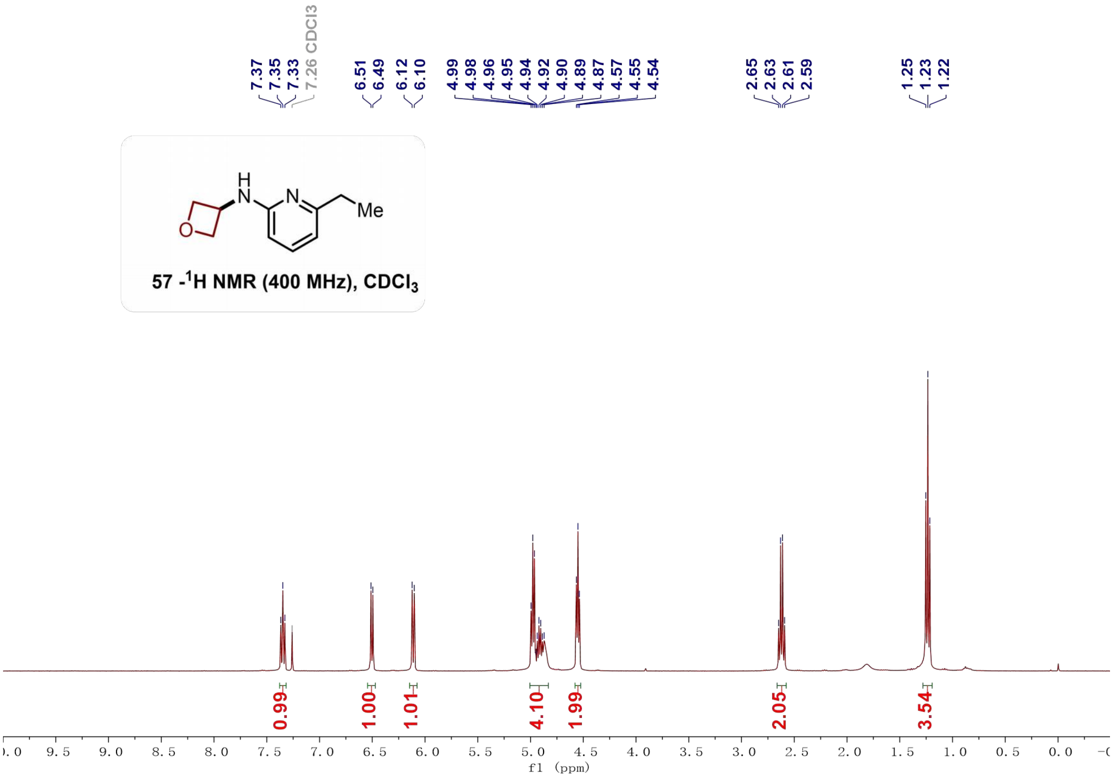

Supplementary Figure 168. ^{1}H NMR (400 MHz, $CDCl_3$) spectrum of compound **57**

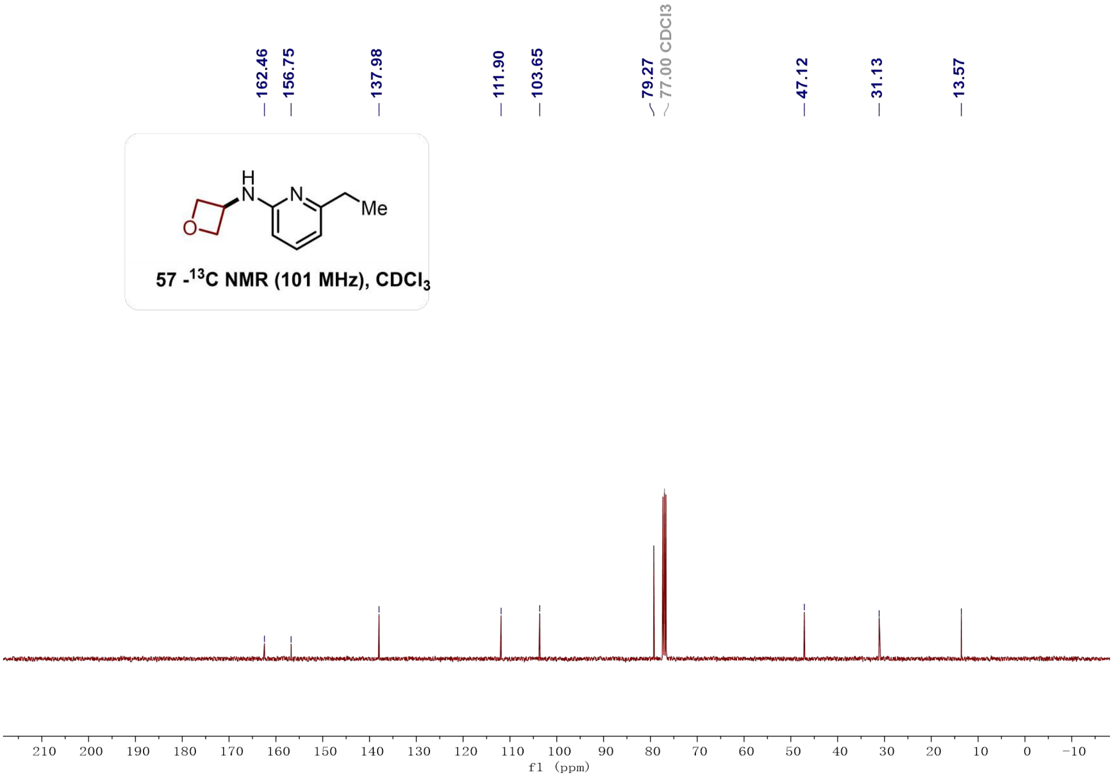

Supplementary Figure 169. ^{13}C NMR (101 MHz, $CDCl_3$) spectrum of compound **57**

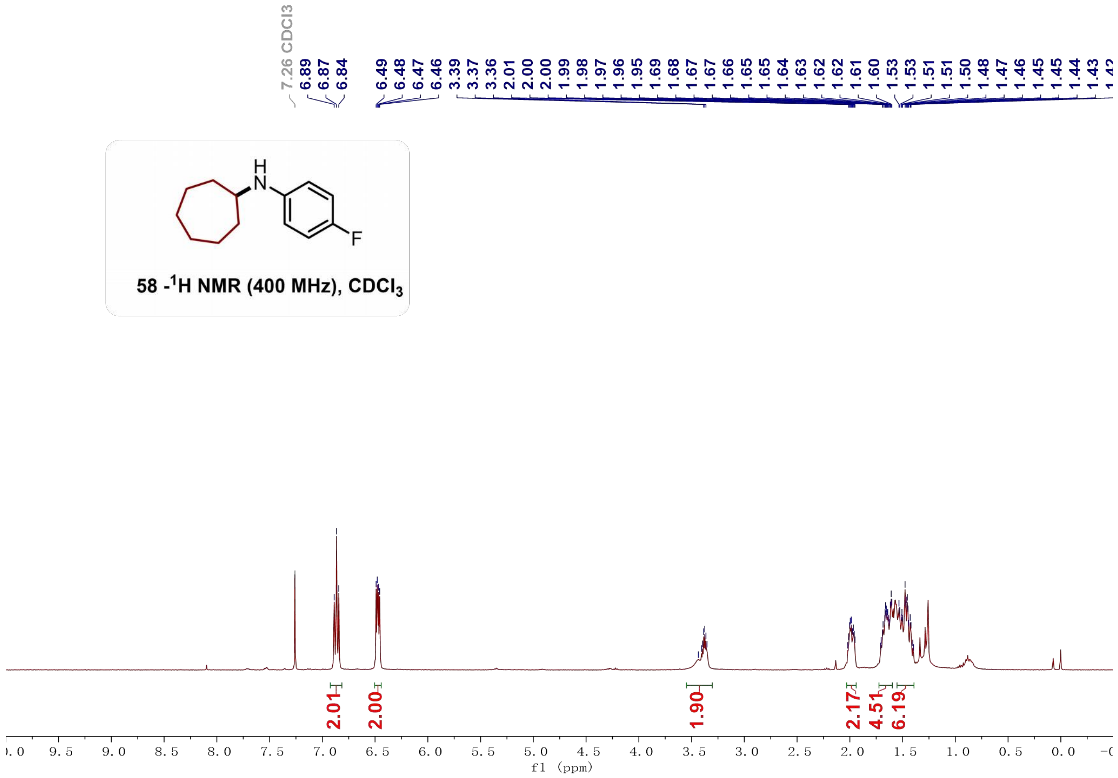

Supplementary Figure 170. ^{1}H NMR (400 MHz, $CDCl_3$) spectrum of compound **58**

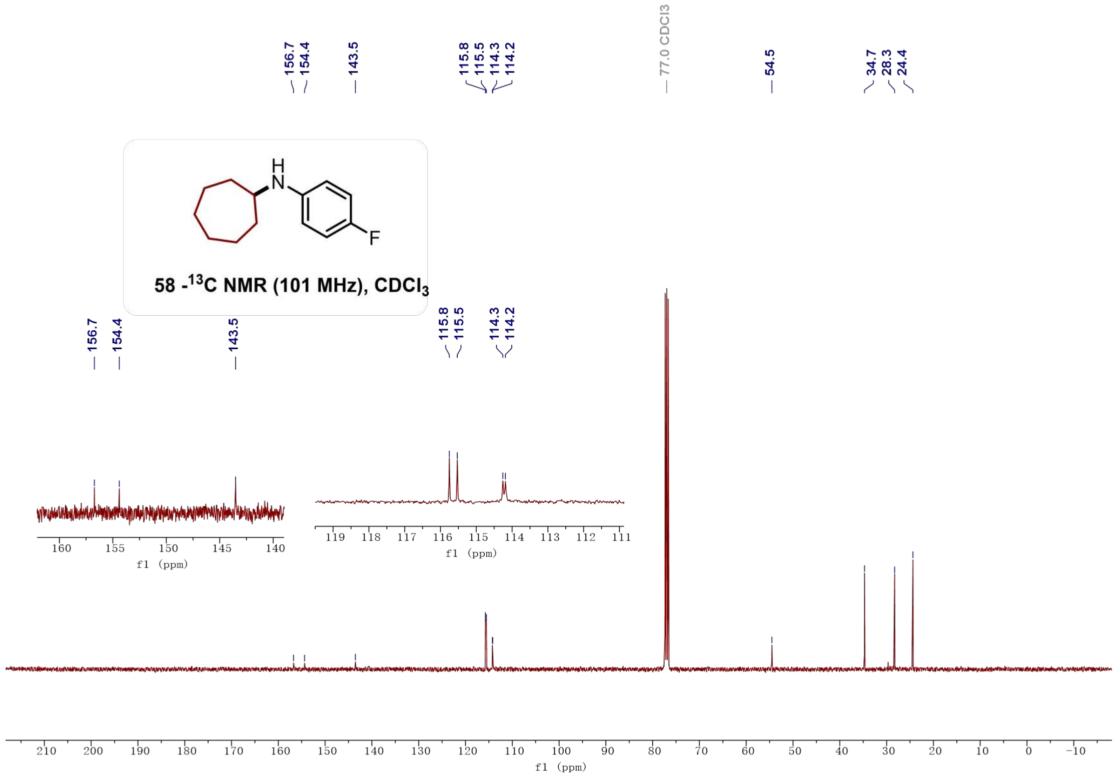

Supplementary Figure 171. ^{13}C NMR (101 MHz, $CDCl_3$) spectrum of compound **58**

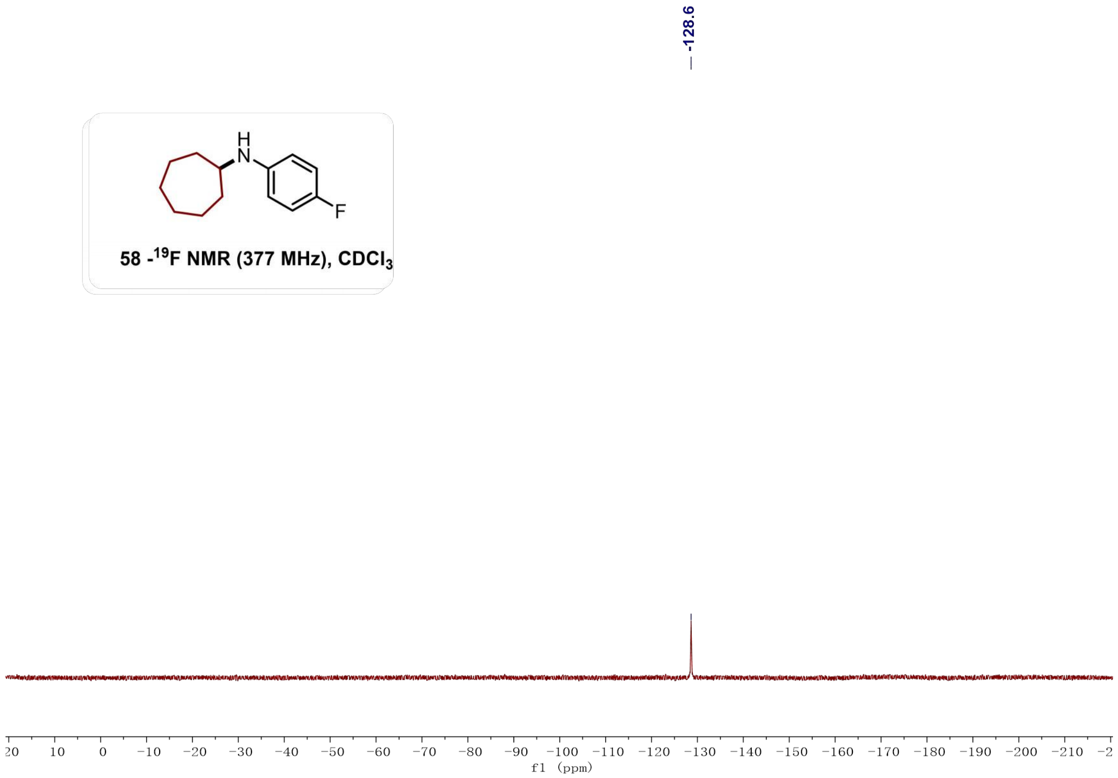

Supplementary Figure 172. ^{19}F NMR (377 MHz, $CDCl_3$) spectrum of compound **58**

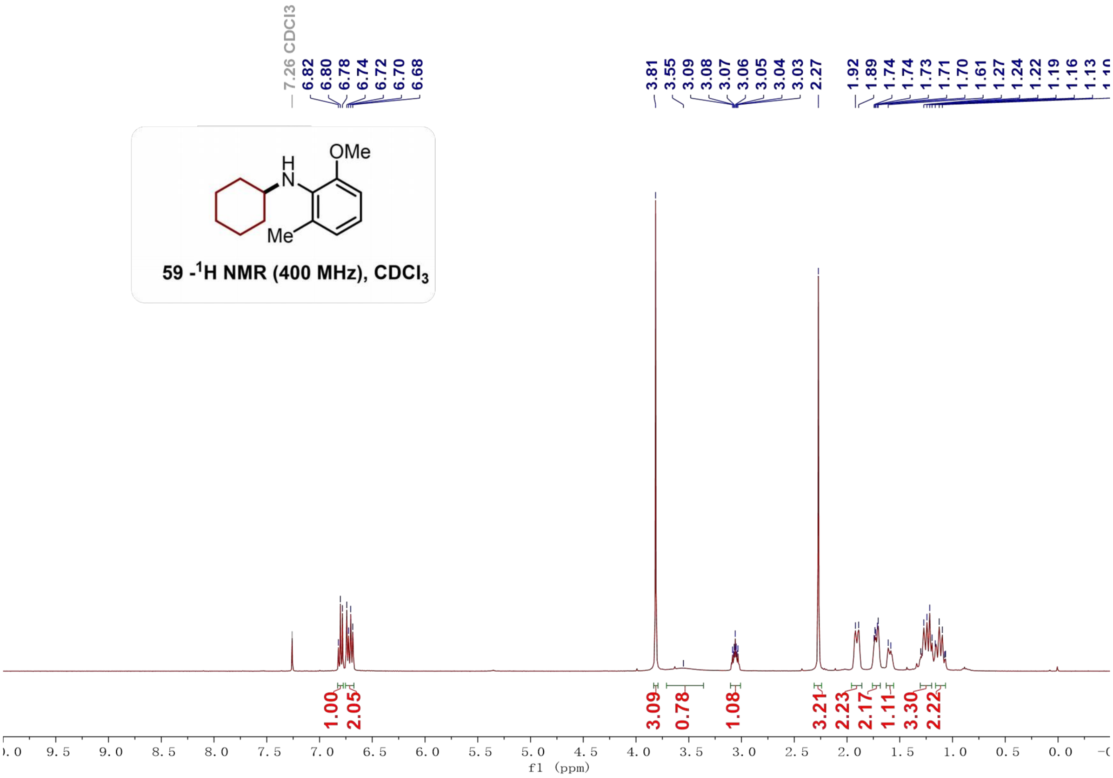

Supplementary Figure 173. ^{1}H NMR (400 MHz, $CDCl_3$) spectrum of compound **59**

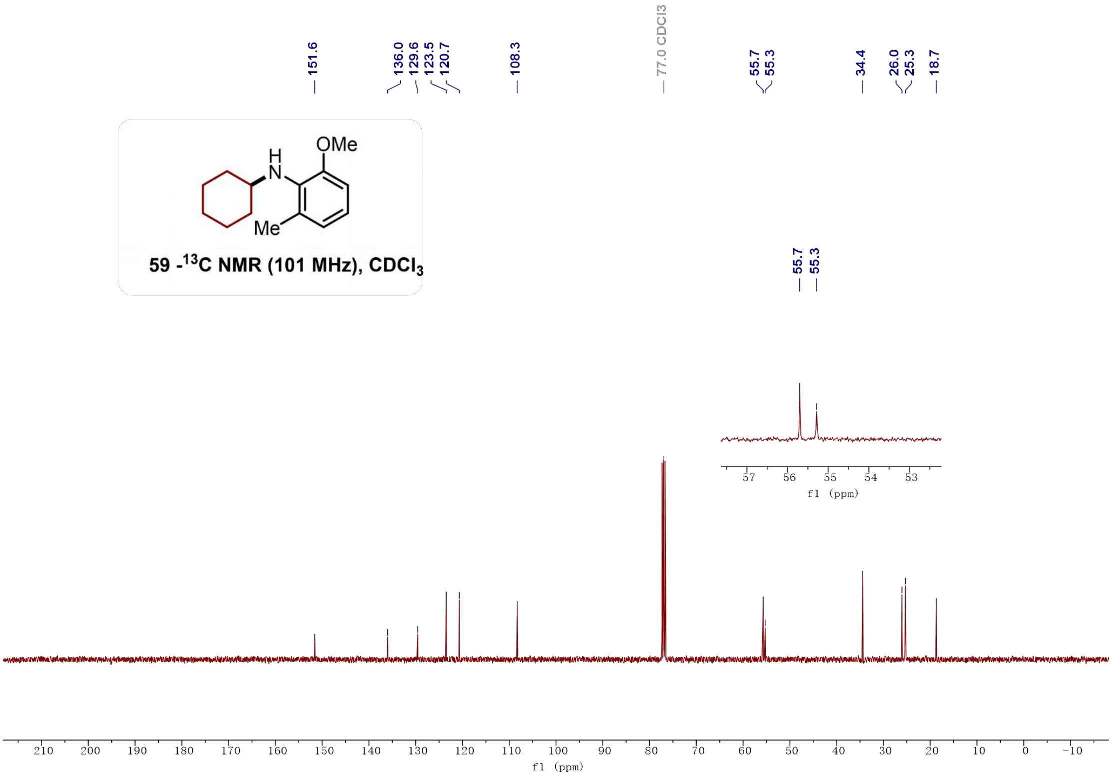

Supplementary Figure 174. ^{13}C NMR (101 MHz, $CDCl_3$) spectrum of compound **59**

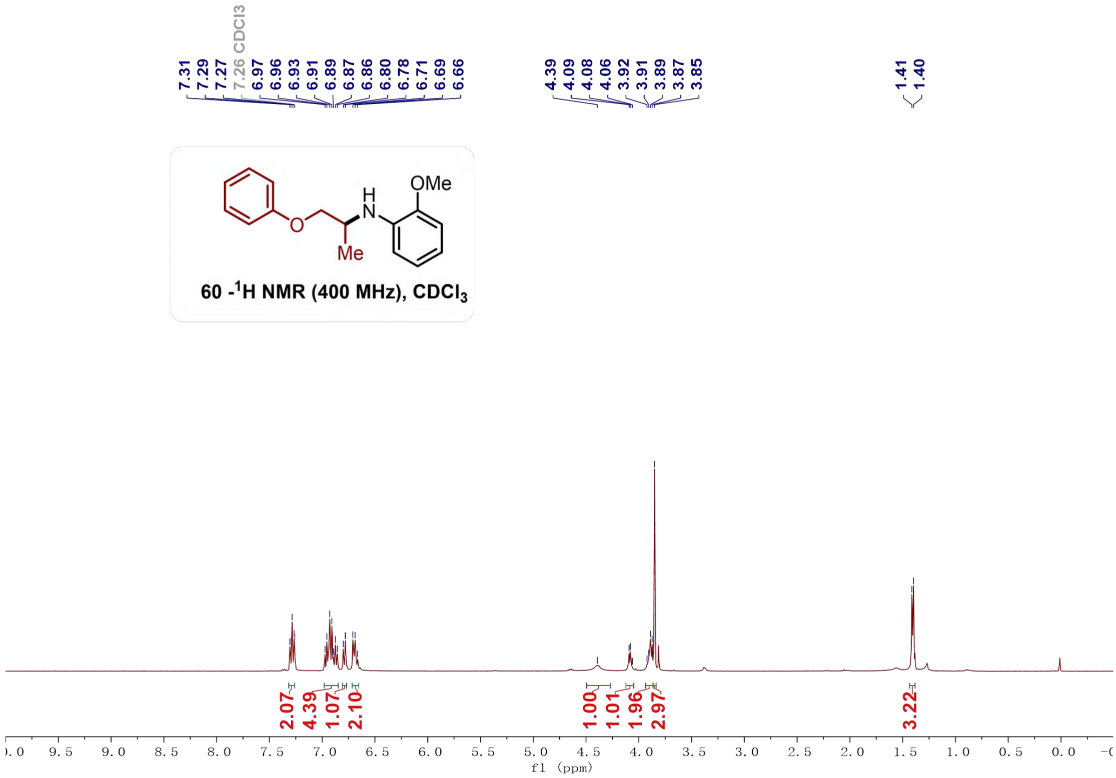

Supplementary Figure 175. ^{1}H NMR (400 MHz, $CDCl_3$) spectrum of compound **60**

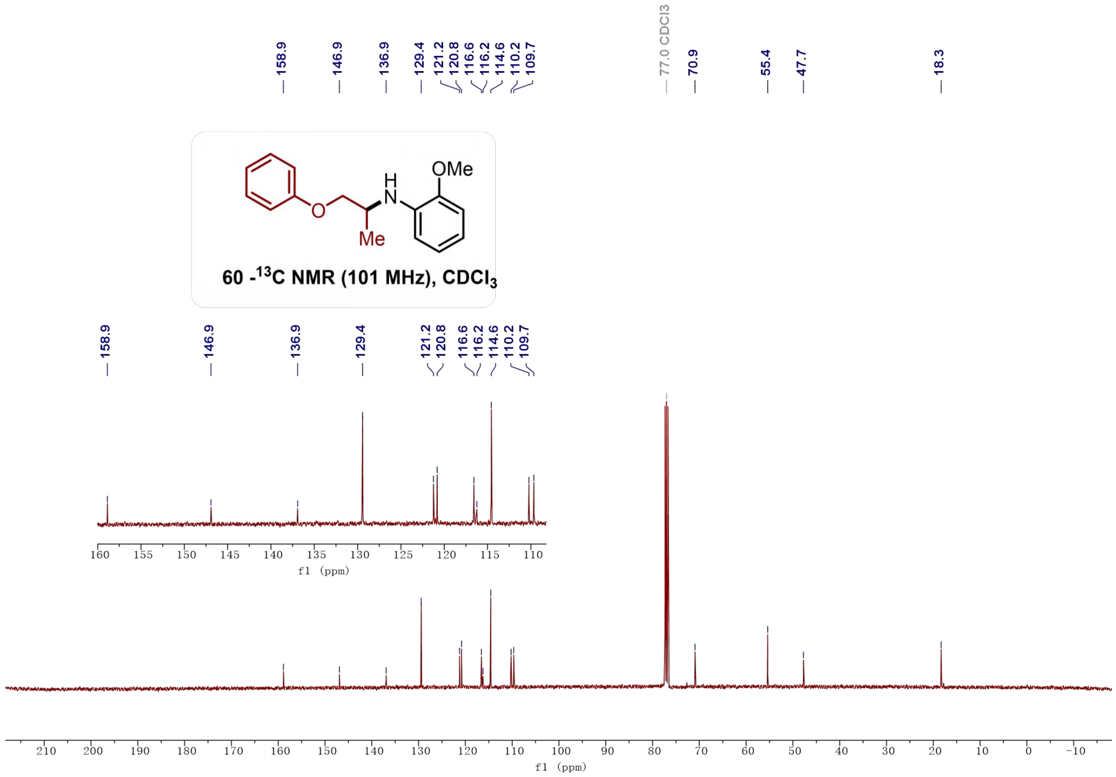

Supplementary Figure 176. ^{13}C NMR (101 MHz, $CDCl_3$) spectrum of compound **60**

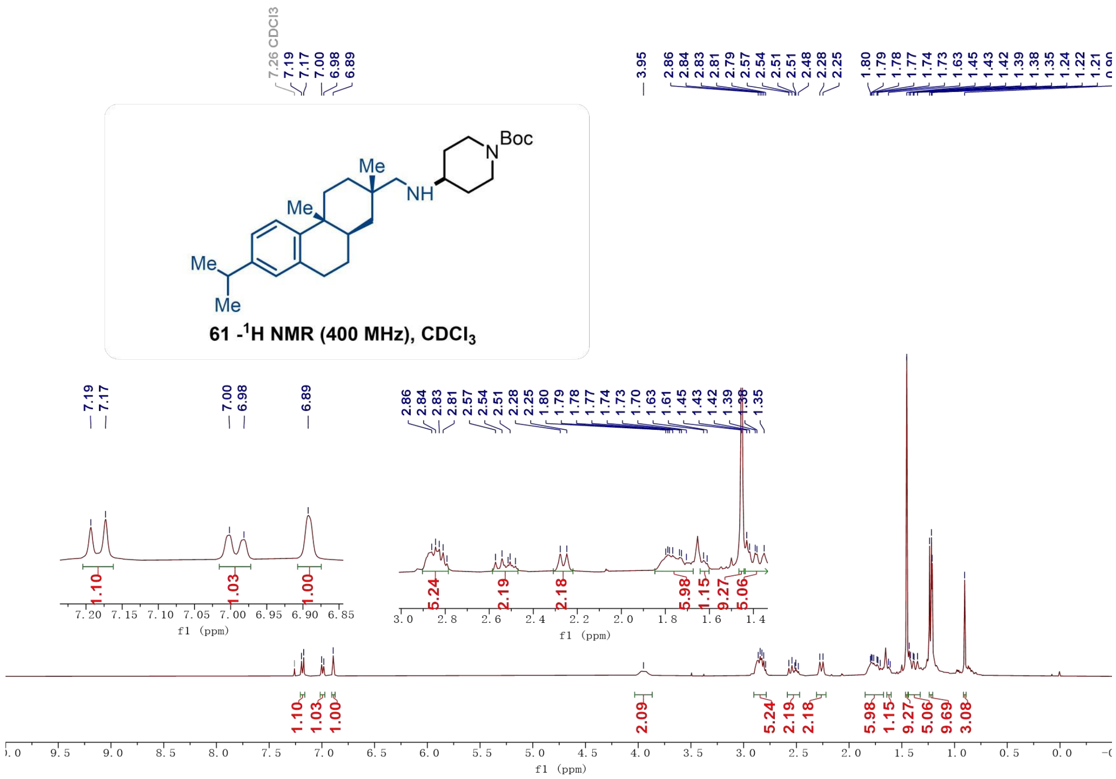

Supplementary Figure 177. ^{1}H NMR (400 MHz, $CDCl_3$) spectrum of compound **61**

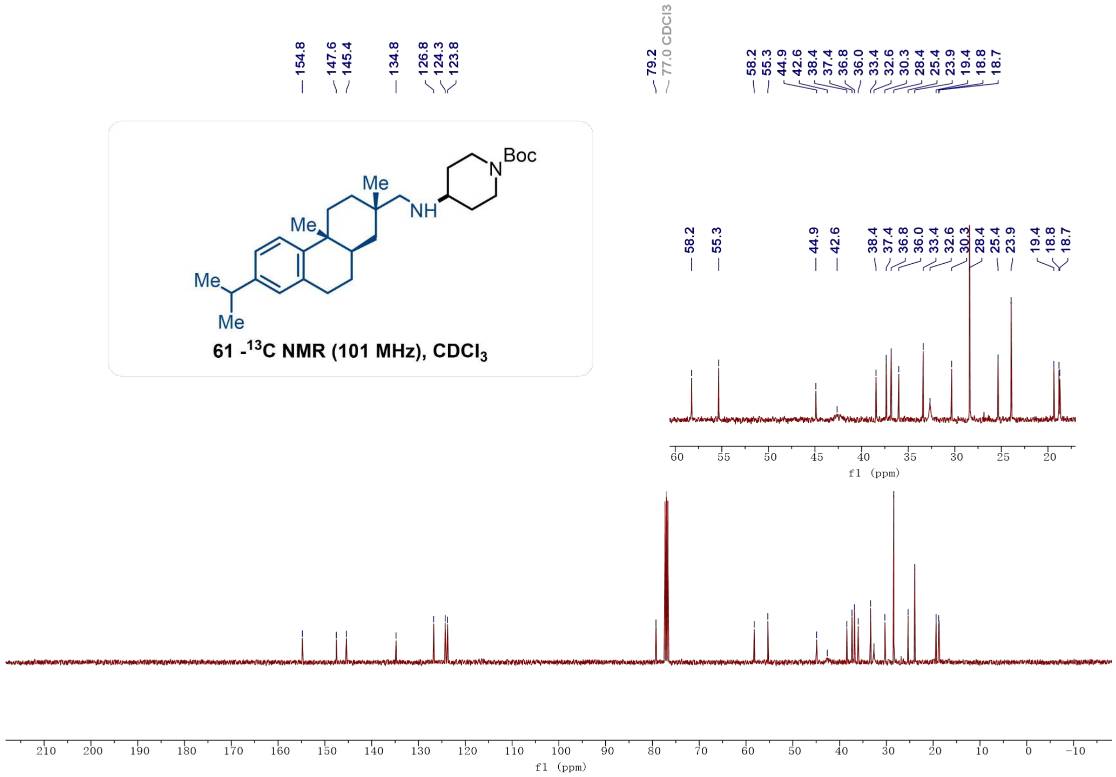

Supplementary Figure 178. ^{13}C NMR (101 MHz, $CDCl_3$) spectrum of compound **61**

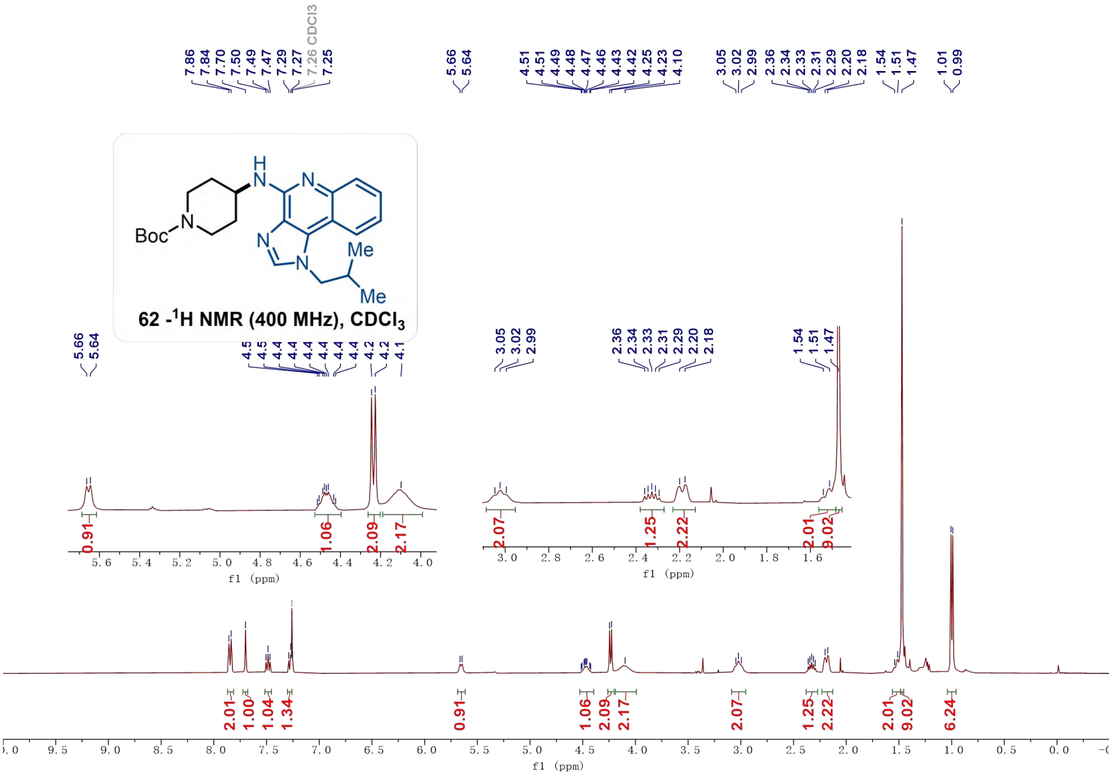

Supplementary Figure 179. ^{1}H NMR (400 MHz, $CDCl_3$) spectrum of compound **62**

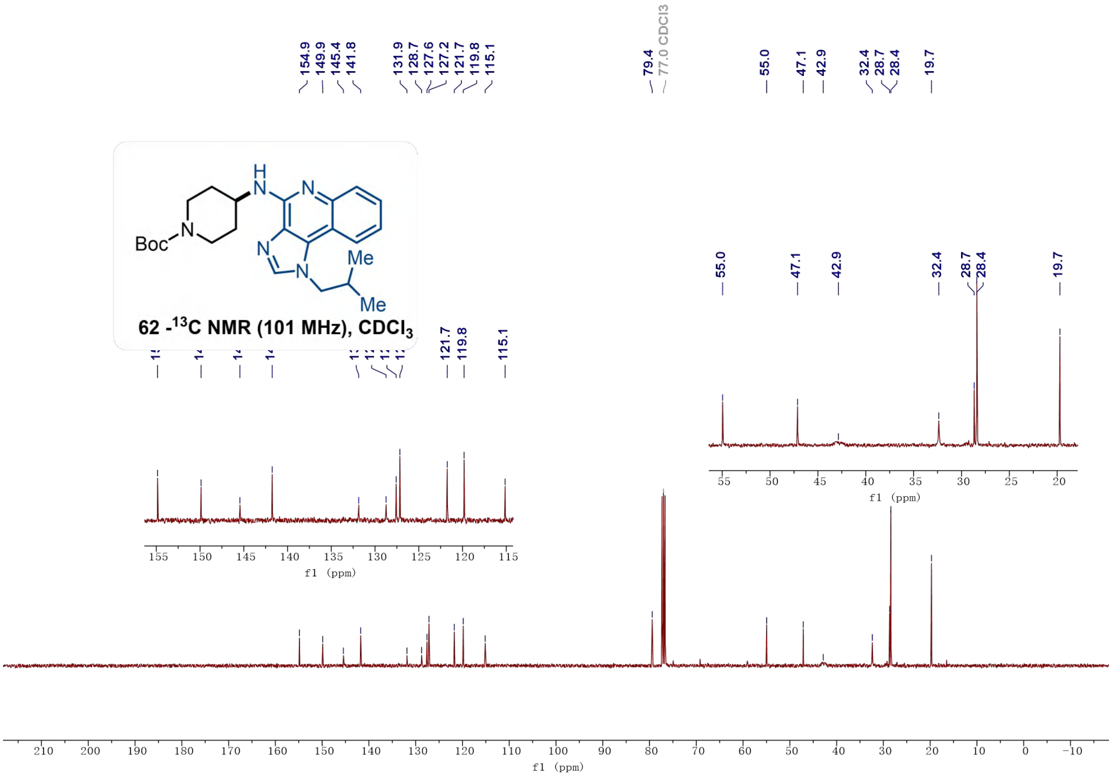

Supplementary Figure 180. ^{13}C NMR (101 MHz, $CDCl_3$) spectrum of compound **62**

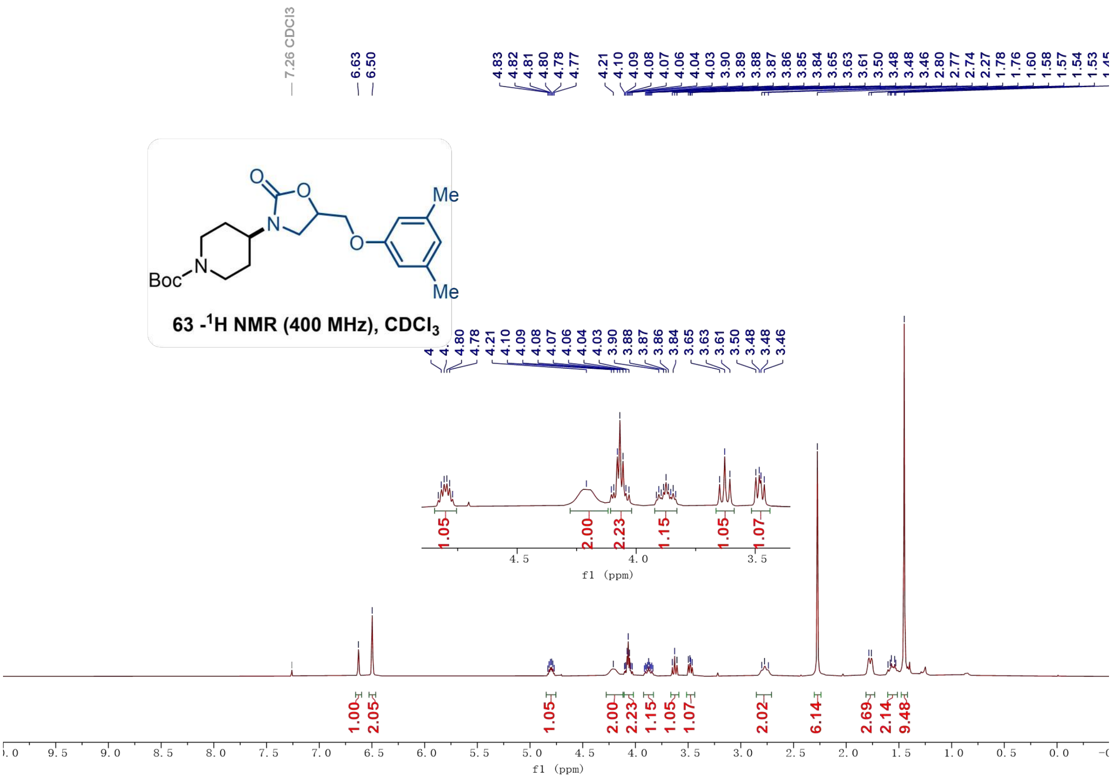

Supplementary Figure 181. ^{1}H NMR (400 MHz, $CDCl_3$) spectrum of compound **63**

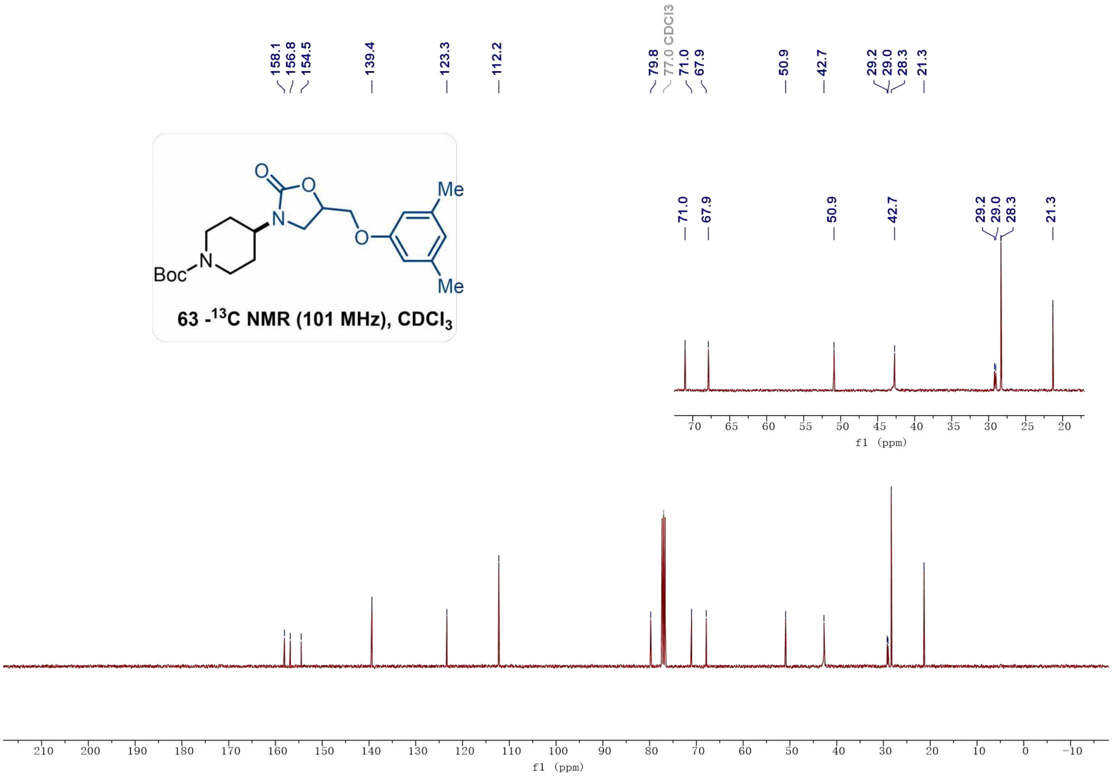

Supplementary Figure 182. ^{13}C NMR (101 MHz, $CDCl_3$) spectrum of compound **63**

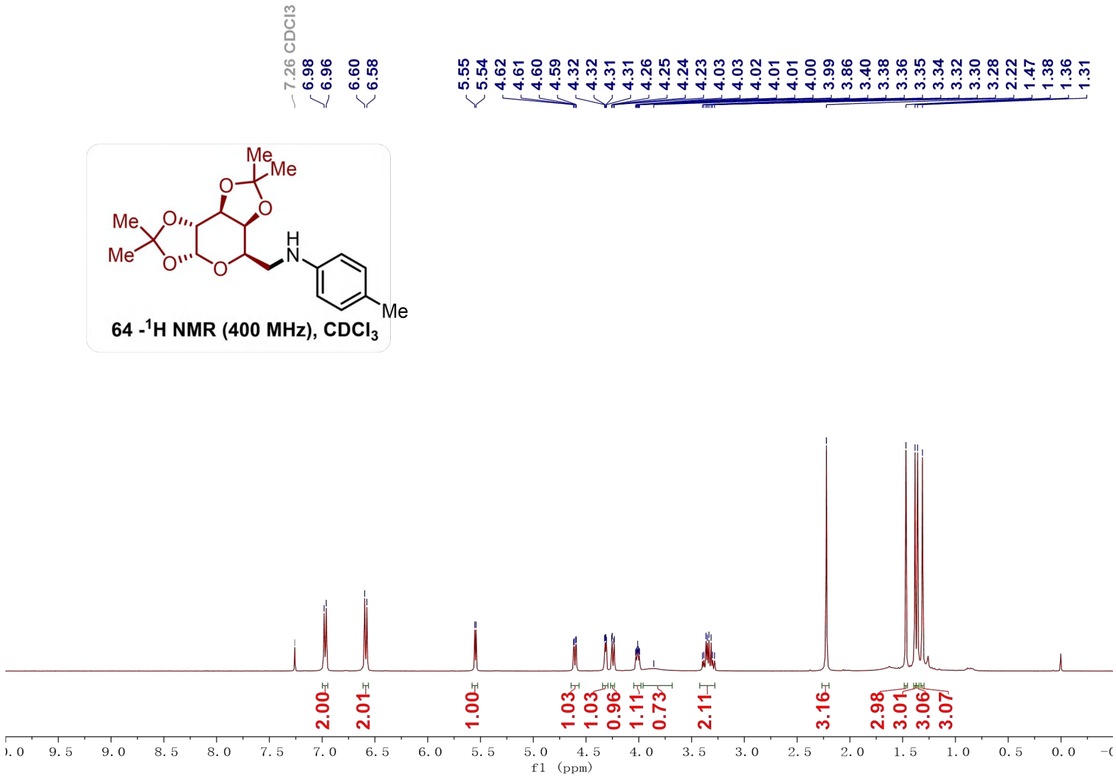

Supplementary Figure 183. ¹H NMR (400 MHz, $CDCl_3$) spectrum of compound **64**

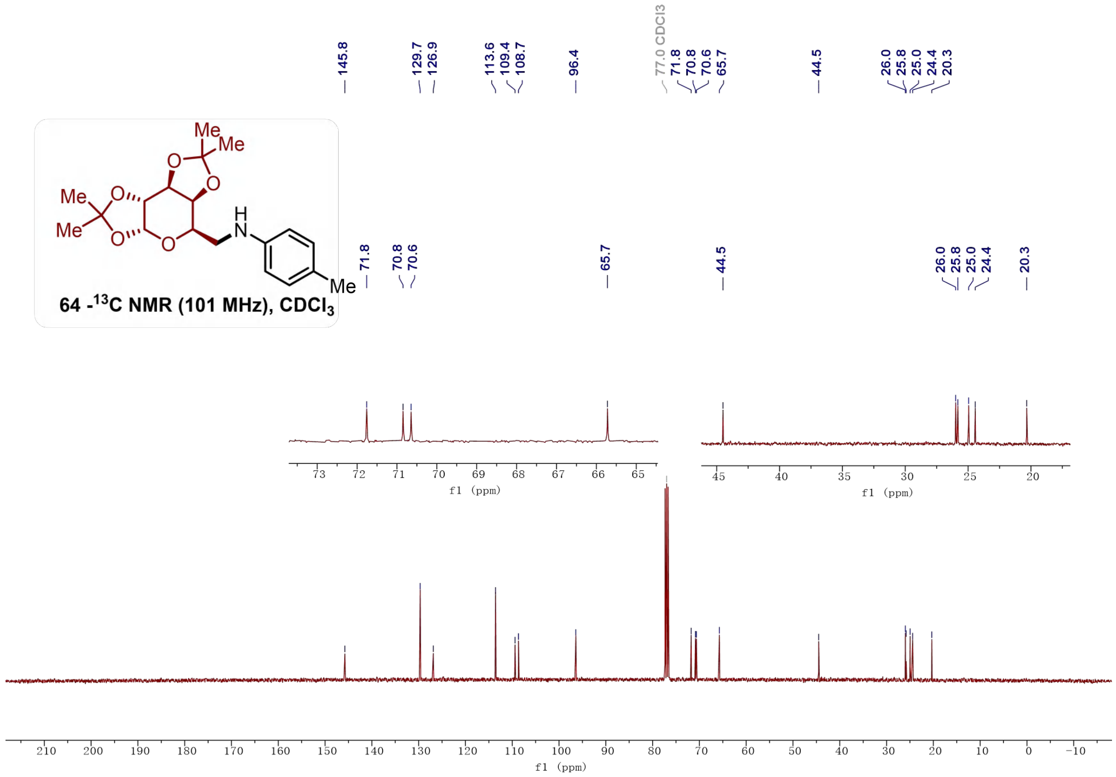

Supplementary Figure 184. ¹³C NMR (101 MHz, $CDCl_3$) spectrum of compound **64**

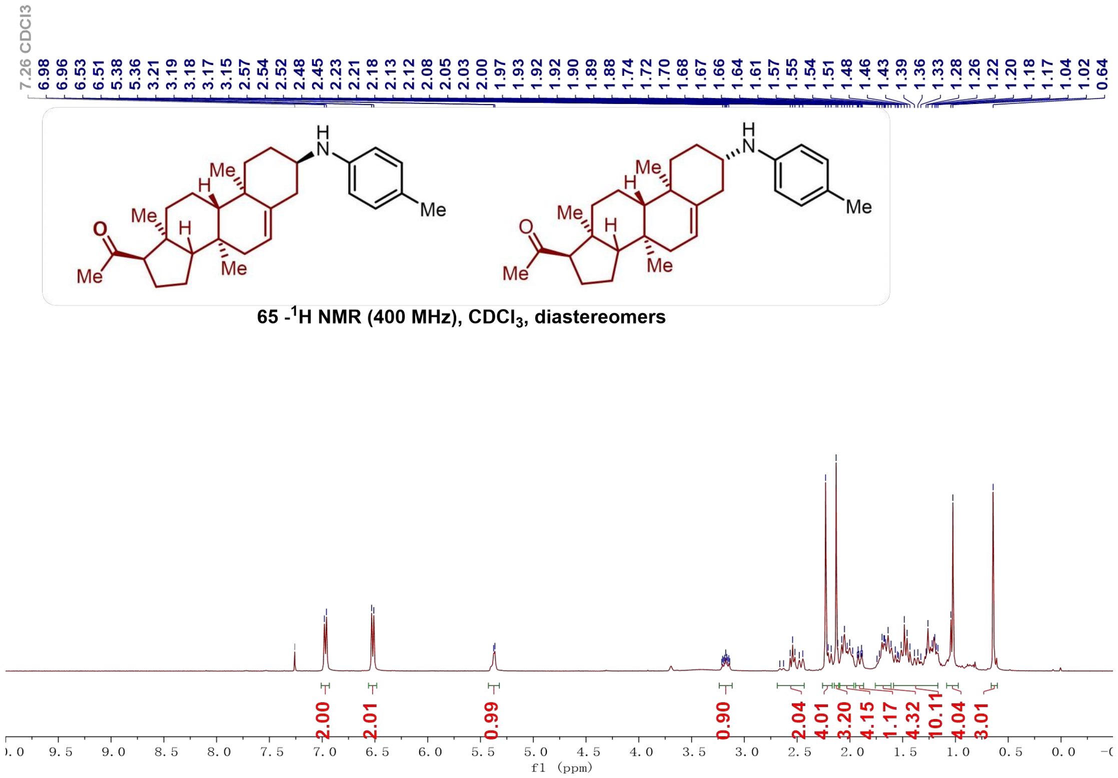

Supplementary Figure 185. ^{1}H NMR (400 MHz, $CDCl_3$) spectrum of compound **65**

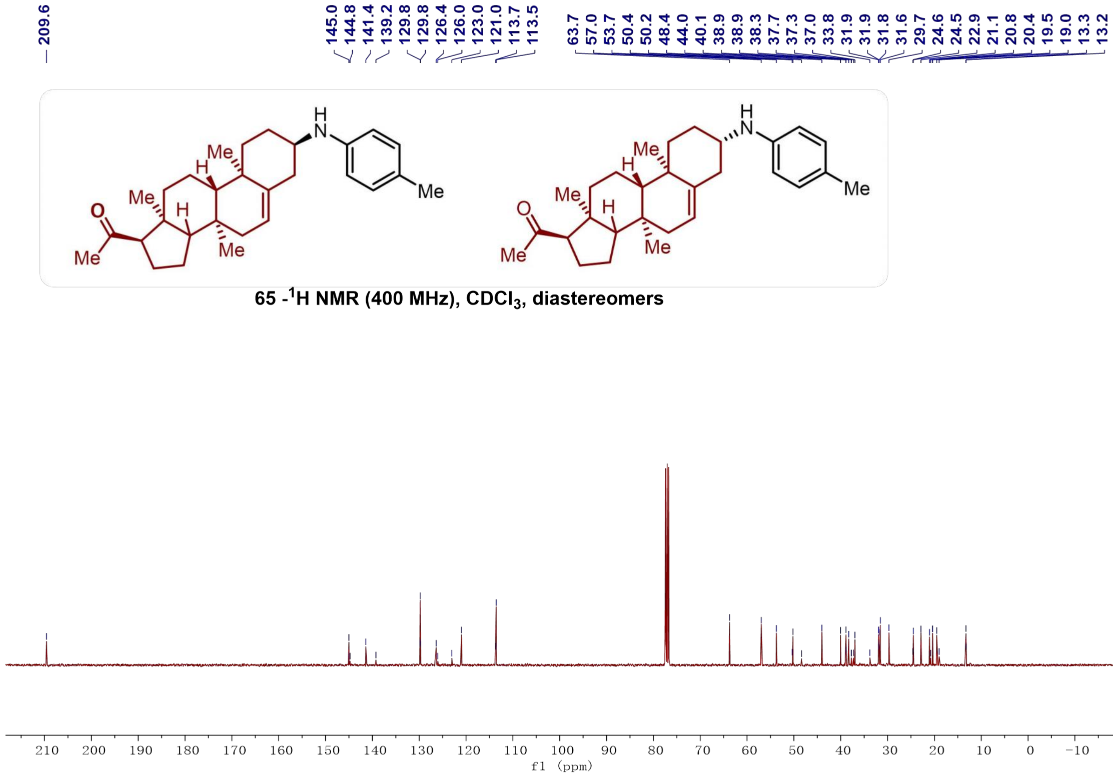

Supplementary Figure 186. ^{13}C NMR (101 MHz, $CDCl_3$) spectrum of compound **65**

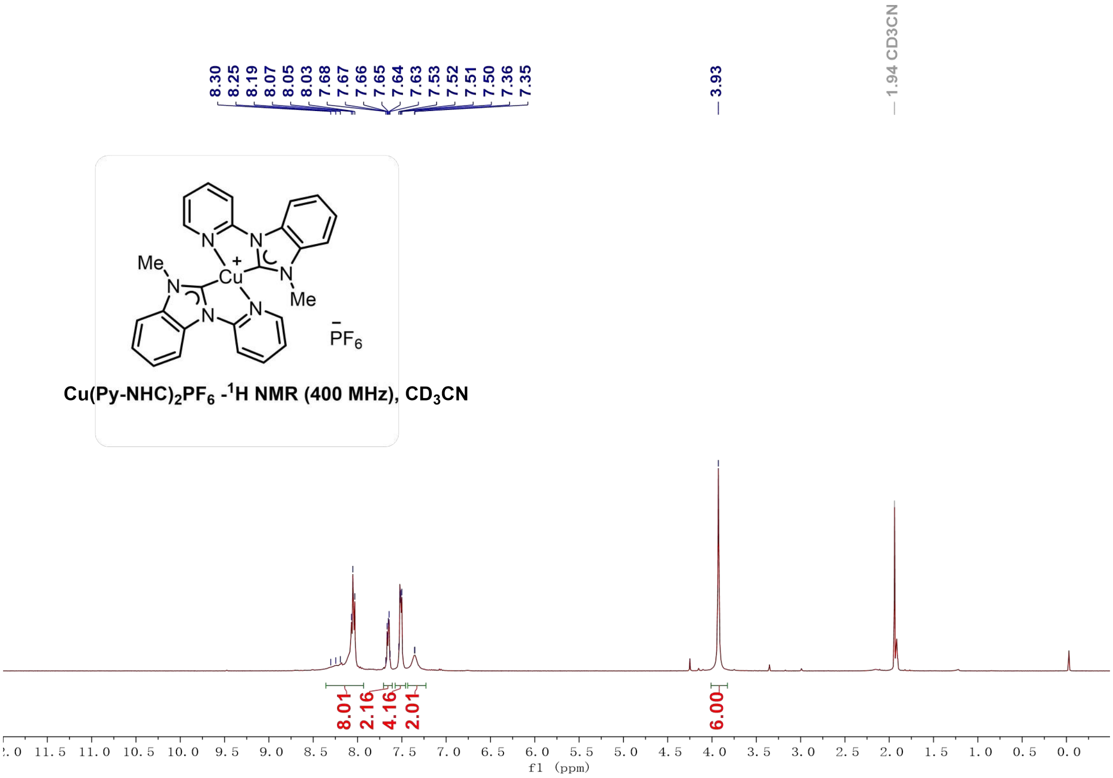

Supplementary Figure 187. ^{1}H NMR (400 MHz, CD_3CN) spectrum of **Cu(PyNHC)$_2$PF$_6$**

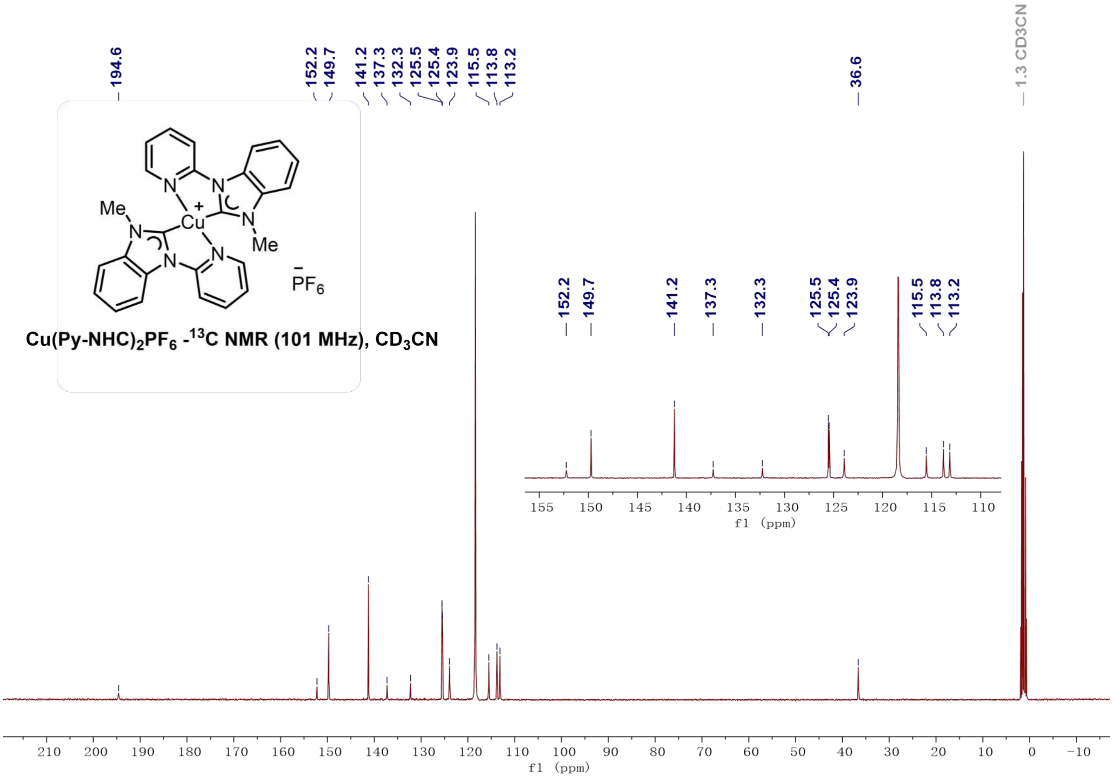

Supplementary Figure 188. ^{13}C NMR (101 MHz, CD_3CN) spectrum of compound **Cu(PyNHC)$_2$PF$_6$**

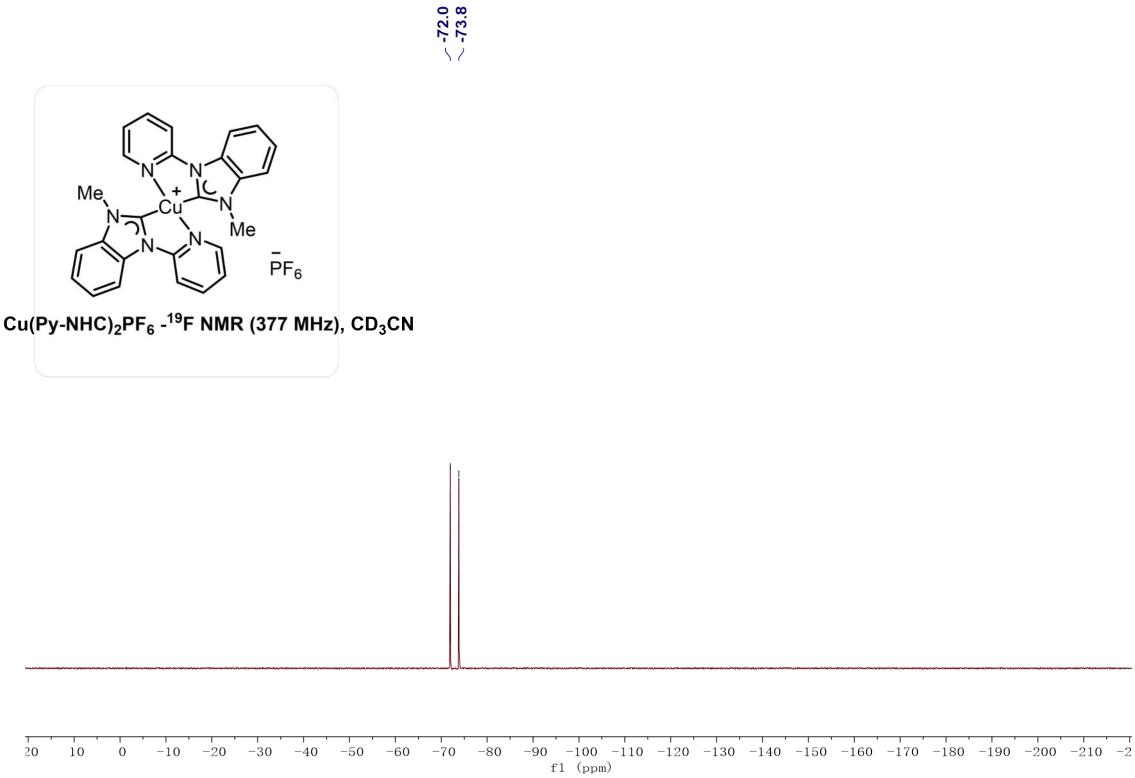

Supplementary Figure 189. ^{19}F NMR (377 MHz, CD_3CN) spectrum of compound **Cu(PyNHC)$_2$PF$_6$**

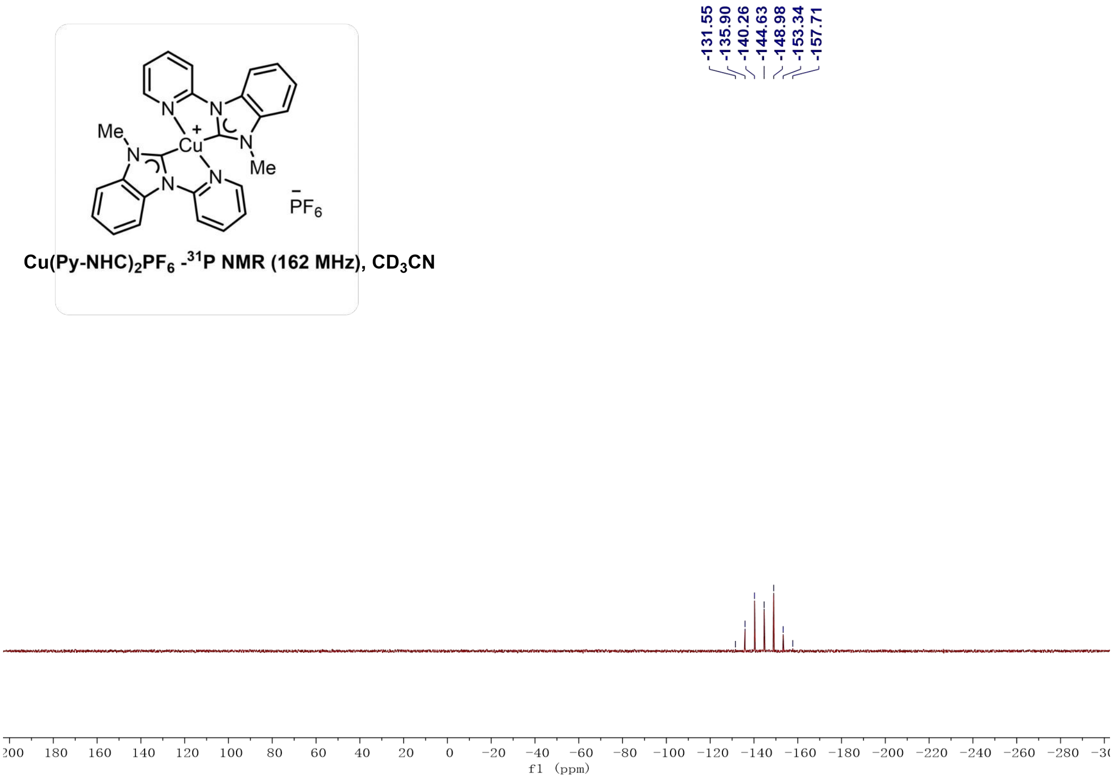

Supplementary Figure 190. ^{31}P NMR (162 MHz, CD_3CN spectrum of compound **Cu(PyNHC)$_2$PF$_6$**

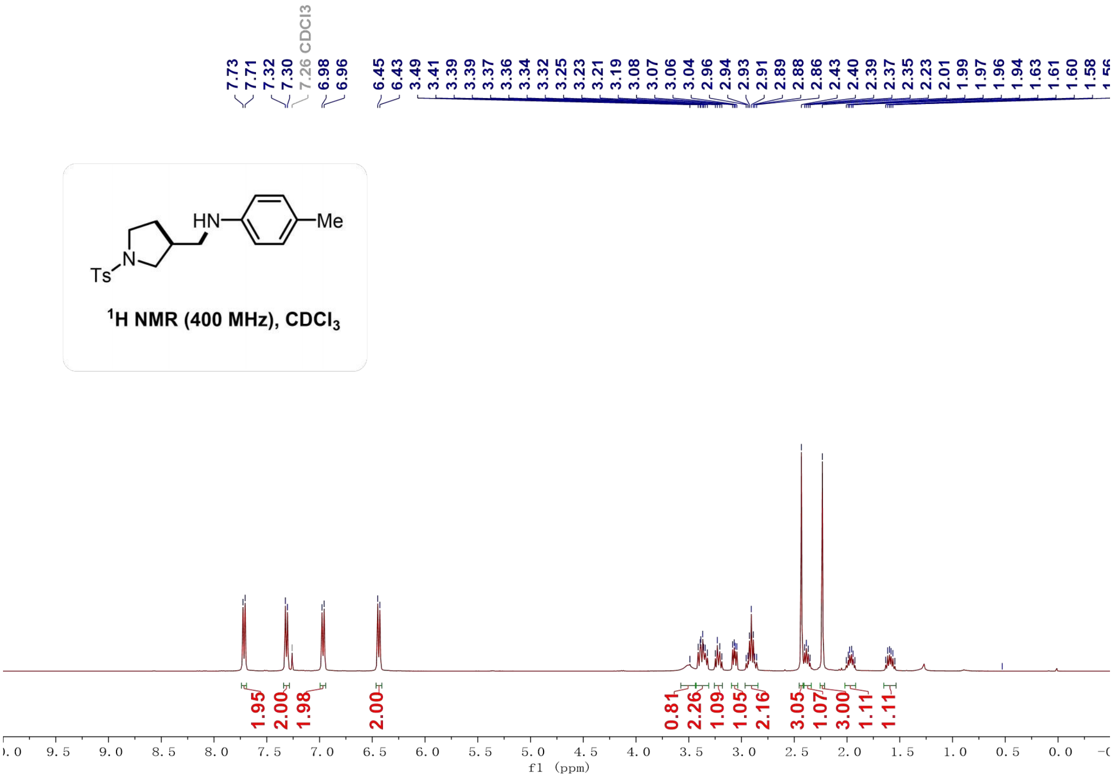

Supplementary Figure 191. ^{1}H NMR (400 MHz, $CDCl_3$) spectrum of compound **66**

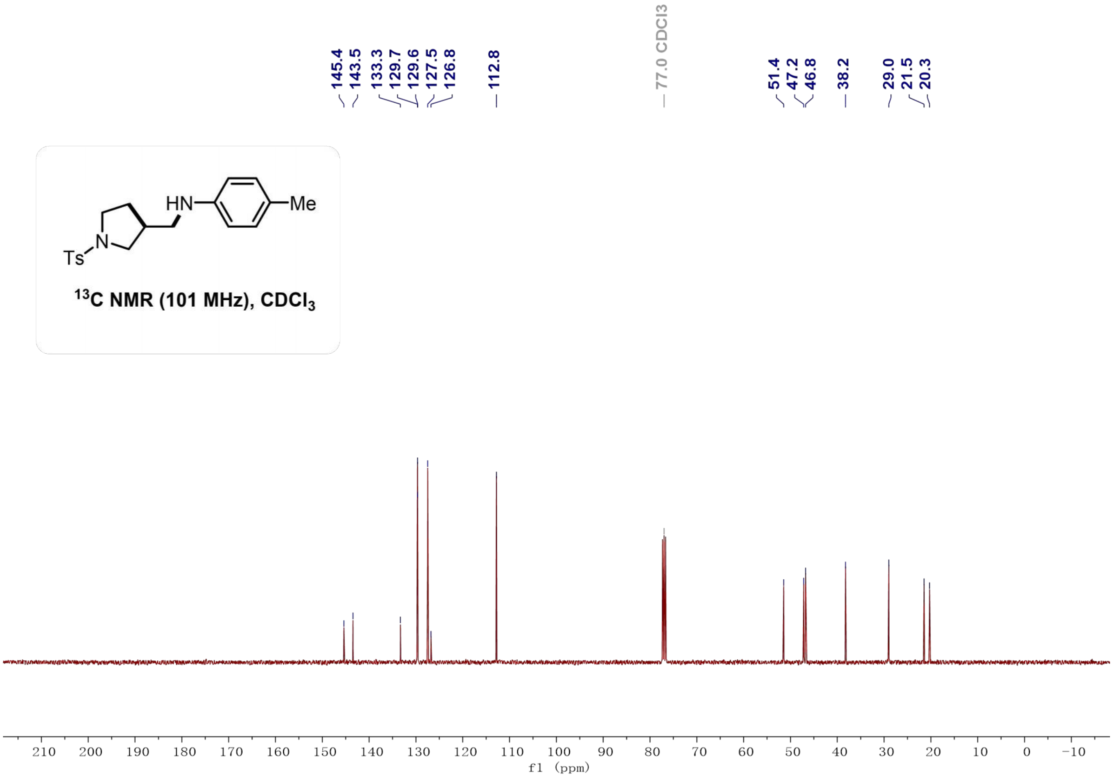

Supplementary Figure 192. ^{13}C NMR (101 MHz, $CDCl_3$) spectrum of compound **66**

Supplementary References

6. Reference

1. Buettner, C. S., Schnürch, M. & Bica-Schröder, K. Photocatalytic Hydroalkylation of Aryl-Alkenes. *J. Org. Chem.* **87**, 11042-11047 (2022).
2. Górski, B., Barthelemy, A.-L., Douglas, J. J., Juliá, F. & Leonori, D. Copper-catalysed amination of alkyl iodides enabled by halogen-atom transfer. *Nature Catalysis* **4**, 623-630 (2021).
3. Lu, X.-Y. *et al.* 1,1-Disubstituted olefin synthesis via Ni-catalyzed Markovnikov hydroalkylation of alkynes with alkyl halides. *Chem. Commun.* **52**, 5324-5327 (2016).
4. Matier, C. D., Schwaben, J., Peters, J. C. & Fu, G. C. Copper-Catalyzed Alkylation of Aliphatic Amines Induced by Visible Light. *J. Am. Chem. Soc.* **139**, 17707-17710 (2017).
5. Hara, R. *et al.* Boronic Acid Mediated Carbocyanation of Olefins and Vinylation of Alkyl Iodides. *Eur. J. Org. Chem.* **2018**, 4058-4063 (2018).
6. Koketsu, M., Kuberan, B. & Linhardt, R. J. Stereoselective Synthesis of the α-Glycoside of a KDO "C"-Disaccharide. *Org. Lett.* **2**, 3361-3363 (2000).
7. Xue, W., Qu, Z.-W., Grimme, S. & Oestreich, M. Copper-Catalyzed Cross-Coupling of Silicon Pronucleophiles with Unactivated Alkyl Electrophiles Coupled with Radical Cyclization. *J. Am. Chem. Soc.* **138**, 14222-14225 (2016).
8. Zhang, Z., Górski, B. & Leonori, D. Merging Halogen-Atom Transfer (XAT) and Copper Catalysis for the Modular Suzuki–Miyaura-Type Cross-Coupling of Alkyl Iodides and Organoborons. *J. Am. Chem. Soc.* **144**, 1986-1992 (2022).
9. Liu, Q. *et al.* Transition-Metal-Free Borylation of Alkyl Iodides via a Radical Mechanism. *Org. Lett.* **21**, 6597-6602 (2019).
10. Liu, Y., Xu, Y., Jung, S. H. & Chae, J. A Facile and Green Protocol for Nucleophilic Substitution Reactions of Sulfonate Esters by Recyclable Ionic Liquids. *Synlett* **23**, 2692-2698 (2012).
11. Liu, S. *et al.* Four-coordinate N-heterocyclic carbene (NHC) copper(I) complexes with brightly luminescence properties. *J. Coord. Chem.* **70**, 584-599 (2017).
12. Luo, W.-Q., Du, X.-G., Chen, L.-Y. & Jin, C.-M. Synthesis, structure, and anticancer activity of four silver(I)-N-heterocyclic carbene complexes and one polymer containing quinolin-8-yl groups. *J. Organomet. Chem.* **952**, 122033 (2021).
13. Rolt, A. *et al.* Discovery and Optimization of a 4-Aminopiperidine Scaffold for Inhibition of Hepatitis C Virus Assembly. *J. Med. Chem.* **64**, 9431-9443 (2021).
14. Ru bio-Perez, L., Florescence, F. J., Dharma, P., Velasquez, L. & Cabrera, A. Stable Preformed Choral Palladium Catalysts for the One-Pot Asymmetric Reductive Amination of Ketones. *Org. Lett.* **11**, 265-268 (2009).
15. Kuang, Y., Ning, Y., Zhu, J. & Wang, Y. Dirhodium(II)-Catalyzed (3 + 2) Cycloaddition of the N-Arylaminocyclopropane with Alkene Derivatives. *Org.*

Lett. **20**, 2693-2697 (2018).

16. Zheng, J., Huang, L., Huang, C., Wu, W. & Jiang, H. Synthesis of Polysubstituted Pyrroles via Pd-Catalyzed Oxidative Alkene C–H Bond Arylation and Amination. *J. Org. Chem.* **80**, 1235-1242 (2015).
17. Gaussian 16, Revision B.01, Frisch, M. J.; Trucks, G. W.; Schlegel, H. B.; Scuseria, G. E.; Robb, M. A.; Cheeseman, J. R.; Scalmani, G.; Barone, V.; Petersson, G. A.; Nakatsuji, H.; Li, X.; Caricato, M.; Marenich, A. V.; Bloino, J.; Janesko, B. G.; Gomperts, R.; Mennucci, B.; Hratchian, H. P.; Ortiz, J. V.; Izmaylov, A. F.; Sonnenberg, J. L.; Williams-Young, D.; Ding, F.; Lipparini, F.; Egidi, F.; Goings, J.; Peng, B.; Petrone, A.; Henderson, T.; Ranasinghe, D.; Zakrzewski, V. G.; Gao, J.; Rega, N.; Zheng, G.; Liang, W.; Hada, M.; Ehara, M.; Toyota, K.; Fukuda, R.; Hasegawa, J.; Ishida, M.; Nakajima, T.; Honda, Y.; Kitao, O.; Nakai, H.; Vreven, T.; Throssell, K.; Montgomery, J. A., Jr.; Peralta, J. E.; Ogliaro, F.; Bearpark, M. J.; Heyd, J. J.; Brothers, E. N.; Kudin, K. N.; Staroverov, V. N.; Keith, T. A.; Kobayashi, R.; Normand, J.; Raghavachari, K.; Rendell, A. P.; Burant, J. C.; Iyengar, S. S.; Tomasi, J.; Cossi, M.; Millam, J. M.; Klene, M.; Adamo, C.; Cammi, R.; Ochterski, J. W.; Martin, R. L.; Morokuma, K.; Farkas, O.; Foresman, J. B.; Fox, D. J. Gaussian, Inc., Wallingford CT, 2016.
